# Predicting Drugs Side Effects Based on Chemical-Chemical Interactions and Protein-Chemical Interactions

**DOI:** 10.1155/2013/485034

**Published:** 2013-09-04

**Authors:** Lei Chen, Tao Huang, Jian Zhang, Ming-Yue Zheng, Kai-Yan Feng, Yu-Dong Cai, Kuo-Chen Chou

**Affiliations:** ^1^College of Information Engineering, Shanghai Maritime University, Shanghai 201306, China; ^2^Key Laboratory of Systems Biology, Shanghai Institutes for Biological Sciences, Chinese Academy of Sciences, Shanghai 200031, China; ^3^Shanghai Center for Bioinformation Technology, Shanghai 200235, China; ^4^Department of Genetics and Genomic Sciences, Mount Sinai School of Medicine, New York City, NY 10029, USA; ^5^Department of Ophthalmology, Shanghai First People's Hospital, School of Medicine, Shanghai Jiao Tong University, Shanghai 200080, China; ^6^State Key Laboratory of Drug Research, Shanghai Institute of Materia Medica, Shanghai 201203, China; ^7^Beijing Genomics Institute, Shenzhen Beishan Industrial Zone, Shenzhen 518083, China; ^8^Institute of Systems Biology, Shanghai University, Shanghai 200444, China; ^9^Gordon Life Science Institute, Belmont, Massachusetts 02478, USA; ^10^Center of Excellence in Genomic Medicine Research (CEGMR), King Abdulaziz University, Jeddah, Saudi Arabia

## Abstract

A drug side effect is an undesirable effect which occurs in addition to the intended therapeutic effect of the drug. The unexpected side effects that many patients suffer from are the major causes of large-scale drug withdrawal. To address the problem, it is highly demanded by pharmaceutical industries to develop computational methods for predicting the side effects of drugs. In this study, a novel computational method was developed to predict the side effects of drug compounds by hybridizing the chemical-chemical and protein-chemical interactions. Compared to most of the previous works, our method can rank the potential side effects for any query drug according to their predicted level of risk. A training dataset and test datasets were constructed from the benchmark dataset that contains 835 drug compounds to evaluate the method. By a jackknife test on the training dataset, the 1st order prediction accuracy was 86.30%, while it was 89.16% on the test dataset. It is expected that the new method may become a useful tool for drug design, and that the findings obtained by hybridizing various interactions in a network system may provide useful insights for conducting in-depth pharmacological research as well, particularly at the level of systems biomedicine.

## 1. Introduction

Many drugs approved by Food and Drug Administration (FDA) were recalled each year after some unexpected side effects were discovered; for example, in 2010, Reductil/Meridia, Mylotarg, and Avandia were withdrawn. According to the “Drug Recall” (http://www.drugrecalls.com/drugrecalls.html), about 20 million people had taken the drugs in 1997 and 1998 that were later withdrawn. The drug side effects may have seriously harmful consequences to human beings [1]. For instance, the antiobesity drug fenfluramine/phentermine, also known as fen-phen, may cause heart disease and hypertension. Developing and producing drugs that were later found having serious side effects would be a disaster to a pharmaceutical company. For instance, the withdrawal of the aforementioned antiobesity drug has cost Wyeth more than $21 billion in America alone [2]. Therefore, it will not only avoid causing harm to patients but also avoid wasting lots of money if we can discover the side effects of a drug compound in the early phase of drug discovery.

Many efforts have been made in this regard, such as utilizing the drug perturbed gene expression profiles or biological pathways, to predict the side effects of drugs [1, 3–7], using chemical structures for the prediction of drugs side effects [8–10]. Although, most of the methods can only provide whether the query drug has some side effects, they cannot determine which side effects are most likely to happen or even the order information of the side effects. In this study, we proposed a novel computational method to predict the side effects of drugs based on chemical-chemical interaction and protein-chemical interaction. Compared to most of the previous studies, our method can provide the order information of the side effects, that is, prioritizing the side effects from the most likely one to the least likely one.

During the past decade, many compound databases have been constructed, such as KEGG (Kyoto Encyclopedia of Genes and Genomes) [11] and STITCH (Search Tool for Interactions of Chemicals) [12]. KEGG provides the information of chemical substances and reactions, while STITCH provides the interaction information of chemicals and proteins. Thus we can acquire the properties of many compounds and their other information from these databases. For those compounds not being covered by these databases, their properties can be inferred from the property-known compounds stored in the databases [13–16]. Likewise, the drugs side effects can also be inferred as elaborated below.

Recently, it was evidenced that interactive proteins are more likely to share common biological functions [17–20], and that interactive compounds are also more likely to share common biological functions [13, 16]. Since the side effects are part of biological functions of drugs, it would be feasible to use the chemical-chemical interactions to identify the drugs side effects. Unfortunately, some of the query drugs cannot be predicted for their side effects by this way because their interactive counterparts do not have any information of the side effects. To overcome such difficulty, we proposed to utilize the information of indirect interactions, including both the chemical-chemical interaction and the protein-chemical interaction, to identify the drugs side effects of which the direct chemical-chemical interaction data are not available. To evaluate the method, a benchmark dataset retrieved from SIDER [21] was constructed, which consisted of 835 drug compounds, and it was divided into one training dataset and one test dataset. By a jackknife test on the training dataset, the 1st order prediction accuracy was 86.30%, while it was 89.16% on the test dataset. To confirm the effectiveness of the method, another method based on chemical structure similarity obtained by SMILES string [22] was also conducted on the training and test datasets. Encouraged by the good performance of the method and superiority to the method based on chemical structure similarity, we hope that the proposed method can become a useful tool to predict drugs side effects and screen out drugs with undesired side effects.

## 2. Materials and Methods

### 2.1. Benchmark Dataset

The benchmark dataset used in the current study was downloaded from SIDER [21] at http://sideeffects.embl.de/, which integrated the side effects of 888 drugs from the US Food and Drug Administration (FDA) and other sources [21]. To obtain a high-quality, well-defined benchmark dataset, the data were collected strictly according to the following criteria: (i) only the 100 side effects with most drugs listed in SIDER and the corresponding drugs were included, and (ii) drugs without both chemical-chemical interactions and protein-chemical interactions were also excluded. Finally, we obtained a benchmark dataset** S** that contained 835 drugs belonging to 100 categories of side effects. The codes of the 835 drugs in each of the 100 side effect categories are given in Supplementary Material I available online at http://dx.doi.org/10.1155/2013/485034.

For the convenience of later formulation, let us use the symbols *C*_1_, *C*_2_, *C*_3_,…, *C*_100_ to tag the 100 side effects, where *C*_1_ represents “Nausea,” *C*_2_ “Headache,” *C*_3_ “Vomiting,” and so forth, as described in the table in Supplementary Material II, in which the number of drugs with each of the 100 side effect tags is also given. Thus, the benchmark dataset **S** can be formulated as

(1) $$\begin{array}{c} S\;=\;S_{1}\cup S_{2}\cup \cdots \cup S_{100}, \end{array}$$

where **S**_*i*_ represents the subset that contains the drugs with the side effect *C*_*i*_ (*i* = 1,2,…, 100).

Since many drugs in **S** have multiple side effects that is, they may simultaneously occur in subsets with different side effect tags, it is instructive to introduce the concept of “virtual drug” sample, as illustrated as follows. A drug compound coexisting at two different side effect subsets will be counted as 2 virtual drugs even though they have an identical chemical structure, if coexisting at three different subsets, 3 virtual drugs; and so forth. Accordingly, the total structure-different drug compounds and the total number of the side-effect-different virtual drug compounds can be described by the following equation:

(2) $$\begin{array}{c} N\left(\text{str}\right)=835,\\ N\left(\text{vir}\right)=\sum_{i=1}^{100}N\left(C_{i}\right)=30,114, \end{array}$$

where *N*(str) is the number of the total structure-different drug compounds, *N*(vir) the number of the total side-effect-different virtual drug compounds in **S**, and *N*(*C*_*i*_) the number of drugs with the side-effect tag *C*_*i*_. Substituting the numbers of *N*(*C*_*i*_) (*i* =  1, 2,…, 100) in the table in Supplementary Material II into (2), we obtained *N*(vir) = 30,114 fully consistent with the results in (2) and the table in Supplementary Material II.

**Figure 1:**
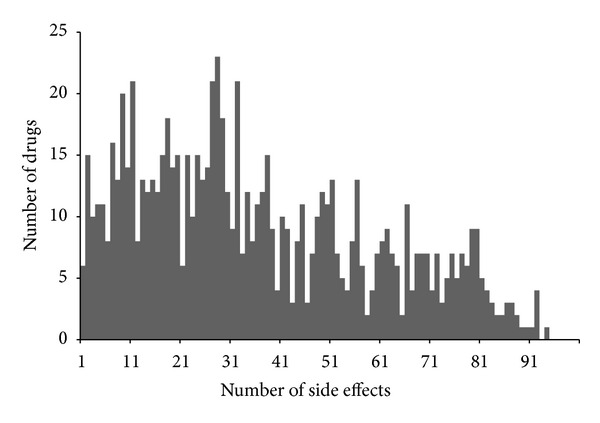
A histogram of the number of drugs versus the number of side effects.

It can be seen from (2) that the total number of the side-effect-different virtual drug compounds is much greater than that of the total structure-different drug compounds. To provide an intuitive view about their distribution, a histogram of the number of drugs versus the number of side effects is given in Figure 1, from which we can see that, of the 835 drugs, only 6 have one side effect while the majority has more than 10 side effects. Thus, the prediction of drugs side effects is a multilabel classification problem. Like the case in dealing with compounds with multiple properties [13, 16], the proposed method would provide the order information of side effects from the most likely to the least likely.

To evaluate the methods as described below sufficiently, we randomly selected 10% (83) samples from **S** to compose the test dataset, denoted by **S**_te_, while the remaining 752 samples in **S** were used to construct the training dataset, denoted by **S**_tr⁡_.

### 2.2. Chemical-Chemical Interactions and Protein-Chemical Interactions

It was evidenced that interactive proteins are more likely to share common biological functions than noninteractive ones [17–20]. Likewise, it has been indicated by some pioneer studies [13, 16] that interactive compounds follow the similar rules. Since side effect is one part of biological functions of drugs, using the properties of interactive compounds to identify drugs side effects is a feasible scheme.

To obtain the information of interactive compounds, we downloaded the data of chemical-chemical interactions from STITCH (http://stitch.embl.de/, chemical_chemical.links.detailed.v3.0.tsv.gz) [12], a well-known database containing known and predicted chemical-chemical interaction and protein-chemical interaction data from experiments, literature, or other reliable sources. In the datafile obtained each interaction unit contains two chemicals and five scores with titles “Similarity,” “Experimental,” “Database,” “Textmining,” and “Combined_score,” respectively. Since the last score combines the information of other scores, we utilized the last score to indicate the interactivity of two chemicals in this study; that is, compounds in the interaction unit with “Combined_score” greater than zero were deemed to be interactive compounds. The interactive compounds thus considered here satisfy one of the following three properties: (I) they participate in the same reactions; (II) they share similar structures or activities; (III) they have literature associations. These three properties always indicate that the interactive compounds occupy the same biological pathways, suggesting they may induce similar side effects. It is confirmed that using chemical-chemical interactions retrieved from STITCH to identify drugs side effects is feasible. The “Combined_score” is termed as confidence score, because its value always indicates the likelihood that two interactive compounds can interact in a way that two compounds with high “Combined_score” mean that they can interact with high probability. For any two-drug compounds *d*_1_ and *d*_2_, their interaction confidence score was denoted by *Q* ^*c*^(*d*_1_, *d*_2_). Particularly, if the interaction between *d*_1_ and *d*_2_ did not exist, their interaction confidence score was set to zero; that is, *Q* ^*c*^(*d*_1_, *d*_2_) = 0.

Since the data of chemical-chemical interactions in STITCH is not very complete at present; that is, some potential chemical-chemical interactions may not be reported in STITCH, predicted methods based on chemical-chemical interactions may have a limitation that samples without interactive counterparts in the training dataset cannot be processed. Thus, it is necessary to give some new schemes to measure the interactions that are not reported in STITCH. It is known that if two drug compounds can interact with a third compound or protein, these two drug compounds are likely to share some common functions. In view of this, we proposed a new scheme to measure the likelihood of interaction of two chemicals based on indirect chemical-chemical and protein-chemical interactions.

The data for the protein-chemical interactions were also downloaded from STITCH (http://stitch.embl.de/, protein_chemical.links.detailed.v3.0.tsv.gz). Each of the interaction units in the datafile obtained contains one compound, one protein, and four scores with titles “Experimental,” “Database,” “Textmining,” and “Combined_score,” respectively. With the similar argument, we used the value of “Combined_score,” also termed as confidence score, to indicate the likelihood of the interaction's occurrence. For one protein *p* and one drug compound *d*, their interaction confidence score was denoted as *Q* ^*p*^(*p*, *d*). If there was no interaction at all between the protein *p* and the drug *d*, it was also set to zero; that is, *Q* ^*p*^(*p*, *d*) = 0.

Now, we are ready to introduce the new scheme to measure the likelihood of interaction of two chemicals. For two compounds *d*_1_ and *d*_2_, suppose *I* ^*c*^(*d*_1_) denote a set of compounds that are directly interacting with the drug *d*_1_ and *I* ^*c*^(*d*_2_) a set of compounds directly interacting with the drug *d*_2_, formulated as

(3) $$\begin{array}{c} I^{c}\left(d_{1}\right)=\left\{d:Q^{c}\left(d,d_{1}\right)>0\right\},\\ I^{c}\left(d_{2}\right)=\left\{d:Q^{c}\left(d,d_{2}\right)>0\right\}. \end{array}$$

In a similar way let *I* ^*p*^(*d*_1_) denote a set of proteins that are directly interacting with the drug *d*_1_ and *I* ^*p*^(*d*_2_) a set of proteins directly interacting with the drug *d*_2_, formulated as

(4) $$\begin{array}{c} I^{p}\left(d_{1}\right)=\left\{p:Q^{p}\left(p,d_{1}\right)>0\right\},\\ I^{p}\left(d_{2}\right)=\left\{p:Q^{p}\left(p,d_{2}\right)>0\right\}. \end{array}$$

According to the set theory, the drug compounds that are interacting with both the drug *d*_1_ and the drug *d*_2_ should be the intersection of the set *I* ^*c*^(*d*_1_) and the set *I* ^*c*^(*d*_2_); that is, they will form a set given by

(5) $$\begin{array}{c} I^{c}\left(d_{1},d_{2}\right)=I^{c}\left(d_{1}\right)\cap I^{c}\left(d_{2}\right). \end{array}$$

Likewise, the human proteins that are interacting with both the drug *d*_1_ and the drug *d*_2_ should be the intersection of the set *I* ^*p*^(*d*_1_) and the set *I* ^*p*^(*d*_2_); that is, they will form a set given by

(6) $$\begin{array}{c} I^{p}\left(d_{1},d_{2}\right)=I^{p}\left(d_{1}\right)\cap I^{p}\left(d_{2}\right). \end{array}$$

Thus, the likelihood of the interaction between *d*_1_ and *d*_2_ can be calculated via the following equation:

(7) Qh(d1,d2)=(∑d′∈Ic(d1,d2)(Qc(d1,d′)+Qc(d2,d′))+∑p′∈Ip(d1,d2)(Qp(p′,d1)+Qp(p′,d2)))×(2|Ic(d1,d2)∪Ip(d1,d2)|)−1,

where ∈ is a symbol in the set theory meaning “member of.” 

### 2.3. Interaction-Based Method

It is instructive to recall that by using the information of protein-protein interactions, some methods have been developed to successfully predict the properties of proteins [17–20, 23]. Actually, the underlying idea of these methods was based on the assumption that interactive proteins are more likely to share common biological functions than noninteractive ones. Similarly, based on the argument in Section 2.2 and some previous studies [13, 16], interactive drugs are more likely to share similar side effects than noninteractive ones. Based on such an underlying idea, the following predicted method based on chemical-chemical and protein-chemical interactions was developed.

For convenience, some notations are necessary. Suppose there are *n* drugs in the training set **S**′, say *d*_1_, *d*_2_, …, *d*_*n*_; the side effects of the drug *d*_*i*_ in the training dataset is described as

(8) $$\begin{array}{c} C\left(d_{i}\right)={\left[c_{i,1},c_{i,2},\ldots ,c_{i,100}\right]}^{T}\;\left(i=1,2,\ldots ,n\right), \end{array}$$

where **T** is the transpose operator and

(9) $$\begin{array}{c} c_{i,j}=\{\begin{array}{cc} 1, & \text{if}\;d_{i}\;\text{has}\;\text{the}\;\text{side}\;\text{effect}\;C_{j},\\ 0, & \text{otherwise}. \end{array} \end{array}$$

*Prediction Based on Chemical-Chemical Interactions.* As elaborated previously, interactive drugs always share similar side effects. The likelihood that the query drug *d* has the side effect *C*_*j*_ can be calculated by

(10) $$\begin{array}{c} \prod_{}^{c}\left(d\to C_{j}\right)=\sum_{d_{i}\in S^{\prime }}Q^{c}\left(d,d_{i}\right)\cdot c_{i,j}\;j=1,2,\ldots ,100. \end{array}$$

According to (10), the greater score ∏^*c*^(*d* → *C*_*j*_) means that there are lots of interactive compounds of *d* that have the side effect *C*_*j*_ or some interactions between *d* and its interactive compounds with the side effect *C*_*j*_ are labeled by high confidence scores. Thus, the greater the score ∏^*c*^(*d* → *C*_*j*_) is, the more likely the drug compound *d* has the *j*th side effect, with ∏^*c*^(*d* → *C*_*j*_) = 0 indicating that the probability for the drug *d* having the *j*th side effect is zero. Since a drug usually has multiple side effects (see Figure 1), the prediction should provide a series of candidate side effects ranging from the most likely one to the least likely one, rather than only giving the most likely one. Thus, for a query drug *d*, suppose we have

(11) ∏c(d→C2)≥∏c(d→C4)≥⋯≥∏c(d→C90)>0

meaning that the highest likelihood of side effect for the drug *d* is *C*_2_ or “Headache” (cf. table in Supplementary Material II), and the second highest is *C*_4_ or “Rash”, and so forth. In other words, *C*_2_ is called the 1st order prediction, *C*_4_ the 2nd order prediction, and so forth. Note that the outcome of (10) might be trivial; that is,

(12) $$\begin{array}{c} \prod_{}^{c}\left(d\to C_{j}\right)=0\;\text{for}\;j=1,2,\ldots ,100 \end{array}$$

implying that no meaningful or direct interactive drug compounds whatsoever can be found in the training dataset **S**′ for the drug *d*. Under such a circumstance, an alternative approach should be used for predicting its side effects, as elaborated below.

*Prediction Based on Hybrid Interactions.* When the query drug *d* did not have any directly interactive drugs in the training dataset **S**′ or the information of its directly interactive drugs was trivial, the data for the indirect chemical-chemical and protein-chemical interactions would be used to predict its side effects. The prediction method was formulated in a similar way as the above method. But now instead of (10), the likelihood that the query drug *d* has the side effect *C*_*j*_ should be calculated by

(13) $$\begin{array}{c} \prod_{}^{h}\left(d\to C_{j}\right)=\sum_{d_{i}\in S^{\prime }}Q^{h}\left(d,d_{i}\right)\cdot c_{i,j}. \end{array}$$

By integrating the above two different approaches, the following steps were adopted to predict the side effects of the query drug *d*.

**Step 1**

The method based on the chemical-chemical interactions; that is, (10), was first utilized to identify its side effects.

**Step 2**

If the outcomes were trivial or no meaningful results were obtained as in the case of (12), the method based on the hybrid interactions, that is, (13), would be utilized to continue the prediction.

### 2.4. Similarity-Based Method

It is known that the compounds with similar structural properties always involve in similar biological activities [24]. The most well-known representing system to obtain the similarity information of two compounds is SMILES (Simplified Molecular Input Line Entry System) [22], which is a line notation for representing molecules and reactions using ASCII strings. Here, we also used this system to obtain the representations of compounds, which were used to calculate the similarity score of two compounds and set up a new computational method to identify drugs side effect. The similarity score between two compounds with their SMILES representations can be obtained from Open Babel [25] that is an open chemical toolbox. For two-drug compounds *d*_1_ and *d*_2_, their similarity score obtained from Open Babel was denoted by *Q* ^*s*^(*d*_1_, *d*_2_). Based on the fact that the compounds with similar structural properties always share the same biological activities, the likelihood that the query drug *d* has the side effect *C*_*j*_ can be calculated by

(14) $$\begin{array}{c} \prod_{}^{s}\left(d\to C_{j}\right)=\underset{d_{i}\in S^{\prime }}{\max}Q^{s}\left(d,d_{i}\right)\cdot c_{i,j} \end{array}$$

meaning that the likelihood that the query drug *d* has the side effect *C*_*j*_ is formulated as the maximum similarity scores between *d* and those drugs with side effect *C*_*j*_ in the training dataset **S**′. Obviously, the greater the score ∏^*s*^(*d* → *C*_*j*_), the more likely the drug compound *d* has the side effect *C*_*j*_. Following the similar procedure of the method based on chemical-chemical interactions, we can also obtain the order information of the query drug *d* in terms of ∏^*s*^(*d* → *C*_*j*_) (*j* = 1,2,…, 100). 

### 2.5. Jackknife Test

In statistical prediction, Jackknife test [16] is often used to examine a predictor for its effectiveness in practical application. In the jackknife test, all the samples in the dataset will be singled out one-by-one and tested by the predictor trained by the remaining samples. During the process of jackknifing, both the training dataset and testing dataset are actually open, and each sample will be in turn moved between the two. The jackknife test can exclude the “memory” effect, and the arbitrariness problem can also be avoided. Thus, the outcome obtained by the jackknife test is always unique for a given benchmark dataset [26]. Accordingly, the jackknife test has been widely recognized and increasingly adopted to investigate the performance of various predictors [27–36]. Thus, the jackknife test was also adopted here to evaluate the anticipated accuracy of the current predicted methods.

### 2.6. Accuracy Measurement

For a query drug, we may identify a series of side effects with the current prediction method. For the *j*th order prediction, its prediction accuracy AC⁡(*j*) can be calculated by

(15) $$\begin{array}{c} \mathrm{AC}\left(j\right)=\frac{P\left(j\right)}{N}\;j=1,2,\ldots ,100, \end{array}$$

where *P*(*j*) denotes the number of drugs whose *j*th order prediction is one of the true side effects and *N* denotes the total number of structure-different drugs in the dataset. According to the prediction method with 100 orders of prediction results, high AC⁡(*j*) with small *j* and low AC⁡(*j*) with large *j* would indicate a good prediction [13, 16, 20]. Generally speaking, it also implies a good performance by the predictor if its 1st order prediction has a high success rate. 

## 3. Results and Discussion

Of the 835 drugs in the benchmark dataset **S**, 83 samples were randomly selected to compose test dataset **S**_te_, while the rest 752 samples composed the training dataset **S**_tr⁡_. The predicted results of the interaction-based method and similarity-based method on the training and test datasets are as follows.

### 3.1. Performance of the Methods on the Training Dataset

**Figure 2:**
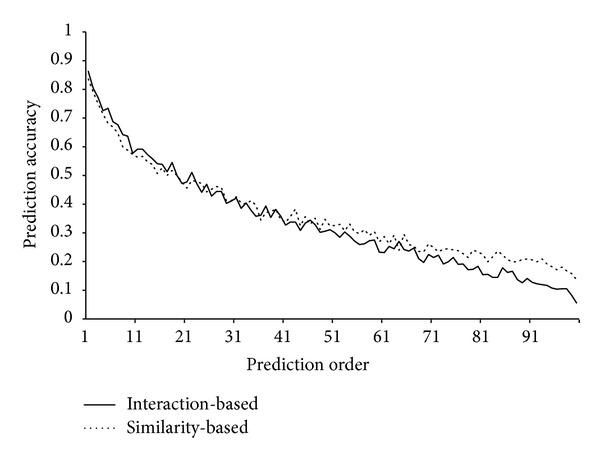
A plot of the prediction accuracy of two methods on the training dataset versus the order of prediction.

For 752 drug compounds in the training dataset **S**_tr⁡_, the interaction-based method and similarity-based method were conducted to make prediction with their performance evaluated by jackknife test. Listed in columns 2 and 4 of Table 1 are the first 20 prediction accuracies obtained by these two methods, from which we can see that the 1st order prediction accuracies of the interaction-based and similarity-based method were 86.30% and 83.64%, respectively, while the 2nd ones were 80.45% and 79.12%, respectively. The total 100 prediction accuracies obtained by these two methods are given in Supplementary Material III, and two curves with prediction accuracies as their *Y*-axis and prediction order as their *X*-axis are shown in Figure 2. It is observed that the prediction accuracies obtained by the interaction-based method descend generally with the increase of the order number, and the same situation also occurred for the prediction accuracies obtained by the similarity-based method. All of these imply that the two methods sorted the side effects of drug compounds in the training dataset quite well, and they are all quite effective in identifying drugs side effects.

### 3.2. Performance of the Methods on the Test Dataset

**Figure 3:**
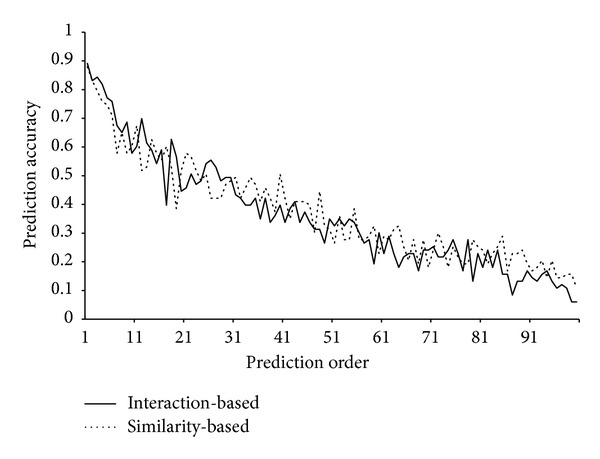
A plot of the prediction accuracy of two methods on the test dataset versus the order of prediction.

For the 83 drug compounds in the test dataset **S**_te_, the side effects of these samples were predicted by the interaction-based and similarity-based method based on the drug compounds in the training dataset **S**_tr⁡_. After processing by (15), 100 prediction accuracies obtained by each method were obtained and were also given in Supplementary Material III. Listed in columns 3 and 5 of Table 1 are the first 20 prediction accuracies obtained by these two methods, from which we can see that the 1st order prediction accuracies obtained by the interaction-based and similarity-based method were 89.16% and 87.95%, respectively. We also plotted two curves with prediction accuracies as their *Y*-axis and prediction order as their *X*-axis, which are shown in Figure 3. It is observed from Figure 3 that the accuracies also exhibit a trend of decrease with the increase of the order number. However, two curves in Figure 3 fluctuate more drastically and frequently than those of Figure 2, which may be caused by the low number of the samples in the test dataset. In any case, the interaction-based and similarity-based methods still sorted the side effects of samples in the test dataset reasonably well, implying again that these two methods are quite effective in identifying drugs side effects.

### 3.3. Comparison of the Interaction-Based and Similarity-Based Method

For 752 samples in the training dataset **S**_tr⁡_ and 83 samples in the test dataset **S**_te_, the interaction-based and similarity-based methods were all used to identify their side effects. Listed in columns 6 and 7 of Table 1 are the differences of the first 20 prediction accuracies obtained by these two methods, from which we can see that the 1st order prediction accuracies obtained by the interaction-based method on the training and test datasets were 2.66% and 1.20% higher than those of similarity-based method. Furthermore, most prediction accuracies in Table 1 obtained by the interaction-based method are higher than the corresponding accuracies obtained by the similarity-based method, indicating that interaction-based method is more effective in identifying drugs side effects. It is also confirmed from Figures 2 and 3 that the curve obtained by the interaction-based method is always above the curve obtained by the similarity-based method when the prediction order is low. However, with the increase of order number, the curve obtained by the similarity-based method keeps up with and exceeds the curve obtained by the interaction-based method, which may be caused by the following two reasons: (I) the high prediction accuracies, obtained by the interaction-based method, with low order number cause the low number of correctly predicted samples with high prediction order; (II) the system of using chemical similarity between two chemicals is more complete than that in STITCH at present, which leads to the fact that the similarity-based method can always identify more side effects than the interaction-based method. It is expected that the interaction-based method can be improved as more and more chemical-chemical and protein-chemical interactions become available in STITCH.

### 3.4. Discussion

It is a multitarget learning problem to predict the side effects of drugs, just like the case in dealing with a protein system with multiple subcellular location sites [37]. For each of the drugs investigated, we need to consider how many different side effects it may have and what are the probabilities these side effects may occur. To deal with this complicated statistical systems like that, we adopted the strategy of the multiple prediction orders, ranging from the most likely side effect prediction order to the least one, that is, giving the information to users, which side effect is most likely, which one is the second likely one, and so forth. Compared to most of the previous studies on the prediction of drugs side effects, our method can provide more information. The multiple prediction orders method can also be utilized to deal with other multi-target learning problems, such as subcellular location prediction [37] and functions of proteins [20].

In addition to the multi-target issue, we also faced the problem of coverage scope. Of the 835 drug compounds in the benchmark dataset, some of them have the information of chemical-chemical interaction, while for the rest such information is missing. To establish a predictor that can be used to predict the side effects of drugs under both the circumstances, the approach of the direct chemical-chemical interaction and the approach of the indirect chemical-chemical interaction were introduced. For the drug compounds belonging to the 1st circumstance, the predictions were conducted based on the direct chemical-chemical interactions (cf. (10)); for the rest drug compounds belonging to the 2nd circumstance, the predictions were conducted based on the hybrid interactions (cf. (13)). Thus, the side effects of all the 835 drugs could be predicted.

Finally, the good performance of the interaction-based method on the training and test datasets suggests that predictions based on the indirect interactions was also quite good, indicating that the entire interaction network—involving all the drug compounds and their direct or indirect interactions, as well as their interactions with human proteins—determines the side effects of drug compounds.

## 4. Conclusions

In this study, we proposed a novel prediction method to identify drugs side effects. For any query drug *d*, its side effects were determined by the following strategy: (1) if there exist interactive compounds of *d* in the training set, only chemical-chemical interactions were used to identify its side effects; (2) otherwise, both chemical-chemical interactions and protein-chemical interactions were employed to make prediction. Good performance of the method on the training and test datasets indicates that our method is quite effective in identifying drugs side effects. We hope that the method would assist in the prediction of drugs side effects during drug development and screening out drug candidates with undesired side effects.

## Supplementary Material

The Supplementary Material contains three files. In details, Supplementary Material I lists the drug compounds and their side effects; Supplementary Material II lists the number of drug compounds with each side effect; Supplementary Material III lists the prediction accuracies obtained by the methods mentioned in this study.

## Figures and Tables

**Table 1:** The first 20 prediction accuracies of the interaction-based and similarity-based methods in identifying the side effects of drugs in the training and test datasets.

Prediction order	Interaction-based		Similarity-based		Difference	
	Training dataset	Test dataset	Training dataset	Test dataset	Training dataset^a^	Test dataset^b^
1	86.30%	89.16%	83.64%	87.95%	2.66%	1.20%
2	80.45%	83.13%	79.12%	83.13%	1.33%	0.00%
3	77.13%	84.34%	75.00%	79.52%	2.13%	4.82%
4	72.61%	81.93%	71.41%	75.90%	1.20%	6.02%
5	73.40%	77.11%	68.22%	74.70%	5.19%	2.41%
6	68.75%	75.90%	66.89%	71.08%	1.86%	4.82%
7	67.69%	67.47%	64.76%	57.83%	2.93%	9.64%
8	64.23%	65.06%	59.97%	65.06%	4.26%	0.00%
9	63.70%	68.67%	58.78%	57.83%	4.92%	10.84%
10	57.71%	57.83%	57.31%	60.24%	0.40%	−2.41%
11	59.18%	60.24%	56.38%	67.47%	2.79%	−7.23%
12	59.18%	69.88%	56.65%	51.81%	2.53%	18.07%
13	57.31%	61.45%	54.79%	53.01%	2.53%	8.43%
14	55.85%	59.04%	53.86%	62.65%	1.99%	−3.61%
15	54.12%	54.22%	50.66%	57.83%	3.46%	−3.61%
16	53.86%	59.04%	52.66%	55.42%	1.20%	3.61%
17	51.33%	39.76%	50.00%	60.24%	1.33%	−20.48%
18	54.52%	62.65%	51.73%	53.01%	2.79%	9.64%
19	50.00%	56.63%	50.00%	38.55%	0.00%	18.07%
20	47.21%	44.58%	47.74%	51.81%	−0.53%	−7.23%

^a^Percentages in this column were calculated by percentages in column 2 minus percentages in column 4.

^b^Percentages in this column were calculated by percentages in column 3 minus percentages in column 5.
