# Supplementary material for: Predicting Drugs Side Effects Based on Chemical-Chemical Interactions and Protein-Chemical Interactions
Source: Biomed Res Int. 2013 Sep 4;2013:485034. doi: 10.1155/2013/485034 (PMC3776367; doi:10.1155/2013/485034)
Supplement: Supplementary file 1 — The Supplementary Material contains three files. In details, Supplementary Material I lists the drug compounds and their side effects; Supplementary Material II lists the number of drug compounds with each side effect; Supplementary Material III lists the prediction accuracies obtained by the methods mentioned in this study. [file 485034.f1.pdf]

Supplementary Material I. The benchmark dataset consists of structure-different 835 drug compounds and 30,114 side-effect-different virtual drugs (see Eq.2 of the paper) classified into 100 categories according to their side effects (see Eq.1 of the paper).

(1) $\mathbb{S}_1$: 703 drug compounds having side effect “Nausea”

CID000000085	CID000000143	CID000000158	CID000000159	CID000000191
CID000000206	CID000000214	CID000000232	CID000000247	CID000000271
CID000000298	CID000000444	CID000000450	CID000000453	CID000000564
CID000000581	CID000000596	CID000000598	CID000000681	CID000000738
CID000000750	CID000000767	CID000000772	CID000000807	CID000000815
CID000000838	CID000000853	CID000000937	CID000000942	CID000001003
CID000001046	CID000001065	CID000001125	CID000001134	CID000001148
CID000001546	CID000001690	CID000001775	CID000001935	CID000001971
CID000001972	CID000001978	CID000001986	CID000002021	CID000002022
CID000002082	CID000002083	CID000002092	CID000002099	CID000002123
CID000002130	CID000002131	CID000002140	CID000002141	CID000002142
CID000002145	CID000002148	CID000002153	CID000002156	CID000002160
CID000002162	CID000002170	CID000002171	CID000002173	CID000002177
CID000002179	CID000002182	CID000002187	CID000002215	CID000002216
CID000002232	CID000002244	CID000002249	CID000002250	CID000002265
CID000002267	CID000002269	CID000002274	CID000002284	CID000002308
CID000002311	CID000002315	CID000002344	CID000002349	CID000002366
CID000002369	CID000002370	CID000002375	CID000002405	CID000002431
CID000002435	CID000002441	CID000002462	CID000002471	CID000002474
CID000002476	CID000002477	CID000002478	CID000002487	CID000002512
CID000002520	CID000002524	CID000002541	CID000002550	CID000002554
CID000002559	CID000002564	CID000002575	CID000002576	CID000002578
CID000002583	CID000002585	CID000002609	CID000002610	CID000002617
CID000002622	CID000002629	CID000002631	CID000002637	CID000002646
CID000002650	CID000002654	CID000002655	CID000002656	CID000002658
CID000002662	CID000002666	CID000002673	CID000002675	CID000002676
CID000002678	CID000002708	CID000002712	CID000002719	CID000002720
CID000002725	CID000002727	CID000002732	CID000002751	CID000002762
CID000002764	CID000002769	CID000002771	CID000002781	CID000002786
CID000002794	CID000002800	CID000002801	CID000002803	CID000002806
CID000002812	CID000002818	CID000002891	CID000002895	CID000002907
CID000002909	CID000002913	CID000002949	CID000002951	CID000002958
CID000002973	CID000002983	CID000002995	CID000003007	CID000003009
CID000003015	CID000003016	CID000003019	CID000003032	CID000003040
CID000003042	CID000003043	CID000003059	CID000003062	CID000003066
CID000003075	CID000003080	CID000003100	CID000003108	CID000003114
CID000003121	CID000003125	CID000003143	CID000003152	CID000003154
CID000003156	CID000003157	CID000003158	CID000003203	CID000003222

CID000003226	CID000003249	CID000003251	CID000003255	CID000003261
CID000003278	CID000003279	CID000003285	CID000003291	CID000003292
CID000003305	CID000003308	CID000003310	CID000003324	CID000003325
CID000003333	CID000003339	CID000003340	CID000003342	CID000003345
CID000003348	CID000003354	CID000003355	CID000003366	CID000003367
CID000003372	CID000003373	CID000003379	CID000003381	CID000003385
CID000003387	CID000003393	CID000003394	CID000003397	CID000003403
CID000003404	CID000003405	CID000003406	CID000003410	CID000003414
CID000003417	CID000003419	CID000003440	CID000003446	CID000003449
CID000003454	CID000003461	CID000003463	CID000003467	CID000003475
CID000003476	CID000003478	CID000003488	CID000003494	CID000003512
CID000003518	CID000003519	CID000003559	CID000003562	CID000003636
CID000003637	CID000003639	CID000003640	CID000003647	CID000003648
CID000003652	CID000003657	CID000003661	CID000003675	CID000003676
CID000003685	CID000003690	CID000003696	CID000003698	CID000003702
CID000003706	CID000003715	CID000003724	CID000003730	CID000003734
CID000003736	CID000003737	CID000003739	CID000003741	CID000003742
CID000003746	CID000003749	CID000003750	CID000003759	CID000003763
CID000003767	CID000003779	CID000003783	CID000003784	CID000003793
CID000003821	CID000003823	CID000003825	CID000003826	CID000003827
CID000003869	CID000003877	CID000003878	CID000003883	CID000003899
CID000003902	CID000003911	CID000003914	CID000003915	CID000003928
CID000003929	CID000003937	CID000003948	CID000003950	CID000003954
CID000003956	CID000003957	CID000003958	CID000003961	CID000003962
CID000003964	CID000004011	CID000004032	CID000004033	CID000004036
CID000004044	CID000004046	CID000004053	CID000004054	CID000004057
CID000004058	CID000004060	CID000004062	CID000004064	CID000004078
CID000004086	CID000004091	CID000004095	CID000004100	CID000004101
CID000004107	CID000004112	CID000004114	CID000004121	CID000004138
CID000004140	CID000004158	CID000004159	CID000004160	CID000004163
CID000004170	CID000004171	CID000004173	CID000004174	CID000004178
CID000004189	CID000004192	CID000004195	CID000004196	CID000004200
CID000004201	CID000004205	CID000004211	CID000004212	CID000004236
CID000004253	CID000004259	CID000004264	CID000004409	CID000004411
CID000004419	CID000004421	CID000004425	CID000004428	CID000004436
CID000004440	CID000004449	CID000004451	CID000004463	CID000004473
CID000004485	CID000004493	CID000004497	CID000004506	CID000004509
CID000004510	CID000004513	CID000004536	CID000004539	CID000004542
CID000004543	CID000004547	CID000004583	CID000004585	CID000004599
CID000004601	CID000004603	CID000004607	CID000004609	CID000004614
CID000004616	CID000004635	CID000004638	CID000004645	CID000004666
CID000004679	CID000004689	CID000004723	CID000004724	CID000004727
CID000004730	CID000004736	CID000004737	CID000004739	CID000004740
CID000004745	CID000004775	CID000004819	CID000004828	CID000004834

CID000004845	CID000004856	CID000004870	CID000004873	CID000004885
CID000004889	CID000004891	CID000004893	CID000004894	CID000004908
CID000004909	CID000004911	CID000004913	CID000004914	CID000004915
CID000004919	CID000004920	CID000004932	CID000004934	CID000004943
CID000004946	CID000004976	CID000004991	CID000005002	CID000005005
CID000005029	CID000005035	CID000005038	CID000005039	CID000005040
CID000005052	CID000005064	CID000005070	CID000005071	CID000005073
CID000005076	CID000005077	CID000005078	CID000005090	CID000005095
CID000005152	CID000005155	CID000005193	CID000005195	CID000005206
CID000005210	CID000005212	CID000005215	CID000005245	CID000005253
CID000005267	CID000005291	CID000005297	CID000005300	CID000005344
CID000005352	CID000005358	CID000005372	CID000005376	CID000005379
CID000005391	CID000005394	CID000005396	CID000005401	CID000005402
CID000005403	CID000005408	CID000005412	CID000005426	CID000005430
CID000005452	CID000005453	CID000005454	CID000005466	CID000005472
CID000005478	CID000005479	CID000005486	CID000005487	CID000005496
CID000005503	CID000005504	CID000005505	CID000005508	CID000005514
CID000005515	CID000005516	CID000005523	CID000005525	CID000005526
CID000005530	CID000005533	CID000005538	CID000005544	CID000005546
CID000005556	CID000005566	CID000005572	CID000005578	CID000005582
CID000005584	CID000005591	CID000005593	CID000005625	CID000005645
CID000005647	CID000005650	CID000005651	CID000005665	CID000005672
CID000005717	CID000005718	CID000005719	CID000005721	CID000005726
CID000005731	CID000005732	CID000005734	CID000005735	CID000005746
CID000005771	CID000005775	CID000005978	CID000006049	CID000006058
CID000006476	CID000006691	CID000007029	CID000008612	CID000009034
CID000009433	CID000009904	CID000010100	CID000010631	CID000012555
CID000013342	CID000014888	CID000016850	CID000018140	CID000019090
CID000020585	CID000023897	CID000025419	CID000027400	CID000027661
CID000027991	CID000028112	CID000030623	CID000031477	CID000032800
CID000034312	CID000036339	CID000036811	CID000038904	CID000039042
CID000040159	CID000040976	CID000041317	CID000041693	CID000041744
CID000041774	CID000041781	CID000042113	CID000042615	CID000044564
CID000047725	CID000050294	CID000050614	CID000051263	CID000051634
CID000054454	CID000054547	CID000054688	CID000054786	CID000056959
CID000057469	CID000057537	CID000059708	CID000059768	CID000060184
CID000060198	CID000060612	CID000060613	CID000060714	CID000060753
CID000060754	CID000060787	CID000060795	CID000060843	CID000060852
CID000060865	CID000060871	CID000060877	CID000060953	CID000062816
CID000062819	CID000062867	CID000062924	CID000062959	CID000064147
CID000065027	CID000065999	CID000068740	CID000068844	CID000071158
CID000071273	CID000071301	CID000071329	CID000071616	CID000072054
CID000072938	CID000074989	CID000077992	CID000077993	CID000077999
CID000082146	CID000083786	CID000093860	CID000096312	CID000104741

CID000104758	CID000104865	CID000110634	CID000110635	CID000115237
CID000119182	CID000119607	CID000122316	CID000123606	CID000123620
CID000123631	CID000124087	CID000125017	CID000125889	CID000130881
CID000147912	CID000148192	CID000150311	CID000150610	CID000153941
CID000158440	CID000160051	CID000163742	CID000166548	CID000170361
CID000176168	CID000176870	CID000197712	CID000213039	CID000216239
CID000216326	CID000443871	CID000444013	CID000444033	CID000450096
CID000477468	CID000657298	CID000667490	CID001349907	CID002761171
CID003002190	CID003062316	CID003081884	CID003086672	CID004183806
CID004659568	CID004659569	CID005229711	CID005281007	CID005281104
CID005282044	CID005311181	CID005311297	CID005329102	CID005353894
CID005361912	CID005362070	CID005362420	CID005381226	CID005473385
CID005481350	CID005487301	CID005493381	CID005493444	CID006323497
CID006398525	CID006398970	CID006435110	CID006436173	CID006447131
CID006918453	CID009571074	CID011947681		

(2) $\mathbb{S}_2$: 663 drug compounds having side effect “Headache”

CID000000085	CID000000158	CID000000159	CID000000191	CID000000206
CID000000214	CID000000232	CID000000298	CID000000401	CID000000444
CID000000450	CID000000453	CID000000564	CID000000596	CID000000598
CID000000681	CID000000727	CID000000738	CID000000767	CID000000772
CID000000807	CID000000815	CID000000838	CID000000853	CID000000937
CID000000942	CID000000951	CID000001003	CID000001065	CID000001125
CID000001134	CID000001148	CID000001206	CID000001546	CID000001775
CID000001935	CID000001971	CID000001972	CID000001978	CID000001986
CID000002022	CID000002082	CID000002083	CID000002092	CID000002099
CID000002118	CID000002130	CID000002142	CID000002145	CID000002153
CID000002156	CID000002160	CID000002162	CID000002170	CID000002171
CID000002177	CID000002179	CID000002182	CID000002187	CID000002215
CID000002216	CID000002232	CID000002249	CID000002250	CID000002267
CID000002269	CID000002274	CID000002284	CID000002308	CID000002311
CID000002315	CID000002349	CID000002366	CID000002369	CID000002370
CID000002375	CID000002405	CID000002435	CID000002441	CID000002443
CID000002462	CID000002471	CID000002474	CID000002476	CID000002477
CID000002478	CID000002487	CID000002512	CID000002520	CID000002524
CID000002541	CID000002550	CID000002551	CID000002559	CID000002564
CID000002575	CID000002576	CID000002578	CID000002583	CID000002585
CID000002609	CID000002610	CID000002622	CID000002631	CID000002646
CID000002650	CID000002654	CID000002656	CID000002658	CID000002662
CID000002666	CID000002675	CID000002676	CID000002678	CID000002713
CID000002719	CID000002720	CID000002727	CID000002732	CID000002749
CID000002751	CID000002756	CID000002762	CID000002764	CID000002769
CID000002771	CID000002781	CID000002786	CID000002794	CID000002800
CID000002801	CID000002802	CID000002803	CID000002806	CID000002818

CID000002891	CID000002895	CID000002907	CID000002909	CID000002913
CID000002949	CID000002951	CID000002955	CID000002958	CID000002973
CID000002978	CID000002983	CID000002995	CID000003003	CID000003007
CID000003009	CID000003015	CID000003016	CID000003019	CID000003032
CID000003042	CID000003043	CID000003059	CID000003062	CID000003066
CID000003075	CID000003080	CID000003100	CID000003105	CID000003108
CID000003114	CID000003117	CID000003125	CID000003143	CID000003148
CID000003151	CID000003152	CID000003154	CID000003156	CID000003157
CID000003158	CID000003161	CID000003203	CID000003219	CID000003222
CID000003241	CID000003251	CID000003261	CID000003278	CID000003279
CID000003285	CID000003291	CID000003292	CID000003305	CID000003308
CID000003310	CID000003324	CID000003325	CID000003333	CID000003339
CID000003340	CID000003342	CID000003345	CID000003348	CID000003350
CID000003354	CID000003355	CID000003366	CID000003367	CID000003372
CID000003373	CID000003379	CID000003381	CID000003382	CID000003385
CID000003387	CID000003393	CID000003394	CID000003403	CID000003404
CID000003406	CID000003410	CID000003414	CID000003417	CID000003419
CID000003440	CID000003446	CID000003449	CID000003454	CID000003461
CID000003463	CID000003467	CID000003475	CID000003476	CID000003478
CID000003494	CID000003510	CID000003512	CID000003519	CID000003559
CID000003637	CID000003639	CID000003640	CID000003647	CID000003648
CID000003652	CID000003657	CID000003658	CID000003661	CID000003675
CID000003676	CID000003685	CID000003696	CID000003702	CID000003706
CID000003715	CID000003724	CID000003730	CID000003734	CID000003736
CID000003737	CID000003739	CID000003741	CID000003742	CID000003746
CID000003749	CID000003750	CID000003759	CID000003779	CID000003780
CID000003784	CID000003793	CID000003823	CID000003825	CID000003826
CID000003827	CID000003869	CID000003877	CID000003878	CID000003883
CID000003899	CID000003902	CID000003911	CID000003914	CID000003915
CID000003929	CID000003937	CID000003948	CID000003954	CID000003956
CID000003957	CID000003958	CID000003961	CID000003962	CID000003964
CID000004011	CID000004036	CID000004044	CID000004046	CID000004054
CID000004057	CID000004058	CID000004062	CID000004064	CID000004075
CID000004086	CID000004091	CID000004095	CID000004107	CID000004112
CID000004114	CID000004121	CID000004138	CID000004140	CID000004158
CID000004159	CID000004160	CID000004168	CID000004170	CID000004171
CID000004173	CID000004174	CID000004178	CID000004189	CID000004192
CID000004195	CID000004196	CID000004197	CID000004200	CID000004201
CID000004205	CID000004212	CID000004236	CID000004253	CID000004259
CID000004264	CID000004409	CID000004411	CID000004419	CID000004421
CID000004428	CID000004436	CID000004440	CID000004449	CID000004451
CID000004463	CID000004473	CID000004485	CID000004493	CID000004497
CID000004506	CID000004509	CID000004510	CID000004513	CID000004536
CID000004539	CID000004542	CID000004543	CID000004547	CID000004583

CID000004585	CID000004594	CID000004595	CID000004599	CID000004601
CID000004609	CID000004614	CID000004616	CID000004634	CID000004635
CID000004666	CID000004679	CID000004723	CID000004724	CID000004725
CID000004736	CID000004737	CID000004739	CID000004740	CID000004745
CID000004748	CID000004771	CID000004775	CID000004819	CID000004828
CID000004829	CID000004834	CID000004845	CID000004856	CID000004865
CID000004870	CID000004885	CID000004889	CID000004891	CID000004893
CID000004894	CID000004900	CID000004911	CID000004914	CID000004915
CID000004917	CID000004920	CID000004932	CID000004934	CID000004943
CID000004946	CID000004976	CID000004991	CID000005002	CID000005005
CID000005029	CID000005035	CID000005038	CID000005039	CID000005040
CID000005052	CID000005064	CID000005070	CID000005071	CID000005073
CID000005076	CID000005077	CID000005078	CID000005090	CID000005095
CID000005152	CID000005155	CID000005193	CID000005195	CID000005206
CID000005210	CID000005212	CID000005215	CID000005245	CID000005253
CID000005267	CID000005291	CID000005344	CID000005352	CID000005358
CID000005372	CID000005376	CID000005379	CID000005391	CID000005394
CID000005396	CID000005401	CID000005402	CID000005403	CID000005404
CID000005408	CID000005412	CID000005419	CID000005426	CID000005430
CID000005452	CID000005453	CID000005466	CID000005472	CID000005478
CID000005479	CID000005486	CID000005496	CID000005503	CID000005505
CID000005508	CID000005512	CID000005515	CID000005523	CID000005525
CID000005530	CID000005533	CID000005538	CID000005544	CID000005546
CID000005556	CID000005566	CID000005572	CID000005584	CID000005593
CID000005596	CID000005625	CID000005636	CID000005645	CID000005647
CID000005650	CID000005656	CID000005665	CID000005672	CID000005717
CID000005718	CID000005719	CID000005726	CID000005731	CID000005732
CID000005734	CID000005735	CID000005746	CID000005775	CID000005978
CID000006049	CID000006058	CID000006476	CID000006691	CID000007029
CID000008612	CID000009034	CID000009433	CID000010100	CID000010631
CID000012536	CID000012555	CID000012620	CID000013342	CID000014888
CID000016362	CID000016850	CID000018140	CID000019090	CID000020585
CID000027400	CID000027661	CID000027991	CID000028112	CID000030623
CID000031378	CID000031477	CID000032797	CID000032800	CID000034312
CID000036811	CID000039042	CID000039507	CID000039860	CID000040159
CID000040976	CID000041317	CID000041693	CID000041744	CID000041781
CID000042113	CID000042615	CID000044564	CID000047725	CID000050294
CID000051263	CID000051634	CID000054454	CID000054547	CID000054688
CID000054786	CID000056959	CID000057469	CID000057537	CID000059708
CID000059768	CID000060184	CID000060198	CID000060612	CID000060613
CID000060714	CID000060753	CID000060754	CID000060787	CID000060795
CID000060852	CID000060865	CID000060871	CID000060877	CID000060953
CID000062816	CID000062819	CID000062867	CID000062924	CID000062959
CID000064147	CID000065027	CID000065999	CID000068740	CID000068844

CID000071158	CID000071273	CID000071301	CID000071329	CID000071616
CID000072054	CID000072938	CID000074989	CID000077992	CID000077993
CID000077999	CID000082146	CID000083786	CID000093860	CID000096312
CID000104741	CID000104758	CID000104865	CID000110634	CID000110635
CID000115237	CID000119182	CID000119607	CID000122316	CID000123606
CID000123620	CID000124087	CID000125017	CID000125889	CID000130881
CID000147912	CID000148192	CID000148211	CID000150310	CID000150311
CID000150610	CID000151165	CID000153941	CID000158440	CID000160051
CID000163742	CID000166548	CID000170361	CID000176168	CID000176870
CID000197712	CID000213039	CID000216239	CID000216326	CID000222786
CID000444013	CID000444033	CID000450096	CID000477468	CID000657298
CID001349907	CID002761171	CID003002190	CID003062316	CID003081884
CID003086672	CID004183806	CID004659569	CID005229711	CID005281007
CID005281104	CID005282044	CID005282226	CID005311027	CID005311181
CID005329102	CID005353894	CID005353980	CID005361912	CID005362070
CID005362420	CID005381226	CID005481350	CID005487301	CID005493381
CID005493444	CID006323497	CID006398525	CID006398970	CID006436173
CID006447131	CID009571074	CID011947681		

(3) $\mathbb{S}_3$: 652 drug compounds having side effect “Vomiting”

CID000000085	CID000000143	CID000000158	CID000000159	CID000000191
CID000000206	CID000000232	CID000000271	CID000000298	CID000000444
CID000000450	CID000000453	CID000000564	CID000000581	CID000000596
CID000000598	CID000000681	CID000000738	CID000000750	CID000000767
CID000000772	CID000000807	CID000000815	CID000000853	CID000000937
CID000000951	CID000001003	CID000001046	CID000001065	CID000001125
CID000001134	CID000001148	CID000001546	CID000001690	CID000001775
CID000001935	CID000001971	CID000001972	CID000001978	CID000001986
CID000002022	CID000002082	CID000002083	CID000002118	CID000002123
CID000002130	CID000002131	CID000002140	CID000002141	CID000002142
CID000002145	CID000002148	CID000002153	CID000002156	CID000002160
CID000002162	CID000002170	CID000002171	CID000002173	CID000002177
CID000002179	CID000002182	CID000002187	CID000002215	CID000002216
CID000002232	CID000002244	CID000002250	CID000002265	CID000002267
CID000002269	CID000002274	CID000002284	CID000002311	CID000002315
CID000002344	CID000002349	CID000002369	CID000002375	CID000002405
CID000002431	CID000002441	CID000002443	CID000002462	CID000002471
CID000002474	CID000002476	CID000002477	CID000002478	CID000002487
CID000002512	CID000002520	CID000002524	CID000002541	CID000002550
CID000002554	CID000002559	CID000002564	CID000002575	CID000002576
CID000002578	CID000002585	CID000002609	CID000002610	CID000002617
CID000002622	CID000002629	CID000002631	CID000002637	CID000002646
CID000002650	CID000002654	CID000002655	CID000002656	CID000002658
CID000002662	CID000002666	CID000002673	CID000002675	CID000002676

CID000002678	CID000002708	CID000002719	CID000002720	CID000002725
CID000002727	CID000002732	CID000002751	CID000002762	CID000002764
CID000002769	CID000002771	CID000002781	CID000002786	CID000002794
CID000002800	CID000002801	CID000002802	CID000002803	CID000002806
CID000002812	CID000002818	CID000002891	CID000002895	CID000002907
CID000002909	CID000002913	CID000002949	CID000002951	CID000002955
CID000002958	CID000002983	CID000002995	CID000003007	CID000003015
CID000003016	CID000003019	CID000003032	CID000003040	CID000003042
CID000003043	CID000003059	CID000003062	CID000003066	CID000003075
CID000003080	CID000003100	CID000003108	CID000003114	CID000003121
CID000003125	CID000003143	CID000003152	CID000003154	CID000003157
CID000003158	CID000003203	CID000003222	CID000003226	CID000003251
CID000003255	CID000003261	CID000003278	CID000003279	CID000003285
CID000003291	CID000003292	CID000003305	CID000003308	CID000003310
CID000003324	CID000003325	CID000003333	CID000003339	CID000003340
CID000003342	CID000003345	CID000003348	CID000003354	CID000003355
CID000003365	CID000003366	CID000003367	CID000003372	CID000003373
CID000003379	CID000003381	CID000003385	CID000003393	CID000003394
CID000003397	CID000003403	CID000003404	CID000003406	CID000003410
CID000003414	CID000003417	CID000003419	CID000003440	CID000003446
CID000003449	CID000003454	CID000003461	CID000003463	CID000003467
CID000003475	CID000003476	CID000003478	CID000003488	CID000003494
CID000003512	CID000003518	CID000003559	CID000003562	CID000003637
CID000003639	CID000003647	CID000003648	CID000003652	CID000003657
CID000003661	CID000003672	CID000003675	CID000003676	CID000003685
CID000003690	CID000003696	CID000003698	CID000003702	CID000003706
CID000003715	CID000003724	CID000003730	CID000003734	CID000003736
CID000003737	CID000003739	CID000003741	CID000003742	CID000003746
CID000003749	CID000003750	CID000003763	CID000003767	CID000003783
CID000003784	CID000003793	CID000003821	CID000003823	CID000003825
CID000003826	CID000003827	CID000003869	CID000003877	CID000003878
CID000003883	CID000003899	CID000003902	CID000003911	CID000003928
CID000003929	CID000003937	CID000003948	CID000003950	CID000003954
CID000003956	CID000003957	CID000003958	CID000003961	CID000003962
CID000003964	CID000004011	CID000004032	CID000004033	CID000004036
CID000004044	CID000004046	CID000004053	CID000004054	CID000004057
CID000004058	CID000004060	CID000004062	CID000004064	CID000004075
CID000004078	CID000004086	CID000004091	CID000004095	CID000004100
CID000004101	CID000004107	CID000004112	CID000004121	CID000004138
CID000004140	CID000004158	CID000004159	CID000004163	CID000004170
CID000004171	CID000004173	CID000004174	CID000004178	CID000004192
CID000004196	CID000004200	CID000004201	CID000004205	CID000004211
CID000004212	CID000004236	CID000004253	CID000004259	CID000004409
CID000004411	CID000004419	CID000004421	CID000004425	CID000004428

CID000004440	CID000004449	CID000004451	CID000004463	CID000004473
CID000004485	CID000004493	CID000004497	CID000004506	CID000004509
CID000004510	CID000004513	CID000004539	CID000004542	CID000004543
CID000004547	CID000004583	CID000004585	CID000004599	CID000004601
CID000004603	CID000004607	CID000004609	CID000004614	CID000004635
CID000004638	CID000004645	CID000004666	CID000004679	CID000004691
CID000004727	CID000004730	CID000004736	CID000004737	CID000004739
CID000004740	CID000004745	CID000004775	CID000004819	CID000004828
CID000004834	CID000004845	CID000004856	CID000004865	CID000004870
CID000004873	CID000004885	CID000004889	CID000004891	CID000004893
CID000004908	CID000004909	CID000004911	CID000004913	CID000004914
CID000004915	CID000004919	CID000004920	CID000004932	CID000004934
CID000004943	CID000004946	CID000004976	CID000004991	CID000004993
CID000005002	CID000005005	CID000005029	CID000005035	CID000005038
CID000005039	CID000005040	CID000005052	CID000005064	CID000005070
CID000005071	CID000005073	CID000005076	CID000005077	CID000005078
CID000005090	CID000005095	CID000005152	CID000005155	CID000005193
CID000005195	CID000005203	CID000005206	CID000005210	CID000005212
CID000005215	CID000005245	CID000005253	CID000005267	CID000005291
CID000005297	CID000005300	CID000005344	CID000005352	CID000005358
CID000005372	CID000005376	CID000005379	CID000005391	CID000005394
CID000005396	CID000005401	CID000005402	CID000005403	CID000005408
CID000005412	CID000005426	CID000005430	CID000005452	CID000005453
CID000005454	CID000005466	CID000005472	CID000005478	CID000005479
CID000005487	CID000005496	CID000005504	CID000005508	CID000005514
CID000005515	CID000005516	CID000005523	CID000005525	CID000005526
CID000005533	CID000005538	CID000005544	CID000005546	CID000005556
CID000005566	CID000005572	CID000005578	CID000005582	CID000005584
CID000005591	CID000005593	CID000005596	CID000005625	CID000005645
CID000005647	CID000005650	CID000005656	CID000005665	CID000005672
CID000005717	CID000005718	CID000005721	CID000005726	CID000005731
CID000005732	CID000005734	CID000005735	CID000005746	CID000005771
CID000005775	CID000005978	CID000006049	CID000006058	CID000006476
CID000006691	CID000007029	CID000008612	CID000009034	CID000009433
CID000009904	CID000010100	CID000010631	CID000012555	CID000013342
CID000014888	CID000016850	CID000018140	CID000019090	CID000020585
CID000025419	CID000027661	CID000027991	CID000028112	CID000030623
CID000032800	CID000034312	CID000036339	CID000038904	CID000040159
CID000040976	CID000041693	CID000041744	CID000041774	CID000041781
CID000042113	CID000042615	CID000047725	CID000050294	CID000050614
CID000051263	CID000051634	CID000054454	CID000054547	CID000054688
CID000054786	CID000056959	CID000057469	CID000057537	CID000059708
CID000059768	CID000060184	CID000060198	CID000060612	CID000060613
CID000060714	CID000060754	CID000060787	CID000060795	CID000060843

CID000060852	CID000060871	CID000060877	CID000060953	CID000062816
CID000062819	CID000062867	CID000062924	CID000062959	CID000064147
CID000065027	CID000065999	CID000068740	CID000071158	CID000071273
CID000071301	CID000071616	CID000072054	CID000072938	CID000074989
CID000077992	CID000077993	CID000077999	CID000082146	CID000083786
CID000093860	CID000096312	CID000104741	CID000104758	CID000104865
CID000110634	CID000110635	CID000115237	CID000119182	CID000119607
CID000122316	CID000123606	CID000123620	CID000123631	CID000124087
CID000125017	CID000125889	CID000130881	CID000147912	CID000148192
CID000150610	CID000153941	CID000158440	CID000163742	CID000166548
CID000170361	CID000176168	CID000176870	CID000213039	CID000216239
CID000216326	CID000444013	CID000450096	CID000477468	CID000657298
CID000667490	CID001349907	CID002761171	CID003002190	CID003062316
CID003081884	CID003086672	CID004183806	CID004659568	CID004659569
CID005229711	CID005281007	CID005281104	CID005282044	CID005311181
CID005311297	CID005329102	CID005361912	CID005362070	CID005381226
CID005473385	CID005481350	CID005487301	CID005493381	CID006323497
CID006398525	CID006398970	CID006435110	CID006436173	CID006447131
CID006918453	CID009571074			

(4) $\mathbb{S}_4$: 604 drug compounds having side effect “Rash”

CID000000085	CID000000137	CID000000143	CID000000158	CID000000159
CID000000214	CID000000232	CID000000401	CID000000444	CID000000450
CID000000564	CID000000581	CID000000596	CID000000598	CID000000612
CID000000738	CID000000767	CID000000772	CID000000807	CID000000815
CID000000853	CID000000937	CID000000942	CID000001003	CID000001065
CID000001125	CID000001134	CID000001546	CID000001775	CID000001935
CID000001971	CID000001972	CID000001978	CID000002022	CID000002082
CID000002083	CID000002092	CID000002099	CID000002118	CID000002123
CID000002130	CID000002140	CID000002142	CID000002145	CID000002156
CID000002160	CID000002162	CID000002170	CID000002177	CID000002179
CID000002182	CID000002187	CID000002216	CID000002232	CID000002249
CID000002250	CID000002266	CID000002267	CID000002269	CID000002274
CID000002284	CID000002308	CID000002311	CID000002315	CID000002344
CID000002349	CID000002369	CID000002375	CID000002405	CID000002431
CID000002435	CID000002441	CID000002443	CID000002462	CID000002471
CID000002476	CID000002477	CID000002478	CID000002487	CID000002512
CID000002519	CID000002520	CID000002522	CID000002524	CID000002541
CID000002550	CID000002554	CID000002559	CID000002575	CID000002576
CID000002578	CID000002583	CID000002585	CID000002609	CID000002610
CID000002617	CID000002622	CID000002631	CID000002637	CID000002646
CID000002650	CID000002654	CID000002655	CID000002656	CID000002658
CID000002662	CID000002666	CID000002673	CID000002676	CID000002678
CID000002708	CID000002720	CID000002732	CID000002749	CID000002751

CID000002756	CID000002762	CID000002764	CID000002769	CID000002771
CID000002786	CID000002794	CID000002800	CID000002801	CID000002802
CID000002803	CID000002806	CID000002812	CID000002818	CID000002891
CID000002895	CID000002905	CID000002907	CID000002909	CID000002913
CID000002951	CID000002958	CID000002973	CID000002978	CID000002995
CID000003003	CID000003007	CID000003009	CID000003015	CID000003016
CID000003019	CID000003032	CID000003040	CID000003042	CID000003043
CID000003059	CID000003062	CID000003066	CID000003075	CID000003108
CID000003114	CID000003121	CID000003143	CID000003148	CID000003151
CID000003152	CID000003154	CID000003157	CID000003158	CID000003161
CID000003198	CID000003203	CID000003222	CID000003261	CID000003278
CID000003279	CID000003285	CID000003292	CID000003305	CID000003308
CID000003310	CID000003325	CID000003333	CID000003339	CID000003342
CID000003345	CID000003348	CID000003350	CID000003355	CID000003365
CID000003366	CID000003367	CID000003373	CID000003379	CID000003381
CID000003385	CID000003386	CID000003393	CID000003394	CID000003397
CID000003403	CID000003405	CID000003406	CID000003410	CID000003414
CID000003417	CID000003419	CID000003440	CID000003446	CID000003449
CID000003454	CID000003461	CID000003463	CID000003467	CID000003475
CID000003478	CID000003510	CID000003519	CID000003636	CID000003637
CID000003639	CID000003640	CID000003647	CID000003648	CID000003657
CID000003658	CID000003661	CID000003672	CID000003675	CID000003685
CID000003696	CID000003702	CID000003706	CID000003715	CID000003724
CID000003734	CID000003736	CID000003737	CID000003741	CID000003742
CID000003746	CID000003749	CID000003750	CID000003783	CID000003784
CID000003793	CID000003821	CID000003823	CID000003825	CID000003826
CID000003827	CID000003869	CID000003877	CID000003883	CID000003890
CID000003899	CID000003902	CID000003911	CID000003914	CID000003915
CID000003929	CID000003937	CID000003948	CID000003954	CID000003956
CID000003957	CID000003958	CID000003961	CID000003962	CID000003964
CID000003998	CID000004011	CID000004030	CID000004033	CID000004036
CID000004046	CID000004054	CID000004064	CID000004075	CID000004078
CID000004091	CID000004101	CID000004107	CID000004112	CID000004114
CID000004121	CID000004138	CID000004140	CID000004158	CID000004168
CID000004170	CID000004171	CID000004173	CID000004174	CID000004178
CID000004189	CID000004192	CID000004195	CID000004196	CID000004197
CID000004200	CID000004201	CID000004205	CID000004212	CID000004236
CID000004253	CID000004259	CID000004264	CID000004409	CID000004411
CID000004419	CID000004421	CID000004428	CID000004449	CID000004451
CID000004473	CID000004485	CID000004493	CID000004497	CID000004510
CID000004513	CID000004536	CID000004539	CID000004542	CID000004543
CID000004583	CID000004585	CID000004594	CID000004595	CID000004599
CID000004603	CID000004607	CID000004609	CID000004614	CID000004623
CID000004635	CID000004666	CID000004675	CID000004679	CID000004691

CID000004723	CID000004724	CID000004725	CID000004739	CID000004740
CID000004745	CID000004775	CID000004819	CID000004828	CID000004834
CID000004845	CID000004856	CID000004865	CID000004870	CID000004873
CID000004885	CID000004889	CID000004893	CID000004894	CID000004913
CID000004915	CID000004919	CID000004920	CID000004927	CID000004932
CID000004943	CID000004946	CID000004976	CID000004991	CID000005002
CID000005005	CID000005029	CID000005035	CID000005038	CID000005039
CID000005040	CID000005052	CID000005064	CID000005070	CID000005071
CID000005073	CID000005076	CID000005077	CID000005078	CID000005090
CID000005095	CID000005152	CID000005155	CID000005195	CID000005203
CID000005206	CID000005210	CID000005212	CID000005245	CID000005253
CID000005291	CID000005297	CID000005314	CID000005342	CID000005344
CID000005352	CID000005358	CID000005372	CID000005376	CID000005379
CID000005381	CID000005394	CID000005396	CID000005401	CID000005402
CID000005408	CID000005412	CID000005426	CID000005453	CID000005454
CID000005466	CID000005472	CID000005478	CID000005479	CID000005487
CID000005496	CID000005504	CID000005514	CID000005515	CID000005523
CID000005525	CID000005530	CID000005533	CID000005538	CID000005544
CID000005546	CID000005566	CID000005578	CID000005582	CID000005584
CID000005596	CID000005625	CID000005645	CID000005647	CID000005650
CID000005651	CID000005656	CID000005665	CID000005672	CID000005717
CID000005718	CID000005719	CID000005721	CID000005726	CID000005731
CID000005732	CID000005734	CID000005735	CID000005978	CID000006049
CID000006058	CID000006476	CID000006691	CID000007029	CID000010631
CID000012536	CID000012620	CID000014888	CID000016362	CID000018140
CID000019090	CID000020585	CID000023897	CID000025419	CID000027661
CID000027686	CID000027991	CID000028112	CID000031378	CID000031477
CID000032800	CID000034312	CID000038904	CID000039042	CID000039860
CID000040159	CID000040976	CID000041317	CID000041693	CID000041774
CID000041781	CID000042615	CID000044564	CID000047319	CID000047320
CID000047641	CID000047725	CID000048175	CID000050294	CID000050614
CID000051577	CID000052421	CID000054454	CID000054547	CID000054688
CID000054786	CID000057469	CID000057537	CID000059708	CID000060164
CID000060184	CID000060198	CID000060613	CID000060714	CID000060754
CID000060787	CID000060795	CID000060843	CID000060852	CID000060871
CID000060877	CID000060953	CID000062816	CID000062819	CID000062867
CID000062924	CID000062959	CID000065027	CID000065999	CID000068740
CID000071158	CID000071273	CID000071301	CID000071329	CID000071616
CID000072054	CID000072938	CID000074989	CID000077992	CID000077993
CID000077999	CID000082146	CID000083786	CID000093860	CID000104741
CID000104758	CID000104865	CID000110634	CID000110635	CID000119607
CID000122316	CID000123606	CID000123631	CID000124087	CID000125017
CID000125889	CID000130881	CID000147912	CID000148192	CID000148211
CID000150310	CID000150311	CID000150610	CID000151165	CID000152945

CID000153941 CID000158440 CID000163742 CID000166548 CID000170361
CID000176870 CID000197712 CID000213039 CID000216239 CID000216326
CID000443871 CID000444013 CID000444033 CID000450096 CID000477468
CID000657298 CID001349907 CID002761171 CID003002190 CID003062316
CID003081884 CID004183806 CID004479097 CID004659568 CID004659569
CID005229711 CID005281104 CID005282044 CID005311027 CID005311297
CID005329102 CID005353980 CID005361912 CID005362420 CID005381226
CID005481350 CID005487301 CID005493381 CID005493444 CID006323497
CID006398525 CID006398970 CID006435110 CID006436173 CID006447131
CID006918453 CID009571074 CID011947681 CID011954225

(5) $\mathbb{S}_5$: 588 drug compounds having side effect “Dizziness”

CID000000085 CID000000158 CID000000159 CID000000191 CID000000214
CID000000444 CID000000450 CID000000453 CID000000564 CID000000596
CID000000598 CID000000727 CID000000738 CID000000767 CID000000807
CID000000838 CID000000937 CID000000942 CID000001003 CID000001065
CID000001125 CID000001134 CID000001148 CID000001206 CID000001546
CID000001690 CID000001775 CID000001935 CID000001971 CID000001972
CID000001978 CID000001986 CID000002021 CID000002022 CID000002082
CID000002083 CID000002092 CID000002123 CID000002130 CID000002140
CID000002141 CID000002145 CID000002156 CID000002160 CID000002162
CID000002170 CID000002171 CID000002179 CID000002182 CID000002187
CID000002215 CID000002216 CID000002249 CID000002250 CID000002267
CID000002269 CID000002274 CID000002284 CID000002311 CID000002315
CID000002349 CID000002369 CID000002375 CID000002405 CID000002431
CID000002435 CID000002441 CID000002443 CID000002462 CID000002471
CID000002474 CID000002476 CID000002478 CID000002487 CID000002512
CID000002520 CID000002541 CID000002550 CID000002554 CID000002564
CID000002576 CID000002578 CID000002583 CID000002585 CID000002609
CID000002610 CID000002622 CID000002646 CID000002650 CID000002654
CID000002656 CID000002658 CID000002662 CID000002666 CID000002673
CID000002675 CID000002676 CID000002678 CID000002720 CID000002725
CID000002726 CID000002727 CID000002732 CID000002733 CID000002751
CID000002756 CID000002762 CID000002764 CID000002769 CID000002771
CID000002781 CID000002786 CID000002794 CID000002800 CID000002801
CID000002803 CID000002806 CID000002818 CID000002891 CID000002895
CID000002907 CID000002909 CID000002913 CID000002949 CID000002951
CID000002958 CID000002973 CID000002978 CID000002983 CID000002995
CID000003007 CID000003009 CID000003015 CID000003016 CID000003019
CID000003032 CID000003042 CID000003059 CID000003062 CID000003066
CID000003075 CID000003100 CID000003108 CID000003114 CID000003121
CID000003143 CID000003148 CID000003151 CID000003152 CID000003154
CID000003156 CID000003157 CID000003158 CID000003168 CID000003203
CID000003222 CID000003255 CID000003261 CID000003279 CID000003285

CID000003291	CID000003292	CID000003308	CID000003310	CID000003324
CID000003325	CID000003333	CID000003339	CID000003340	CID000003342
CID000003345	CID000003348	CID000003350	CID000003355	CID000003365
CID000003373	CID000003379	CID000003381	CID000003393	CID000003394
CID000003403	CID000003404	CID000003406	CID000003410	CID000003414
CID000003417	CID000003419	CID000003440	CID000003446	CID000003449
CID000003454	CID000003463	CID000003467	CID000003475	CID000003476
CID000003478	CID000003494	CID000003510	CID000003512	CID000003518
CID000003519	CID000003636	CID000003637	CID000003639	CID000003647
CID000003648	CID000003652	CID000003657	CID000003661	CID000003672
CID000003675	CID000003676	CID000003690	CID000003696	CID000003702
CID000003706	CID000003715	CID000003724	CID000003734	CID000003736
CID000003739	CID000003746	CID000003749	CID000003750	CID000003759
CID000003779	CID000003783	CID000003784	CID000003793	CID000003823
CID000003825	CID000003826	CID000003827	CID000003869	CID000003877
CID000003883	CID000003899	CID000003902	CID000003911	CID000003914
CID000003929	CID000003937	CID000003948	CID000003954	CID000003956
CID000003957	CID000003958	CID000003961	CID000003962	CID000003964
CID000004011	CID000004032	CID000004036	CID000004044	CID000004046
CID000004054	CID000004057	CID000004058	CID000004060	CID000004062
CID000004064	CID000004075	CID000004078	CID000004086	CID000004091
CID000004095	CID000004107	CID000004112	CID000004114	CID000004121
CID000004138	CID000004140	CID000004158	CID000004163	CID000004168
CID000004170	CID000004171	CID000004173	CID000004174	CID000004178
CID000004192	CID000004195	CID000004196	CID000004200	CID000004201
CID000004211	CID000004236	CID000004253	CID000004259	CID000004264
CID000004409	CID000004411	CID000004419	CID000004421	CID000004428
CID000004436	CID000004440	CID000004449	CID000004451	CID000004473
CID000004485	CID000004493	CID000004497	CID000004506	CID000004509
CID000004510	CID000004513	CID000004536	CID000004539	CID000004542
CID000004543	CID000004583	CID000004585	CID000004594	CID000004595
CID000004599	CID000004601	CID000004603	CID000004609	CID000004614
CID000004616	CID000004635	CID000004666	CID000004679	CID000004723
CID000004724	CID000004736	CID000004737	CID000004739	CID000004740
CID000004745	CID000004748	CID000004771	CID000004812	CID000004819
CID000004828	CID000004834	CID000004845	CID000004856	CID000004865
CID000004870	CID000004885	CID000004889	CID000004891	CID000004893
CID000004911	CID000004913	CID000004914	CID000004915	CID000004917
CID000004920	CID000004927	CID000004932	CID000004934	CID000004943
CID000004946	CID000004976	CID000005002	CID000005005	CID000005029
CID000005038	CID000005039	CID000005040	CID000005052	CID000005064
CID000005070	CID000005071	CID000005073	CID000005076	CID000005077
CID000005078	CID000005090	CID000005095	CID000005152	CID000005155
CID000005193	CID000005195	CID000005206	CID000005210	CID000005212

CID000005245	CID000005253	CID000005291	CID000005344	CID000005352
CID000005358	CID000005372	CID000005376	CID000005379	CID000005391
CID000005394	CID000005401	CID000005402	CID000005403	CID000005408
CID000005412	CID000005430	CID000005453	CID000005466	CID000005472
CID000005478	CID000005479	CID000005486	CID000005487	CID000005496
CID000005503	CID000005508	CID000005512	CID000005514	CID000005516
CID000005523	CID000005525	CID000005526	CID000005530	CID000005533
CID000005538	CID000005544	CID000005546	CID000005556	CID000005566
CID000005572	CID000005584	CID000005625	CID000005636	CID000005645
CID000005647	CID000005650	CID000005651	CID000005665	CID000005672
CID000005717	CID000005718	CID000005719	CID000005721	CID000005726
CID000005731	CID000005732	CID000005734	CID000005735	CID000005775
CID000005978	CID000006058	CID000006476	CID000006691	CID000007029
CID000008612	CID000010100	CID000010631	CID000013342	CID000014888
CID000016362	CID000020585	CID000027400	CID000027661	CID000027686
CID000027991	CID000028112	CID000030623	CID000031477	CID000032800
CID000034312	CID000039042	CID000039860	CID000040159	CID000040976
CID000041317	CID000041693	CID000041781	CID000042113	CID000042615
CID000044564	CID000047725	CID000050294	CID000051263	CID000051634
CID000054454	CID000054547	CID000054688	CID000054786	CID000056959
CID000057469	CID000057537	CID000059708	CID000059768	CID000060184
CID000060198	CID000060612	CID000060613	CID000060714	CID000060753
CID000060754	CID000060787	CID000060795	CID000060852	CID000060865
CID000060877	CID000060953	CID000062816	CID000062819	CID000062867
CID000062924	CID000062959	CID000064147	CID000065027	CID000065999
CID000068740	CID000068844	CID000071158	CID000071273	CID000071301
CID000071329	CID000071616	CID000072054	CID000072938	CID000074989
CID000077992	CID000077993	CID000077999	CID000082146	CID000083786
CID000093860	CID000096312	CID000104741	CID000104865	CID000110634
CID000110635	CID000115237	CID000119182	CID000119607	CID000122316
CID000123606	CID000124087	CID000125017	CID000125889	CID000130881
CID000147912	CID000148192	CID000148211	CID000150310	CID000150311
CID000150610	CID000151165	CID000152945	CID000153941	CID000158440
CID000163742	CID000166548	CID000170361	CID000176870	CID000197712
CID000213039	CID000216239	CID000216326	CID000443871	CID000444013
CID000444033	CID000450096	CID000477468	CID002761171	CID003002190
CID003062316	CID003081884	CID003086672	CID004183806	CID004479097
CID004659568	CID004659569	CID005229711	CID005281104	CID005282044
CID005311027	CID005311181	CID005329102	CID005353894	CID005353980
CID005362420	CID005381226	CID005481350	CID005487301	CID005493381
CID005493444	CID006323497	CID006398525	CID006398970	CID006435110
CID006436173	CID009571074	CID011947681		

(6) $\mathbb{S}_6$: 582 drug compounds having side effect “Diarrhea”

CID000000085	CID000000143	CID000000158	CID000000159	CID000000206
CID000000214	CID000000247	CID000000298	CID000000444	CID000000450
CID000000453	CID000000564	CID000000596	CID000000598	CID000000750
CID000000767	CID000000772	CID000000807	CID000000815	CID000000853
CID000000937	CID000000942	CID000001003	CID000001065	CID000001125
CID000001134	CID000001206	CID000001546	CID000001690	CID000001775
CID000001935	CID000001971	CID000001972	CID000001978	CID000001986
CID000002022	CID000002083	CID000002092	CID000002130	CID000002131
CID000002140	CID000002153	CID000002156	CID000002160	CID000002162
CID000002170	CID000002173	CID000002177	CID000002179	CID000002182
CID000002187	CID000002215	CID000002216	CID000002232	CID000002244
CID000002249	CID000002250	CID000002265	CID000002267	CID000002269
CID000002274	CID000002284	CID000002311	CID000002315	CID000002369
CID000002370	CID000002375	CID000002405	CID000002431	CID000002443
CID000002462	CID000002471	CID000002476	CID000002478	CID000002512
CID000002520	CID000002541	CID000002550	CID000002554	CID000002559
CID000002564	CID000002575	CID000002578	CID000002585	CID000002609
CID000002610	CID000002617	CID000002622	CID000002629	CID000002631
CID000002637	CID000002646	CID000002650	CID000002654	CID000002655
CID000002656	CID000002658	CID000002662	CID000002666	CID000002675
CID000002676	CID000002678	CID000002708	CID000002719	CID000002720
CID000002725	CID000002727	CID000002732	CID000002751	CID000002756
CID000002762	CID000002764	CID000002769	CID000002771	CID000002781
CID000002794	CID000002800	CID000002801	CID000002802	CID000002806
CID000002818	CID000002891	CID000002895	CID000002907	CID000002909
CID000002913	CID000002951	CID000002958	CID000002973	CID000002978
CID000002983	CID000002995	CID000003007	CID000003015	CID000003016
CID000003019	CID000003032	CID000003040	CID000003043	CID000003059
CID000003062	CID000003066	CID000003075	CID000003100	CID000003108
CID000003114	CID000003121	CID000003125	CID000003143	CID000003148
CID000003151	CID000003152	CID000003154	CID000003156	CID000003157
CID000003158	CID000003203	CID000003222	CID000003255	CID000003278
CID000003285	CID000003291	CID000003292	CID000003305	CID000003308
CID000003310	CID000003324	CID000003325	CID000003333	CID000003339
CID000003340	CID000003342	CID000003345	CID000003348	CID000003355
CID000003366	CID000003367	CID000003379	CID000003385	CID000003386
CID000003393	CID000003394	CID000003397	CID000003403	CID000003404
CID000003406	CID000003410	CID000003414	CID000003417	CID000003419
CID000003440	CID000003446	CID000003449	CID000003454	CID000003461
CID000003463	CID000003475	CID000003476	CID000003478	CID000003488
CID000003510	CID000003512	CID000003518	CID000003519	CID000003559
CID000003636	CID000003637	CID000003639	CID000003647	CID000003648
CID000003652	CID000003657	CID000003672	CID000003685	CID000003690
CID000003696	CID000003702	CID000003706	CID000003715	CID000003724

CID000003730	CID000003736	CID000003741	CID000003746	CID000003749
CID000003759	CID000003784	CID000003793	CID000003823	CID000003825
CID000003826	CID000003827	CID000003869	CID000003877	CID000003878
CID000003883	CID000003899	CID000003902	CID000003911	CID000003914
CID000003928	CID000003929	CID000003937	CID000003948	CID000003956
CID000003957	CID000003961	CID000003962	CID000003998	CID000004011
CID000004033	CID000004036	CID000004044	CID000004046	CID000004053
CID000004054	CID000004064	CID000004086	CID000004091	CID000004095
CID000004100	CID000004112	CID000004121	CID000004138	CID000004140
CID000004158	CID000004163	CID000004168	CID000004170	CID000004171
CID000004173	CID000004178	CID000004196	CID000004200	CID000004201
CID000004205	CID000004211	CID000004212	CID000004236	CID000004253
CID000004259	CID000004264	CID000004409	CID000004411	CID000004421
CID000004425	CID000004428	CID000004440	CID000004449	CID000004451
CID000004463	CID000004473	CID000004485	CID000004493	CID000004497
CID000004506	CID000004509	CID000004513	CID000004539	CID000004542
CID000004543	CID000004547	CID000004583	CID000004585	CID000004594
CID000004595	CID000004603	CID000004607	CID000004609	CID000004614
CID000004634	CID000004635	CID000004638	CID000004645	CID000004666
CID000004679	CID000004689	CID000004691	CID000004724	CID000004727
CID000004730	CID000004736	CID000004739	CID000004740	CID000004745
CID000004748	CID000004771	CID000004819	CID000004828	CID000004829
CID000004834	CID000004845	CID000004856	CID000004870	CID000004873
CID000004885	CID000004889	CID000004891	CID000004893	CID000004913
CID000004915	CID000004920	CID000004932	CID000004943	CID000004946
CID000004976	CID000004991	CID000005002	CID000005005	CID000005029
CID000005035	CID000005038	CID000005039	CID000005040	CID000005052
CID000005064	CID000005070	CID000005071	CID000005073	CID000005076
CID000005077	CID000005078	CID000005090	CID000005095	CID000005152
CID000005155	CID000005195	CID000005203	CID000005210	CID000005212
CID000005215	CID000005245	CID000005253	CID000005267	CID000005291
CID000005300	CID000005344	CID000005352	CID000005358	CID000005372
CID000005376	CID000005391	CID000005394	CID000005396	CID000005401
CID000005402	CID000005408	CID000005412	CID000005426	CID000005430
CID000005452	CID000005454	CID000005466	CID000005472	CID000005478
CID000005479	CID000005487	CID000005496	CID000005504	CID000005508
CID000005512	CID000005514	CID000005515	CID000005523	CID000005525
CID000005526	CID000005530	CID000005533	CID000005538	CID000005544
CID000005546	CID000005556	CID000005584	CID000005625	CID000005645
CID000005647	CID000005650	CID000005656	CID000005665	CID000005672
CID000005717	CID000005718	CID000005721	CID000005726	CID000005731
CID000005732	CID000005734	CID000005735	CID000005746	CID000005775
CID000005978	CID000006049	CID000006058	CID000006476	CID000006691
CID000007029	CID000009433	CID000009904	CID000010631	CID000013342

CID000014888	CID000018140	CID000019090	CID000020585	CID000025419
CID000027661	CID000027991	CID000028112	CID000030623	CID000032800
CID000034312	CID000038904	CID000039042	CID000039860	CID000040159
CID000040976	CID000041317	CID000041744	CID000041774	CID000041781
CID000042615	CID000047725	CID000050294	CID000050614	CID000051577
CID000051634	CID000054454	CID000054547	CID000054688	CID000054786
CID000057469	CID000057537	CID000059708	CID000060184	CID000060198
CID000060612	CID000060613	CID000060714	CID000060753	CID000060754
CID000060787	CID000060795	CID000060843	CID000060852	CID000060865
CID000060871	CID000060877	CID000060953	CID000062816	CID000062819
CID000062924	CID000062959	CID000064147	CID000065027	CID000065999
CID000068740	CID000068844	CID000071158	CID000071273	CID000071301
CID000071329	CID000071616	CID000072054	CID000072938	CID000074989
CID000077992	CID000077993	CID000077999	CID000082146	CID000083786
CID000093860	CID000096312	CID000104741	CID000104758	CID000104865
CID000110634	CID000110635	CID000115237	CID000119182	CID000119607
CID000122316	CID000123606	CID000123620	CID000123631	CID000124087
CID000125017	CID000125889	CID000130881	CID000147912	CID000148192
CID000148211	CID000150310	CID000150311	CID000150610	CID000151165
CID000153941	CID000158440	CID000160051	CID000163742	CID000166548
CID000170361	CID000176168	CID000176870	CID000197712	CID000213039
CID000216239	CID000216326	CID000443871	CID000444013	CID000477468
CID000667490	CID002761171	CID003002190	CID003062316	CID003081884
CID004183806	CID004479097	CID004659568	CID004659569	CID005229711
CID005281007	CID005281104	CID005282044	CID005311181	CID005311297
CID005329102	CID005353980	CID005361912	CID005362070	CID005381226
CID005481350	CID005487301	CID005493381	CID005493444	CID006323497
CID006398525	CID006398970	CID006435110	CID006447131	CID006918453
CID009571074	CID011954225			

(7) $\mathbb{S}_7$: 561 drug compounds having side effect “Pruritus”

CID000000085	CID000000137	CID000000143	CID000000159	CID000000214
CID000000271	CID000000298	CID000000444	CID000000564	CID000000581
CID000000596	CID000000727	CID000000738	CID000000767	CID000000772
CID000000807	CID000000853	CID000000937	CID000000942	CID000001003
CID000001046	CID000001065	CID000001134	CID000001546	CID000001775
CID000001935	CID000001972	CID000001978	CID000002022	CID000002083
CID000002088	CID000002092	CID000002118	CID000002123	CID000002130
CID000002140	CID000002141	CID000002145	CID000002156	CID000002162
CID000002170	CID000002177	CID000002182	CID000002187	CID000002216
CID000002244	CID000002249	CID000002250	CID000002266	CID000002269
CID000002274	CID000002284	CID000002311	CID000002315	CID000002349
CID000002366	CID000002369	CID000002375	CID000002405	CID000002435
CID000002441	CID000002462	CID000002471	CID000002474	CID000002476

CID000002477	CID000002478	CID000002484	CID000002487	CID000002512
CID000002520	CID000002522	CID000002524	CID000002541	CID000002550
CID000002554	CID000002559	CID000002575	CID000002576	CID000002585
CID000002609	CID000002610	CID000002617	CID000002622	CID000002631
CID000002637	CID000002646	CID000002650	CID000002654	CID000002655
CID000002656	CID000002658	CID000002662	CID000002673	CID000002675
CID000002676	CID000002708	CID000002719	CID000002727	CID000002749
CID000002751	CID000002762	CID000002764	CID000002769	CID000002771
CID000002786	CID000002794	CID000002800	CID000002801	CID000002802
CID000002803	CID000002806	CID000002812	CID000002818	CID000002891
CID000002895	CID000002907	CID000002909	CID000002949	CID000002951
CID000002955	CID000002958	CID000002973	CID000002978	CID000002995
CID000003000	CID000003003	CID000003009	CID000003015	CID000003016
CID000003032	CID000003040	CID000003042	CID000003043	CID000003059
CID000003066	CID000003075	CID000003105	CID000003108	CID000003114
CID000003121	CID000003143	CID000003148	CID000003151	CID000003152
CID000003154	CID000003156	CID000003157	CID000003198	CID000003203
CID000003219	CID000003222	CID000003251	CID000003255	CID000003261
CID000003279	CID000003285	CID000003305	CID000003308	CID000003310
CID000003325	CID000003333	CID000003339	CID000003342	CID000003345
CID000003350	CID000003355	CID000003365	CID000003366	CID000003367
CID000003372	CID000003373	CID000003375	CID000003379	CID000003381
CID000003382	CID000003385	CID000003386	CID000003392	CID000003393
CID000003394	CID000003403	CID000003404	CID000003405	CID000003410
CID000003414	CID000003417	CID000003419	CID000003440	CID000003446
CID000003454	CID000003461	CID000003463	CID000003467	CID000003475
CID000003476	CID000003478	CID000003488	CID000003494	CID000003510
CID000003519	CID000003553	CID000003637	CID000003640	CID000003648
CID000003652	CID000003672	CID000003675	CID000003685	CID000003696
CID000003702	CID000003706	CID000003715	CID000003724	CID000003734
CID000003736	CID000003737	CID000003741	CID000003742	CID000003746
CID000003749	CID000003784	CID000003793	CID000003823	CID000003825
CID000003869	CID000003877	CID000003878	CID000003883	CID000003890
CID000003899	CID000003902	CID000003911	CID000003914	CID000003929
CID000003937	CID000003948	CID000003954	CID000003956	CID000003961
CID000003962	CID000003964	CID000003998	CID000004011	CID000004036
CID000004044	CID000004046	CID000004053	CID000004054	CID000004058
CID000004062	CID000004064	CID000004075	CID000004078	CID000004086
CID000004091	CID000004095	CID000004101	CID000004107	CID000004112
CID000004114	CID000004158	CID000004170	CID000004171	CID000004173
CID000004192	CID000004195	CID000004196	CID000004200	CID000004201
CID000004205	CID000004236	CID000004253	CID000004259	CID000004264
CID000004409	CID000004411	CID000004419	CID000004421	CID000004428
CID000004440	CID000004449	CID000004451	CID000004463	CID000004485

CID000004493	CID000004497	CID000004509	CID000004510	CID000004513
CID000004536	CID000004539	CID000004542	CID000004543	CID000004583
CID000004585	CID000004594	CID000004595	CID000004599	CID000004601
CID000004607	CID000004609	CID000004614	CID000004623	CID000004634
CID000004635	CID000004666	CID000004679	CID000004691	CID000004725
CID000004727	CID000004736	CID000004739	CID000004740	CID000004745
CID000004748	CID000004819	CID000004828	CID000004834	CID000004845
CID000004856	CID000004865	CID000004885	CID000004889	CID000004893
CID000004911	CID000004913	CID000004914	CID000004915	CID000004917
CID000004920	CID000004932	CID000004943	CID000004976	CID000005002
CID000005005	CID000005029	CID000005038	CID000005039	CID000005040
CID000005052	CID000005064	CID000005070	CID000005073	CID000005076
CID000005077	CID000005078	CID000005090	CID000005095	CID000005155
CID000005195	CID000005203	CID000005206	CID000005210	CID000005212
CID000005215	CID000005245	CID000005253	CID000005291	CID000005318
CID000005320	CID000005344	CID000005352	CID000005358	CID000005359
CID000005372	CID000005376	CID000005379	CID000005394	CID000005396
CID000005401	CID000005402	CID000005404	CID000005408	CID000005426
CID000005430	CID000005453	CID000005454	CID000005466	CID000005472
CID000005478	CID000005479	CID000005487	CID000005496	CID000005503
CID000005505	CID000005514	CID000005515	CID000005516	CID000005523
CID000005525	CID000005533	CID000005538	CID000005544	CID000005556
CID000005566	CID000005578	CID000005582	CID000005584	CID000005596
CID000005625	CID000005636	CID000005645	CID000005647	CID000005650
CID000005651	CID000005656	CID000005672	CID000005717	CID000005718
CID000005719	CID000005726	CID000005731	CID000005732	CID000005734
CID000005735	CID000005775	CID000006691	CID000007187	CID000008612
CID000009034	CID000010631	CID000012536	CID000012620	CID000014888
CID000016362	CID000016850	CID000018140	CID000019090	CID000020585
CID000027661	CID000027991	CID000028112	CID000032797	CID000034312
CID000036811	CID000038904	CID000039042	CID000039507	CID000039860
CID000040159	CID000040976	CID000041317	CID000041693	CID000041744
CID000042113	CID000042615	CID000044564	CID000047319	CID000047320
CID000047641	CID000048175	CID000050614	CID000051263	CID000052421
CID000054454	CID000054547	CID000054688	CID000054786	CID000057469
CID000057537	CID000059708	CID000059768	CID000060164	CID000060184
CID000060198	CID000060613	CID000060714	CID000060754	CID000060787
CID000060795	CID000060843	CID000060871	CID000060877	CID000060953
CID000062819	CID000062867	CID000062924	CID000062959	CID000064147
CID000065027	CID000065863	CID000065999	CID000068740	CID000068844
CID000071158	CID000071273	CID000071301	CID000071616	CID000072054
CID000072938	CID000074989	CID000077992	CID000077993	CID000077999
CID000082146	CID000083786	CID000093860	CID000104741	CID000104758
CID000104865	CID000110634	CID000110635	CID000115237	CID000119182

CID000119607	CID000122316	CID000123606	CID000123620	CID000123631
CID000125889	CID000130881	CID000147912	CID000148192	CID000148211
CID000150610	CID000151165	CID000152945	CID000158440	CID000163742
CID000166548	CID000170361	CID000176870	CID000197712	CID000213039
CID000216239	CID000216326	CID000443871	CID000444013	CID000450096
CID000477468	CID000657298	CID001349907	CID003002190	CID003062316
CID003081884	CID003086672	CID004479097	CID004630253	CID004659568
CID004659569	CID005281104	CID005282044	CID005282226	CID005311027
CID005311297	CID005353980	CID005362070	CID005362420	CID005381226
CID005481350	CID005487301	CID006323497	CID006398525	CID006398970
CID006435110	CID006436173	CID006447131	CID006918453	CID009571074
CID011954225				

(8) $\mathbb{S}_8$: 490 drug compounds having side effect “Urticaria”

CID000000119	CID000000143	CID000000159	CID000000298	CID000000444
CID000000450	CID000000453	CID000000581	CID000000596	CID000000727
CID000000750	CID000000772	CID000000807	CID000000853	CID000000937
CID000001003	CID000001046	CID000001065	CID000001206	CID000001546
CID000001690	CID000001775	CID000001935	CID000001971	CID000001972
CID000001978	CID000001986	CID000002021	CID000002022	CID000002082
CID000002083	CID000002088	CID000002092	CID000002099	CID000002118
CID000002140	CID000002141	CID000002145	CID000002148	CID000002160
CID000002162	CID000002170	CID000002171	CID000002173	CID000002179
CID000002182	CID000002187	CID000002244	CID000002250	CID000002269
CID000002274	CID000002284	CID000002308	CID000002315	CID000002349
CID000002366	CID000002369	CID000002375	CID000002405	CID000002462
CID000002471	CID000002474	CID000002476	CID000002477	CID000002478
CID000002487	CID000002520	CID000002524	CID000002541	CID000002550
CID000002554	CID000002559	CID000002575	CID000002585	CID000002609
CID000002610	CID000002617	CID000002622	CID000002631	CID000002637
CID000002646	CID000002650	CID000002654	CID000002656	CID000002658
CID000002662	CID000002666	CID000002673	CID000002675	CID000002676
CID000002708	CID000002713	CID000002720	CID000002726	CID000002727
CID000002732	CID000002751	CID000002762	CID000002764	CID000002769
CID000002771	CID000002786	CID000002800	CID000002801	CID000002802
CID000002803	CID000002806	CID000002812	CID000002818	CID000002891
CID000002895	CID000002907	CID000002909	CID000002949	CID000002951
CID000002958	CID000002973	CID000002983	CID000002995	CID000003007
CID000003015	CID000003016	CID000003032	CID000003040	CID000003042
CID000003059	CID000003066	CID000003075	CID000003108	CID000003114
CID000003125	CID000003148	CID000003151	CID000003152	CID000003154
CID000003157	CID000003203	CID000003222	CID000003255	CID000003261
CID000003285	CID000003291	CID000003305	CID000003308	CID000003310
CID000003324	CID000003325	CID000003333	CID000003339	CID000003342

CID000003345	CID000003350	CID000003354	CID000003355	CID000003365
CID000003366	CID000003372	CID000003373	CID000003379	CID000003385
CID000003386	CID000003394	CID000003403	CID000003410	CID000003419
CID000003440	CID000003446	CID000003454	CID000003463	CID000003467
CID000003475	CID000003476	CID000003478	CID000003488	CID000003494
CID000003510	CID000003512	CID000003636	CID000003637	CID000003639
CID000003647	CID000003648	CID000003652	CID000003672	CID000003676
CID000003685	CID000003696	CID000003702	CID000003706	CID000003715
CID000003724	CID000003734	CID000003736	CID000003737	CID000003741
CID000003742	CID000003746	CID000003749	CID000003784	CID000003793
CID000003823	CID000003825	CID000003826	CID000003827	CID000003869
CID000003877	CID000003878	CID000003883	CID000003899	CID000003902
CID000003911	CID000003914	CID000003928	CID000003929	CID000003937
CID000003948	CID000003954	CID000003956	CID000003961	CID000003962
CID000003998	CID000004030	CID000004036	CID000004044	CID000004046
CID000004053	CID000004054	CID000004057	CID000004058	CID000004060
CID000004062	CID000004064	CID000004075	CID000004086	CID000004091
CID000004095	CID000004100	CID000004107	CID000004112	CID000004114
CID000004121	CID000004158	CID000004168	CID000004170	CID000004171
CID000004173	CID000004189	CID000004192	CID000004196	CID000004200
CID000004201	CID000004205	CID000004212	CID000004236	CID000004253
CID000004259	CID000004264	CID000004409	CID000004419	CID000004421
CID000004428	CID000004440	CID000004449	CID000004451	CID000004463
CID000004485	CID000004493	CID000004509	CID000004513	CID000004536
CID000004539	CID000004542	CID000004543	CID000004583	CID000004585
CID000004594	CID000004595	CID000004599	CID000004601	CID000004603
CID000004607	CID000004609	CID000004614	CID000004616	CID000004635
CID000004645	CID000004679	CID000004691	CID000004725	CID000004727
CID000004730	CID000004739	CID000004740	CID000004748	CID000004771
CID000004834	CID000004856	CID000004870	CID000004885	CID000004889
CID000004891	CID000004893	CID000004894	CID000004900	CID000004911
CID000004913	CID000004914	CID000004915	CID000004917	CID000004920
CID000004932	CID000004934	CID000004943	CID000004946	CID000004976
CID000004991	CID000005005	CID000005029	CID000005038	CID000005039
CID000005070	CID000005073	CID000005076	CID000005077	CID000005078
CID000005090	CID000005095	CID000005152	CID000005195	CID000005203
CID000005206	CID000005210	CID000005212	CID000005215	CID000005245
CID000005267	CID000005297	CID000005344	CID000005358	CID000005372
CID000005379	CID000005396	CID000005402	CID000005408	CID000005412
CID000005426	CID000005452	CID000005453	CID000005454	CID000005466
CID000005472	CID000005478	CID000005479	CID000005486	CID000005487
CID000005496	CID000005503	CID000005505	CID000005508	CID000005514
CID000005515	CID000005523	CID000005530	CID000005533	CID000005538
CID000005544	CID000005566	CID000005584	CID000005596	CID000005625

CID000005645 CID000005647 CID000005650 CID000005651 CID000005656
CID000005672 CID000005717 CID000005718 CID000005719 CID000005721
CID000005726 CID000005731 CID000005732 CID000005734 CID000005735
CID000006058 CID000006476 CID000006691 CID000007029 CID000008612
CID000009034 CID000010631 CID000014888 CID000016362 CID000016850
CID000020585 CID000030623 CID000031378 CID000032797 CID000034312
CID000038904 CID000039042 CID000041317 CID000041774 CID000042615
CID000047319 CID000047725 CID000048175 CID000050614 CID000051263
CID000052421 CID000054454 CID000054547 CID000054688 CID000059768
CID000060164 CID000060184 CID000060613 CID000060714 CID000060754
CID000060787 CID000060795 CID000060877 CID000060953 CID000062816
CID000062867 CID000062924 CID000062959 CID000064147 CID000065999
CID000068740 CID000068844 CID000071158 CID000071273 CID000071301
CID000071616 CID000072938 CID000074989 CID000077993 CID000077999
CID000083786 CID000093860 CID000104741 CID000104865 CID000110635
CID000119607 CID000122316 CID000123631 CID000125889 CID000130881
CID000147912 CID000148192 CID000150311 CID000150610 CID000151165
CID000152945 CID000158440 CID000166548 CID000170361 CID000216239
CID000216326 CID000443871 CID000444013 CID000444033 CID000477468
CID000657298 CID001349907 CID003002190 CID003062316 CID003081884
CID004479097 CID004659568 CID004659569 CID005281007 CID005281104
CID005282044 CID005311297 CID005353980 CID005362420 CID005381226
CID005481350 CID005487301 CID005493381 CID006323497 CID006398525
CID006435110 CID006436173 CID006447131 CID006918453 CID009571074

(9) $\mathbb{S}_9$: 480 drug compounds having side effect “Fever”

CID000000085 CID000000158 CID000000159 CID000000214 CID000000298
CID000000444 CID000000450 CID000000453 CID000000581 CID000000596
CID000000598 CID000000738 CID000000767 CID000000772 CID000000807
CID000000815 CID000000853 CID000000861 CID000000937 CID000000942
CID000001046 CID000001065 CID000001125 CID000001134 CID000001546
CID000001775 CID000001935 CID000001971 CID000001978 CID000001986
CID000002021 CID000002082 CID000002083 CID000002088 CID000002118
CID000002130 CID000002141 CID000002142 CID000002145 CID000002156
CID000002160 CID000002170 CID000002179 CID000002182 CID000002187
CID000002232 CID000002249 CID000002250 CID000002265 CID000002267
CID000002269 CID000002274 CID000002284 CID000002311 CID000002315
CID000002344 CID000002369 CID000002375 CID000002405 CID000002431
CID000002462 CID000002474 CID000002476 CID000002477 CID000002478
CID000002487 CID000002524 CID000002541 CID000002550 CID000002554
CID000002559 CID000002575 CID000002576 CID000002578 CID000002585
CID000002609 CID000002610 CID000002622 CID000002629 CID000002631
CID000002637 CID000002646 CID000002650 CID000002654 CID000002655
CID000002656 CID000002658 CID000002662 CID000002666 CID000002675

CID000002676	CID000002678	CID000002708	CID000002720	CID000002726
CID000002756	CID000002769	CID000002771	CID000002794	CID000002800
CID000002801	CID000002802	CID000002803	CID000002806	CID000002818
CID000002907	CID000002909	CID000002949	CID000002955	CID000002958
CID000002973	CID000002995	CID000003007	CID000003015	CID000003016
CID000003019	CID000003032	CID000003040	CID000003043	CID000003059
CID000003066	CID000003075	CID000003114	CID000003121	CID000003143
CID000003148	CID000003152	CID000003154	CID000003156	CID000003157
CID000003158	CID000003203	CID000003222	CID000003261	CID000003278
CID000003279	CID000003285	CID000003292	CID000003308	CID000003310
CID000003324	CID000003325	CID000003333	CID000003339	CID000003340
CID000003342	CID000003345	CID000003348	CID000003355	CID000003365
CID000003366	CID000003367	CID000003372	CID000003379	CID000003381
CID000003386	CID000003394	CID000003403	CID000003404	CID000003406
CID000003410	CID000003414	CID000003417	CID000003419	CID000003440
CID000003446	CID000003449	CID000003454	CID000003461	CID000003467
CID000003475	CID000003494	CID000003510	CID000003637	CID000003639
CID000003648	CID000003657	CID000003675	CID000003676	CID000003685
CID000003690	CID000003696	CID000003698	CID000003702	CID000003706
CID000003715	CID000003724	CID000003730	CID000003734	CID000003736
CID000003737	CID000003739	CID000003741	CID000003742	CID000003749
CID000003750	CID000003784	CID000003823	CID000003826	CID000003827
CID000003869	CID000003877	CID000003883	CID000003899	CID000003902
CID000003911	CID000003928	CID000003929	CID000003937	CID000003961
CID000003962	CID000004011	CID000004044	CID000004046	CID000004053
CID000004064	CID000004075	CID000004078	CID000004095	CID000004107
CID000004112	CID000004121	CID000004138	CID000004158	CID000004163
CID000004168	CID000004171	CID000004173	CID000004178	CID000004196
CID000004201	CID000004205	CID000004212	CID000004236	CID000004253
CID000004259	CID000004409	CID000004411	CID000004419	CID000004425
CID000004428	CID000004440	CID000004449	CID000004451	CID000004463
CID000004485	CID000004493	CID000004509	CID000004513	CID000004539
CID000004542	CID000004543	CID000004583	CID000004585	CID000004594
CID000004595	CID000004607	CID000004609	CID000004614	CID000004616
CID000004635	CID000004666	CID000004679	CID000004691	CID000004724
CID000004727	CID000004730	CID000004737	CID000004739	CID000004740
CID000004745	CID000004748	CID000004819	CID000004828	CID000004834
CID000004856	CID000004885	CID000004889	CID000004891	CID000004893
CID000004911	CID000004913	CID000004915	CID000004917	CID000004920
CID000004932	CID000004943	CID000004946	CID000004976	CID000005002
CID000005029	CID000005035	CID000005038	CID000005039	CID000005040
CID000005064	CID000005070	CID000005073	CID000005076	CID000005077
CID000005078	CID000005090	CID000005095	CID000005152	CID000005155
CID000005193	CID000005195	CID000005203	CID000005206	CID000005210

CID000005212	CID000005215	CID000005245	CID000005253	CID000005267
CID000005291	CID000005297	CID000005300	CID000005344	CID000005352
CID000005358	CID000005372	CID000005379	CID000005394	CID000005396
CID000005401	CID000005402	CID000005404	CID000005426	CID000005452
CID000005453	CID000005466	CID000005478	CID000005479	CID000005486
CID000005487	CID000005496	CID000005508	CID000005514	CID000005515
CID000005523	CID000005525	CID000005533	CID000005538	CID000005544
CID000005566	CID000005578	CID000005582	CID000005584	CID000005591
CID000005625	CID000005645	CID000005651	CID000005656	CID000005665
CID000005672	CID000005717	CID000005718	CID000005719	CID000005726
CID000005731	CID000005732	CID000005735	CID000005746	CID000005978
CID000006049	CID000006058	CID000006476	CID000006691	CID000010631
CID000014888	CID000019090	CID000027661	CID000028112	CID000030623
CID000034312	CID000038904	CID000039860	CID000040159	CID000040976
CID000041317	CID000041693	CID000041744	CID000042113	CID000042615
CID000047725	CID000050294	CID000050614	CID000054454	CID000054547
CID000054786	CID000057469	CID000059708	CID000059768	CID000060184
CID000060198	CID000060612	CID000060613	CID000060714	CID000060754
CID000060787	CID000060795	CID000060843	CID000060871	CID000060877
CID000060953	CID000062819	CID000062924	CID000062959	CID000064147
CID000065027	CID000065999	CID000068740	CID000071158	CID000071273
CID000071616	CID000072938	CID000074989	CID000077992	CID000077993
CID000082146	CID000083786	CID000093860	CID000096312	CID000104741
CID000104758	CID000104865	CID000115237	CID000119182	CID000119607
CID000122316	CID000123606	CID000123620	CID000123631	CID000124087
CID000125889	CID000130881	CID000145068	CID000147912	CID000148192
CID000148211	CID000150610	CID000151165	CID000158440	CID000163742
CID000166548	CID000170361	CID000176870	CID000197712	CID000213039
CID000216239	CID000216326	CID000443871	CID000444013	CID000477468
CID000657298	CID000667490	CID001349907	CID003062316	CID003081884
CID004183806	CID004659568	CID004659569	CID005229711	CID005281007
CID005281104	CID005282044	CID005311297	CID005329102	CID005353980
CID005361912	CID005362070	CID005362420	CID005381226	CID005481350
CID005493381	CID006398970	CID006435110	CID006447131	CID009571074

(10) $\mathbb{S}_{10}$: 461 drug compounds having side effect “Edema”

CID000000137	CID000000159	CID000000187	CID000000191	CID000000206
CID000000214	CID000000247	CID000000444	CID000000450	CID000000564
CID000000581	CID000000596	CID000000598	CID000000738	CID000000750
CID000000772	CID000000807	CID000000937	CID000000942	CID000001003
CID000001546	CID000001690	CID000001775	CID000001935	CID000001971
CID000001972	CID000001978	CID000002019	CID000002022	CID000002083
CID000002092	CID000002118	CID000002130	CID000002140	CID000002141
CID000002156	CID000002160	CID000002162	CID000002170	CID000002182

CID000002187	CID000002215	CID000002216	CID000002249	CID000002250
CID000002266	CID000002267	CID000002269	CID000002284	CID000002311
CID000002349	CID000002369	CID000002375	CID000002405	CID000002435
CID000002443	CID000002462	CID000002474	CID000002477	CID000002478
CID000002487	CID000002512	CID000002520	CID000002554	CID000002578
CID000002583	CID000002585	CID000002609	CID000002650	CID000002656
CID000002662	CID000002675	CID000002676	CID000002678	CID000002712
CID000002713	CID000002726	CID000002749	CID000002751	CID000002762
CID000002764	CID000002769	CID000002771	CID000002794	CID000002800
CID000002801	CID000002802	CID000002803	CID000002806	CID000002812
CID000002818	CID000002895	CID000002907	CID000002909	CID000002913
CID000002949	CID000002958	CID000002973	CID000002995	CID000003009
CID000003015	CID000003032	CID000003040	CID000003059	CID000003066
CID000003075	CID000003108	CID000003114	CID000003121	CID000003125
CID000003143	CID000003148	CID000003151	CID000003152	CID000003154
CID000003157	CID000003158	CID000003222	CID000003251	CID000003261
CID000003285	CID000003308	CID000003325	CID000003333	CID000003339
CID000003345	CID000003350	CID000003355	CID000003365	CID000003367
CID000003372	CID000003379	CID000003381	CID000003385	CID000003386
CID000003394	CID000003397	CID000003403	CID000003404	CID000003410
CID000003414	CID000003419	CID000003446	CID000003449	CID000003454
CID000003461	CID000003463	CID000003467	CID000003475	CID000003478
CID000003494	CID000003510	CID000003518	CID000003519	CID000003637
CID000003648	CID000003652	CID000003657	CID000003672	CID000003675
CID000003676	CID000003696	CID000003715	CID000003724	CID000003734
CID000003736	CID000003737	CID000003741	CID000003742	CID000003746
CID000003749	CID000003750	CID000003784	CID000003793	CID000003825
CID000003826	CID000003869	CID000003878	CID000003883	CID000003890
CID000003902	CID000003911	CID000003915	CID000003929	CID000003937
CID000003948	CID000003958	CID000003961	CID000003964	CID000003998
CID000004011	CID000004036	CID000004044	CID000004046	CID000004053
CID000004060	CID000004062	CID000004075	CID000004078	CID000004086
CID000004091	CID000004095	CID000004114	CID000004138	CID000004158
CID000004163	CID000004168	CID000004171	CID000004173	CID000004178
CID000004189	CID000004201	CID000004205	CID000004212	CID000004236
CID000004253	CID000004259	CID000004409	CID000004419	CID000004428
CID000004440	CID000004449	CID000004451	CID000004463	CID000004473
CID000004485	CID000004493	CID000004497	CID000004513	CID000004536
CID000004539	CID000004542	CID000004543	CID000004547	CID000004583
CID000004585	CID000004594	CID000004595	CID000004603	CID000004607
CID000004609	CID000004614	CID000004616	CID000004634	CID000004635
CID000004638	CID000004666	CID000004679	CID000004691	CID000004725
CID000004730	CID000004736	CID000004739	CID000004740	CID000004745
CID000004748	CID000004775	CID000004819	CID000004828	CID000004829

CID000004845 CID000004856 CID000004865 CID000004885 CID000004889
CID000004893 CID000004913 CID000004914 CID000004915 CID000004917
CID000004920 CID000004927 CID000004932 CID000004943 CID000004976
CID000005002 CID000005005 CID000005029 CID000005038 CID000005039
CID000005040 CID000005052 CID000005070 CID000005073 CID000005076
CID000005077 CID000005078 CID000005095 CID000005152 CID000005195
CID000005203 CID000005210 CID000005212 CID000005215 CID000005234
CID000005253 CID000005291 CID000005300 CID000005320 CID000005344
CID000005352 CID000005358 CID000005372 CID000005376 CID000005379
CID000005381 CID000005394 CID000005396 CID000005401 CID000005408
CID000005426 CID000005452 CID000005453 CID000005454 CID000005466
CID000005478 CID000005486 CID000005487 CID000005504 CID000005508
CID000005512 CID000005514 CID000005516 CID000005523 CID000005525
CID000005530 CID000005533 CID000005538 CID000005566 CID000005584
CID000005591 CID000005596 CID000005625 CID000005636 CID000005645
CID000005647 CID000005650 CID000005656 CID000005665 CID000005717
CID000005718 CID000005719 CID000005721 CID000005726 CID000005731
CID000005732 CID000005734 CID000005735 CID000005746 CID000005978
CID000006256 CID000006476 CID000006691 CID000008612 CID000009904
CID000010631 CID000014888 CID000018140 CID000019090 CID000027400
CID000027661 CID000027686 CID000027991 CID000031378 CID000031477
CID000032797 CID000034312 CID000039507 CID000039860 CID000040976
CID000041317 CID000041774 CID000042615 CID000044564 CID000047725
CID000052421 CID000054454 CID000054547 CID000054786 CID000057469
CID000059708 CID000059768 CID000060164 CID000060184 CID000060198
CID000060613 CID000060714 CID000060787 CID000060795 CID000060843
CID000060865 CID000060953 CID000062819 CID000062924 CID000062959
CID000064147 CID000065999 CID000068740 CID000071158 CID000071273
CID000071329 CID000071616 CID000072054 CID000072938 CID000077992
CID000077993 CID000077999 CID000082146 CID000083786 CID000093860
CID000096312 CID000104758 CID000104865 CID000110634 CID000110635
CID000115237 CID000119182 CID000119607 CID000122316 CID000123631
CID000124087 CID000125889 CID000130881 CID000147912 CID000148192
CID000150610 CID000151165 CID000152945 CID000158440 CID000163742
CID000170361 CID000176870 CID000197712 CID000213039 CID000216239
CID000216326 CID000444013 CID000444033 CID000657298 CID001349907
CID003002190 CID003062316 CID003081884 CID004183806 CID004659568
CID004659569 CID005281104 CID005282044 CID005311027 CID005311297
CID005329102 CID005353980 CID005362420 CID005381226 CID005487301
CID005493381 CID005493444 CID006398970 CID006435110 CID006447131
CID009571074

(11) $\mathbb{S}_{11}$: 448 drug compounds having side effect “Abdominal pain”

CID000000158 CID000000159 CID000000206 CID000000214 CID000000444

CID000000450	CID000000564	CID000000596	CID000000598	CID000000738
CID000000767	CID000000853	CID000000942	CID000001003	CID000001065
CID000001125	CID000001134	CID000001546	CID000001690	CID000001935
CID000001971	CID000001972	CID000001978	CID000002022	CID000002082
CID000002083	CID000002092	CID000002118	CID000002153	CID000002156
CID000002162	CID000002170	CID000002177	CID000002179	CID000002182
CID000002187	CID000002216	CID000002232	CID000002250	CID000002265
CID000002267	CID000002269	CID000002284	CID000002311	CID000002349
CID000002375	CID000002405	CID000002431	CID000002462	CID000002471
CID000002478	CID000002487	CID000002512	CID000002524	CID000002541
CID000002550	CID000002554	CID000002578	CID000002585	CID000002609
CID000002610	CID000002617	CID000002622	CID000002631	CID000002637
CID000002646	CID000002650	CID000002654	CID000002656	CID000002658
CID000002662	CID000002666	CID000002675	CID000002676	CID000002678
CID000002708	CID000002751	CID000002762	CID000002764	CID000002769
CID000002771	CID000002786	CID000002801	CID000002802	CID000002803
CID000002806	CID000002895	CID000002909	CID000002951	CID000002955
CID000002958	CID000002973	CID000002978	CID000003007	CID000003015
CID000003016	CID000003032	CID000003040	CID000003042	CID000003043
CID000003062	CID000003066	CID000003080	CID000003108	CID000003121
CID000003125	CID000003143	CID000003148	CID000003152	CID000003154
CID000003157	CID000003161	CID000003203	CID000003222	CID000003255
CID000003261	CID000003279	CID000003285	CID000003291	CID000003308
CID000003310	CID000003324	CID000003333	CID000003339	CID000003340
CID000003342	CID000003345	CID000003348	CID000003355	CID000003366
CID000003379	CID000003386	CID000003394	CID000003403	CID000003404
CID000003406	CID000003410	CID000003414	CID000003417	CID000003419
CID000003440	CID000003446	CID000003449	CID000003454	CID000003475
CID000003476	CID000003478	CID000003488	CID000003510	CID000003519
CID000003648	CID000003661	CID000003672	CID000003685	CID000003698
CID000003702	CID000003706	CID000003736	CID000003746	CID000003749
CID000003750	CID000003793	CID000003823	CID000003825	CID000003826
CID000003827	CID000003869	CID000003877	CID000003878	CID000003883
CID000003899	CID000003902	CID000003911	CID000003929	CID000003937
CID000003948	CID000003954	CID000003956	CID000003957	CID000003961
CID000003962	CID000004030	CID000004036	CID000004044	CID000004046
CID000004075	CID000004091	CID000004095	CID000004158	CID000004163
CID000004170	CID000004171	CID000004173	CID000004178	CID000004189
CID000004195	CID000004196	CID000004200	CID000004205	CID000004212
CID000004236	CID000004253	CID000004259	CID000004264	CID000004409
CID000004419	CID000004421	CID000004428	CID000004449	CID000004451
CID000004463	CID000004485	CID000004493	CID000004509	CID000004510
CID000004513	CID000004539	CID000004542	CID000004583	CID000004585
CID000004594	CID000004595	CID000004599	CID000004603	CID000004607

CID000004609	CID000004614	CID000004634	CID000004635	CID000004666
CID000004679	CID000004691	CID000004739	CID000004740	CID000004745
CID000004775	CID000004819	CID000004828	CID000004845	CID000004856
CID000004873	CID000004885	CID000004889	CID000004891	CID000004893
CID000004913	CID000004915	CID000004920	CID000004932	CID000004946
CID000004991	CID000005002	CID000005005	CID000005029	CID000005035
CID000005038	CID000005040	CID000005064	CID000005070	CID000005071
CID000005073	CID000005076	CID000005077	CID000005078	CID000005090
CID000005095	CID000005152	CID000005155	CID000005195	CID000005203
CID000005210	CID000005212	CID000005215	CID000005245	CID000005253
CID000005291	CID000005344	CID000005372	CID000005376	CID000005379
CID000005394	CID000005396	CID000005401	CID000005402	CID000005404
CID000005408	CID000005426	CID000005430	CID000005453	CID000005466
CID000005479	CID000005496	CID000005508	CID000005512	CID000005514
CID000005515	CID000005523	CID000005525	CID000005530	CID000005538
CID000005544	CID000005591	CID000005625	CID000005645	CID000005647
CID000005650	CID000005656	CID000005665	CID000005672	CID000005717
CID000005718	CID000005719	CID000005721	CID000005726	CID000005731
CID000005732	CID000005734	CID000005735	CID000005978	CID000006058
CID000006476	CID000006691	CID000009034	CID000009433	CID000010100
CID000010631	CID000013342	CID000014888	CID000019090	CID000027661
CID000027991	CID000028112	CID000030623	CID000034312	CID000038904
CID000039042	CID000039860	CID000040159	CID000040976	CID000041317
CID000041744	CID000041774	CID000042615	CID000050294	CID000050614
CID000051577	CID000051634	CID000054454	CID000054547	CID000054688
CID000054786	CID000056959	CID000057469	CID000057537	CID000059708
CID000060184	CID000060198	CID000060612	CID000060613	CID000060754
CID000060787	CID000060795	CID000060843	CID000060852	CID000060865
CID000060871	CID000060877	CID000060953	CID000062816	CID000062819
CID000062924	CID000062959	CID000064147	CID000065027	CID000065999
CID000068740	CID000071158	CID000071273	CID000071301	CID000071329
CID000071616	CID000072054	CID000072938	CID000074989	CID000077992
CID000077999	CID000082146	CID000093860	CID000096312	CID000104741
CID000104758	CID000104865	CID000110634	CID000110635	CID000115237
CID000119182	CID000119607	CID000122316	CID000123620	CID000123631
CID000125889	CID000130881	CID000147912	CID000148192	CID000148211
CID000150310	CID000150311	CID000150610	CID000151165	CID000158440
CID000160051	CID000163742	CID000170361	CID000176168	CID000176870
CID000197712	CID000213039	CID000216239	CID000216326	CID000443871
CID000477468	CID002761171	CID003002190	CID003062316	CID003081884
CID003086672	CID004183806	CID004659568	CID004659569	CID005229711
CID005281104	CID005282044	CID005311297	CID005329102	CID005353980
CID005361912	CID005362070	CID005481350	CID005487301	CID005493381
CID005493444	CID006398970	CID006435110	CID006436173	CID006447131

CID006918453 CID009571074 CID011947681

(12) $\mathbb{S}_{12}$: 447 drug compounds having side effect “Somnolence”

CID000000159 CID000000191 CID000000206 CID000000401 CID000000444
CID000000450 CID000000596 CID000000598 CID000000767 CID000000807
CID000000937 CID000000942 CID000001148 CID000001690 CID000001775
CID000001935 CID000001972 CID000001978 CID000001986 CID000002022
CID000002083 CID000002118 CID000002130 CID000002140 CID000002141
CID000002145 CID000002160 CID000002162 CID000002170 CID000002182
CID000002187 CID000002215 CID000002216 CID000002244 CID000002249
CID000002250 CID000002267 CID000002269 CID000002284 CID000002311
CID000002349 CID000002366 CID000002375 CID000002381 CID000002405
CID000002435 CID000002441 CID000002443 CID000002462 CID000002474
CID000002476 CID000002477 CID000002478 CID000002487 CID000002512
CID000002520 CID000002524 CID000002541 CID000002550 CID000002554
CID000002564 CID000002576 CID000002578 CID000002585 CID000002609
CID000002610 CID000002646 CID000002654 CID000002658 CID000002662
CID000002676 CID000002678 CID000002712 CID000002725 CID000002726
CID000002733 CID000002751 CID000002756 CID000002762 CID000002764
CID000002769 CID000002771 CID000002781 CID000002794 CID000002801
CID000002802 CID000002803 CID000002818 CID000002895 CID000002905
CID000002909 CID000002913 CID000002951 CID000002958 CID000002978
CID000002995 CID000003007 CID000003016 CID000003019 CID000003032
CID000003042 CID000003059 CID000003066 CID000003075 CID000003100
CID000003108 CID000003114 CID000003117 CID000003121 CID000003148
CID000003152 CID000003154 CID000003157 CID000003158 CID000003168
CID000003203 CID000003222 CID000003261 CID000003285 CID000003291
CID000003308 CID000003310 CID000003324 CID000003325 CID000003333
CID000003339 CID000003342 CID000003345 CID000003348 CID000003350
CID000003354 CID000003355 CID000003365 CID000003372 CID000003373
CID000003385 CID000003386 CID000003393 CID000003394 CID000003397
CID000003404 CID000003406 CID000003410 CID000003414 CID000003417
CID000003419 CID000003440 CID000003446 CID000003449 CID000003454
CID000003461 CID000003463 CID000003475 CID000003478 CID000003494
CID000003510 CID000003519 CID000003559 CID000003648 CID000003657
CID000003658 CID000003661 CID000003672 CID000003675 CID000003676
CID000003690 CID000003696 CID000003702 CID000003706 CID000003715
CID000003724 CID000003730 CID000003736 CID000003741 CID000003746
CID000003749 CID000003750 CID000003759 CID000003784 CID000003793
CID000003823 CID000003825 CID000003826 CID000003869 CID000003883
CID000003902 CID000003911 CID000003915 CID000003929 CID000003937
CID000003948 CID000003954 CID000003956 CID000003957 CID000003961
CID000003964 CID000004011 CID000004034 CID000004044 CID000004046
CID000004054 CID000004057 CID000004060 CID000004062 CID000004064

CID000004075	CID000004078	CID000004086	CID000004100	CID000004107
CID000004112	CID000004158	CID000004163	CID000004168	CID000004170
CID000004171	CID000004173	CID000004178	CID000004192	CID000004195
CID000004200	CID000004211	CID000004212	CID000004253	CID000004259
CID000004409	CID000004419	CID000004421	CID000004428	CID000004436
CID000004440	CID000004449	CID000004451	CID000004463	CID000004473
CID000004485	CID000004493	CID000004506	CID000004509	CID000004513
CID000004539	CID000004542	CID000004543	CID000004583	CID000004585
CID000004594	CID000004595	CID000004601	CID000004609	CID000004614
CID000004616	CID000004635	CID000004679	CID000004691	CID000004723
CID000004737	CID000004739	CID000004740	CID000004745	CID000004748
CID000004768	CID000004775	CID000004819	CID000004828	CID000004845
CID000004856	CID000004885	CID000004891	CID000004893	CID000004909
CID000004914	CID000004915	CID000004917	CID000004920	CID000004927
CID000004932	CID000004934	CID000004943	CID000004946	CID000004976
CID000004991	CID000005002	CID000005005	CID000005029	CID000005038
CID000005039	CID000005040	CID000005052	CID000005070	CID000005071
CID000005073	CID000005076	CID000005077	CID000005078	CID000005090
CID000005095	CID000005155	CID000005193	CID000005195	CID000005203
CID000005206	CID000005210	CID000005212	CID000005267	CID000005291
CID000005344	CID000005352	CID000005358	CID000005372	CID000005379
CID000005391	CID000005394	CID000005401	CID000005403	CID000005419
CID000005430	CID000005452	CID000005453	CID000005454	CID000005466
CID000005478	CID000005479	CID000005487	CID000005508	CID000005512
CID000005514	CID000005523	CID000005525	CID000005530	CID000005533
CID000005538	CID000005556	CID000005566	CID000005572	CID000005584
CID000005625	CID000005645	CID000005650	CID000005656	CID000005665
CID000005718	CID000005719	CID000005726	CID000005731	CID000005732
CID000005734	CID000005735	CID000005746	CID000006058	CID000006476
CID000007029	CID000008612	CID000010100	CID000010631	CID000012555
CID000014888	CID000016362	CID000023897	CID000027400	CID000027661
CID000027686	CID000027991	CID000028112	CID000031477	CID000034312
CID000039860	CID000040976	CID000041317	CID000041693	CID000042615
CID000044564	CID000051263	CID000054547	CID000054688	CID000057537
CID000059708	CID000059768	CID000060184	CID000060612	CID000060613
CID000060754	CID000060787	CID000060795	CID000060865	CID000060877
CID000062816	CID000062867	CID000062959	CID000064147	CID000065027
CID000065999	CID000068740	CID000071158	CID000071273	CID000071301
CID000071616	CID000072054	CID000072938	CID000077992	CID000077993
CID000083786	CID000096312	CID000104865	CID000110634	CID000110635
CID000115237	CID000119182	CID000119607	CID000122316	CID000123606
CID000124087	CID000125017	CID000125889	CID000147912	CID000148192
CID000148211	CID000150610	CID000151165	CID000153941	CID000158440
CID000170361	CID000213039	CID000216326	CID000444013	CID000477468

CID000657298 CID001349907 CID002761171 CID003002190 CID003062316
CID003081884 CID004183806 CID004659568 CID004659569 CID005229711
CID005281104 CID005282044 CID005311027 CID005311297 CID005353894
CID005353980 CID005381226 CID005481350 CID006398525 CID006398970
CID006435110 CID009571074

(13) $\mathbb{S}_{13}$: 431 drug compounds having side effect “Pain”

CID000000085 CID000000119 CID000000137 CID000000158 CID000000159
CID000000175 CID000000191 CID000000206 CID000000214 CID000000444
CID000000450 CID000000453 CID000000564 CID000000581 CID000000596
CID000000598 CID000000727 CID000000738 CID000000750 CID000000772
CID000000807 CID000000815 CID000000937 CID000000942 CID000000951
CID000001065 CID000001125 CID000001134 CID000001546 CID000001775
CID000001971 CID000001972 CID000001978 CID000001986 CID000002082
CID000002083 CID000002092 CID000002099 CID000002118 CID000002156
CID000002162 CID000002177 CID000002179 CID000002182 CID000002187
CID000002216 CID000002232 CID000002250 CID000002267 CID000002269
CID000002274 CID000002308 CID000002349 CID000002369 CID000002375
CID000002405 CID000002435 CID000002462 CID000002471 CID000002478
CID000002512 CID000002520 CID000002524 CID000002541 CID000002578
CID000002585 CID000002609 CID000002617 CID000002622 CID000002629
CID000002631 CID000002646 CID000002650 CID000002655 CID000002656
CID000002658 CID000002678 CID000002708 CID000002713 CID000002749
CID000002751 CID000002762 CID000002764 CID000002769 CID000002771
CID000002786 CID000002794 CID000002801 CID000002802 CID000002806
CID000002818 CID000002907 CID000002909 CID000002958 CID000002973
CID000003015 CID000003016 CID000003019 CID000003043 CID000003075
CID000003080 CID000003108 CID000003114 CID000003121 CID000003143
CID000003148 CID000003152 CID000003154 CID000003157 CID000003161
CID000003203 CID000003251 CID000003255 CID000003261 CID000003278
CID000003310 CID000003325 CID000003339 CID000003348 CID000003350
CID000003355 CID000003367 CID000003373 CID000003379 CID000003381
CID000003385 CID000003386 CID000003393 CID000003403 CID000003404
CID000003406 CID000003410 CID000003414 CID000003417 CID000003419
CID000003440 CID000003449 CID000003454 CID000003461 CID000003463
CID000003467 CID000003475 CID000003476 CID000003478 CID000003494
CID000003510 CID000003640 CID000003657 CID000003658 CID000003661
CID000003702 CID000003724 CID000003730 CID000003734 CID000003736
CID000003737 CID000003741 CID000003742 CID000003746 CID000003750
CID000003784 CID000003793 CID000003823 CID000003877 CID000003878
CID000003883 CID000003890 CID000003899 CID000003902 CID000003928
CID000003929 CID000003937 CID000003948 CID000003956 CID000003958
CID000003961 CID000003962 CID000003998 CID000004046 CID000004053
CID000004054 CID000004075 CID000004086 CID000004107 CID000004112

CID000004158	CID000004163	CID000004170	CID000004171	CID000004173
CID000004174	CID000004178	CID000004192	CID000004195	CID000004200
CID000004201	CID000004205	CID000004236	CID000004259	CID000004264
CID000004428	CID000004440	CID000004449	CID000004451	CID000004473
CID000004493	CID000004513	CID000004536	CID000004542	CID000004583
CID000004585	CID000004594	CID000004595	CID000004599	CID000004609
CID000004623	CID000004634	CID000004666	CID000004679	CID000004691
CID000004724	CID000004725	CID000004727	CID000004739	CID000004745
CID000004812	CID000004819	CID000004834	CID000004856	CID000004865
CID000004873	CID000004885	CID000004889	CID000004893	CID000004911
CID000004913	CID000004915	CID000004920	CID000004932	CID000004943
CID000004946	CID000005002	CID000005029	CID000005040	CID000005064
CID000005070	CID000005073	CID000005076	CID000005077	CID000005078
CID000005095	CID000005152	CID000005155	CID000005195	CID000005203
CID000005206	CID000005212	CID000005234	CID000005245	CID000005253
CID000005291	CID000005300	CID000005352	CID000005358	CID000005359
CID000005372	CID000005376	CID000005394	CID000005401	CID000005402
CID000005403	CID000005404	CID000005408	CID000005430	CID000005453
CID000005466	CID000005472	CID000005478	CID000005486	CID000005487
CID000005496	CID000005512	CID000005514	CID000005515	CID000005525
CID000005538	CID000005544	CID000005556	CID000005596	CID000005625
CID000005636	CID000005645	CID000005647	CID000005651	CID000005656
CID000005665	CID000005672	CID000005717	CID000005718	CID000005719
CID000005721	CID000005726	CID000005731	CID000005732	CID000005734
CID000005735	CID000005746	CID000005775	CID000005978	CID000006049
CID000006691	CID000007029	CID000009034	CID000009433	CID000012536
CID000013342	CID000014888	CID000016850	CID000019090	CID000025419
CID000025517	CID000027661	CID000027686	CID000027991	CID000030623
CID000032797	CID000032800	CID000034312	CID000036339	CID000036811
CID000038904	CID000039042	CID000039860	CID000040976	CID000041317
CID000041744	CID000042615	CID000051263	CID000051634	CID000052421
CID000054547	CID000054688	CID000054786	CID000057469	CID000059708
CID000059768	CID000060164	CID000060184	CID000060198	CID000060612
CID000060613	CID000060714	CID000060754	CID000060787	CID000060795
CID000060843	CID000060865	CID000060953	CID000062819	CID000062959
CID000064147	CID000065027	CID000065999	CID000068740	CID000071158
CID000071329	CID000071616	CID000072054	CID000072938	CID000074989
CID000077992	CID000077993	CID000077999	CID000082146	CID000083786
CID000093860	CID000096312	CID000104741	CID000104758	CID000104865
CID000110634	CID000110635	CID000115237	CID000119182	CID000119607
CID000123606	CID000123620	CID000123631	CID000130881	CID000147912
CID000148192	CID000148211	CID000150610	CID000151165	CID000158440
CID000160051	CID000163742	CID000170361	CID000176870	CID000216239
CID000216326	CID000444013	CID000444033	CID000450096	CID000477468

CID003002190 CID003062316 CID003081884 CID003086672 CID004479097
CID004659568 CID004659569 CID005229711 CID005281007 CID005281104
CID005282044 CID005282226 CID005311027 CID005311297 CID005329102
CID005353894 CID005361912 CID005362070 CID005362420 CID005481350
CID005487301 CID005493381 CID005493444 CID006323497 CID006436173
CID009571074

(14) $\mathbb{S}_{14}$: 431 drug compounds having side effect “Constipation”

CID000000085 CID000000143 CID000000159 CID000000271 CID000000444
CID000000450 CID000000596 CID000000598 CID000000738 CID000000807
CID000000942 CID000001206 CID000001546 CID000001690 CID000001775
CID000001935 CID000001972 CID000001978 CID000002022 CID000002083
CID000002092 CID000002099 CID000002118 CID000002130 CID000002156
CID000002160 CID000002162 CID000002170 CID000002182 CID000002187
CID000002215 CID000002216 CID000002249 CID000002250 CID000002267
CID000002269 CID000002284 CID000002311 CID000002315 CID000002344
CID000002369 CID000002375 CID000002381 CID000002405 CID000002443
CID000002476 CID000002477 CID000002478 CID000002487 CID000002512
CID000002520 CID000002524 CID000002541 CID000002550 CID000002554
CID000002564 CID000002578 CID000002609 CID000002622 CID000002654
CID000002662 CID000002676 CID000002678 CID000002712 CID000002720
CID000002725 CID000002726 CID000002732 CID000002751 CID000002762
CID000002764 CID000002769 CID000002771 CID000002781 CID000002786
CID000002794 CID000002800 CID000002801 CID000002802 CID000002803
CID000002806 CID000002818 CID000002895 CID000002907 CID000002909
CID000002913 CID000002949 CID000002951 CID000002958 CID000002995
CID000003007 CID000003015 CID000003016 CID000003019 CID000003032
CID000003042 CID000003059 CID000003075 CID000003100 CID000003114
CID000003121 CID000003143 CID000003148 CID000003151 CID000003152
CID000003157 CID000003158 CID000003203 CID000003222 CID000003249
CID000003261 CID000003285 CID000003308 CID000003310 CID000003324
CID000003325 CID000003333 CID000003339 CID000003340 CID000003342
CID000003345 CID000003355 CID000003365 CID000003367 CID000003372
CID000003379 CID000003386 CID000003393 CID000003394 CID000003403
CID000003404 CID000003410 CID000003414 CID000003417 CID000003419
CID000003440 CID000003446 CID000003449 CID000003454 CID000003461
CID000003463 CID000003475 CID000003478 CID000003494 CID000003510
CID000003519 CID000003559 CID000003637 CID000003639 CID000003647
CID000003648 CID000003657 CID000003661 CID000003672 CID000003675
CID000003690 CID000003696 CID000003702 CID000003715 CID000003736
CID000003746 CID000003749 CID000003750 CID000003759 CID000003784
CID000003793 CID000003825 CID000003826 CID000003869 CID000003878
CID000003883 CID000003899 CID000003902 CID000003911 CID000003929
CID000003937 CID000003948 CID000003954 CID000003958 CID000003961

CID000003962	CID000003964	CID000004011	CID000004032	CID000004036
CID000004044	CID000004054	CID000004057	CID000004058	CID000004075
CID000004078	CID000004091	CID000004095	CID000004121	CID000004138
CID000004158	CID000004163	CID000004170	CID000004171	CID000004173
CID000004178	CID000004200	CID000004205	CID000004212	CID000004236
CID000004253	CID000004259	CID000004409	CID000004411	CID000004428
CID000004440	CID000004449	CID000004473	CID000004485	CID000004493
CID000004506	CID000004509	CID000004513	CID000004539	CID000004542
CID000004543	CID000004547	CID000004583	CID000004585	CID000004594
CID000004595	CID000004601	CID000004609	CID000004614	CID000004635
CID000004666	CID000004679	CID000004691	CID000004736	CID000004737
CID000004739	CID000004740	CID000004745	CID000004748	CID000004771
CID000004775	CID000004819	CID000004828	CID000004856	CID000004870
CID000004885	CID000004889	CID000004893	CID000004915	CID000004917
CID000004919	CID000004920	CID000004932	CID000004934	CID000004946
CID000004976	CID000005002	CID000005005	CID000005029	CID000005038
CID000005039	CID000005040	CID000005064	CID000005070	CID000005071
CID000005073	CID000005076	CID000005077	CID000005078	CID000005090
CID000005095	CID000005152	CID000005155	CID000005193	CID000005195
CID000005203	CID000005210	CID000005212	CID000005245	CID000005291
CID000005352	CID000005358	CID000005372	CID000005376	CID000005379
CID000005394	CID000005401	CID000005426	CID000005452	CID000005454
CID000005466	CID000005479	CID000005487	CID000005508	CID000005512
CID000005514	CID000005515	CID000005516	CID000005523	CID000005525
CID000005530	CID000005533	CID000005538	CID000005556	CID000005566
CID000005572	CID000005584	CID000005596	CID000005625	CID000005645
CID000005647	CID000005650	CID000005656	CID000005665	CID000005672
CID000005718	CID000005719	CID000005726	CID000005731	CID000005732
CID000005734	CID000005735	CID000005978	CID000006058	CID000006476
CID000007029	CID000010100	CID000010631	CID000013342	CID000014888
CID000016362	CID000019090	CID000023897	CID000027661	CID000027686
CID000027991	CID000030623	CID000034312	CID000038904	CID000039042
CID000039860	CID000040976	CID000041317	CID000041781	CID000042615
CID000047725	CID000051634	CID000054454	CID000054547	CID000054688
CID000056959	CID000057537	CID000059708	CID000059768	CID000060184
CID000060198	CID000060613	CID000060753	CID000060787	CID000060795
CID000060843	CID000060852	CID000060953	CID000062816	CID000062819
CID000062867	CID000062959	CID000064147	CID000065027	CID000065999
CID000068740	CID000071158	CID000071273	CID000071616	CID000072054
CID000072938	CID000074989	CID000077992	CID000077993	CID000077999
CID000082146	CID000093860	CID000096312	CID000104741	CID000104758
CID000104865	CID000115237	CID000119182	CID000119607	CID000122316
CID000123631	CID000125017	CID000125889	CID000147912	CID000148192
CID000148211	CID000150610	CID000151165	CID000158440	CID000160051

CID000163742 CID000166548 CID000170361 CID000176168 CID000176870
CID000197712 CID000213039 CID000216239 CID000216326 CID000444013
CID000477468 CID003002190 CID003062316 CID003081884 CID004183806
CID004659568 CID004659569 CID005281104 CID005282044 CID005329102
CID005362070 CID005362420 CID005493444 CID006323497 CID006398525
CID006398970 CID006435110 CID006436173 CID006447131 CID006918453
CID009571074

(15) $\mathbb{S}_{15}$: 409 drug compounds having side effect “Dyspnea”

CID000000085 CID000000158 CID000000159 CID000000191 CID000000444
CID000000564 CID000000581 CID000000596 CID000000598 CID000000681
CID000000772 CID000000807 CID000000853 CID000000937 CID000000942
CID000001003 CID000001065 CID000001546 CID000001690 CID000001775
CID000001935 CID000001971 CID000001972 CID000001978 CID000002022
CID000002083 CID000002130 CID000002140 CID000002141 CID000002156
CID000002162 CID000002179 CID000002182 CID000002187 CID000002215
CID000002216 CID000002232 CID000002249 CID000002250 CID000002267
CID000002269 CID000002274 CID000002284 CID000002311 CID000002349
CID000002369 CID000002375 CID000002405 CID000002435 CID000002462
CID000002476 CID000002478 CID000002487 CID000002512 CID000002519
CID000002520 CID000002541 CID000002550 CID000002554 CID000002578
CID000002583 CID000002585 CID000002609 CID000002622 CID000002637
CID000002654 CID000002662 CID000002676 CID000002678 CID000002708
CID000002726 CID000002751 CID000002764 CID000002771 CID000002801
CID000002802 CID000002803 CID000002806 CID000002818 CID000002891
CID000002895 CID000002907 CID000002909 CID000002958 CID000002973
CID000003007 CID000003015 CID000003016 CID000003019 CID000003032
CID000003042 CID000003059 CID000003066 CID000003075 CID000003108
CID000003114 CID000003121 CID000003143 CID000003148 CID000003152
CID000003154 CID000003156 CID000003157 CID000003203 CID000003222
CID000003261 CID000003285 CID000003308 CID000003310 CID000003333
CID000003339 CID000003340 CID000003342 CID000003345 CID000003348
CID000003350 CID000003355 CID000003366 CID000003367 CID000003372
CID000003373 CID000003379 CID000003385 CID000003386 CID000003393
CID000003394 CID000003403 CID000003404 CID000003410 CID000003414
CID000003419 CID000003446 CID000003454 CID000003461 CID000003475
CID000003476 CID000003478 CID000003488 CID000003510 CID000003518
CID000003519 CID000003559 CID000003636 CID000003637 CID000003648
CID000003657 CID000003672 CID000003676 CID000003702 CID000003706
CID000003715 CID000003724 CID000003734 CID000003736 CID000003737
CID000003741 CID000003742 CID000003746 CID000003749 CID000003750
CID000003779 CID000003784 CID000003793 CID000003825 CID000003826
CID000003869 CID000003878 CID000003883 CID000003890 CID000003899
CID000003902 CID000003911 CID000003914 CID000003915 CID000003929

CID000003937	CID000003948	CID000003961	CID000003962	CID000003964
CID000004044	CID000004046	CID000004053	CID000004054	CID000004075
CID000004091	CID000004112	CID000004140	CID000004158	CID000004163
CID000004168	CID000004171	CID000004178	CID000004192	CID000004195
CID000004200	CID000004205	CID000004212	CID000004236	CID000004253
CID000004259	CID000004409	CID000004419	CID000004425	CID000004428
CID000004440	CID000004449	CID000004451	CID000004473	CID000004485
CID000004493	CID000004497	CID000004506	CID000004509	CID000004510
CID000004539	CID000004542	CID000004583	CID000004585	CID000004594
CID000004609	CID000004614	CID000004635	CID000004679	CID000004691
CID000004724	CID000004736	CID000004739	CID000004740	CID000004745
CID000004812	CID000004819	CID000004828	CID000004829	CID000004856
CID000004885	CID000004889	CID000004893	CID000004917	CID000004920
CID000004932	CID000004943	CID000004946	CID000005002	CID000005005
CID000005029	CID000005038	CID000005040	CID000005052	CID000005064
CID000005070	CID000005071	CID000005073	CID000005076	CID000005077
CID000005078	CID000005090	CID000005095	CID000005195	CID000005203
CID000005206	CID000005210	CID000005212	CID000005234	CID000005245
CID000005253	CID000005291	CID000005352	CID000005358	CID000005372
CID000005376	CID000005379	CID000005391	CID000005394	CID000005396
CID000005401	CID000005403	CID000005408	CID000005426	CID000005454
CID000005466	CID000005478	CID000005487	CID000005496	CID000005514
CID000005515	CID000005516	CID000005523	CID000005525	CID000005538
CID000005544	CID000005566	CID000005625	CID000005645	CID000005647
CID000005650	CID000005651	CID000005656	CID000005672	CID000005718
CID000005719	CID000005721	CID000005726	CID000005731	CID000005732
CID000005734	CID000005735	CID000005746	CID000007029	CID000009034
CID000010631	CID000014888	CID000018140	CID000019090	CID000023897
CID000025419	CID000027661	CID000027686	CID000030623	CID000031477
CID000034312	CID000038904	CID000039860	CID000042113	CID000047319
CID000050294	CID000054454	CID000054547	CID000054688	CID000054786
CID000056959	CID000057469	CID000059768	CID000060184	CID000060198
CID000060612	CID000060613	CID000060714	CID000060754	CID000060787
CID000060795	CID000060843	CID000060953	CID000062924	CID000062959
CID000064147	CID000065027	CID000065999	CID000068740	CID000068844
CID000071158	CID000071273	CID000071301	CID000071329	CID000071616
CID000072938	CID000074989	CID000077992	CID000077993	CID000082146
CID000093860	CID000096312	CID000104741	CID000104865	CID000110634
CID000110635	CID000115237	CID000119182	CID000119607	CID000122316
CID000123606	CID000123631	CID000124087	CID000125889	CID000147912
CID000148192	CID000148211	CID000150610	CID000151165	CID000158440
CID000166548	CID000170361	CID000176870	CID000197712	CID000213039
CID000216239	CID000216326	CID000444013	CID000444033	CID000450096
CID000477468	CID003062316	CID003081884	CID004183806	CID004659568

CID004659569	CID005229711	CID005281104	CID005282044	CID005311027
CID005311181	CID005329102	CID005353980	CID005361912	CID005362420
CID005481350	CID005487301	CID006436173	CID006447131	

(16) $\mathbb{S}_{16}$: 409 drug compounds having side effect “Anorexia”

CID000000085	CID000000143	CID000000159	CID000000271	CID000000444
CID000000596	CID000000598	CID000000767	CID000000937	CID000001046
CID000001065	CID000001148	CID000001546	CID000001690	CID000001775
CID000001935	CID000001971	CID000001972	CID000001978	CID000001986
CID000002022	CID000002083	CID000002118	CID000002130	CID000002145
CID000002156	CID000002160	CID000002162	CID000002170	CID000002179
CID000002182	CID000002187	CID000002249	CID000002250	CID000002269
CID000002284	CID000002315	CID000002344	CID000002369	CID000002375
CID000002443	CID000002462	CID000002476	CID000002477	CID000002478
CID000002487	CID000002512	CID000002524	CID000002550	CID000002554
CID000002559	CID000002564	CID000002575	CID000002578	CID000002585
CID000002609	CID000002617	CID000002654	CID000002658	CID000002662
CID000002676	CID000002678	CID000002708	CID000002719	CID000002720
CID000002725	CID000002727	CID000002732	CID000002751	CID000002762
CID000002764	CID000002771	CID000002781	CID000002794	CID000002801
CID000002802	CID000002803	CID000002818	CID000002895	CID000002907
CID000002909	CID000002913	CID000002951	CID000002958	CID000002983
CID000002995	CID000003007	CID000003009	CID000003015	CID000003016
CID000003019	CID000003032	CID000003042	CID000003043	CID000003059
CID000003062	CID000003066	CID000003075	CID000003100	CID000003114
CID000003121	CID000003143	CID000003148	CID000003152	CID000003154
CID000003157	CID000003158	CID000003203	CID000003222	CID000003249
CID000003255	CID000003278	CID000003279	CID000003291	CID000003308
CID000003310	CID000003324	CID000003325	CID000003339	CID000003342
CID000003345	CID000003355	CID000003365	CID000003366	CID000003367
CID000003372	CID000003385	CID000003386	CID000003393	CID000003394
CID000003397	CID000003403	CID000003404	CID000003405	CID000003414
CID000003417	CID000003440	CID000003446	CID000003449	CID000003454
CID000003461	CID000003476	CID000003478	CID000003559	CID000003637
CID000003639	CID000003647	CID000003648	CID000003652	CID000003657
CID000003685	CID000003690	CID000003696	CID000003698	CID000003702
CID000003706	CID000003715	CID000003734	CID000003750	CID000003767
CID000003793	CID000003821	CID000003825	CID000003826	CID000003877
CID000003878	CID000003883	CID000003899	CID000003902	CID000003911
CID000003929	CID000003937	CID000003948	CID000003956	CID000003961
CID000003962	CID000004032	CID000004033	CID000004036	CID000004046
CID000004053	CID000004054	CID000004075	CID000004078	CID000004091
CID000004095	CID000004100	CID000004112	CID000004121	CID000004158
CID000004170	CID000004173	CID000004200	CID000004205	CID000004211

CID000004212	CID000004236	CID000004253	CID000004259	CID000004409
CID000004411	CID000004428	CID000004449	CID000004451	CID000004493
CID000004509	CID000004513	CID000004539	CID000004542	CID000004543
CID000004583	CID000004585	CID000004594	CID000004609	CID000004614
CID000004634	CID000004635	CID000004645	CID000004666	CID000004679
CID000004723	CID000004727	CID000004739	CID000004740	CID000004745
CID000004748	CID000004819	CID000004845	CID000004856	CID000004870
CID000004885	CID000004889	CID000004891	CID000004909	CID000004911
CID000004913	CID000004915	CID000004920	CID000004932	CID000004946
CID000004976	CID000004993	CID000005002	CID000005005	CID000005029
CID000005038	CID000005040	CID000005052	CID000005064	CID000005070
CID000005071	CID000005073	CID000005076	CID000005077	CID000005078
CID000005095	CID000005155	CID000005195	CID000005203	CID000005210
CID000005212	CID000005215	CID000005291	CID000005344	CID000005352
CID000005372	CID000005376	CID000005379	CID000005391	CID000005394
CID000005396	CID000005401	CID000005402	CID000005412	CID000005426
CID000005430	CID000005452	CID000005453	CID000005454	CID000005466
CID000005472	CID000005478	CID000005479	CID000005496	CID000005514
CID000005515	CID000005516	CID000005523	CID000005525	CID000005530
CID000005533	CID000005538	CID000005556	CID000005566	CID000005584
CID000005591	CID000005625	CID000005645	CID000005647	CID000005650
CID000005656	CID000005672	CID000005718	CID000005719	CID000005721
CID000005726	CID000005731	CID000005732	CID000005734	CID000005735
CID000005746	CID000005978	CID000006049	CID000006058	CID000006476
CID000010631	CID000013342	CID000014888	CID000018140	CID000020585
CID000027661	CID000027991	CID000028112	CID000030623	CID000034312
CID000038904	CID000039860	CID000040976	CID000041317	CID000047725
CID000051634	CID000054454	CID000054547	CID000054688	CID000054786
CID000057537	CID000059708	CID000059768	CID000060184	CID000060198
CID000060613	CID000060754	CID000060787	CID000060795	CID000060843
CID000060953	CID000062816	CID000062867	CID000062959	CID000064147
CID000065027	CID000065999	CID000068740	CID000071158	CID000071616
CID000072938	CID000074989	CID000077992	CID000077993	CID000082146
CID000083786	CID000093860	CID000096312	CID000104741	CID000104758
CID000104865	CID000119182	CID000119607	CID000122316	CID000123620
CID000123631	CID000124087	CID000125889	CID000147912	CID000148192
CID000148211	CID000150610	CID000151165	CID000170361	CID000176870
CID000197712	CID000213039	CID000216239	CID000216326	CID000444013
CID000477468	CID000667490	CID002761171	CID003002190	CID003062316
CID003081884	CID004659568	CID004659569	CID005281007	CID005281104
CID005282044	CID005329102	CID005353980	CID005361912	CID005362070
CID005381226	CID005473385	CID005481350	CID006323497	CID006398970
CID006435110	CID006436173	CID006918453	CID009571074	

(17) $\mathbb{S}_{17}$: 409 drug compounds having side effect “Insomnia”

CID000000085	CID000000159	CID000000450	CID000000596	CID000000598
CID000000738	CID000000807	CID000000853	CID000000937	CID000000942
CID000001134	CID000001206	CID000001546	CID000001690	CID000001775
CID000001935	CID000001971	CID000001972	CID000001978	CID000002021
CID000002022	CID000002083	CID000002118	CID000002130	CID000002153
CID000002156	CID000002160	CID000002162	CID000002170	CID000002171
CID000002182	CID000002187	CID000002215	CID000002216	CID000002249
CID000002250	CID000002269	CID000002274	CID000002284	CID000002311
CID000002369	CID000002375	CID000002405	CID000002435	CID000002443
CID000002462	CID000002476	CID000002478	CID000002487	CID000002512
CID000002520	CID000002524	CID000002550	CID000002554	CID000002564
CID000002576	CID000002578	CID000002583	CID000002585	CID000002609
CID000002646	CID000002654	CID000002662	CID000002676	CID000002678
CID000002725	CID000002726	CID000002751	CID000002762	CID000002764
CID000002769	CID000002781	CID000002800	CID000002801	CID000002802
CID000002803	CID000002806	CID000002818	CID000002895	CID000002909
CID000002913	CID000002951	CID000002958	CID000002995	CID000003003
CID000003007	CID000003016	CID000003032	CID000003042	CID000003059
CID000003066	CID000003075	CID000003100	CID000003114	CID000003121
CID000003125	CID000003143	CID000003151	CID000003152	CID000003154
CID000003157	CID000003161	CID000003203	CID000003222	CID000003285
CID000003292	CID000003308	CID000003325	CID000003333	CID000003339
CID000003340	CID000003342	CID000003345	CID000003348	CID000003355
CID000003365	CID000003373	CID000003379	CID000003385	CID000003394
CID000003403	CID000003404	CID000003410	CID000003414	CID000003417
CID000003419	CID000003446	CID000003449	CID000003454	CID000003461
CID000003475	CID000003476	CID000003478	CID000003494	CID000003510
CID000003512	CID000003519	CID000003559	CID000003640	CID000003648
CID000003661	CID000003672	CID000003675	CID000003696	CID000003702
CID000003715	CID000003724	CID000003736	CID000003746	CID000003750
CID000003779	CID000003784	CID000003793	CID000003821	CID000003825
CID000003826	CID000003827	CID000003877	CID000003878	CID000003883
CID000003899	CID000003902	CID000003911	CID000003929	CID000003937
CID000003948	CID000003956	CID000003958	CID000003961	CID000003962
CID000003964	CID000004011	CID000004036	CID000004044	CID000004046
CID000004054	CID000004057	CID000004060	CID000004075	CID000004086
CID000004095	CID000004107	CID000004114	CID000004158	CID000004163
CID000004168	CID000004170	CID000004171	CID000004173	CID000004192
CID000004195	CID000004196	CID000004201	CID000004236	CID000004253
CID000004259	CID000004409	CID000004428	CID000004451	CID000004473
CID000004485	CID000004493	CID000004513	CID000004536	CID000004539
CID000004542	CID000004543	CID000004583	CID000004585	CID000004594
CID000004603	CID000004609	CID000004614	CID000004635	CID000004638

CID000004679	CID000004723	CID000004724	CID000004736	CID000004739
CID000004740	CID000004745	CID000004748	CID000004771	CID000004819
CID000004828	CID000004845	CID000004856	CID000004865	CID000004885
CID000004889	CID000004893	CID000004894	CID000004915	CID000004917
CID000004920	CID000004927	CID000004932	CID000004934	CID000004943
CID000004946	CID000004976	CID000005002	CID000005005	CID000005029
CID000005035	CID000005038	CID000005039	CID000005040	CID000005064
CID000005070	CID000005071	CID000005073	CID000005076	CID000005077
CID000005078	CID000005090	CID000005095	CID000005152	CID000005155
CID000005203	CID000005206	CID000005210	CID000005212	CID000005215
CID000005245	CID000005253	CID000005291	CID000005344	CID000005352
CID000005372	CID000005376	CID000005379	CID000005394	CID000005401
CID000005408	CID000005419	CID000005426	CID000005454	CID000005466
CID000005478	CID000005479	CID000005514	CID000005523	CID000005525
CID000005530	CID000005533	CID000005538	CID000005544	CID000005566
CID000005584	CID000005625	CID000005636	CID000005645	CID000005647
CID000005650	CID000005665	CID000005717	CID000005718	CID000005726
CID000005731	CID000005734	CID000005978	CID000006476	CID000007029
CID000009433	CID000009904	CID000010631	CID000014888	CID000016362
CID000018140	CID000019090	CID000020585	CID000027661	CID000027686
CID000027991	CID000030623	CID000031378	CID000034312	CID000039042
CID000039860	CID000040976	CID000041317	CID000041781	CID000042615
CID000047725	CID000054454	CID000054547	CID000054688	CID000054786
CID000057469	CID000057537	CID000059708	CID000060198	CID000060613
CID000060753	CID000060787	CID000060795	CID000060852	CID000060877
CID000060953	CID000062816	CID000062819	CID000062867	CID000062924
CID000062959	CID000064147	CID000065027	CID000065999	CID000068740
CID000071158	CID000071273	CID000071301	CID000071329	CID000071616
CID000072054	CID000072938	CID000074989	CID000077992	CID000077993
CID000082146	CID000083786	CID000093860	CID000096312	CID000104741
CID000104865	CID000110634	CID000110635	CID000115237	CID000119607
CID000123606	CID000123620	CID000124087	CID000125889	CID000130881
CID000147912	CID000148192	CID000148211	CID000150610	CID000151165
CID000153941	CID000163742	CID000170361	CID000176870	CID000197712
CID000213039	CID000216326	CID000444013	CID000477468	CID003002190
CID003062316	CID003081884	CID005281104	CID005282044	CID005311027
CID005311181	CID005311297	CID005329102	CID005353980	CID005361912
CID005362070	CID005481350	CID005487301	CID005493381	CID005493444
CID006398525	CID006398970	CID006436173	CID009571074	

(18) $\mathbb{S}_{18}$: 403 drug compounds having side effect “Thrombocytopenia”

CID000000143	CID000000159	CID000000214	CID000000298	CID000000444
CID000000596	CID000000598	CID000000772	CID000001046	CID000001065
CID000001134	CID000001775	CID000001935	CID000001971	CID000001972

CID000002022	CID000002082	CID000002123	CID000002156	CID000002160
CID000002162	CID000002170	CID000002171	CID000002173	CID000002179
CID000002182	CID000002244	CID000002249	CID000002250	CID000002265
CID000002269	CID000002274	CID000002311	CID000002315	CID000002349
CID000002369	CID000002375	CID000002405	CID000002471	CID000002477
CID000002478	CID000002541	CID000002550	CID000002554	CID000002559
CID000002564	CID000002575	CID000002578	CID000002585	CID000002609
CID000002610	CID000002617	CID000002622	CID000002631	CID000002637
CID000002646	CID000002650	CID000002655	CID000002656	CID000002658
CID000002662	CID000002666	CID000002675	CID000002676	CID000002678
CID000002708	CID000002719	CID000002725	CID000002727	CID000002732
CID000002751	CID000002756	CID000002762	CID000002764	CID000002769
CID000002771	CID000002781	CID000002786	CID000002802	CID000002803
CID000002806	CID000002818	CID000002895	CID000002907	CID000002909
CID000002913	CID000002949	CID000002951	CID000002973	CID000002983
CID000002995	CID000003015	CID000003032	CID000003040	CID000003043
CID000003059	CID000003062	CID000003075	CID000003100	CID000003108
CID000003114	CID000003121	CID000003125	CID000003143	CID000003148
CID000003152	CID000003157	CID000003158	CID000003203	CID000003222
CID000003278	CID000003279	CID000003308	CID000003310	CID000003324
CID000003325	CID000003339	CID000003342	CID000003345	CID000003355
CID000003365	CID000003366	CID000003367	CID000003385	CID000003386
CID000003394	CID000003397	CID000003403	CID000003404	CID000003414
CID000003419	CID000003440	CID000003446	CID000003449	CID000003454
CID000003461	CID000003463	CID000003467	CID000003475	CID000003476
CID000003478	CID000003488	CID000003510	CID000003518	CID000003639
CID000003647	CID000003648	CID000003652	CID000003657	CID000003672
CID000003685	CID000003690	CID000003696	CID000003698	CID000003702
CID000003749	CID000003750	CID000003767	CID000003793	CID000003823
CID000003825	CID000003826	CID000003827	CID000003877	CID000003878
CID000003883	CID000003899	CID000003929	CID000003937	CID000003948
CID000003950	CID000003956	CID000003958	CID000003961	CID000003962
CID000003964	CID000004011	CID000004033	CID000004044	CID000004046
CID000004053	CID000004054	CID000004060	CID000004075	CID000004078
CID000004091	CID000004095	CID000004112	CID000004121	CID000004138
CID000004158	CID000004163	CID000004170	CID000004171	CID000004173
CID000004178	CID000004197	CID000004200	CID000004201	CID000004205
CID000004212	CID000004253	CID000004259	CID000004409	CID000004421
CID000004440	CID000004449	CID000004451	CID000004463	CID000004485
CID000004497	CID000004509	CID000004513	CID000004539	CID000004543
CID000004547	CID000004583	CID000004585	CID000004594	CID000004609
CID000004614	CID000004645	CID000004666	CID000004679	CID000004691
CID000004727	CID000004730	CID000004740	CID000004745	CID000004775
CID000004819	CID000004834	CID000004856	CID000004870	CID000004885

CID000004889	CID000004913	CID000004915	CID000004927	CID000004932
CID000004976	CID000004993	CID000005002	CID000005005	CID000005029
CID000005038	CID000005039	CID000005040	CID000005064	CID000005070
CID000005073	CID000005076	CID000005077	CID000005090	CID000005095
CID000005155	CID000005203	CID000005206	CID000005210	CID000005215
CID000005253	CID000005291	CID000005297	CID000005300	CID000005342
CID000005344	CID000005352	CID000005358	CID000005372	CID000005376
CID000005379	CID000005394	CID000005396	CID000005401	CID000005402
CID000005412	CID000005426	CID000005452	CID000005453	CID000005454
CID000005466	CID000005472	CID000005479	CID000005486	CID000005487
CID000005496	CID000005503	CID000005504	CID000005505	CID000005508
CID000005514	CID000005515	CID000005516	CID000005523	CID000005525
CID000005530	CID000005538	CID000005546	CID000005578	CID000005582
CID000005625	CID000005645	CID000005647	CID000005650	CID000005651
CID000005656	CID000005672	CID000005717	CID000005718	CID000005726
CID000005731	CID000005734	CID000005746	CID000005978	CID000010631
CID000013342	CID000014888	CID000018140	CID000020585	CID000027661
CID000027991	CID000030623	CID000034312	CID000036811	CID000038904
CID000039042	CID000041774	CID000042615	CID000047725	CID000050614
CID000051634	CID000054454	CID000054547	CID000054688	CID000054786
CID000057537	CID000059708	CID000060184	CID000060613	CID000060787
CID000060795	CID000060843	CID000060877	CID000060953	CID000062924
CID000062959	CID000064147	CID000065027	CID000065999	CID000068740
CID000071158	CID000071273	CID000071301	CID000071616	CID000072938
CID000074989	CID000082146	CID000083786	CID000093860	CID000096312
CID000104758	CID000104865	CID000115237	CID000119182	CID000119607
CID000123631	CID000125889	CID000130881	CID000147912	CID000150311
CID000150610	CID000151165	CID000163742	CID000166548	CID000170361
CID000176870	CID000216239	CID000216326	CID000444013	CID000477468
CID000657298	CID000667490	CID001349907	CID002761171	CID003062316
CID003081884	CID004183806	CID004659568	CID004659569	CID005281007
CID005282044	CID005329102	CID005353980	CID005361912	CID005381226
CID005473385	CID005481350	CID005493381	CID006323497	CID006398970
CID006918453	CID009571074	CID011954225		

(19) $\mathbb{S}_{19}$: 393 drug compounds having side effect “Paresthesia”

CID000000085	CID000000158	CID000000159	CID000000191	CID000000206
CID000000401	CID000000444	CID000000450	CID000000596	CID000000727
CID000000807	CID000000815	CID000000937	CID000000942	CID000001003
CID000001690	CID000001775	CID000001935	CID000001971	CID000001972
CID000001978	CID000001986	CID000002022	CID000002083	CID000002130
CID000002140	CID000002142	CID000002156	CID000002160	CID000002162
CID000002170	CID000002177	CID000002179	CID000002182	CID000002187
CID000002216	CID000002249	CID000002250	CID000002267	CID000002269

CID000002274	CID000002284	CID000002311	CID000002315	CID000002369
CID000002375	CID000002405	CID000002443	CID000002462	CID000002474
CID000002476	CID000002487	CID000002512	CID000002520	CID000002541
CID000002550	CID000002554	CID000002564	CID000002578	CID000002585
CID000002609	CID000002622	CID000002650	CID000002654	CID000002655
CID000002656	CID000002662	CID000002676	CID000002678	CID000002713
CID000002720	CID000002725	CID000002732	CID000002751	CID000002764
CID000002771	CID000002781	CID000002800	CID000002801	CID000002803
CID000002806	CID000002818	CID000002891	CID000002895	CID000002909
CID000002913	CID000002949	CID000002973	CID000002995	CID000003003
CID000003032	CID000003059	CID000003066	CID000003075	CID000003100
CID000003108	CID000003114	CID000003121	CID000003143	CID000003148
CID000003152	CID000003154	CID000003156	CID000003157	CID000003158
CID000003203	CID000003222	CID000003251	CID000003261	CID000003285
CID000003305	CID000003308	CID000003324	CID000003325	CID000003333
CID000003339	CID000003345	CID000003355	CID000003365	CID000003366
CID000003367	CID000003373	CID000003387	CID000003394	CID000003403
CID000003404	CID000003410	CID000003414	CID000003417	CID000003419
CID000003440	CID000003446	CID000003449	CID000003454	CID000003461
CID000003463	CID000003475	CID000003476	CID000003478	CID000003510
CID000003512	CID000003518	CID000003519	CID000003637	CID000003639
CID000003640	CID000003648	CID000003672	CID000003675	CID000003676
CID000003696	CID000003702	CID000003706	CID000003715	CID000003724
CID000003734	CID000003736	CID000003737	CID000003741	CID000003746
CID000003749	CID000003750	CID000003759	CID000003767	CID000003784
CID000003793	CID000003823	CID000003825	CID000003826	CID000003869
CID000003877	CID000003878	CID000003883	CID000003899	CID000003902
CID000003911	CID000003914	CID000003929	CID000003937	CID000003948
CID000003961	CID000003962	CID000003964	CID000004032	CID000004036
CID000004044	CID000004046	CID000004054	CID000004062	CID000004064
CID000004075	CID000004091	CID000004100	CID000004121	CID000004138
CID000004158	CID000004160	CID000004163	CID000004170	CID000004171
CID000004173	CID000004178	CID000004192	CID000004195	CID000004200
CID000004205	CID000004212	CID000004236	CID000004253	CID000004259
CID000004409	CID000004411	CID000004421	CID000004425	CID000004440
CID000004449	CID000004451	CID000004463	CID000004473	CID000004485
CID000004493	CID000004510	CID000004539	CID000004543	CID000004547
CID000004583	CID000004585	CID000004594	CID000004595	CID000004599
CID000004609	CID000004614	CID000004634	CID000004635	CID000004666
CID000004679	CID000004725	CID000004736	CID000004739	CID000004745
CID000004819	CID000004828	CID000004856	CID000004870	CID000004873
CID000004885	CID000004889	CID000004893	CID000004894	CID000004915
CID000004920	CID000004932	CID000004943	CID000004946	CID000004976
CID000005002	CID000005005	CID000005029	CID000005038	CID000005040

CID000005070	CID000005073	CID000005076	CID000005077	CID000005078
CID000005090	CID000005095	CID000005152	CID000005155	CID000005195
CID000005210	CID000005212	CID000005245	CID000005253	CID000005291
CID000005297	CID000005344	CID000005352	CID000005358	CID000005372
CID000005376	CID000005379	CID000005394	CID000005401	CID000005402
CID000005408	CID000005426	CID000005453	CID000005466	CID000005478
CID000005479	CID000005487	CID000005514	CID000005515	CID000005523
CID000005525	CID000005530	CID000005533	CID000005538	CID000005544
CID000005556	CID000005584	CID000005625	CID000005645	CID000005647
CID000005650	CID000005665	CID000005672	CID000005719	CID000005726
CID000005731	CID000005732	CID000005734	CID000005735	CID000005775
CID000005978	CID000006049	CID000006691	CID000010631	CID000013342
CID000014888	CID000019090	CID000027661	CID000027686	CID000038904
CID000039860	CID000041317	CID000042615	CID000048175	CID000051634
CID000052421	CID000054454	CID000054547	CID000054786	CID000056959
CID000057537	CID000059708	CID000059768	CID000060184	CID000060198
CID000060613	CID000060714	CID000060754	CID000060787	CID000060795
CID000060877	CID000060953	CID000062959	CID000064147	CID000065999
CID000068740	CID000071158	CID000071273	CID000071301	CID000071329
CID000071616	CID000077992	CID000077993	CID000082146	CID000083786
CID000093860	CID000096312	CID000104741	CID000104865	CID000110634
CID000110635	CID000119607	CID000122316	CID000123606	CID000123620
CID000125017	CID000125889	CID000147912	CID000148211	CID000150311
CID000150610	CID000151165	CID000158440	CID000213039	CID000216326
CID000444013	CID000450096	CID000657298	CID001349907	CID003002190
CID003081884	CID004659569	CID005229711	CID005281007	CID005281104
CID005311297	CID005361912	CID005481350		

(20) $\mathbb{S}_{20}$: 385 drug compounds having side effect “Fatigue”

CID000000143	CID000000159	CID000000450	CID000000596	CID000000598
CID000000738	CID000000767	CID000000807	CID000000853	CID000000942
CID000001065	CID000001134	CID000001206	CID000001546	CID000001690
CID000001935	CID000001971	CID000001978	CID000001986	CID000002022
CID000002083	CID000002092	CID000002099	CID000002118	CID000002130
CID000002156	CID000002160	CID000002162	CID000002170	CID000002177
CID000002179	CID000002187	CID000002215	CID000002216	CID000002249
CID000002250	CID000002267	CID000002269	CID000002284	CID000002311
CID000002349	CID000002369	CID000002375	CID000002405	CID000002435
CID000002441	CID000002443	CID000002462	CID000002471	CID000002476
CID000002477	CID000002478	CID000002512	CID000002520	CID000002541
CID000002550	CID000002554	CID000002564	CID000002585	CID000002654
CID000002662	CID000002666	CID000002678	CID000002708	CID000002725
CID000002751	CID000002764	CID000002769	CID000002781	CID000002786
CID000002794	CID000002800	CID000002801	CID000002802	CID000002803

CID000002806	CID000002818	CID000002895	CID000002907	CID000002909
CID000002913	CID000002949	CID000002951	CID000002958	CID000002978
CID000002995	CID000003007	CID000003015	CID000003016	CID000003032
CID000003059	CID000003066	CID000003075	CID000003100	CID000003108
CID000003114	CID000003125	CID000003143	CID000003148	CID000003152
CID000003154	CID000003157	CID000003203	CID000003222	CID000003278
CID000003291	CID000003292	CID000003310	CID000003324	CID000003325
CID000003339	CID000003342	CID000003345	CID000003348	CID000003355
CID000003365	CID000003366	CID000003367	CID000003373	CID000003379
CID000003403	CID000003404	CID000003410	CID000003414	CID000003419
CID000003446	CID000003449	CID000003454	CID000003461	CID000003463
CID000003475	CID000003512	CID000003518	CID000003519	CID000003640
CID000003648	CID000003657	CID000003661	CID000003675	CID000003696
CID000003702	CID000003706	CID000003715	CID000003724	CID000003746
CID000003749	CID000003750	CID000003767	CID000003784	CID000003793
CID000003869	CID000003877	CID000003878	CID000003883	CID000003902
CID000003911	CID000003915	CID000003929	CID000003937	CID000003948
CID000003954	CID000003957	CID000003958	CID000003961	CID000004011
CID000004032	CID000004036	CID000004046	CID000004053	CID000004054
CID000004060	CID000004075	CID000004086	CID000004091	CID000004100
CID000004112	CID000004158	CID000004163	CID000004168	CID000004170
CID000004171	CID000004173	CID000004178	CID000004196	CID000004200
CID000004212	CID000004253	CID000004409	CID000004411	CID000004428
CID000004440	CID000004463	CID000004485	CID000004506	CID000004539
CID000004542	CID000004543	CID000004583	CID000004585	CID000004594
CID000004595	CID000004599	CID000004609	CID000004634	CID000004635
CID000004666	CID000004679	CID000004724	CID000004739	CID000004768
CID000004775	CID000004828	CID000004834	CID000004845	CID000004885
CID000004889	CID000004893	CID000004909	CID000004915	CID000004920
CID000004927	CID000004932	CID000004943	CID000004946	CID000004976
CID000005002	CID000005005	CID000005038	CID000005064	CID000005070
CID000005071	CID000005073	CID000005077	CID000005078	CID000005090
CID000005095	CID000005152	CID000005155	CID000005245	CID000005253
CID000005291	CID000005344	CID000005352	CID000005358	CID000005376
CID000005391	CID000005394	CID000005401	CID000005402	CID000005408
CID000005426	CID000005430	CID000005453	CID000005454	CID000005466
CID000005478	CID000005479	CID000005487	CID000005503	CID000005512
CID000005514	CID000005515	CID000005516	CID000005523	CID000005525
CID000005533	CID000005538	CID000005544	CID000005546	CID000005556
CID000005566	CID000005582	CID000005584	CID000005591	CID000005596
CID000005625	CID000005645	CID000005647	CID000005650	CID000005665
CID000005672	CID000005718	CID000005721	CID000005726	CID000005732
CID000005734	CID000005746	CID000006049	CID000006691	CID000010631
CID000014888	CID000027400	CID000027661	CID000027686	CID000027991

CID000028112	CID000030623	CID000034312	CID000039860	CID000040976
CID000041317	CID000042615	CID000044564	CID000047725	CID000050294
CID000051634	CID000054547	CID000054786	CID000057469	CID000057537
CID000059708	CID000059768	CID000060184	CID000060198	CID000060753
CID000060754	CID000060787	CID000060795	CID000060843	CID000060852
CID000060865	CID000060877	CID000060953	CID000062816	CID000062867
CID000062924	CID000062959	CID000064147	CID000065027	CID000065999
CID000068740	CID000071301	CID000072938	CID000077992	CID000077993
CID000077999	CID000082146	CID000093860	CID000096312	CID000104865
CID000110635	CID000115237	CID000119182	CID000119607	CID000123606
CID000123620	CID000123631	CID000124087	CID000125889	CID000130881
CID000147912	CID000148192	CID000148211	CID000150310	CID000150311
CID000150610	CID000151165	CID000153941	CID000158440	CID000170361
CID000176870	CID000213039	CID000216239	CID000216326	CID000443871
CID000444033	CID000450096	CID000477468	CID003002190	CID003062316
CID004183806	CID004659568	CID004659569	CID005281007	CID005311297
CID005329102	CID005362070	CID005381226	CID005481350	CID005487301
CID005493381	CID005493444	CID006323497	CID006435110	CID006436173

(21) $\mathbb{S}_{21}$: 378 drug compounds having side effect “Hypotension”

CID000000085	CID000000158	CID000000159	CID000000187	CID000000191
CID000000206	CID000000214	CID000000444	CID000000453	CID000000564
CID000000581	CID000000596	CID000000598	CID000000750	CID000000767
CID000000807	CID000000861	CID000000888	CID000000937	CID000001065
CID000001775	CID000001935	CID000001971	CID000001972	CID000001978
CID000002022	CID000002083	CID000002092	CID000002118	CID000002130
CID000002141	CID000002142	CID000002145	CID000002153	CID000002156
CID000002160	CID000002170	CID000002179	CID000002182	CID000002232
CID000002249	CID000002269	CID000002274	CID000002284	CID000002311
CID000002349	CID000002369	CID000002370	CID000002381	CID000002405
CID000002431	CID000002435	CID000002441	CID000002462	CID000002471
CID000002474	CID000002476	CID000002477	CID000002478	CID000002487
CID000002512	CID000002550	CID000002554	CID000002564	CID000002576
CID000002578	CID000002585	CID000002609	CID000002637	CID000002650
CID000002678	CID000002719	CID000002720	CID000002726	CID000002751
CID000002764	CID000002771	CID000002781	CID000002786	CID000002803
CID000002806	CID000002818	CID000002895	CID000002913	CID000002973
CID000002978	CID000002995	CID000003007	CID000003015	CID000003016
CID000003019	CID000003032	CID000003040	CID000003066	CID000003100
CID000003114	CID000003121	CID000003143	CID000003148	CID000003152
CID000003154	CID000003157	CID000003158	CID000003168	CID000003226
CID000003285	CID000003308	CID000003310	CID000003333	CID000003339
CID000003340	CID000003345	CID000003350	CID000003355	CID000003372
CID000003373	CID000003385	CID000003386	CID000003393	CID000003394

CID000003404	CID000003406	CID000003410	CID000003414	CID000003419
CID000003446	CID000003449	CID000003454	CID000003461	CID000003467
CID000003475	CID000003476	CID000003488	CID000003494	CID000003510
CID000003518	CID000003559	CID000003562	CID000003636	CID000003637
CID000003639	CID000003648	CID000003672	CID000003676	CID000003690
CID000003698	CID000003715	CID000003724	CID000003734	CID000003736
CID000003737	CID000003739	CID000003741	CID000003746	CID000003749
CID000003750	CID000003763	CID000003779	CID000003780	CID000003783
CID000003784	CID000003793	CID000003821	CID000003826	CID000003869
CID000003877	CID000003883	CID000003911	CID000003914	CID000003928
CID000003948	CID000003958	CID000003961	CID000003964	CID000004011
CID000004044	CID000004046	CID000004053	CID000004054	CID000004058
CID000004062	CID000004075	CID000004078	CID000004095	CID000004107
CID000004112	CID000004114	CID000004140	CID000004159	CID000004168
CID000004171	CID000004178	CID000004192	CID000004196	CID000004197
CID000004201	CID000004205	CID000004212	CID000004236	CID000004253
CID000004259	CID000004411	CID000004419	CID000004425	CID000004440
CID000004449	CID000004473	CID000004506	CID000004543	CID000004585
CID000004595	CID000004607	CID000004609	CID000004614	CID000004634
CID000004635	CID000004675	CID000004679	CID000004691	CID000004737
CID000004739	CID000004740	CID000004745	CID000004748	CID000004812
CID000004819	CID000004828	CID000004845	CID000004856	CID000004873
CID000004885	CID000004893	CID000004913	CID000004914	CID000004915
CID000004917	CID000004920	CID000004927	CID000004932	CID000004943
CID000004946	CID000004976	CID000005002	CID000005005	CID000005029
CID000005039	CID000005040	CID000005064	CID000005070	CID000005073
CID000005076	CID000005077	CID000005095	CID000005193	CID000005195
CID000005203	CID000005206	CID000005212	CID000005234	CID000005291
CID000005314	CID000005352	CID000005358	CID000005372	CID000005379
CID000005396	CID000005401	CID000005426	CID000005430	CID000005452
CID000005454	CID000005466	CID000005478	CID000005487	CID000005504
CID000005514	CID000005523	CID000005525	CID000005526	CID000005533
CID000005538	CID000005566	CID000005584	CID000005645	CID000005650
CID000005651	CID000005656	CID000005672	CID000005719	CID000005732
CID000005734	CID000005978	CID000006049	CID000006691	CID000008612
CID000009034	CID000009433	CID000014888	CID000016850	CID000020585
CID000023897	CID000027400	CID000027661	CID000028112	CID000032800
CID000034312	CID000036339	CID000036811	CID000038904	CID000039507
CID000039860	CID000041693	CID000041781	CID000047319	CID000047320
CID000047725	CID000051263	CID000054547	CID000054786	CID000056959
CID000057537	CID000059768	CID000060184	CID000060612	CID000060613
CID000060714	CID000060753	CID000060787	CID000060795	CID000060953
CID000062867	CID000062959	CID000064147	CID000065999	CID000068740
CID000071158	CID000071273	CID000071616	CID000074989	CID000077993

CID000093860 CID000096312 CID000104865 CID000110634 CID000110635
CID000115237 CID000119182 CID000119607 CID000123631 CID000125017
CID000125889 CID000145068 CID000147912 CID000148211 CID000150610
CID000151165 CID000158440 CID000163742 CID000166548 CID000170361
CID000176168 CID000216239 CID000216326 CID000315411 CID000444013
CID000477468 CID002761171 CID003002190 CID003062316 CID003081884
CID004183806 CID004659569 CID005281104 CID005282044 CID005282226
CID005311181 CID005487301 CID006435110

(22) $\mathbb{S}_{22}$: 360 drug compounds having side effect "Dyspepsia"

CID000000085 CID000000159 CID000000214 CID000000444 CID000000450
CID000000581 CID000000598 CID000000767 CID000000807 CID000000937
CID000000942 CID000001134 CID000001690 CID000001775 CID000001935
CID000001972 CID000001978 CID000002083 CID000002092 CID000002118
CID000002156 CID000002162 CID000002182 CID000002187 CID000002244
CID000002249 CID000002250 CID000002269 CID000002284 CID000002311
CID000002369 CID000002375 CID000002405 CID000002435 CID000002443
CID000002462 CID000002476 CID000002478 CID000002512 CID000002520
CID000002541 CID000002550 CID000002554 CID000002585 CID000002609
CID000002610 CID000002654 CID000002656 CID000002658 CID000002662
CID000002666 CID000002675 CID000002676 CID000002678 CID000002713
CID000002751 CID000002764 CID000002769 CID000002771 CID000002786
CID000002801 CID000002802 CID000002806 CID000002818 CID000002891
CID000002895 CID000002909 CID000003007 CID000003009 CID000003015
CID000003016 CID000003032 CID000003043 CID000003059 CID000003066
CID000003075 CID000003108 CID000003114 CID000003121 CID000003143
CID000003148 CID000003152 CID000003154 CID000003157 CID000003158
CID000003161 CID000003203 CID000003222 CID000003261 CID000003285
CID000003308 CID000003324 CID000003333 CID000003339 CID000003342
CID000003345 CID000003348 CID000003355 CID000003365 CID000003379
CID000003386 CID000003394 CID000003403 CID000003404 CID000003406
CID000003410 CID000003414 CID000003417 CID000003446 CID000003449
CID000003454 CID000003463 CID000003475 CID000003476 CID000003478
CID000003510 CID000003519 CID000003559 CID000003648 CID000003672
CID000003702 CID000003706 CID000003715 CID000003724 CID000003736
CID000003746 CID000003749 CID000003750 CID000003793 CID000003825
CID000003826 CID000003869 CID000003877 CID000003878 CID000003883
CID000003899 CID000003902 CID000003911 CID000003929 CID000003937
CID000003948 CID000003954 CID000003957 CID000003961 CID000003962
CID000004044 CID000004046 CID000004075 CID000004091 CID000004107
CID000004158 CID000004173 CID000004178 CID000004195 CID000004196
CID000004200 CID000004205 CID000004212 CID000004236 CID000004253
CID000004259 CID000004409 CID000004411 CID000004419 CID000004449
CID000004451 CID000004473 CID000004485 CID000004493 CID000004509

CID000004513	CID000004539	CID000004542	CID000004547	CID000004583
CID000004585	CID000004603	CID000004609	CID000004614	CID000004634
CID000004635	CID000004691	CID000004724	CID000004739	CID000004740
CID000004745	CID000004819	CID000004856	CID000004885	CID000004889
CID000004893	CID000004920	CID000004932	CID000005002	CID000005005
CID000005029	CID000005035	CID000005038	CID000005040	CID000005064
CID000005070	CID000005071	CID000005073	CID000005076	CID000005077
CID000005078	CID000005090	CID000005095	CID000005152	CID000005155
CID000005195	CID000005203	CID000005210	CID000005212	CID000005245
CID000005253	CID000005291	CID000005352	CID000005372	CID000005376
CID000005379	CID000005394	CID000005401	CID000005402	CID000005426
CID000005466	CID000005472	CID000005478	CID000005479	CID000005487
CID000005496	CID000005508	CID000005512	CID000005514	CID000005523
CID000005525	CID000005538	CID000005544	CID000005596	CID000005625
CID000005645	CID000005647	CID000005650	CID000005656	CID000005665
CID000005717	CID000005718	CID000005719	CID000005726	CID000005731
CID000005732	CID000005734	CID000005735	CID000006058	CID000014888
CID000019090	CID000027661	CID000027686	CID000027991	CID000028112
CID000034312	CID000039042	CID000039860	CID000040976	CID000041317
CID000041774	CID000041781	CID000047725	CID000050294	CID000051634
CID000054454	CID000054547	CID000054688	CID000054786	CID000057469
CID000057537	CID000059708	CID000059768	CID000060184	CID000060198
CID000060613	CID000060787	CID000060795	CID000060852	CID000060871
CID000060877	CID000060953	CID000062816	CID000062924	CID000062959
CID000064147	CID000065027	CID000065999	CID000068740	CID000068844
CID000071158	CID000071273	CID000071616	CID000072054	CID000072938
CID000074989	CID000077992	CID000077993	CID000082146	CID000083786
CID000093860	CID000104758	CID000104865	CID000110634	CID000110635
CID000115237	CID000119607	CID000122316	CID000123606	CID000123620
CID000124087	CID000130881	CID000147912	CID000148192	CID000148211
CID000150610	CID000151165	CID000153941	CID000158440	CID000160051
CID000163742	CID000166548	CID000170361	CID000176870	CID000213039
CID000216239	CID000216326	CID000443871	CID000444013	CID000444033
CID000477468	CID003002190	CID003062316	CID003081884	CID004183806
CID004479097	CID004659568	CID004659569	CID005281104	CID005282044
CID005282226	CID005311027	CID005311297	CID005329102	CID005353980
CID005361912	CID005362070	CID005481350	CID005487301	CID005493444
CID006323497	CID006398525	CID006398970	CID006918453	CID009571074

(23) $\mathbb{S}_{23}$: 363 drug compounds having side effect "Tachycardia"

CID000000085	CID000000158	CID000000159	CID000000214	CID000000444
CID000000453	CID000000581	CID000000596	CID000000598	CID000000681
CID000000750	CID000000807	CID000000853	CID000000861	CID000000937
CID000001065	CID000001206	CID000001546	CID000001775	CID000001935

CID000001972	CID000002022	CID000002083	CID000002092	CID000002130
CID000002140	CID000002141	CID000002145	CID000002153	CID000002156
CID000002160	CID000002162	CID000002170	CID000002179	CID000002182
CID000002267	CID000002284	CID000002344	CID000002349	CID000002369
CID000002370	CID000002405	CID000002435	CID000002441	CID000002462
CID000002474	CID000002476	CID000002477	CID000002478	CID000002487
CID000002541	CID000002550	CID000002554	CID000002564	CID000002576
CID000002578	CID000002585	CID000002658	CID000002662	CID000002678
CID000002726	CID000002751	CID000002756	CID000002764	CID000002769
CID000002771	CID000002781	CID000002800	CID000002801	CID000002803
CID000002818	CID000002895	CID000002905	CID000002907	CID000002913
CID000002958	CID000002973	CID000002978	CID000002995	CID000003003
CID000003007	CID000003015	CID000003032	CID000003042	CID000003062
CID000003066	CID000003080	CID000003100	CID000003105	CID000003108
CID000003121	CID000003143	CID000003148	CID000003157	CID000003158
CID000003168	CID000003203	CID000003222	CID000003251	CID000003255
CID000003308	CID000003310	CID000003333	CID000003339	CID000003340
CID000003342	CID000003345	CID000003348	CID000003354	CID000003372
CID000003373	CID000003379	CID000003386	CID000003394	CID000003404
CID000003406	CID000003410	CID000003419	CID000003446	CID000003454
CID000003475	CID000003494	CID000003510	CID000003519	CID000003559
CID000003636	CID000003637	CID000003640	CID000003648	CID000003672
CID000003675	CID000003676	CID000003696	CID000003702	CID000003715
CID000003734	CID000003736	CID000003741	CID000003746	CID000003749
CID000003779	CID000003783	CID000003784	CID000003793	CID000003825
CID000003826	CID000003878	CID000003883	CID000003899	CID000003902
CID000003911	CID000003937	CID000003948	CID000003957	CID000003958
CID000003961	CID000003964	CID000004011	CID000004044	CID000004046
CID000004053	CID000004054	CID000004057	CID000004058	CID000004062
CID000004064	CID000004075	CID000004086	CID000004095	CID000004140
CID000004158	CID000004163	CID000004171	CID000004192	CID000004196
CID000004197	CID000004201	CID000004205	CID000004212	CID000004236
CID000004253	CID000004259	CID000004419	CID000004425	CID000004428
CID000004449	CID000004473	CID000004485	CID000004493	CID000004497
CID000004510	CID000004543	CID000004583	CID000004585	CID000004594
CID000004595	CID000004601	CID000004609	CID000004614	CID000004635
CID000004675	CID000004679	CID000004691	CID000004736	CID000004739
CID000004740	CID000004745	CID000004748	CID000004771	CID000004819
CID000004828	CID000004845	CID000004856	CID000004885	CID000004893
CID000004894	CID000004914	CID000004915	CID000004920	CID000004927
CID000004932	CID000004934	CID000004943	CID000004976	CID000005002
CID000005005	CID000005029	CID000005038	CID000005039	CID000005040
CID000005064	CID000005070	CID000005071	CID000005073	CID000005076
CID000005077	CID000005078	CID000005090	CID000005095	CID000005152

CID000005195	CID000005203	CID000005206	CID000005210	CID000005212
CID000005291	CID000005314	CID000005344	CID000005352	CID000005358
CID000005372	CID000005379	CID000005396	CID000005401	CID000005403
CID000005426	CID000005452	CID000005454	CID000005466	CID000005487
CID000005504	CID000005512	CID000005514	CID000005523	CID000005525
CID000005530	CID000005533	CID000005538	CID000005544	CID000005556
CID000005572	CID000005584	CID000005593	CID000005596	CID000005625
CID000005645	CID000005647	CID000005656	CID000005672	CID000005718
CID000005719	CID000005731	CID000005732	CID000005734	CID000005735
CID000005775	CID000007029	CID000008612	CID000009034	CID000009433
CID000010631	CID000014888	CID000020585	CID000023897	CID000027661
CID000027686	CID000027991	CID000034312	CID000036339	CID000036811
CID000038904	CID000039860	CID000041693	CID000042113	CID000047319
CID000047725	CID000051263	CID000054688	CID000054786	CID000057469
CID000060612	CID000060613	CID000060753	CID000060795	CID000060953
CID000062816	CID000062819	CID000062867	CID000062959	CID000064147
CID000065999	CID000071158	CID000071273	CID000071616	CID000072054
CID000072938	CID000077992	CID000077993	CID000082146	CID000093860
CID000104865	CID000110634	CID000110635	CID000115237	CID000119182
CID000119607	CID000123606	CID000124087	CID000125017	CID000125889
CID000130881	CID000145068	CID000147912	CID000148211	CID000150610
CID000151165	CID000158440	CID000170361	CID000197712	CID000213039
CID000216239	CID000216326	CID000444013	CID000450096	CID000477468
CID003062316	CID003081884	CID004479097	CID004659569	CID005229711
CID005282044	CID005353894	CID011947681		

(24) $\mathbb{S}_{24}$: 361 drug compounds having side effect “Hypersensitivity”

CID000000143	CID000000298	CID000000401	CID000000444	CID000000450
CID000000453	CID000000564	CID000000581	CID000000596	CID000000772
CID000000807	CID000000853	CID000000937	CID000000942	CID000001003
CID000001046	CID000001065	CID000001206	CID000001775	CID000002022
CID000002082	CID000002083	CID000002088	CID000002118	CID000002140
CID000002145	CID000002148	CID000002171	CID000002173	CID000002179
CID000002244	CID000002265	CID000002274	CID000002308	CID000002311
CID000002315	CID000002349	CID000002366	CID000002369	CID000002375
CID000002405	CID000002462	CID000002474	CID000002476	CID000002478
CID000002512	CID000002520	CID000002522	CID000002524	CID000002541
CID000002554	CID000002559	CID000002575	CID000002576	CID000002578
CID000002583	CID000002585	CID000002609	CID000002610	CID000002622
CID000002629	CID000002631	CID000002646	CID000002650	CID000002655
CID000002656	CID000002658	CID000002662	CID000002666	CID000002673
CID000002675	CID000002676	CID000002678	CID000002708	CID000002713
CID000002720	CID000002732	CID000002751	CID000002756	CID000002762
CID000002764	CID000002771	CID000002786	CID000002801	CID000002806

CID000002818	CID000002895	CID000002907	CID000002909	CID000002951
CID000002958	CID000002973	CID000002978	CID000002983	CID000003007
CID000003015	CID000003016	CID000003019	CID000003059	CID000003066
CID000003108	CID000003121	CID000003125	CID000003143	CID000003148
CID000003154	CID000003157	CID000003203	CID000003222	CID000003255
CID000003279	CID000003285	CID000003305	CID000003310	CID000003325
CID000003342	CID000003348	CID000003350	CID000003367	CID000003381
CID000003385	CID000003387	CID000003403	CID000003404	CID000003410
CID000003440	CID000003446	CID000003461	CID000003475	CID000003476
CID000003494	CID000003510	CID000003512	CID000003636	CID000003637
CID000003639	CID000003640	CID000003647	CID000003648	CID000003657
CID000003661	CID000003676	CID000003685	CID000003690	CID000003698
CID000003702	CID000003706	CID000003715	CID000003724	CID000003734
CID000003736	CID000003737	CID000003739	CID000003741	CID000003742
CID000003746	CID000003750	CID000003823	CID000003826	CID000003869
CID000003877	CID000003883	CID000003899	CID000003914	CID000003928
CID000003937	CID000003954	CID000003958	CID000003961	CID000003962
CID000004030	CID000004033	CID000004046	CID000004053	CID000004057
CID000004060	CID000004064	CID000004075	CID000004107	CID000004112
CID000004121	CID000004138	CID000004158	CID000004170	CID000004171
CID000004173	CID000004178	CID000004192	CID000004200	CID000004212
CID000004236	CID000004259	CID000004409	CID000004419	CID000004428
CID000004440	CID000004451	CID000004463	CID000004485	CID000004509
CID000004513	CID000004539	CID000004542	CID000004547	CID000004583
CID000004594	CID000004595	CID000004599	CID000004601	CID000004603
CID000004609	CID000004645	CID000004675	CID000004679	CID000004730
CID000004737	CID000004740	CID000004748	CID000004812	CID000004834
CID000004856	CID000004870	CID000004889	CID000004891	CID000004900
CID000004911	CID000004914	CID000004915	CID000004920	CID000004946
CID000004993	CID000005002	CID000005005	CID000005038	CID000005039
CID000005040	CID000005064	CID000005073	CID000005076	CID000005077
CID000005078	CID000005090	CID000005095	CID000005193	CID000005203
CID000005210	CID000005245	CID000005267	CID000005291	CID000005314
CID000005352	CID000005358	CID000005359	CID000005376	CID000005396
CID000005408	CID000005412	CID000005430	CID000005453	CID000005478
CID000005479	CID000005486	CID000005496	CID000005514	CID000005515
CID000005523	CID000005525	CID000005538	CID000005544	CID000005546
CID000005556	CID000005578	CID000005645	CID000005647	CID000005650
CID000005665	CID000005672	CID000005717	CID000005718	CID000005731
CID000005732	CID000005735	CID000005771	CID000005978	CID000006256
CID000006691	CID000010631	CID000016850	CID000025419	CID000027661
CID000027686	CID000032797	CID000034312	CID000038904	CID000039042
CID000042615	CID000044564	CID000047320	CID000047725	CID000050614
CID000054454	CID000057469	CID000060164	CID000060754	CID000060787

CID000060795	CID000060843	CID000060865	CID000060953	CID000062816
CID000062924	CID000064147	CID000065027	CID000065999	CID000068740
CID000071301	CID000072054	CID000074989	CID000082146	CID000083786
CID000093860	CID000104741	CID000104865	CID000110634	CID000110635
CID000119607	CID000123620	CID000124087	CID000125017	CID000130881
CID000147912	CID000148211	CID000150311	CID000150610	CID000151165
CID000158440	CID000170361	CID000197712	CID000213039	CID000216239
CID000216326	CID000443871	CID000444033	CID000450096	CID000477468
CID002761171	CID003002190	CID003062316	CID003081884	CID004183806
CID005281007	CID005282226	CID005311297	CID005353980	CID005362420
CID005381226	CID005487301	CID005493381	CID006398525	CID006436173
CID006447131				

(25) $\mathbb{S}_{25}$: 363 drug compounds having side effect “Confusion”

CID000000159	CID000000206	CID000000271	CID000000298	CID000000401
CID000000444	CID000000564	CID000000596	CID000000598	CID000000767
CID000000772	CID000000807	CID000000942	CID000001065	CID000001690
CID000001775	CID000001935	CID000001972	CID000001978	CID000001986
CID000002022	CID000002099	CID000002118	CID000002130	CID000002140
CID000002156	CID000002160	CID000002170	CID000002171	CID000002179
CID000002182	CID000002187	CID000002215	CID000002244	CID000002249
CID000002267	CID000002274	CID000002284	CID000002344	CID000002349
CID000002369	CID000002375	CID000002405	CID000002431	CID000002441
CID000002462	CID000002476	CID000002477	CID000002478	CID000002487
CID000002512	CID000002520	CID000002550	CID000002554	CID000002564
CID000002578	CID000002585	CID000002609	CID000002622	CID000002646
CID000002662	CID000002666	CID000002678	CID000002708	CID000002712
CID000002725	CID000002751	CID000002756	CID000002764	CID000002769
CID000002771	CID000002781	CID000002801	CID000002802	CID000002803
CID000002806	CID000002818	CID000002895	CID000002907	CID000002909
CID000002913	CID000002951	CID000002958	CID000002978	CID000002995
CID000003007	CID000003016	CID000003019	CID000003032	CID000003040
CID000003042	CID000003059	CID000003062	CID000003066	CID000003075
CID000003100	CID000003114	CID000003121	CID000003125	CID000003143
CID000003148	CID000003152	CID000003154	CID000003157	CID000003158
CID000003203	CID000003222	CID000003255	CID000003261	CID000003278
CID000003279	CID000003285	CID000003305	CID000003308	CID000003310
CID000003324	CID000003325	CID000003333	CID000003342	CID000003345
CID000003354	CID000003355	CID000003366	CID000003367	CID000003373
CID000003385	CID000003386	CID000003393	CID000003394	CID000003397
CID000003404	CID000003405	CID000003414	CID000003419	CID000003440
CID000003446	CID000003449	CID000003454	CID000003463	CID000003467
CID000003475	CID000003478	CID000003494	CID000003512	CID000003519
CID000003559	CID000003648	CID000003661	CID000003672	CID000003676

CID000003690	CID000003696	CID000003715	CID000003724	CID000003734
CID000003736	CID000003741	CID000003750	CID000003825	CID000003826
CID000003877	CID000003878	CID000003883	CID000003911	CID000003914
CID000003937	CID000003948	CID000003958	CID000003961	CID000003964
CID000004011	CID000004044	CID000004046	CID000004054	CID000004057
CID000004060	CID000004075	CID000004078	CID000004095	CID000004100
CID000004107	CID000004112	CID000004158	CID000004168	CID000004171
CID000004173	CID000004178	CID000004192	CID000004195	CID000004212
CID000004236	CID000004253	CID000004259	CID000004409	CID000004419
CID000004428	CID000004440	CID000004449	CID000004473	CID000004485
CID000004506	CID000004509	CID000004513	CID000004539	CID000004543
CID000004583	CID000004585	CID000004594	CID000004601	CID000004603
CID000004614	CID000004634	CID000004635	CID000004666	CID000004679
CID000004691	CID000004736	CID000004737	CID000004739	CID000004740
CID000004745	CID000004748	CID000004819	CID000004828	CID000004845
CID000004856	CID000004873	CID000004885	CID000004915	CID000004920
CID000004927	CID000004932	CID000004934	CID000004943	CID000004976
CID000004991	CID000005002	CID000005005	CID000005029	CID000005038
CID000005039	CID000005040	CID000005070	CID000005071	CID000005073
CID000005076	CID000005077	CID000005078	CID000005090	CID000005095
CID000005193	CID000005195	CID000005203	CID000005206	CID000005210
CID000005267	CID000005291	CID000005300	CID000005358	CID000005372
CID000005379	CID000005391	CID000005394	CID000005396	CID000005401
CID000005402	CID000005408	CID000005426	CID000005430	CID000005452
CID000005453	CID000005466	CID000005478	CID000005479	CID000005487
CID000005496	CID000005512	CID000005514	CID000005523	CID000005530
CID000005533	CID000005538	CID000005556	CID000005572	CID000005582
CID000005584	CID000005625	CID000005645	CID000005647	CID000005656
CID000005665	CID000005718	CID000005719	CID000005721	CID000005726
CID000005731	CID000005732	CID000005734	CID000005735	CID000005746
CID000006058	CID000006476	CID000010631	CID000014888	CID000018140
CID000019090	CID000025419	CID000027400	CID000027661	CID000028112
CID000034312	CID000039860	CID000041693	CID000051263	CID000054547
CID000054688	CID000057537	CID000059708	CID000059768	CID000060184
CID000060198	CID000060612	CID000060613	CID000060753	CID000060787
CID000060795	CID000060953	CID000062959	CID000064147	CID000068740
CID000071158	CID000071273	CID000071616	CID000072054	CID000077992
CID000077993	CID000082146	CID000083786	CID000093860	CID000096312
CID000115237	CID000119607	CID000122316	CID000125889	CID000147912
CID000148192	CID000150610	CID000151165	CID000158440	CID000163742
CID000213039	CID000216326	CID000444013	CID003062316	CID003081884
CID004183806	CID004659568	CID004659569	CID005281007	CID005281104
CID005282044	CID005361912	CID005381226		

(26) $\mathbb{S}_{26}$: 360 drug compounds having side effect "Leukopenia"

CID000000143	CID000000298	CID000000444	CID000000564	CID000000596
CID000000598	CID000000937	CID000001065	CID000001775	CID000001935
CID000001971	CID000001972	CID000001986	CID000002022	CID000002082
CID000002123	CID000002130	CID000002140	CID000002145	CID000002160
CID000002162	CID000002170	CID000002171	CID000002173	CID000002179
CID000002187	CID000002244	CID000002265	CID000002269	CID000002311
CID000002315	CID000002349	CID000002477	CID000002478	CID000002541
CID000002554	CID000002559	CID000002575	CID000002576	CID000002578
CID000002585	CID000002609	CID000002617	CID000002622	CID000002631
CID000002637	CID000002650	CID000002655	CID000002656	CID000002658
CID000002662	CID000002666	CID000002673	CID000002675	CID000002676
CID000002708	CID000002720	CID000002726	CID000002727	CID000002732
CID000002751	CID000002764	CID000002769	CID000002771	CID000002786
CID000002802	CID000002806	CID000002818	CID000002895	CID000002907
CID000002909	CID000002913	CID000002949	CID000002951	CID000002973
CID000003015	CID000003016	CID000003019	CID000003032	CID000003040
CID000003043	CID000003075	CID000003121	CID000003143	CID000003156
CID000003157	CID000003158	CID000003161	CID000003222	CID000003261
CID000003279	CID000003291	CID000003305	CID000003308	CID000003310
CID000003324	CID000003325	CID000003339	CID000003345	CID000003354
CID000003355	CID000003365	CID000003366	CID000003367	CID000003372
CID000003385	CID000003386	CID000003393	CID000003394	CID000003397
CID000003403	CID000003404	CID000003414	CID000003419	CID000003440
CID000003446	CID000003454	CID000003461	CID000003463	CID000003467
CID000003475	CID000003476	CID000003478	CID000003488	CID000003510
CID000003512	CID000003518	CID000003559	CID000003637	CID000003639
CID000003647	CID000003648	CID000003652	CID000003657	CID000003672
CID000003675	CID000003685	CID000003690	CID000003702	CID000003715
CID000003750	CID000003784	CID000003793	CID000003823	CID000003826
CID000003878	CID000003883	CID000003899	CID000003902	CID000003928
CID000003929	CID000003937	CID000003948	CID000003950	CID000003956
CID000003961	CID000003962	CID000003964	CID000004033	CID000004036
CID000004044	CID000004046	CID000004053	CID000004054	CID000004060
CID000004064	CID000004075	CID000004078	CID000004107	CID000004112
CID000004121	CID000004138	CID000004158	CID000004168	CID000004170
CID000004171	CID000004173	CID000004178	CID000004200	CID000004201
CID000004205	CID000004212	CID000004253	CID000004259	CID000004409
CID000004421	CID000004449	CID000004451	CID000004485	CID000004493
CID000004506	CID000004509	CID000004539	CID000004547	CID000004583
CID000004585	CID000004594	CID000004609	CID000004614	CID000004616
CID000004635	CID000004666	CID000004679	CID000004691	CID000004727
CID000004730	CID000004739	CID000004740	CID000004745	CID000004748
CID000004775	CID000004819	CID000004834	CID000004856	CID000004870

CID000004885	CID000004889	CID000004908	CID000004911	CID000004915
CID000004917	CID000004927	CID000004932	CID000004976	CID000004993
CID000005002	CID000005029	CID000005038	CID000005039	CID000005040
CID000005064	CID000005070	CID000005073	CID000005076	CID000005090
CID000005095	CID000005155	CID000005195	CID000005203	CID000005210
CID000005212	CID000005215	CID000005253	CID000005291	CID000005297
CID000005300	CID000005342	CID000005344	CID000005352	CID000005372
CID000005376	CID000005394	CID000005396	CID000005408	CID000005426
CID000005430	CID000005452	CID000005453	CID000005454	CID000005466
CID000005479	CID000005487	CID000005496	CID000005503	CID000005504
CID000005505	CID000005514	CID000005515	CID000005516	CID000005525
CID000005530	CID000005566	CID000005578	CID000005625	CID000005645
CID000005647	CID000005656	CID000005672	CID000005718	CID000005726
CID000005731	CID000005732	CID000005734	CID000005746	CID000005978
CID000006058	CID000006476	CID000007029	CID000013342	CID000016362
CID000018140	CID000019090	CID000020585	CID000023897	CID000027661
CID000030623	CID000034312	CID000038904	CID000039860	CID000042615
CID000047725	CID000050294	CID000050614	CID000051634	CID000054454
CID000054547	CID000054688	CID000059708	CID000060184	CID000060613
CID000060787	CID000060795	CID000060843	CID000060953	CID000062867
CID000062924	CID000062959	CID000064147	CID000068740	CID000071158
CID000071616	CID000072938	CID000074989	CID000077993	CID000082146
CID000083786	CID000093860	CID000096312	CID000104741	CID000104758
CID000119182	CID000119607	CID000122316	CID000125889	CID000130881
CID000147912	CID000153941	CID000166548	CID000216239	CID000216326
CID000477468	CID000657298	CID000667490	CID001349907	CID003002190
CID003081884	CID004659568	CID005281007	CID005281104	CID005282044
CID005329102	CID005353980	CID005361912	CID005381226	CID006323497
CID006398970	CID006435110	CID006918453	CID009571074	CID011954225

(27) $\mathbb{S}_{27}$: 354 drug compounds having side effect "Dry mouth"

CID000000159	CID000000191	CID000000214	CID000000444	CID000000738
CID000000767	CID000000807	CID000000937	CID000000942	CID000001148
CID000001690	CID000001775	CID000001972	CID000002083	CID000002099
CID000002130	CID000002156	CID000002160	CID000002162	CID000002170
CID000002182	CID000002187	CID000002216	CID000002249	CID000002250
CID000002267	CID000002269	CID000002284	CID000002344	CID000002369
CID000002375	CID000002381	CID000002405	CID000002435	CID000002441
CID000002443	CID000002462	CID000002471	CID000002476	CID000002478
CID000002487	CID000002512	CID000002520	CID000002524	CID000002541
CID000002550	CID000002554	CID000002559	CID000002575	CID000002585
CID000002654	CID000002662	CID000002678	CID000002713	CID000002726
CID000002751	CID000002764	CID000002769	CID000002771	CID000002801
CID000002802	CID000002803	CID000002818	CID000002895	CID000002909

CID000002958	CID000002978	CID000002995	CID000003007	CID000003016
CID000003019	CID000003032	CID000003042	CID000003043	CID000003066
CID000003075	CID000003108	CID000003114	CID000003121	CID000003125
CID000003143	CID000003148	CID000003151	CID000003152	CID000003154
CID000003157	CID000003158	CID000003161	CID000003203	CID000003222
CID000003261	CID000003308	CID000003324	CID000003325	CID000003333
CID000003339	CID000003342	CID000003345	CID000003348	CID000003354
CID000003355	CID000003365	CID000003366	CID000003372	CID000003373
CID000003386	CID000003393	CID000003394	CID000003404	CID000003410
CID000003414	CID000003417	CID000003419	CID000003446	CID000003449
CID000003454	CID000003475	CID000003476	CID000003494	CID000003510
CID000003518	CID000003519	CID000003559	CID000003636	CID000003648
CID000003658	CID000003672	CID000003675	CID000003696	CID000003702
CID000003724	CID000003736	CID000003737	CID000003741	CID000003742
CID000003746	CID000003759	CID000003784	CID000003825	CID000003826
CID000003827	CID000003869	CID000003877	CID000003878	CID000003883
CID000003899	CID000003902	CID000003911	CID000003915	CID000003929
CID000003937	CID000003948	CID000003954	CID000003957	CID000003961
CID000003962	CID000003964	CID000004011	CID000004034	CID000004044
CID000004057	CID000004058	CID000004075	CID000004078	CID000004086
CID000004095	CID000004158	CID000004170	CID000004171	CID000004173
CID000004178	CID000004195	CID000004200	CID000004205	CID000004236
CID000004253	CID000004259	CID000004264	CID000004409	CID000004411
CID000004419	CID000004428	CID000004440	CID000004449	CID000004473
CID000004485	CID000004493	CID000004513	CID000004539	CID000004543
CID000004583	CID000004585	CID000004594	CID000004595	CID000004609
CID000004614	CID000004635	CID000004679	CID000004691	CID000004736
CID000004740	CID000004745	CID000004748	CID000004828	CID000004845
CID000004856	CID000004885	CID000004893	CID000004915	CID000004919
CID000004920	CID000004927	CID000004932	CID000004943	CID000004976
CID000005002	CID000005005	CID000005029	CID000005038	CID000005064
CID000005070	CID000005071	CID000005073	CID000005076	CID000005077
CID000005078	CID000005090	CID000005095	CID000005152	CID000005155
CID000005195	CID000005203	CID000005206	CID000005210	CID000005212
CID000005245	CID000005358	CID000005372	CID000005376	CID000005391
CID000005394	CID000005401	CID000005402	CID000005403	CID000005408
CID000005426	CID000005454	CID000005466	CID000005478	CID000005487
CID000005512	CID000005514	CID000005523	CID000005525	CID000005530
CID000005533	CID000005538	CID000005544	CID000005546	CID000005556
CID000005566	CID000005584	CID000005596	CID000005625	CID000005645
CID000005647	CID000005650	CID000005656	CID000005718	CID000005719
CID000005731	CID000005732	CID000005734	CID000005735	CID000007029
CID000014888	CID000016362	CID000019090	CID000023897	CID000027400
CID000027661	CID000027991	CID000028112	CID000034312	CID000039860

CID000041317	CID000042615	CID000047725	CID000051634	CID000054547
CID000054688	CID000056959	CID000057537	CID000059768	CID000060184
CID000060612	CID000060613	CID000060714	CID000060754	CID000060787
CID000060795	CID000060865	CID000060953	CID000062867	CID000062924
CID000062959	CID000064147	CID000065999	CID000068740	CID000068844
CID000071158	CID000071616	CID000072054	CID000072938	CID000077992
CID000077993	CID000082146	CID000104865	CID000110634	CID000110635
CID000115237	CID000119607	CID000122316	CID000123606	CID000123620
CID000123631	CID000124087	CID000125017	CID000125889	CID000147912
CID000148192	CID000148211	CID000150610	CID000151165	CID000158440
CID000170361	CID000213039	CID000216239	CID000216326	CID000444013
CID000444033	CID000450096	CID003002190	CID003081884	CID004659568
CID004659569	CID005281104	CID005282044	CID005311027	CID005329102
CID005362070	CID006398525	CID006398970	CID009571074	

(28) $\mathbb{S}_{28}$: 341 drug compounds having side effect “Cough”

CID000000085	CID000000158	CID000000159	CID000000191	CID000000214
CID000000444	CID000000450	CID000000581	CID000000596	CID000000598
CID000000807	CID000000937	CID000000942	CID000001125	CID000001134
CID000001546	CID000001690	CID000001775	CID000001935	CID000001971
CID000001972	CID000001978	CID000002083	CID000002140	CID000002156
CID000002162	CID000002182	CID000002187	CID000002232	CID000002249
CID000002267	CID000002269	CID000002308	CID000002311	CID000002369
CID000002375	CID000002405	CID000002435	CID000002462	CID000002476
CID000002478	CID000002487	CID000002541	CID000002550	CID000002578
CID000002585	CID000002609	CID000002658	CID000002662	CID000002676
CID000002678	CID000002708	CID000002713	CID000002726	CID000002751
CID000002764	CID000002769	CID000002771	CID000002786	CID000002801
CID000002802	CID000002806	CID000002818	CID000002907	CID000002909
CID000002955	CID000002958	CID000002978	CID000003015	CID000003016
CID000003019	CID000003032	CID000003075	CID000003108	CID000003121
CID000003143	CID000003148	CID000003152	CID000003154	CID000003156
CID000003157	CID000003161	CID000003203	CID000003222	CID000003241
CID000003261	CID000003285	CID000003310	CID000003325	CID000003333
CID000003339	CID000003345	CID000003348	CID000003355	CID000003367
CID000003373	CID000003379	CID000003381	CID000003386	CID000003394
CID000003403	CID000003404	CID000003410	CID000003414	CID000003419
CID000003446	CID000003449	CID000003454	CID000003461	CID000003463
CID000003475	CID000003510	CID000003648	CID000003702	CID000003706
CID000003734	CID000003736	CID000003737	CID000003741	CID000003742
CID000003746	CID000003749	CID000003750	CID000003763	CID000003784
CID000003793	CID000003826	CID000003877	CID000003878	CID000003883
CID000003899	CID000003902	CID000003911	CID000003915	CID000003929
CID000003937	CID000003948	CID000003957	CID000004053	CID000004054

CID000004075	CID000004086	CID000004112	CID000004158	CID000004170
CID000004192	CID000004200	CID000004201	CID000004205	CID000004212
CID000004236	CID000004253	CID000004259	CID000004264	CID000004409
CID000004411	CID000004428	CID000004440	CID000004449	CID000004485
CID000004493	CID000004509	CID000004513	CID000004542	CID000004583
CID000004585	CID000004594	CID000004599	CID000004609	CID000004634
CID000004635	CID000004666	CID000004679	CID000004691	CID000004724
CID000004739	CID000004745	CID000004748	CID000004819	CID000004845
CID000004885	CID000004889	CID000004893	CID000004915	CID000004917
CID000004920	CID000004932	CID000004943	CID000005002	CID000005005
CID000005035	CID000005038	CID000005040	CID000005064	CID000005070
CID000005071	CID000005073	CID000005076	CID000005077	CID000005078
CID000005090	CID000005095	CID000005152	CID000005155	CID000005195
CID000005203	CID000005206	CID000005210	CID000005212	CID000005245
CID000005253	CID000005291	CID000005344	CID000005358	CID000005372
CID000005376	CID000005394	CID000005401	CID000005402	CID000005426
CID000005454	CID000005466	CID000005478	CID000005496	CID000005514
CID000005515	CID000005523	CID000005525	CID000005538	CID000005544
CID000005566	CID000005625	CID000005636	CID000005645	CID000005650
CID000005656	CID000005665	CID000005672	CID000005718	CID000005726
CID000005731	CID000005732	CID000005734	CID000005746	CID000012536
CID000012555	CID000014888	CID000019090	CID000027661	CID000027686
CID000027991	CID000030623	CID000031477	CID000034312	CID000039860
CID000040976	CID000041317	CID000041781	CID000042113	CID000047725
CID000050294	CID000054547	CID000054688	CID000054786	CID000057469
CID000057537	CID000059708	CID000060184	CID000060198	CID000060613
CID000060714	CID000060787	CID000060795	CID000060865	CID000060871
CID000060877	CID000060953	CID000062819	CID000062924	CID000062959
CID000064147	CID000065027	CID000065999	CID000068740	CID000071158
CID000071273	CID000071329	CID000071616	CID000072054	CID000074989
CID000077993	CID000077999	CID000082146	CID000093860	CID000096312
CID000104741	CID000104758	CID000104865	CID000115237	CID000119182
CID000119607	CID000122316	CID000123620	CID000123631	CID000125889
CID000130881	CID000147912	CID000148192	CID000150310	CID000150311
CID000150610	CID000151165	CID000158440	CID000160051	CID000163742
CID000166548	CID000170361	CID000176870	CID000197712	CID000213039
CID000216239	CID000216326	CID000443871	CID000444013	CID000444033
CID000477468	CID003062316	CID003081884	CID004659569	CID005229711
CID005281104	CID005282044	CID005311027	CID005311181	CID005329102
CID005353980	CID005362070	CID005362420	CID005493381	CID005493444
CID006447131				

(29) $\mathbb{S}_{29}$: 347 drug compounds having side effect “Asthenia”

CID000000085	CID000000159	CID000000214	CID000000444	CID000000450

CID000000596	CID000000598	CID000000937	CID000001065	CID000001546
CID000001690	CID000001775	CID000001935	CID000001972	CID000002022
CID000002083	CID000002088	CID000002092	CID000002118	CID000002156
CID000002162	CID000002179	CID000002182	CID000002187	CID000002216
CID000002250	CID000002269	CID000002284	CID000002311	CID000002349
CID000002369	CID000002375	CID000002405	CID000002435	CID000002443
CID000002462	CID000002476	CID000002478	CID000002487	CID000002512
CID000002520	CID000002541	CID000002550	CID000002554	CID000002578
CID000002583	CID000002585	CID000002609	CID000002662	CID000002676
CID000002678	CID000002751	CID000002764	CID000002771	CID000002801
CID000002802	CID000002803	CID000002806	CID000002891	CID000002895
CID000002907	CID000002909	CID000002978	CID000003007	CID000003009
CID000003015	CID000003016	CID000003032	CID000003043	CID000003059
CID000003066	CID000003075	CID000003108	CID000003121	CID000003143
CID000003151	CID000003152	CID000003154	CID000003157	CID000003203
CID000003219	CID000003222	CID000003261	CID000003285	CID000003308
CID000003310	CID000003325	CID000003333	CID000003339	CID000003342
CID000003345	CID000003350	CID000003355	CID000003365	CID000003373
CID000003386	CID000003394	CID000003403	CID000003404	CID000003410
CID000003414	CID000003417	CID000003446	CID000003449	CID000003454
CID000003461	CID000003475	CID000003476	CID000003478	CID000003488
CID000003510	CID000003518	CID000003519	CID000003639	CID000003648
CID000003652	CID000003657	CID000003672	CID000003676	CID000003690
CID000003702	CID000003706	CID000003736	CID000003749	CID000003750
CID000003793	CID000003826	CID000003869	CID000003878	CID000003883
CID000003899	CID000003902	CID000003911	CID000003914	CID000003929
CID000003937	CID000003948	CID000003958	CID000003961	CID000003962
CID000004033	CID000004044	CID000004046	CID000004054	CID000004075
CID000004091	CID000004095	CID000004138	CID000004158	CID000004168
CID000004170	CID000004195	CID000004196	CID000004201	CID000004205
CID000004212	CID000004236	CID000004253	CID000004259	CID000004409
CID000004428	CID000004449	CID000004451	CID000004473	CID000004485
CID000004493	CID000004509	CID000004510	CID000004513	CID000004539
CID000004542	CID000004583	CID000004585	CID000004594	CID000004614
CID000004635	CID000004666	CID000004679	CID000004691	CID000004724
CID000004739	CID000004745	CID000004819	CID000004856	CID000004885
CID000004889	CID000004891	CID000004893	CID000004920	CID000004927
CID000004943	CID000004946	CID000005002	CID000005005	CID000005029
CID000005038	CID000005040	CID000005064	CID000005070	CID000005071
CID000005073	CID000005076	CID000005077	CID000005078	CID000005090
CID000005095	CID000005155	CID000005195	CID000005203	CID000005206
CID000005210	CID000005212	CID000005245	CID000005253	CID000005291
CID000005344	CID000005372	CID000005376	CID000005379	CID000005394
CID000005396	CID000005401	CID000005408	CID000005426	CID000005466

CID000005472 CID000005478 CID000005487 CID000005496 CID000005508
CID000005514 CID000005515 CID000005516 CID000005523 CID000005525
CID000005538 CID000005544 CID000005625 CID000005645 CID000005647
CID000005650 CID000005656 CID000005665 CID000005672 CID000005717
CID000005718 CID000005719 CID000005726 CID000005731 CID000005732
CID000005734 CID000005735 CID000005746 CID000006691 CID000010631
CID000019090 CID000027661 CID000027991 CID000031477 CID000034312
CID000038904 CID000039860 CID000040976 CID000041744 CID000041781
CID000042615 CID000051634 CID000054454 CID000054547 CID000054688
CID000054786 CID000056959 CID000057537 CID000059708 CID000059768
CID000060184 CID000060198 CID000060613 CID000060753 CID000060754
CID000060787 CID000060795 CID000060852 CID000060865 CID000060871
CID000060877 CID000060953 CID000062959 CID000064147 CID000065027
CID000065999 CID000068740 CID000071158 CID000071273 CID000071301
CID000071329 CID000071616 CID000072054 CID000072938 CID000074989
CID000077992 CID000077993 CID000082146 CID000083786 CID000093860
CID000096312 CID000104741 CID000104758 CID000110634 CID000110635
CID000115237 CID000119607 CID000122316 CID000123606 CID000123631
CID000125017 CID000125889 CID000130881 CID000147912 CID000148192
CID000150610 CID000151165 CID000160051 CID000170361 CID000213039
CID000216239 CID000216326 CID000443871 CID000444013 CID003002190
CID003062316 CID003081884 CID004183806 CID004659568 CID004659569
CID005281104 CID005282044 CID005311027 CID005311297 CID005329102
CID005361912 CID005362420 CID005481350 CID005493444 CID006398970
CID006435110 CID009571074

(30) $\mathbb{S}_{30}$: 344 drug compounds having side effect “Arthralgia”

CID000000158 CID000000159 CID000000444 CID000000450 CID000000596
CID000000598 CID000000738 CID000000767 CID000000853 CID000000937
CID000000942 CID000001046 CID000001065 CID000001125 CID000001134
CID000001546 CID000001690 CID000001775 CID000001935 CID000001971
CID000001972 CID000001978 CID000002022 CID000002092 CID000002118
CID000002140 CID000002142 CID000002162 CID000002182 CID000002187
CID000002215 CID000002250 CID000002265 CID000002269 CID000002311
CID000002349 CID000002369 CID000002405 CID000002435 CID000002462
CID000002477 CID000002478 CID000002512 CID000002520 CID000002541
CID000002550 CID000002554 CID000002585 CID000002609 CID000002610
CID000002658 CID000002662 CID000002666 CID000002673 CID000002676
CID000002678 CID000002713 CID000002751 CID000002756 CID000002764
CID000002769 CID000002771 CID000002786 CID000002800 CID000002801
CID000002802 CID000002803 CID000002806 CID000002818 CID000002909
CID000002949 CID000002958 CID000002973 CID000003032 CID000003040
CID000003043 CID000003066 CID000003075 CID000003108 CID000003121
CID000003143 CID000003148 CID000003152 CID000003154 CID000003157

CID000003161	CID000003203	CID000003222	CID000003261	CID000003279
CID000003285	CID000003305	CID000003325	CID000003333	CID000003339
CID000003345	CID000003355	CID000003367	CID000003381	CID000003386
CID000003393	CID000003403	CID000003404	CID000003410	CID000003414
CID000003419	CID000003446	CID000003449	CID000003454	CID000003461
CID000003463	CID000003467	CID000003475	CID000003478	CID000003488
CID000003519	CID000003637	CID000003648	CID000003702	CID000003706
CID000003736	CID000003784	CID000003793	CID000003877	CID000003878
CID000003883	CID000003899	CID000003902	CID000003911	CID000003937
CID000003948	CID000003961	CID000003962	CID000004046	CID000004054
CID000004075	CID000004112	CID000004138	CID000004158	CID000004159
CID000004163	CID000004170	CID000004171	CID000004173	CID000004178
CID000004200	CID000004205	CID000004212	CID000004236	CID000004253
CID000004259	CID000004421	CID000004428	CID000004440	CID000004449
CID000004451	CID000004463	CID000004473	CID000004485	CID000004509
CID000004539	CID000004547	CID000004583	CID000004585	CID000004594
CID000004607	CID000004609	CID000004634	CID000004635	CID000004666
CID000004679	CID000004691	CID000004727	CID000004739	CID000004745
CID000004819	CID000004828	CID000004885	CID000004889	CID000004893
CID000004913	CID000004915	CID000004920	CID000005002	CID000005005
CID000005029	CID000005035	CID000005038	CID000005039	CID000005040
CID000005064	CID000005070	CID000005073	CID000005076	CID000005077
CID000005078	CID000005090	CID000005095	CID000005152	CID000005155
CID000005195	CID000005203	CID000005210	CID000005212	CID000005215
CID000005245	CID000005291	CID000005344	CID000005352	CID000005358
CID000005372	CID000005376	CID000005379	CID000005394	CID000005401
CID000005402	CID000005408	CID000005412	CID000005426	CID000005466
CID000005478	CID000005479	CID000005487	CID000005512	CID000005514
CID000005515	CID000005523	CID000005525	CID000005533	CID000005538
CID000005625	CID000005645	CID000005647	CID000005650	CID000005656
CID000005665	CID000005672	CID000005717	CID000005718	CID000005719
CID000005721	CID000005726	CID000005731	CID000005732	CID000005734
CID000005978	CID000006049	CID000010631	CID000014888	CID000027661
CID000027686	CID000028112	CID000034312	CID000039860	CID000040976
CID000041317	CID000041781	CID000042615	CID000047725	CID000054454
CID000054786	CID000057469	CID000057537	CID000059708	CID000060184
CID000060198	CID000060613	CID000060753	CID000060754	CID000060787
CID000060795	CID000060843	CID000060852	CID000060877	CID000060953
CID000062816	CID000062924	CID000062959	CID000064147	CID000065999
CID000068740	CID000071158	CID000071273	CID000071329	CID000071616
CID000072054	CID000072938	CID000077992	CID000077993	CID000077999
CID000082146	CID000083786	CID000093860	CID000096312	CID000104758
CID000104865	CID000110634	CID000110635	CID000115237	CID000119182
CID000119607	CID000122316	CID000123606	CID000123620	CID000125889

CID000130881	CID000147912	CID000148192	CID000148211	CID000150311
CID000150610	CID000151165	CID000158440	CID000170361	CID000176870
CID000197712	CID000213039	CID000216239	CID000216326	CID000444013
CID000444033	CID000477468	CID000657298	CID001349907	CID003062316
CID003081884	CID004183806	CID004659568	CID004659569	CID005281104
CID005311297	CID005329102	CID005353980	CID005361912	CID005362070
CID005362420	CID005481350	CID005493381	CID005493444	CID006323497
CID006435110	CID006436173	CID006447131	CID009571074	

(31) $\mathbb{S}_{31}$: 337 drug compounds having side effect "Vertigo"

CID000000085	CID000000401	CID000000444	CID000000450	CID000000453
CID000000750	CID000000767	CID000000807	CID000000815	CID000000937
CID000000942	CID000001065	CID000001690	CID000001775	CID000001935
CID000001972	CID000001978	CID000002082	CID000002083	CID000002088
CID000002092	CID000002118	CID000002123	CID000002131	CID000002156
CID000002162	CID000002244	CID000002249	CID000002267	CID000002269
CID000002274	CID000002311	CID000002315	CID000002369	CID000002405
CID000002431	CID000002443	CID000002462	CID000002471	CID000002476
CID000002477	CID000002487	CID000002512	CID000002520	CID000002541
CID000002554	CID000002564	CID000002576	CID000002585	CID000002609
CID000002610	CID000002662	CID000002676	CID000002678	CID000002720
CID000002725	CID000002732	CID000002751	CID000002764	CID000002771
CID000002781	CID000002786	CID000002800	CID000002801	CID000002802
CID000002806	CID000002818	CID000002895	CID000002909	CID000002913
CID000003003	CID000003009	CID000003016	CID000003032	CID000003059
CID000003066	CID000003075	CID000003100	CID000003108	CID000003114
CID000003121	CID000003148	CID000003152	CID000003154	CID000003157
CID000003158	CID000003222	CID000003255	CID000003278	CID000003285
CID000003308	CID000003310	CID000003339	CID000003345	CID000003354
CID000003355	CID000003365	CID000003366	CID000003373	CID000003379
CID000003381	CID000003386	CID000003394	CID000003403	CID000003404
CID000003406	CID000003419	CID000003440	CID000003446	CID000003449
CID000003463	CID000003467	CID000003478	CID000003510	CID000003519
CID000003559	CID000003639	CID000003640	CID000003647	CID000003648
CID000003652	CID000003661	CID000003672	CID000003702	CID000003715
CID000003724	CID000003736	CID000003741	CID000003749	CID000003750
CID000003779	CID000003793	CID000003825	CID000003826	CID000003869
CID000003878	CID000003883	CID000003899	CID000003902	CID000003911
CID000003928	CID000003929	CID000003937	CID000003948	CID000003958
CID000003961	CID000003962	CID000004033	CID000004044	CID000004046
CID000004054	CID000004064	CID000004075	CID000004107	CID000004121
CID000004158	CID000004159	CID000004170	CID000004171	CID000004173
CID000004192	CID000004200	CID000004205	CID000004211	CID000004236
CID000004253	CID000004259	CID000004409	CID000004419	CID000004421

CID000004440	CID000004449	CID000004473	CID000004485	CID000004509
CID000004510	CID000004539	CID000004542	CID000004583	CID000004585
CID000004594	CID000004603	CID000004609	CID000004614	CID000004616
CID000004635	CID000004666	CID000004679	CID000004691	CID000004739
CID000004745	CID000004828	CID000004856	CID000004870	CID000004885
CID000004889	CID000004891	CID000004893	CID000004894	CID000004900
CID000004909	CID000004920	CID000004932	CID000004946	CID000004991
CID000005002	CID000005005	CID000005029	CID000005035	CID000005038
CID000005039	CID000005064	CID000005070	CID000005073	CID000005076
CID000005077	CID000005078	CID000005090	CID000005095	CID000005195
CID000005203	CID000005210	CID000005212	CID000005215	CID000005245
CID000005253	CID000005291	CID000005297	CID000005344	CID000005352
CID000005358	CID000005372	CID000005379	CID000005391	CID000005394
CID000005401	CID000005408	CID000005426	CID000005466	CID000005478
CID000005479	CID000005487	CID000005496	CID000005503	CID000005512
CID000005514	CID000005516	CID000005523	CID000005525	CID000005533
CID000005544	CID000005625	CID000005645	CID000005650	CID000005651
CID000005656	CID000005665	CID000005672	CID000005718	CID000005719
CID000005726	CID000005731	CID000005732	CID000005734	CID000005735
CID000005978	CID000010631	CID000013342	CID000014888	CID000027661
CID000027686	CID000028112	CID000031378	CID000034312	CID000039860
CID000042615	CID000051634	CID000054454	CID000054547	CID000054688
CID000056959	CID000057537	CID000059708	CID000060184	CID000060613
CID000060787	CID000060795	CID000060852	CID000060953	CID000062816
CID000062959	CID000064147	CID000065999	CID000068740	CID000071158
CID000071273	CID000071301	CID000071616	CID000072938	CID000077992
CID000077993	CID000083786	CID000093860	CID000104741	CID000104865
CID000110634	CID000110635	CID000119182	CID000119607	CID000122316
CID000123606	CID000125889	CID000130881	CID000147912	CID000150610
CID000158440	CID000170361	CID000213039	CID000216326	CID000222786
CID000444013	CID000450096	CID000657298	CID001349907	CID003002190
CID003062316	CID003081884	CID004659569	CID005229711	CID005281104
CID005282044	CID005353980	CID005362420	CID005487301	CID005493444
CID006398525	CID006435110			

(32) $\mathbb{S}_{32}$: 331 drug compounds having side effect “Myalgia”

CID000000085	CID000000158	CID000000159	CID000000444	CID000000450
CID000000564	CID000000596	CID000000598	CID000000738	CID000000937
CID000000942	CID000001046	CID000001065	CID000001134	CID000001546
CID000001690	CID000001775	CID000001935	CID000001971	CID000001972
CID000001978	CID000002022	CID000002083	CID000002088	CID000002099
CID000002118	CID000002145	CID000002162	CID000002182	CID000002187
CID000002216	CID000002250	CID000002265	CID000002267	CID000002269
CID000002284	CID000002311	CID000002349	CID000002369	CID000002375

CID000002405	CID000002435	CID000002462	CID000002478	CID000002520
CID000002524	CID000002541	CID000002550	CID000002554	CID000002578
CID000002585	CID000002609	CID000002662	CID000002676	CID000002678
CID000002713	CID000002751	CID000002756	CID000002764	CID000002769
CID000002771	CID000002786	CID000002800	CID000002801	CID000002802
CID000002806	CID000002818	CID000002891	CID000002895	CID000002909
CID000002951	CID000002958	CID000002973	CID000002978	CID000003032
CID000003040	CID000003043	CID000003066	CID000003075	CID000003108
CID000003121	CID000003143	CID000003148	CID000003152	CID000003157
CID000003161	CID000003203	CID000003222	CID000003251	CID000003261
CID000003285	CID000003325	CID000003333	CID000003339	CID000003345
CID000003348	CID000003355	CID000003365	CID000003367	CID000003379
CID000003385	CID000003394	CID000003403	CID000003404	CID000003410
CID000003414	CID000003417	CID000003419	CID000003446	CID000003449
CID000003454	CID000003461	CID000003463	CID000003475	CID000003478
CID000003488	CID000003518	CID000003519	CID000003648	CID000003702
CID000003749	CID000003793	CID000003825	CID000003826	CID000003877
CID000003878	CID000003883	CID000003899	CID000003902	CID000003911
CID000003937	CID000003948	CID000003961	CID000003962	CID000004046
CID000004053	CID000004075	CID000004091	CID000004112	CID000004138
CID000004158	CID000004163	CID000004200	CID000004205	CID000004212
CID000004236	CID000004259	CID000004428	CID000004440	CID000004449
CID000004451	CID000004463	CID000004473	CID000004485	CID000004497
CID000004509	CID000004513	CID000004539	CID000004542	CID000004583
CID000004585	CID000004594	CID000004599	CID000004607	CID000004609
CID000004635	CID000004666	CID000004679	CID000004739	CID000004740
CID000004745	CID000004819	CID000004828	CID000004829	CID000004885
CID000004889	CID000004891	CID000004893	CID000004913	CID000004915
CID000004920	CID000004932	CID000004943	CID000004991	CID000005005
CID000005029	CID000005035	CID000005038	CID000005039	CID000005040
CID000005064	CID000005070	CID000005073	CID000005076	CID000005077
CID000005078	CID000005090	CID000005095	CID000005152	CID000005155
CID000005195	CID000005210	CID000005212	CID000005245	CID000005253
CID000005291	CID000005314	CID000005344	CID000005352	CID000005358
CID000005372	CID000005376	CID000005379	CID000005394	CID000005401
CID000005402	CID000005408	CID000005426	CID000005466	CID000005479
CID000005496	CID000005514	CID000005515	CID000005523	CID000005525
CID000005538	CID000005544	CID000005625	CID000005645	CID000005650
CID000005656	CID000005672	CID000005717	CID000005718	CID000005719
CID000005726	CID000005731	CID000005732	CID000005734	CID000005735
CID000005978	CID000006049	CID000007029	CID000010631	CID000014888
CID000027400	CID000027661	CID000027686	CID000031477	CID000039042
CID000039860	CID000040976	CID000041317	CID000041781	CID000042113
CID000042615	CID000054454	CID000054547	CID000054786	CID000057469

CID000057537 CID000059708 CID000060184 CID000060198 CID000060613
CID000060753 CID000060754 CID000060787 CID000060795 CID000060843
CID000060852 CID000060877 CID000060953 CID000062924 CID000062959
CID000065027 CID000065999 CID000068740 CID000071158 CID000071273
CID000071616 CID000072938 CID000074989 CID000077992 CID000077993
CID000082146 CID000083786 CID000093860 CID000096312 CID000104741
CID000110634 CID000110635 CID000119182 CID000119607 CID000123606
CID000123620 CID000124087 CID000125889 CID000130881 CID000147912
CID000148192 CID000148211 CID000150311 CID000151165 CID000158440
CID000160051 CID000170361 CID000176870 CID000197712 CID000213039
CID000216239 CID000216326 CID000444013 CID000657298 CID001349907
CID003062316 CID003081884 CID004183806 CID004659569 CID005229711
CID005281007 CID005281104 CID005282044 CID005311297 CID005329102
CID005361912 CID005362070 CID005481350 CID005487301 CID006436173
CID009571074

(33) $\mathbb{S}_{33}$: 334 drug compounds having side effect “Anxiety”

CID000000085 CID000000159 CID000000444 CID000000450 CID000000596
CID000000598 CID000000681 CID000000807 CID000000838 CID000000853
CID000000942 CID000000951 CID000001690 CID000001935 CID000001971
CID000001972 CID000001978 CID000002083 CID000002099 CID000002130
CID000002131 CID000002160 CID000002162 CID000002171 CID000002187
CID000002215 CID000002249 CID000002267 CID000002269 CID000002284
CID000002311 CID000002349 CID000002369 CID000002375 CID000002405
CID000002431 CID000002435 CID000002443 CID000002462 CID000002474
CID000002476 CID000002478 CID000002487 CID000002512 CID000002541
CID000002554 CID000002578 CID000002609 CID000002662 CID000002676
CID000002678 CID000002751 CID000002756 CID000002762 CID000002764
CID000002769 CID000002786 CID000002800 CID000002801 CID000002802
CID000002803 CID000002806 CID000002818 CID000002891 CID000002895
CID000002909 CID000002949 CID000002958 CID000002978 CID000002995
CID000003007 CID000003019 CID000003032 CID000003062 CID000003066
CID000003080 CID000003108 CID000003121 CID000003125 CID000003148
CID000003152 CID000003154 CID000003157 CID000003161 CID000003168
CID000003203 CID000003261 CID000003285 CID000003308 CID000003325
CID000003333 CID000003339 CID000003340 CID000003345 CID000003355
CID000003373 CID000003379 CID000003387 CID000003394 CID000003397
CID000003403 CID000003406 CID000003410 CID000003414 CID000003446
CID000003449 CID000003454 CID000003475 CID000003478 CID000003510
CID000003519 CID000003559 CID000003637 CID000003648 CID000003661
CID000003672 CID000003676 CID000003696 CID000003702 CID000003715
CID000003724 CID000003736 CID000003741 CID000003749 CID000003793
CID000003821 CID000003826 CID000003877 CID000003878 CID000003883
CID000003899 CID000003902 CID000003911 CID000003948 CID000003961

CID000003962 CID000004044 CID000004046 CID000004054 CID000004062
CID000004075 CID000004095 CID000004158 CID000004160 CID000004168
CID000004170 CID000004171 CID000004192 CID000004195 CID000004196
CID000004205 CID000004212 CID000004236 CID000004253 CID000004259
CID000004409 CID000004419 CID000004428 CID000004440 CID000004449
CID000004451 CID000004473 CID000004485 CID000004493 CID000004513
CID000004539 CID000004542 CID000004543 CID000004583 CID000004585
CID000004594 CID000004595 CID000004599 CID000004603 CID000004609
CID000004614 CID000004634 CID000004635 CID000004679 CID000004727
CID000004737 CID000004739 CID000004740 CID000004745 CID000004819
CID000004828 CID000004845 CID000004856 CID000004885 CID000004889
CID000004893 CID000004914 CID000004920 CID000004932 CID000004943
CID000004946 CID000004976 CID000005002 CID000005005 CID000005029
CID000005038 CID000005040 CID000005052 CID000005064 CID000005070
CID000005071 CID000005073 CID000005076 CID000005077 CID000005078
CID000005090 CID000005095 CID000005152 CID000005193 CID000005195
CID000005210 CID000005212 CID000005245 CID000005253 CID000005291
CID000005344 CID000005358 CID000005372 CID000005376 CID000005379
CID000005391 CID000005394 CID000005401 CID000005403 CID000005408
CID000005426 CID000005466 CID000005478 CID000005487 CID000005512
CID000005514 CID000005523 CID000005525 CID000005530 CID000005538
CID000005556 CID000005584 CID000005625 CID000005650 CID000005665
CID000005718 CID000005719 CID000005721 CID000005726 CID000005731
CID000005732 CID000005734 CID000005735 CID000007029 CID000008612
CID000009034 CID000010631 CID000014888 CID000018140 CID000027661
CID000027686 CID000031477 CID000034312 CID000039860 CID000040976
CID000041317 CID000042615 CID000047725 CID000054454 CID000054547
CID000054688 CID000054786 CID000057469 CID000057537 CID000059708
CID000059768 CID000060184 CID000060198 CID000060613 CID000060714
CID000060754 CID000060787 CID000060795 CID000062816 CID000062819
CID000062867 CID000062924 CID000062959 CID000064147 CID000065999
CID000068740 CID000071158 CID000071273 CID000071329 CID000071616
CID000074989 CID000077992 CID000077993 CID000093860 CID000104741
CID000104865 CID000110634 CID000115237 CID000119182 CID000119607
CID000122316 CID000123606 CID000125017 CID000125889 CID000147912
CID000148192 CID000148211 CID000150610 CID000151165 CID000158440
CID000163742 CID000170361 CID000176870 CID000213039 CID000216326
CID000444013 CID000477468 CID003002190 CID003062316 CID003081884
CID004183806 CID004659568 CID004659569 CID005229711 CID005282226
CID005311027 CID005311297 CID005481350 CID005493381

(34) $\mathbb{S}_{34}$: 328 drug compounds having side effect "Syncope"

CID000000158 CID000000159 CID000000206 CID000000214 CID000000444
CID000000450 CID000000564 CID000000581 CID000000596 CID000000767

CID000000807	CID000000937	CID000001003	CID000001065	CID000001775
CID000001935	CID000001972	CID000001978	CID000002083	CID000002092
CID000002140	CID000002141	CID000002160	CID000002162	CID000002170
CID000002182	CID000002249	CID000002250	CID000002269	CID000002284
CID000002311	CID000002349	CID000002369	CID000002375	CID000002405
CID000002431	CID000002435	CID000002443	CID000002462	CID000002474
CID000002477	CID000002487	CID000002512	CID000002520	CID000002541
CID000002550	CID000002554	CID000002576	CID000002583	CID000002585
CID000002609	CID000002662	CID000002678	CID000002712	CID000002726
CID000002751	CID000002764	CID000002771	CID000002800	CID000002801
CID000002803	CID000002806	CID000002818	CID000002895	CID000002913
CID000002949	CID000002958	CID000003003	CID000003007	CID000003015
CID000003016	CID000003032	CID000003042	CID000003059	CID000003075
CID000003108	CID000003114	CID000003143	CID000003148	CID000003152
CID000003154	CID000003157	CID000003168	CID000003203	CID000003222
CID000003261	CID000003285	CID000003308	CID000003325	CID000003333
CID000003345	CID000003355	CID000003379	CID000003386	CID000003393
CID000003394	CID000003404	CID000003419	CID000003446	CID000003449
CID000003463	CID000003478	CID000003510	CID000003518	CID000003519
CID000003640	CID000003648	CID000003661	CID000003672	CID000003676
CID000003696	CID000003702	CID000003715	CID000003724	CID000003730
CID000003734	CID000003736	CID000003737	CID000003741	CID000003742
CID000003749	CID000003750	CID000003759	CID000003780	CID000003784
CID000003793	CID000003826	CID000003827	CID000003869	CID000003878
CID000003883	CID000003911	CID000003914	CID000003937	CID000003948
CID000003961	CID000003964	CID000004011	CID000004032	CID000004044
CID000004046	CID000004054	CID000004058	CID000004062	CID000004064
CID000004078	CID000004086	CID000004091	CID000004095	CID000004107
CID000004158	CID000004170	CID000004171	CID000004173	CID000004178
CID000004196	CID000004200	CID000004205	CID000004236	CID000004253
CID000004259	CID000004409	CID000004419	CID000004428	CID000004440
CID000004449	CID000004473	CID000004485	CID000004493	CID000004506
CID000004510	CID000004542	CID000004583	CID000004585	CID000004594
CID000004595	CID000004609	CID000004614	CID000004616	CID000004634
CID000004635	CID000004666	CID000004679	CID000004691	CID000004736
CID000004737	CID000004739	CID000004745	CID000004748	CID000004775
CID000004819	CID000004828	CID000004845	CID000004856	CID000004885
CID000004893	CID000004894	CID000004914	CID000004915	CID000004920
CID000004927	CID000004932	CID000004943	CID000005002	CID000005005
CID000005029	CID000005035	CID000005038	CID000005040	CID000005052
CID000005070	CID000005071	CID000005073	CID000005076	CID000005077
CID000005078	CID000005090	CID000005095	CID000005193	CID000005195
CID000005203	CID000005206	CID000005210	CID000005212	CID000005245
CID000005253	CID000005291	CID000005344	CID000005352	CID000005358

CID000005372	CID000005379	CID000005401	CID000005426	CID000005454
CID000005466	CID000005478	CID000005487	CID000005514	CID000005523
CID000005525	CID000005533	CID000005538	CID000005544	CID000005556
CID000005591	CID000005596	CID000005625	CID000005645	CID000005650
CID000005656	CID000005718	CID000005719	CID000005721	CID000005726
CID000005731	CID000005732	CID000005734	CID000005746	CID000006691
CID000008612	CID000010631	CID000016850	CID000027661	CID000028112
CID000034312	CID000039860	CID000041781	CID000042615	CID000054547
CID000054786	CID000056959	CID000057469	CID000057537	CID000059768
CID000060184	CID000060613	CID000060714	CID000060753	CID000060754
CID000060787	CID000060953	CID000062867	CID000062959	CID000065999
CID000068740	CID000071158	CID000071273	CID000071301	CID000071329
CID000071616	CID000072938	CID000077992	CID000077993	CID000082146
CID000093860	CID000104865	CID000110634	CID000110635	CID000115237
CID000119607	CID000122316	CID000123606	CID000125017	CID000125889
CID000147912	CID000148192	CID000150610	CID000151165	CID000158440
CID000170361	CID000176870	CID000216239	CID000216326	CID000444013
CID003002190	CID003062316	CID003081884	CID004183806	CID004659568
CID004659569	CID005229711	CID005281104	CID005282044	CID005311181
CID005362420	CID005487301	CID006436173		

(35) $\mathbb{S}_{35}$: 325 drug compounds having side effect “Hypertension”

CID000000085	CID000000158	CID000000191	CID000000214	CID000000444
CID000000453	CID000000564	CID000000596	CID000000598	CID000000681
CID000000807	CID000000838	CID000000861	CID000000942	CID000000951
CID000001690	CID000001775	CID000001935	CID000001972	CID000002022
CID000002083	CID000002130	CID000002140	CID000002141	CID000002160
CID000002170	CID000002182	CID000002187	CID000002267	CID000002269
CID000002284	CID000002349	CID000002375	CID000002431	CID000002435
CID000002443	CID000002462	CID000002474	CID000002476	CID000002477
CID000002478	CID000002487	CID000002524	CID000002554	CID000002578
CID000002662	CID000002676	CID000002678	CID000002713	CID000002764
CID000002771	CID000002786	CID000002800	CID000002806	CID000002818
CID000002895	CID000002909	CID000002958	CID000002995	CID000003003
CID000003007	CID000003032	CID000003066	CID000003105	CID000003108
CID000003121	CID000003143	CID000003148	CID000003152	CID000003154
CID000003158	CID000003161	CID000003168	CID000003203	CID000003249
CID000003285	CID000003308	CID000003310	CID000003339	CID000003345
CID000003355	CID000003372	CID000003373	CID000003379	CID000003386
CID000003394	CID000003397	CID000003404	CID000003410	CID000003414
CID000003446	CID000003449	CID000003454	CID000003461	CID000003467
CID000003475	CID000003478	CID000003494	CID000003510	CID000003559
CID000003640	CID000003648	CID000003672	CID000003676	CID000003690
CID000003696	CID000003715	CID000003724	CID000003730	CID000003734

CID000003736	CID000003737	CID000003741	CID000003746	CID000003750
CID000003779	CID000003780	CID000003793	CID000003825	CID000003826
CID000003827	CID000003878	CID000003883	CID000003899	CID000003902
CID000003911	CID000003929	CID000003948	CID000003958	CID000003964
CID000004011	CID000004044	CID000004046	CID000004054	CID000004062
CID000004075	CID000004086	CID000004091	CID000004140	CID000004158
CID000004159	CID000004168	CID000004173	CID000004178	CID000004195
CID000004200	CID000004205	CID000004211	CID000004212	CID000004236
CID000004253	CID000004259	CID000004409	CID000004419	CID000004425
CID000004436	CID000004449	CID000004493	CID000004497	CID000004542
CID000004543	CID000004547	CID000004583	CID000004585	CID000004594
CID000004609	CID000004614	CID000004634	CID000004679	CID000004691
CID000004736	CID000004739	CID000004740	CID000004745	CID000004748
CID000004819	CID000004829	CID000004856	CID000004885	CID000004894
CID000004900	CID000004914	CID000004915	CID000004920	CID000004932
CID000004943	CID000004976	CID000005002	CID000005029	CID000005040
CID000005070	CID000005071	CID000005073	CID000005076	CID000005077
CID000005078	CID000005090	CID000005095	CID000005152	CID000005195
CID000005203	CID000005206	CID000005245	CID000005291	CID000005314
CID000005344	CID000005352	CID000005358	CID000005372	CID000005376
CID000005379	CID000005394	CID000005396	CID000005408	CID000005412
CID000005426	CID000005466	CID000005486	CID000005504	CID000005514
CID000005523	CID000005533	CID000005538	CID000005544	CID000005584
CID000005625	CID000005636	CID000005645	CID000005647	CID000005656
CID000005672	CID000005718	CID000005719	CID000005731	CID000005732
CID000005734	CID000005735	CID000005746	CID000005775	CID000005978
CID000006058	CID000013342	CID000014888	CID000018140	CID000019090
CID000027661	CID000027991	CID000031378	CID000031477	CID000032800
CID000034312	CID000036339	CID000038904	CID000039860	CID000040976
CID000041317	CID000041693	CID000042113	CID000047725	CID000051263
CID000054547	CID000057469	CID000057537	CID000060198	CID000060612
CID000060613	CID000060714	CID000060753	CID000060787	CID000060795
CID000060843	CID000060852	CID000060871	CID000060953	CID000062819
CID000062924	CID000062959	CID000064147	CID000068740	CID000071158
CID000071273	CID000071329	CID000071616	CID000072054	CID000077993
CID000077999	CID000082146	CID000093860	CID000110634	CID000110635
CID000119182	CID000119607	CID000123606	CID000125889	CID000147912
CID000148192	CID000148211	CID000150610	CID000151165	CID000158440
CID000163742	CID000166548	CID000170361	CID000213039	CID000216239
CID000216326	CID000222786	CID000444013	CID000444033	CID000477468
CID003062316	CID003081884	CID004183806	CID004659568	CID004659569
CID005229711	CID005281104	CID005282044	CID005282226	CID005311027
CID005329102	CID005362420	CID005473385	CID006323497	CID006398970

(36) $\mathbb{S}_{36}$: 326 drug compounds having side effect "Anemia"

CID000000085	CID000000159	CID000000214	CID000000298	CID000000444
CID000000596	CID000000598	CID000000772	CID000001775	CID000001935
CID000001971	CID000001972	CID000002022	CID000002088	CID000002140
CID000002142	CID000002145	CID000002156	CID000002171	CID000002173
CID000002179	CID000002182	CID000002187	CID000002244	CID000002250
CID000002269	CID000002274	CID000002284	CID000002369	CID000002375
CID000002462	CID000002478	CID000002541	CID000002550	CID000002554
CID000002559	CID000002575	CID000002578	CID000002585	CID000002617
CID000002622	CID000002637	CID000002650	CID000002655	CID000002656
CID000002662	CID000002676	CID000002708	CID000002726	CID000002751
CID000002756	CID000002764	CID000002769	CID000002771	CID000002786
CID000002794	CID000002801	CID000002802	CID000002806	CID000002818
CID000002907	CID000002909	CID000002951	CID000003015	CID000003016
CID000003032	CID000003043	CID000003075	CID000003100	CID000003121
CID000003125	CID000003143	CID000003148	CID000003152	CID000003161
CID000003203	CID000003222	CID000003249	CID000003305	CID000003308
CID000003310	CID000003324	CID000003325	CID000003333	CID000003339
CID000003342	CID000003345	CID000003365	CID000003366	CID000003367
CID000003385	CID000003386	CID000003394	CID000003397	CID000003404
CID000003406	CID000003414	CID000003419	CID000003440	CID000003446
CID000003449	CID000003454	CID000003461	CID000003463	CID000003467
CID000003475	CID000003476	CID000003478	CID000003488	CID000003510
CID000003518	CID000003559	CID000003648	CID000003652	CID000003657
CID000003672	CID000003685	CID000003690	CID000003702	CID000003706
CID000003715	CID000003749	CID000003750	CID000003825	CID000003826
CID000003877	CID000003878	CID000003883	CID000003899	CID000003929
CID000003937	CID000003948	CID000003950	CID000003961	CID000004044
CID000004053	CID000004054	CID000004060	CID000004075	CID000004078
CID000004112	CID000004158	CID000004170	CID000004196	CID000004205
CID000004212	CID000004236	CID000004253	CID000004259	CID000004409
CID000004440	CID000004449	CID000004451	CID000004463	CID000004485
CID000004493	CID000004497	CID000004509	CID000004513	CID000004542
CID000004583	CID000004585	CID000004594	CID000004603	CID000004609
CID000004614	CID000004635	CID000004666	CID000004679	CID000004691
CID000004740	CID000004745	CID000004775	CID000004829	CID000004856
CID000004885	CID000004889	CID000004909	CID000004911	CID000004915
CID000004920	CID000004932	CID000004943	CID000005002	CID000005005
CID000005029	CID000005039	CID000005040	CID000005064	CID000005070
CID000005073	CID000005076	CID000005077	CID000005095	CID000005155
CID000005195	CID000005203	CID000005210	CID000005212	CID000005245
CID000005291	CID000005300	CID000005342	CID000005344	CID000005352
CID000005358	CID000005372	CID000005376	CID000005394	CID000005396
CID000005402	CID000005408	CID000005412	CID000005426	CID000005452

CID000005466 CID000005487 CID000005496 CID000005514 CID000005515
CID000005523 CID000005525 CID000005530 CID000005533 CID000005538
CID000005578 CID000005582 CID000005591 CID000005625 CID000005645
CID000005656 CID000005672 CID000005718 CID000005719 CID000005726
CID000005732 CID000005734 CID000005735 CID000005746 CID000005978
CID000006049 CID000006058 CID000006691 CID000010631 CID000013342
CID000014888 CID000016362 CID000018140 CID000019090 CID000025419
CID000027661 CID000027686 CID000030623 CID000038904 CID000039042
CID000039860 CID000047725 CID000054454 CID000054547 CID000054688
CID000054786 CID000060184 CID000060612 CID000060613 CID000060787
CID000060795 CID000060843 CID000060877 CID000060953 CID000062867
CID000062959 CID000064147 CID000065027 CID000065999 CID000068740
CID000071158 CID000071273 CID000071616 CID000072938 CID000074989
CID000077993 CID000077999 CID000082146 CID000083786 CID000093860
CID000096312 CID000104741 CID000104758 CID000104865 CID000119182
CID000119607 CID000122316 CID000123631 CID000125889 CID000130881
CID000147912 CID000148211 CID000150610 CID000151165 CID000163742
CID000170361 CID000197712 CID000213039 CID000216239 CID000477468
CID000667490 CID003002190 CID003062316 CID003081884 CID004183806
CID004659569 CID005281104 CID005282044 CID005329102 CID005353980
CID005361912 CID005362070 CID005362420 CID005381226 CID005481350
CID005493381 CID005493444 CID006323497 CID006398970 CID006435110
CID006918453

(37) $\mathbb{S}_{37}$: 322 drug compounds having side effect “Chest pain”

CID000000085 CID000000158 CID000000159 CID000000191 CID000000444
CID000000450 CID000000453 CID000000596 CID000000598 CID000000738
CID000000807 CID000001065 CID000001134 CID000001935 CID000001972
CID000001978 CID000002022 CID000002083 CID000002092 CID000002140
CID000002141 CID000002162 CID000002182 CID000002187 CID000002215
CID000002216 CID000002232 CID000002249 CID000002250 CID000002267
CID000002269 CID000002274 CID000002284 CID000002311 CID000002369
CID000002375 CID000002405 CID000002435 CID000002462 CID000002471
CID000002477 CID000002478 CID000002487 CID000002520 CID000002541
CID000002550 CID000002554 CID000002578 CID000002585 CID000002609
CID000002658 CID000002662 CID000002676 CID000002678 CID000002751
CID000002764 CID000002769 CID000002771 CID000002800 CID000002801
CID000002803 CID000002806 CID000002818 CID000002895 CID000002909
CID000002958 CID000003007 CID000003032 CID000003059 CID000003075
CID000003108 CID000003114 CID000003121 CID000003143 CID000003148
CID000003152 CID000003154 CID000003156 CID000003157 CID000003203
CID000003222 CID000003255 CID000003261 CID000003285 CID000003292
CID000003333 CID000003339 CID000003340 CID000003345 CID000003355
CID000003366 CID000003373 CID000003379 CID000003385 CID000003393

CID000003403	CID000003404	CID000003410	CID000003414	CID000003419
CID000003446	CID000003449	CID000003454	CID000003461	CID000003463
CID000003475	CID000003510	CID000003518	CID000003519	CID000003648
CID000003661	CID000003698	CID000003702	CID000003715	CID000003724
CID000003734	CID000003736	CID000003741	CID000003746	CID000003749
CID000003783	CID000003784	CID000003793	CID000003826	CID000003878
CID000003883	CID000003890	CID000003899	CID000003902	CID000003914
CID000003937	CID000003948	CID000003961	CID000003962	CID000004046
CID000004053	CID000004054	CID000004075	CID000004086	CID000004091
CID000004112	CID000004140	CID000004158	CID000004163	CID000004170
CID000004171	CID000004173	CID000004178	CID000004197	CID000004205
CID000004212	CID000004236	CID000004253	CID000004259	CID000004428
CID000004449	CID000004473	CID000004485	CID000004493	CID000004509
CID000004513	CID000004539	CID000004542	CID000004547	CID000004583
CID000004585	CID000004594	CID000004595	CID000004609	CID000004634
CID000004635	CID000004666	CID000004679	CID000004739	CID000004740
CID000004745	CID000004819	CID000004828	CID000004829	CID000004845
CID000004885	CID000004889	CID000004893	CID000004920	CID000004932
CID000004943	CID000005002	CID000005005	CID000005035	CID000005038
CID000005039	CID000005040	CID000005064	CID000005070	CID000005073
CID000005076	CID000005077	CID000005078	CID000005090	CID000005095
CID000005152	CID000005195	CID000005210	CID000005212	CID000005234
CID000005245	CID000005253	CID000005291	CID000005352	CID000005358
CID000005372	CID000005376	CID000005379	CID000005401	CID000005466
CID000005478	CID000005496	CID000005508	CID000005512	CID000005514
CID000005515	CID000005523	CID000005525	CID000005533	CID000005538
CID000005556	CID000005596	CID000005625	CID000005645	CID000005650
CID000005665	CID000005672	CID000005719	CID000005726	CID000005731
CID000005732	CID000005734	CID000005735	CID000006691	CID000010631
CID000014888	CID000018140	CID000019090	CID000027661	CID000027686
CID000027991	CID000034312	CID000036811	CID000039860	CID000041317
CID000041744	CID000041781	CID000050294	CID000054547	CID000054688
CID000054786	CID000057469	CID000059708	CID000059768	CID000060184
CID000060198	CID000060613	CID000060714	CID000060753	CID000060754
CID000060787	CID000060795	CID000060843	CID000060953	CID000062816
CID000062819	CID000062959	CID000065999	CID000068740	CID000068844
CID000071158	CID000071273	CID000071301	CID000071329	CID000071616
CID000077992	CID000082146	CID000096312	CID000104741	CID000104865
CID000110634	CID000110635	CID000119607	CID000122316	CID000123606
CID000123620	CID000125889	CID000130881	CID000147912	CID000148192
CID000150311	CID000150610	CID000158440	CID000163742	CID000170361
CID000197712	CID000216326	CID000444013	CID000444033	CID000450096
CID003062316	CID003081884	CID003086672	CID004183806	CID004659569
CID005229711	CID005281104	CID005282044	CID005282226	CID005311027

CID005311181 CID005361912 CID005362420 CID005481350 CID005487301
CID006398970 CID006436173

(38) $\mathbb{S}_{38}$: 299 drug compounds having side effect “Infection”

CID000000085 CID000000143 CID000000159 CID000000206 CID000000214
CID000000444 CID000000450 CID000000453 CID000000596 CID000000738
CID000000767 CID000000807 CID000001546 CID000001690 CID000001775
CID000001935 CID000002083 CID000002088 CID000002092 CID000002099
CID000002118 CID000002153 CID000002179 CID000002182 CID000002187
CID000002232 CID000002250 CID000002265 CID000002267 CID000002284
CID000002311 CID000002369 CID000002375 CID000002405 CID000002435
CID000002462 CID000002476 CID000002478 CID000002520 CID000002554
CID000002578 CID000002585 CID000002662 CID000002678 CID000002708
CID000002726 CID000002751 CID000002771 CID000002801 CID000002802
CID000002803 CID000002806 CID000002818 CID000002891 CID000002907
CID000002909 CID000002958 CID000002973 CID000003000 CID000003003
CID000003007 CID000003016 CID000003032 CID000003075 CID000003121
CID000003143 CID000003152 CID000003157 CID000003241 CID000003285
CID000003305 CID000003308 CID000003310 CID000003325 CID000003333
CID000003339 CID000003340 CID000003345 CID000003348 CID000003367
CID000003375 CID000003381 CID000003382 CID000003385 CID000003386
CID000003392 CID000003394 CID000003403 CID000003404 CID000003410
CID000003417 CID000003419 CID000003446 CID000003454 CID000003461
CID000003475 CID000003510 CID000003553 CID000003640 CID000003648
CID000003657 CID000003672 CID000003676 CID000003685 CID000003690
CID000003702 CID000003715 CID000003737 CID000003746 CID000003749
CID000003750 CID000003825 CID000003826 CID000003827 CID000003878
CID000003890 CID000003899 CID000003902 CID000003911 CID000003937
CID000003958 CID000003961 CID000003962 CID000004033 CID000004044
CID000004053 CID000004075 CID000004091 CID000004112 CID000004114
CID000004158 CID000004159 CID000004196 CID000004201 CID000004205
CID000004212 CID000004236 CID000004253 CID000004428 CID000004440
CID000004449 CID000004473 CID000004510 CID000004513 CID000004599
CID000004609 CID000004614 CID000004634 CID000004635 CID000004666
CID000004679 CID000004691 CID000004739 CID000004740 CID000004745
CID000004748 CID000004819 CID000004856 CID000004873 CID000004885
CID000004889 CID000004894 CID000004915 CID000004917 CID000004920
CID000004932 CID000005002 CID000005035 CID000005040 CID000005070
CID000005073 CID000005077 CID000005090 CID000005095 CID000005152
CID000005195 CID000005210 CID000005212 CID000005234 CID000005245
CID000005253 CID000005291 CID000005320 CID000005358 CID000005372
CID000005376 CID000005394 CID000005396 CID000005408 CID000005426
CID000005453 CID000005466 CID000005478 CID000005487 CID000005512
CID000005514 CID000005515 CID000005523 CID000005525 CID000005538

CID000005544 CID000005596 CID000005645 CID000005656 CID000005672
CID000005717 CID000005718 CID000005721 CID000005731 CID000005732
CID000005735 CID000006503 CID000010631 CID000012536 CID000014888
CID000019090 CID000025419 CID000025517 CID000027661 CID000027686
CID000027991 CID000028112 CID000030623 CID000031378 CID000032797
CID000034312 CID000038904 CID000039042 CID000039860 CID000040976
CID000041317 CID000042615 CID000047725 CID000048175 CID000052421
CID000054454 CID000057469 CID000059708 CID000060184 CID000060198
CID000060612 CID000060787 CID000060795 CID000060843 CID000060852
CID000060865 CID000060953 CID000062819 CID000062924 CID000064147
CID000065999 CID000068740 CID000071158 CID000071273 CID000071329
CID000074989 CID000077993 CID000082146 CID000093860 CID000096312
CID000104758 CID000104865 CID000115237 CID000119182 CID000119607
CID000122316 CID000123620 CID000123631 CID000124087 CID000125889
CID000130881 CID000145068 CID000148211 CID000150311 CID000151165
CID000158440 CID000160051 CID000163742 CID000170361 CID000176870
CID000197712 CID000216239 CID000216326 CID000444033 CID003062316
CID003081884 CID004183806 CID004630253 CID004659569 CID005281104
CID005282226 CID005311027 CID005311297 CID005329102 CID005362070
CID005487301 CID005493381 CID006436173 CID006447131

(39) $\mathbb{S}_{39}$: 302 drug compounds having side effect “Palpitations”

CID000000085 CID000000159 CID000000191 CID000000444 CID000000450
CID000000681 CID000000838 CID000000853 CID000000937 CID000000942
CID000001065 CID000001206 CID000001690 CID000001775 CID000001935
CID000001978 CID000002083 CID000002160 CID000002162 CID000002170
CID000002182 CID000002216 CID000002249 CID000002250 CID000002267
CID000002269 CID000002284 CID000002311 CID000002349 CID000002369
CID000002405 CID000002435 CID000002441 CID000002462 CID000002477
CID000002487 CID000002512 CID000002520 CID000002541 CID000002550
CID000002564 CID000002583 CID000002585 CID000002609 CID000002656
CID000002658 CID000002662 CID000002678 CID000002751 CID000002762
CID000002764 CID000002769 CID000002771 CID000002781 CID000002800
CID000002801 CID000002803 CID000002806 CID000002818 CID000002895
CID000002913 CID000002958 CID000002978 CID000002995 CID000003007
CID000003016 CID000003019 CID000003032 CID000003042 CID000003059
CID000003062 CID000003066 CID000003075 CID000003100 CID000003108
CID000003121 CID000003148 CID000003151 CID000003154 CID000003157
CID000003168 CID000003203 CID000003222 CID000003255 CID000003261
CID000003285 CID000003308 CID000003325 CID000003333 CID000003339
CID000003340 CID000003342 CID000003345 CID000003348 CID000003354
CID000003355 CID000003373 CID000003379 CID000003393 CID000003394
CID000003404 CID000003410 CID000003414 CID000003419 CID000003446
CID000003449 CID000003475 CID000003476 CID000003494 CID000003510

CID000003519	CID000003637	CID000003648	CID000003661	CID000003672
CID000003696	CID000003702	CID000003715	CID000003746	CID000003759
CID000003779	CID000003784	CID000003825	CID000003826	CID000003869
CID000003878	CID000003883	CID000003899	CID000003902	CID000003911
CID000003914	CID000003937	CID000003957	CID000003961	CID000004011
CID000004036	CID000004044	CID000004046	CID000004057	CID000004058
CID000004064	CID000004075	CID000004086	CID000004091	CID000004095
CID000004140	CID000004158	CID000004170	CID000004171	CID000004173
CID000004178	CID000004196	CID000004205	CID000004236	CID000004253
CID000004259	CID000004409	CID000004428	CID000004440	CID000004449
CID000004473	CID000004485	CID000004493	CID000004497	CID000004506
CID000004510	CID000004539	CID000004542	CID000004543	CID000004547
CID000004583	CID000004585	CID000004594	CID000004595	CID000004601
CID000004614	CID000004635	CID000004679	CID000004740	CID000004745
CID000004771	CID000004819	CID000004828	CID000004845	CID000004856
CID000004885	CID000004893	CID000004920	CID000004932	CID000004934
CID000004976	CID000005002	CID000005005	CID000005029	CID000005038
CID000005040	CID000005070	CID000005071	CID000005073	CID000005076
CID000005077	CID000005078	CID000005090	CID000005095	CID000005152
CID000005195	CID000005210	CID000005212	CID000005253	CID000005344
CID000005352	CID000005358	CID000005372	CID000005379	CID000005391
CID000005394	CID000005401	CID000005403	CID000005419	CID000005426
CID000005466	CID000005478	CID000005479	CID000005512	CID000005514
CID000005523	CID000005525	CID000005530	CID000005533	CID000005538
CID000005584	CID000005596	CID000005625	CID000005645	CID000005650
CID000005718	CID000005719	CID000005731	CID000005732	CID000005734
CID000005735	CID000007029	CID000010631	CID000014888	CID000019090
CID000020585	CID000027661	CID000027686	CID000027991	CID000031477
CID000034312	CID000036811	CID000039860	CID000042615	CID000054547
CID000054786	CID000056959	CID000057469	CID000060184	CID000060753
CID000060795	CID000062924	CID000062959	CID000065999	CID000071158
CID000071329	CID000071616	CID000072054	CID000072938	CID000077992
CID000077993	CID000104865	CID000110634	CID000110635	CID000115237
CID000119607	CID000123606	CID000124087	CID000125017	CID000125889
CID000147912	CID000148192	CID000150311	CID000151165	CID000158440
CID000170361	CID000197712	CID000216326	CID000444013	CID000444033
CID000450096	CID003002190	CID003062316	CID003081884	CID004659569
CID005229711	CID005281104	CID005282044	CID005311027	CID005311181
CID005311297	CID011947681			

(40) $\mathbb{S}_{40}$: 308 drug compounds having side effect “Alopecia”

CID000000143	CID000000444	CID000000450	CID000000596	CID000000598
CID000000727	CID000000767	CID000000772	CID000000853	CID000000937
CID000001148	CID000001690	CID000001935	CID000001972	CID000001978

CID000002019	CID000002022	CID000002082	CID000002088	CID000002099
CID000002123	CID000002156	CID000002160	CID000002162	CID000002170
CID000002179	CID000002182	CID000002187	CID000002249	CID000002250
CID000002265	CID000002284	CID000002311	CID000002369	CID000002375
CID000002405	CID000002443	CID000002462	CID000002477	CID000002478
CID000002512	CID000002520	CID000002550	CID000002554	CID000002578
CID000002585	CID000002662	CID000002676	CID000002678	CID000002708
CID000002719	CID000002720	CID000002749	CID000002751	CID000002756
CID000002764	CID000002771	CID000002800	CID000002801	CID000002802
CID000002803	CID000002895	CID000002907	CID000002909	CID000002949
CID000002958	CID000002995	CID000003009	CID000003015	CID000003032
CID000003043	CID000003075	CID000003108	CID000003121	CID000003143
CID000003152	CID000003154	CID000003157	CID000003158	CID000003203
CID000003222	CID000003285	CID000003305	CID000003308	CID000003310
CID000003325	CID000003339	CID000003342	CID000003345	CID000003355
CID000003365	CID000003367	CID000003381	CID000003385	CID000003386
CID000003387	CID000003394	CID000003403	CID000003404	CID000003446
CID000003454	CID000003461	CID000003463	CID000003467	CID000003510
CID000003518	CID000003519	CID000003559	CID000003639	CID000003648
CID000003652	CID000003657	CID000003672	CID000003685	CID000003690
CID000003696	CID000003706	CID000003715	CID000003746	CID000003750
CID000003793	CID000003823	CID000003825	CID000003826	CID000003869
CID000003877	CID000003878	CID000003883	CID000003899	CID000003902
CID000003911	CID000003914	CID000003937	CID000003950	CID000003957
CID000003958	CID000003961	CID000003962	CID000003964	CID000004011
CID000004033	CID000004036	CID000004044	CID000004046	CID000004053
CID000004054	CID000004060	CID000004075	CID000004112	CID000004158
CID000004160	CID000004163	CID000004171	CID000004178	CID000004200
CID000004205	CID000004212	CID000004409	CID000004411	CID000004428
CID000004440	CID000004449	CID000004485	CID000004493	CID000004509
CID000004542	CID000004543	CID000004547	CID000004583	CID000004585
CID000004594	CID000004599	CID000004609	CID000004614	CID000004638
CID000004666	CID000004679	CID000004691	CID000004724	CID000004727
CID000004739	CID000004745	CID000004819	CID000004828	CID000004845
CID000004856	CID000004885	CID000004889	CID000004893	CID000004911
CID000004915	CID000004920	CID000004932	CID000004946	CID000004976
CID000004991	CID000005005	CID000005029	CID000005038	CID000005039
CID000005064	CID000005070	CID000005073	CID000005077	CID000005090
CID000005095	CID000005195	CID000005203	CID000005210	CID000005253
CID000005291	CID000005352	CID000005372	CID000005376	CID000005394
CID000005396	CID000005402	CID000005408	CID000005426	CID000005453
CID000005466	CID000005478	CID000005487	CID000005514	CID000005515
CID000005516	CID000005525	CID000005530	CID000005533	CID000005538
CID000005584	CID000005625	CID000005645	CID000005647	CID000005650

CID000005656 CID000005672 CID000005718 CID000005719 CID000005734
CID000005735 CID000005746 CID000005978 CID000006691 CID000007029
CID000009904 CID000010631 CID000013342 CID000018140 CID000019090
CID000020585 CID000024011 CID000028112 CID000030623 CID000032797
CID000034312 CID000038904 CID000039042 CID000040976 CID000041317
CID000047725 CID000054454 CID000054547 CID000057469 CID000059708
CID000060184 CID000060198 CID000060613 CID000060787 CID000060795
CID000060843 CID000060953 CID000064147 CID000068740 CID000068844
CID000071158 CID000071616 CID000077993 CID000082146 CID000083786
CID000104758 CID000119607 CID000122316 CID000123631 CID000125889
CID000130881 CID000147912 CID000148192 CID000148211 CID000151165
CID000176870 CID000213039 CID000216239 CID000216326 CID000657298
CID000667490 CID001349907 CID003062316 CID003081884 CID004183806
CID004659569 CID005281007 CID005281104 CID005329102 CID005353980
CID005362070 CID005487301 CID006918453

(41) $\mathbb{S}_{41}$: 301 drug compounds having side effect “Malaise”

CID000000143 CID000000444 CID000000450 CID000000564 CID000000596
CID000000598 CID000000738 CID000000807 CID000001134 CID000001546
CID000001690 CID000001775 CID000001935 CID000001971 CID000001972
CID000001978 CID000001986 CID000002022 CID000002083 CID000002088
CID000002092 CID000002099 CID000002118 CID000002131 CID000002156
CID000002160 CID000002162 CID000002182 CID000002187 CID000002216
CID000002250 CID000002265 CID000002267 CID000002269 CID000002274
CID000002284 CID000002349 CID000002369 CID000002370 CID000002405
CID000002443 CID000002462 CID000002476 CID000002477 CID000002512
CID000002550 CID000002585 CID000002609 CID000002631 CID000002656
CID000002676 CID000002678 CID000002733 CID000002751 CID000002764
CID000002771 CID000002801 CID000002802 CID000002803 CID000002818
CID000002895 CID000002907 CID000002909 CID000002951 CID000002958
CID000002978 CID000002995 CID000003003 CID000003015 CID000003019
CID000003032 CID000003040 CID000003066 CID000003075 CID000003108
CID000003114 CID000003121 CID000003148 CID000003152 CID000003157
CID000003203 CID000003261 CID000003278 CID000003279 CID000003308
CID000003310 CID000003339 CID000003342 CID000003345 CID000003355
CID000003365 CID000003367 CID000003373 CID000003379 CID000003386
CID000003394 CID000003403 CID000003404 CID000003405 CID000003410
CID000003414 CID000003446 CID000003449 CID000003454 CID000003461
CID000003475 CID000003510 CID000003519 CID000003640 CID000003648
CID000003657 CID000003672 CID000003675 CID000003696 CID000003702
CID000003706 CID000003715 CID000003724 CID000003734 CID000003736
CID000003767 CID000003793 CID000003825 CID000003826 CID000003869
CID000003877 CID000003878 CID000003883 CID000003899 CID000003902
CID000003911 CID000003937 CID000003948 CID000003961 CID000003962

CID000004036 CID000004044 CID000004046 CID000004054 CID000004075
CID000004100 CID000004112 CID000004114 CID000004158 CID000004163
CID000004170 CID000004178 CID000004200 CID000004205 CID000004212
CID000004253 CID000004259 CID000004409 CID000004428 CID000004440
CID000004449 CID000004451 CID000004463 CID000004473 CID000004485
CID000004493 CID000004509 CID000004543 CID000004583 CID000004585
CID000004594 CID000004595 CID000004607 CID000004614 CID000004634
CID000004635 CID000004666 CID000004679 CID000004691 CID000004740
CID000004745 CID000004856 CID000004885 CID000004889 CID000004891
CID000004893 CID000004894 CID000004913 CID000004932 CID000004976
CID000005002 CID000005005 CID000005029 CID000005038 CID000005039
CID000005040 CID000005064 CID000005070 CID000005073 CID000005076
CID000005077 CID000005078 CID000005095 CID000005152 CID000005195
CID000005203 CID000005212 CID000005352 CID000005358 CID000005372
CID000005394 CID000005401 CID000005402 CID000005408 CID000005426
CID000005466 CID000005479 CID000005487 CID000005496 CID000005503
CID000005514 CID000005515 CID000005523 CID000005525 CID000005533
CID000005538 CID000005544 CID000005584 CID000005591 CID000005625
CID000005645 CID000005656 CID000005672 CID000005717 CID000005718
CID000005719 CID000005721 CID000005726 CID000005731 CID000005732
CID000005734 CID000005735 CID000005746 CID000005978 CID000006049
CID000006691 CID000007029 CID000010631 CID000013342 CID000019090
CID000027661 CID000027686 CID000030623 CID000034312 CID000038904
CID000039860 CID000041317 CID000041744 CID000042615 CID000050294
CID000054454 CID000054547 CID000054786 CID000060184 CID000060613
CID000060714 CID000060754 CID000060787 CID000060795 CID000060865
CID000062819 CID000062924 CID000062959 CID000064147 CID000065027
CID000065999 CID000068740 CID000071158 CID000071273 CID000071616
CID000077992 CID000077993 CID000083786 CID000093860 CID000104758
CID000119607 CID000122316 CID000125889 CID000147912 CID000148192
CID000150610 CID000151165 CID000170361 CID000216239 CID000216326
CID000444013 CID000450096 CID003062316 CID003081884 CID004659568
CID004659569 CID005281007 CID005281104 CID005282044 CID005362420
CID006436173

(42) $\mathbb{S}_{42}$: 302 drug compounds having side effect “Allergic reaction”

CID000000085 CID000000232 CID000000271 CID000000444 CID000000450
CID000000453 CID000000598 CID000000738 CID000000750 CID000000772
CID000000807 CID000001071 CID000001690 CID000001972 CID000002021
CID000002083 CID000002130 CID000002141 CID000002162 CID000002171
CID000002179 CID000002187 CID000002232 CID000002250 CID000002266
CID000002267 CID000002269 CID000002344 CID000002349 CID000002369
CID000002375 CID000002435 CID000002441 CID000002462 CID000002474
CID000002476 CID000002477 CID000002478 CID000002520 CID000002524

CID000002554	CID000002576	CID000002578	CID000002609	CID000002610
CID000002617	CID000002631	CID000002637	CID000002650	CID000002654
CID000002662	CID000002666	CID000002675	CID000002676	CID000002678
CID000002708	CID000002726	CID000002756	CID000002762	CID000002764
CID000002769	CID000002771	CID000002786	CID000002800	CID000002802
CID000002803	CID000002806	CID000002895	CID000002909	CID000002958
CID000002973	CID000003003	CID000003015	CID000003016	CID000003032
CID000003040	CID000003042	CID000003075	CID000003105	CID000003108
CID000003121	CID000003143	CID000003152	CID000003154	CID000003157
CID000003203	CID000003255	CID000003261	CID000003285	CID000003291
CID000003308	CID000003310	CID000003339	CID000003345	CID000003365
CID000003366	CID000003372	CID000003373	CID000003379	CID000003381
CID000003384	CID000003385	CID000003386	CID000003393	CID000003394
CID000003410	CID000003446	CID000003454	CID000003461	CID000003467
CID000003475	CID000003488	CID000003494	CID000003510	CID000003640
CID000003648	CID000003652	CID000003658	CID000003676	CID000003690
CID000003702	CID000003724	CID000003734	CID000003736	CID000003741
CID000003793	CID000003823	CID000003825	CID000003826	CID000003827
CID000003878	CID000003883	CID000003899	CID000003911	CID000003928
CID000003948	CID000003954	CID000003956	CID000004043	CID000004053
CID000004054	CID000004064	CID000004075	CID000004078	CID000004158
CID000004159	CID000004168	CID000004173	CID000004192	CID000004196
CID000004200	CID000004201	CID000004212	CID000004236	CID000004253
CID000004259	CID000004264	CID000004419	CID000004440	CID000004449
CID000004451	CID000004463	CID000004473	CID000004485	CID000004510
CID000004542	CID000004583	CID000004585	CID000004594	CID000004595
CID000004599	CID000004607	CID000004609	CID000004635	CID000004679
CID000004691	CID000004725	CID000004736	CID000004739	CID000004819
CID000004885	CID000004891	CID000004893	CID000004894	CID000004914
CID000004915	CID000004917	CID000004919	CID000004920	CID000004932
CID000004934	CID000004991	CID000005029	CID000005073	CID000005076
CID000005077	CID000005095	CID000005155	CID000005195	CID000005203
CID000005206	CID000005210	CID000005212	CID000005215	CID000005245
CID000005320	CID000005344	CID000005372	CID000005379	CID000005394
CID000005396	CID000005401	CID000005408	CID000005426	CID000005452
CID000005453	CID000005454	CID000005466	CID000005472	CID000005478
CID000005486	CID000005487	CID000005496	CID000005512	CID000005514
CID000005523	CID000005533	CID000005538	CID000005544	CID000005566
CID000005593	CID000005625	CID000005636	CID000005650	CID000005656
CID000005672	CID000005718	CID000005721	CID000005731	CID000005732
CID000005734	CID000005735	CID000006691	CID000009433	CID000010631
CID000027661	CID000027991	CID000031378	CID000031477	CID000038904
CID000039042	CID000039860	CID000040976	CID000041693	CID000042615
CID000047319	CID000047725	CID000051263	CID000054547	CID000054688

CID000057537	CID000059708	CID000060613	CID000060787	CID000060795
CID000060843	CID000060852	CID000060877	CID000062819	CID000062867
CID000062959	CID000064147	CID000068740	CID000068844	CID000071158
CID000071273	CID000071616	CID000074989	CID000077993	CID000082146
CID000119607	CID000122316	CID000123631	CID000125889	CID000147912
CID000148192	CID000152945	CID000163742	CID000444013	CID003002190
CID003081884	CID004479097	CID004659568	CID004659569	CID005281104
CID005282044	CID005362420	CID005481350	CID005493444	CID006398970
CID006447131	CID009571074			

(43) $\mathbb{S}_{43}$: 292 drug compounds having side effect "Back pain"

CID000000085	CID000000158	CID000000159	CID000000191	CID000000214
CID000000444	CID000000450	CID000000453	CID000000596	CID000000598
CID000000738	CID000000750	CID000000807	CID000000942	CID000001134
CID000001775	CID000001935	CID000001972	CID000001978	CID000002083
CID000002092	CID000002118	CID000002162	CID000002182	CID000002187
CID000002215	CID000002232	CID000002250	CID000002267	CID000002284
CID000002308	CID000002311	CID000002375	CID000002405	CID000002462
CID000002474	CID000002476	CID000002478	CID000002487	CID000002541
CID000002554	CID000002578	CID000002585	CID000002609	CID000002662
CID000002676	CID000002678	CID000002713	CID000002769	CID000002771
CID000002786	CID000002800	CID000002801	CID000002802	CID000002806
CID000002891	CID000002949	CID000002951	CID000002958	CID000003016
CID000003019	CID000003032	CID000003108	CID000003121	CID000003143
CID000003152	CID000003154	CID000003157	CID000003161	CID000003261
CID000003285	CID000003310	CID000003333	CID000003339	CID000003340
CID000003345	CID000003348	CID000003379	CID000003381	CID000003386
CID000003403	CID000003404	CID000003406	CID000003410	CID000003414
CID000003417	CID000003446	CID000003449	CID000003475	CID000003676
CID000003702	CID000003706	CID000003724	CID000003736	CID000003746
CID000003750	CID000003784	CID000003793	CID000003826	CID000003878
CID000003883	CID000003890	CID000003899	CID000003902	CID000003911
CID000003937	CID000003948	CID000003961	CID000003962	CID000004054
CID000004062	CID000004075	CID000004112	CID000004158	CID000004163
CID000004170	CID000004173	CID000004195	CID000004196	CID000004200
CID000004201	CID000004205	CID000004212	CID000004236	CID000004253
CID000004259	CID000004428	CID000004449	CID000004451	CID000004485
CID000004493	CID000004513	CID000004539	CID000004542	CID000004547
CID000004583	CID000004585	CID000004594	CID000004599	CID000004609
CID000004634	CID000004635	CID000004666	CID000004679	CID000004691
CID000004740	CID000004745	CID000004819	CID000004845	CID000004885
CID000004893	CID000004920	CID000005002	CID000005005	CID000005040
CID000005064	CID000005070	CID000005073	CID000005076	CID000005077
CID000005090	CID000005095	CID000005152	CID000005155	CID000005195

CID000005203	CID000005210	CID000005212	CID000005245	CID000005253
CID000005291	CID000005358	CID000005372	CID000005376	CID000005379
CID000005391	CID000005394	CID000005401	CID000005426	CID000005466
CID000005487	CID000005496	CID000005514	CID000005515	CID000005523
CID000005525	CID000005538	CID000005544	CID000005596	CID000005625
CID000005636	CID000005645	CID000005650	CID000005656	CID000005665
CID000005672	CID000005717	CID000005718	CID000005719	CID000005726
CID000005731	CID000005732	CID000005735	CID000005978	CID000008612
CID000010631	CID000014888	CID000027661	CID000027991	CID000034312
CID000039860	CID000040976	CID000041317	CID000041744	CID000042615
CID000051634	CID000054547	CID000054688	CID000054786	CID000057469
CID000057537	CID000059708	CID000060184	CID000060198	CID000060613
CID000060787	CID000060795	CID000060852	CID000060865	CID000060953
CID000062816	CID000062819	CID000062924	CID000062959	CID000064147
CID000065999	CID000068740	CID000071158	CID000071273	CID000071329
CID000071616	CID000072054	CID000072938	CID000077992	CID000077993
CID000077999	CID000082146	CID000093860	CID000096312	CID000104741
CID000104865	CID000110634	CID000110635	CID000115237	CID000119182
CID000119607	CID000123606	CID000123620	CID000125889	CID000130881
CID000147912	CID000148192	CID000150311	CID000151165	CID000158440
CID000160051	CID000163742	CID000166548	CID000170361	CID000216326
CID000443871	CID000444013	CID000444033	CID000450096	CID000477468
CID003002190	CID003081884	CID004183806	CID004659568	CID005229711
CID005281104	CID005282044	CID005282226	CID005311181	CID005329102
CID005362420	CID005481350	CID005487301	CID005493381	CID005493444
CID006398525	CID006447131			

(44) $\mathbb{S}_{44}$: 296 drug compounds having side effect “Tremor”

CID000000158	CID000000159	CID000000191	CID000000401	CID000000444
CID000000596	CID000000767	CID000000838	CID000000853	CID000000942
CID000001065	CID000001206	CID000001775	CID000001935	CID000001972
CID000002022	CID000002083	CID000002099	CID000002118	CID000002130
CID000002140	CID000002142	CID000002153	CID000002156	CID000002160
CID000002162	CID000002170	CID000002284	CID000002349	CID000002369
CID000002405	CID000002462	CID000002474	CID000002476	CID000002477
CID000002487	CID000002520	CID000002554	CID000002564	CID000002576
CID000002578	CID000002609	CID000002676	CID000002678	CID000002708
CID000002725	CID000002726	CID000002751	CID000002764	CID000002769
CID000002771	CID000002781	CID000002801	CID000002802	CID000002818
CID000002895	CID000002909	CID000002913	CID000002949	CID000002958
CID000002995	CID000003007	CID000003032	CID000003066	CID000003075
CID000003100	CID000003108	CID000003121	CID000003125	CID000003148
CID000003152	CID000003157	CID000003158	CID000003203	CID000003261
CID000003308	CID000003333	CID000003342	CID000003345	CID000003355

CID000003365	CID000003372	CID000003373	CID000003386	CID000003394
CID000003403	CID000003404	CID000003410	CID000003414	CID000003419
CID000003446	CID000003449	CID000003454	CID000003476	CID000003478
CID000003510	CID000003518	CID000003519	CID000003637	CID000003648
CID000003658	CID000003661	CID000003672	CID000003675	CID000003676
CID000003696	CID000003730	CID000003734	CID000003736	CID000003739
CID000003741	CID000003746	CID000003749	CID000003759	CID000003779
CID000003793	CID000003826	CID000003869	CID000003878	CID000003883
CID000003911	CID000003937	CID000003948	CID000003958	CID000003961
CID000003962	CID000003964	CID000004011	CID000004032	CID000004044
CID000004046	CID000004054	CID000004058	CID000004060	CID000004062
CID000004075	CID000004078	CID000004086	CID000004091	CID000004095
CID000004158	CID000004168	CID000004173	CID000004178	CID000004192
CID000004197	CID000004205	CID000004236	CID000004253	CID000004259
CID000004409	CID000004419	CID000004428	CID000004440	CID000004449
CID000004473	CID000004485	CID000004539	CID000004543	CID000004583
CID000004585	CID000004594	CID000004595	CID000004601	CID000004609
CID000004614	CID000004616	CID000004635	CID000004679	CID000004691
CID000004736	CID000004740	CID000004745	CID000004771	CID000004819
CID000004845	CID000004856	CID000004885	CID000004889	CID000004914
CID000004915	CID000004917	CID000004920	CID000004927	CID000004932
CID000004943	CID000004976	CID000005002	CID000005029	CID000005038
CID000005040	CID000005070	CID000005071	CID000005073	CID000005076
CID000005077	CID000005078	CID000005095	CID000005152	CID000005203
CID000005210	CID000005212	CID000005358	CID000005372	CID000005379
CID000005391	CID000005394	CID000005401	CID000005403	CID000005419
CID000005426	CID000005452	CID000005466	CID000005487	CID000005514
CID000005516	CID000005523	CID000005530	CID000005533	CID000005538
CID000005566	CID000005584	CID000005625	CID000005647	CID000005656
CID000005665	CID000005718	CID000005719	CID000005726	CID000005731
CID000005732	CID000005734	CID000005735	CID000006049	CID000006058
CID000007029	CID000008612	CID000009433	CID000010631	CID000014888
CID000016362	CID000023897	CID000027661	CID000028112	CID000034312
CID000039860	CID000042615	CID000050294	CID000051634	CID000054454
CID000054688	CID000056959	CID000057537	CID000059708	CID000060184
CID000060613	CID000060714	CID000060754	CID000060787	CID000060795
CID000060953	CID000062867	CID000062924	CID000062959	CID000064147
CID000068740	CID000071158	CID000071273	CID000071616	CID000072938
CID000077992	CID000077993	CID000096312	CID000104865	CID000115237
CID000119182	CID000119607	CID000122316	CID000123606	CID000125017
CID000125889	CID000147912	CID000150610	CID000151165	CID000158440
CID000170361	CID000216326	CID000443871	CID000444013	CID000450096
CID003062316	CID003081884	CID004659569	CID005282044	CID005311297
CID006435110				

(45) $\mathbb{S}_{45}$: 288 drug compounds having side effect “Nervousness”

CID000000159	CID000000191	CID000000444	CID000000450	CID000000807
CID000000838	CID000000853	CID000000937	CID000001065	CID000001690
CID000001775	CID000001935	CID000001972	CID000002083	CID000002118
CID000002130	CID000002153	CID000002162	CID000002170	CID000002182
CID000002187	CID000002216	CID000002249	CID000002267	CID000002269
CID000002311	CID000002344	CID000002349	CID000002369	CID000002375
CID000002443	CID000002462	CID000002474	CID000002476	CID000002477
CID000002478	CID000002487	CID000002512	CID000002550	CID000002554
CID000002564	CID000002585	CID000002609	CID000002610	CID000002646
CID000002662	CID000002678	CID000002725	CID000002751	CID000002762
CID000002764	CID000002769	CID000002771	CID000002781	CID000002800
CID000002801	CID000002802	CID000002803	CID000002891	CID000002895
CID000002909	CID000002913	CID000002949	CID000002951	CID000002978
CID000003007	CID000003016	CID000003032	CID000003042	CID000003059
CID000003066	CID000003075	CID000003100	CID000003108	CID000003114
CID000003121	CID000003151	CID000003152	CID000003154	CID000003157
CID000003158	CID000003203	CID000003222	CID000003261	CID000003285
CID000003308	CID000003333	CID000003339	CID000003340	CID000003342
CID000003345	CID000003348	CID000003354	CID000003355	CID000003373
CID000003379	CID000003386	CID000003393	CID000003394	CID000003397
CID000003404	CID000003410	CID000003414	CID000003417	CID000003446
CID000003449	CID000003454	CID000003475	CID000003476	CID000003478
CID000003494	CID000003510	CID000003519	CID000003648	CID000003652
CID000003672	CID000003676	CID000003696	CID000003702	CID000003715
CID000003724	CID000003746	CID000003749	CID000003779	CID000003784
CID000003825	CID000003826	CID000003827	CID000003878	CID000003883
CID000003902	CID000003911	CID000003937	CID000003948	CID000003956
CID000003957	CID000003958	CID000003961	CID000004011	CID000004044
CID000004054	CID000004057	CID000004060	CID000004075	CID000004086
CID000004114	CID000004158	CID000004170	CID000004171	CID000004178
CID000004192	CID000004195	CID000004205	CID000004236	CID000004253
CID000004259	CID000004409	CID000004419	CID000004425	CID000004428
CID000004436	CID000004473	CID000004485	CID000004493	CID000004506
CID000004513	CID000004539	CID000004542	CID000004583	CID000004585
CID000004594	CID000004609	CID000004614	CID000004634	CID000004635
CID000004679	CID000004691	CID000004737	CID000004739	CID000004740
CID000004745	CID000004819	CID000004828	CID000004845	CID000004856
CID000004885	CID000004889	CID000004893	CID000004914	CID000004915
CID000004920	CID000004927	CID000004934	CID000005002	CID000005005
CID000005029	CID000005038	CID000005040	CID000005052	CID000005064
CID000005071	CID000005073	CID000005076	CID000005077	CID000005078
CID000005095	CID000005152	CID000005193	CID000005195	CID000005203

CID000005206	CID000005210	CID000005212	CID000005352	CID000005372
CID000005379	CID000005391	CID000005401	CID000005403	CID000005408
CID000005426	CID000005466	CID000005478	CID000005487	CID000005514
CID000005523	CID000005533	CID000005538	CID000005556	CID000005572
CID000005625	CID000005645	CID000005656	CID000005665	CID000005718
CID000005719	CID000005726	CID000005731	CID000005732	CID000005734
CID000005735	CID000006058	CID000006476	CID000007029	CID000009433
CID000010631	CID000020585	CID000027400	CID000027661	CID000027686
CID000031477	CID000034312	CID000039860	CID000040976	CID000041317
CID000041781	CID000042615	CID000054547	CID000054688	CID000054786
CID000059708	CID000060184	CID000060613	CID000060795	CID000062959
CID000064147	CID000065999	CID000071158	CID000071273	CID000072938
CID000077992	CID000077993	CID000119607	CID000123606	CID000125017
CID000125889	CID000148192	CID000150610	CID000444013	CID003002190
CID003081884	CID004659568	CID004659569	CID005229711	CID005281104
CID005282044	CID005311027	CID009571074		

(46) $\mathbb{S}_{46}$: 282 drug compounds having side effect “Hemorrhage”

CID000000085	CID000000137	CID000000158	CID000000159	CID000000214
CID000000444	CID000000450	CID000000596	CID000000772	CID000000807
CID000000838	CID000000937	CID000001003	CID000001065	CID000001125
CID000001546	CID000001775	CID000001935	CID000001972	CID000002088
CID000002099	CID000002156	CID000002179	CID000002182	CID000002187
CID000002232	CID000002250	CID000002265	CID000002349	CID000002369
CID000002375	CID000002435	CID000002462	CID000002477	CID000002478
CID000002519	CID000002541	CID000002578	CID000002585	CID000002609
CID000002610	CID000002617	CID000002622	CID000002631	CID000002637
CID000002646	CID000002650	CID000002654	CID000002655	CID000002656
CID000002658	CID000002666	CID000002675	CID000002678	CID000002713
CID000002764	CID000002801	CID000002802	CID000002806	CID000002812
CID000002907	CID000002909	CID000002949	CID000002951	CID000003003
CID000003009	CID000003015	CID000003032	CID000003075	CID000003108
CID000003121	CID000003143	CID000003152	CID000003285	CID000003308
CID000003325	CID000003340	CID000003342	CID000003345	CID000003365
CID000003367	CID000003381	CID000003385	CID000003386	CID000003387
CID000003394	CID000003410	CID000003446	CID000003449	CID000003454
CID000003461	CID000003475	CID000003640	CID000003648	CID000003657
CID000003676	CID000003685	CID000003690	CID000003698	CID000003706
CID000003715	CID000003734	CID000003736	CID000003741	CID000003746
CID000003750	CID000003826	CID000003878	CID000003902	CID000003911
CID000003929	CID000003956	CID000003958	CID000003998	CID000004033
CID000004044	CID000004053	CID000004054	CID000004112	CID000004159
CID000004160	CID000004196	CID000004212	CID000004259	CID000004264
CID000004409	CID000004428	CID000004440	CID000004449	CID000004485

CID000004536 CID000004542 CID000004583 CID000004585 CID000004603
CID000004609 CID000004614 CID000004635 CID000004638 CID000004666
CID000004679 CID000004727 CID000004739 CID000004740 CID000004745
CID000004819 CID000004834 CID000004856 CID000004865 CID000004885
CID000004893 CID000004894 CID000004900 CID000004915 CID000004920
CID000004932 CID000004935 CID000004943 CID000005029 CID000005040
CID000005064 CID000005070 CID000005073 CID000005076 CID000005077
CID000005095 CID000005195 CID000005203 CID000005206 CID000005210
CID000005212 CID000005245 CID000005253 CID000005267 CID000005291
CID000005358 CID000005359 CID000005372 CID000005376 CID000005379
CID000005381 CID000005394 CID000005396 CID000005408 CID000005453
CID000005466 CID000005472 CID000005486 CID000005487 CID000005504
CID000005514 CID000005515 CID000005523 CID000005538 CID000005544
CID000005636 CID000005645 CID000005656 CID000005717 CID000005718
CID000005719 CID000005721 CID000005732 CID000006058 CID000006691
CID000009904 CID000010631 CID000013342 CID000014888 CID000019090
CID000020585 CID000027661 CID000030623 CID000031378 CID000034312
CID000038904 CID000040976 CID000041317 CID000041781 CID000042113
CID000047725 CID000050614 CID000051634 CID000054547 CID000054786
CID000057469 CID000060184 CID000060198 CID000060612 CID000060787
CID000060795 CID000060852 CID000060953 CID000062816 CID000062819
CID000062959 CID000064147 CID000071158 CID000071273 CID000071616
CID000072938 CID000077992 CID000077993 CID000082146 CID000083786
CID000093860 CID000104741 CID000104865 CID000110634 CID000119607
CID000122316 CID000123631 CID000125017 CID000125889 CID000145068
CID000147912 CID000150310 CID000150610 CID000151165 CID000158440
CID000176870 CID000197712 CID000216239 CID000216326 CID000222786
CID000444013 CID000444033 CID000477468 CID000657298 CID003062316
CID003081884 CID003086672 CID004659568 CID004659569 CID005281104
CID005282226 CID005329102 CID005353894 CID005362420 CID005493444
CID006398970 CID009571074

(47) $\mathbb{S}_{47}$: 284 drug compounds having side effect “Arrhythmia”

CID000000085 CID000000158 CID000000159 CID000000191 CID000000206
CID000000444 CID000000598 CID000000681 CID000000767 CID000000807
CID000000838 CID000000861 CID000000937 CID000000951 CID000001546
CID000001690 CID000001972 CID000002083 CID000002092 CID000002099
CID000002130 CID000002140 CID000002141 CID000002153 CID000002160
CID000002162 CID000002170 CID000002179 CID000002182 CID000002216
CID000002249 CID000002250 CID000002269 CID000002284 CID000002311
CID000002349 CID000002369 CID000002375 CID000002405 CID000002435
CID000002443 CID000002474 CID000002478 CID000002487 CID000002512
CID000002524 CID000002554 CID000002578 CID000002583 CID000002631
CID000002678 CID000002751 CID000002764 CID000002771 CID000002800

CID000002801	CID000002803	CID000002818	CID000002895	CID000002907
CID000002909	CID000002958	CID000002995	CID000003003	CID000003007
CID000003015	CID000003019	CID000003032	CID000003062	CID000003105
CID000003108	CID000003121	CID000003143	CID000003148	CID000003152
CID000003154	CID000003156	CID000003157	CID000003203	CID000003226
CID000003255	CID000003261	CID000003308	CID000003325	CID000003333
CID000003339	CID000003345	CID000003367	CID000003372	CID000003373
CID000003385	CID000003386	CID000003394	CID000003404	CID000003410
CID000003414	CID000003419	CID000003449	CID000003454	CID000003461
CID000003478	CID000003510	CID000003559	CID000003562	CID000003640
CID000003648	CID000003661	CID000003672	CID000003690	CID000003696
CID000003698	CID000003702	CID000003715	CID000003724	CID000003734
CID000003736	CID000003737	CID000003741	CID000003742	CID000003750
CID000003763	CID000003779	CID000003793	CID000003821	CID000003823
CID000003825	CID000003826	CID000003869	CID000003883	CID000003911
CID000003914	CID000003937	CID000003948	CID000003958	CID000003961
CID000004011	CID000004044	CID000004053	CID000004062	CID000004064
CID000004078	CID000004095	CID000004114	CID000004158	CID000004159
CID000004171	CID000004205	CID000004212	CID000004236	CID000004259
CID000004409	CID000004485	CID000004539	CID000004542	CID000004547
CID000004583	CID000004594	CID000004595	CID000004603	CID000004609
CID000004614	CID000004634	CID000004666	CID000004679	CID000004691
CID000004739	CID000004740	CID000004745	CID000004775	CID000004819
CID000004828	CID000004856	CID000004885	CID000004891	CID000004894
CID000004914	CID000004915	CID000004943	CID000004976	CID000005005
CID000005029	CID000005038	CID000005039	CID000005052	CID000005064
CID000005073	CID000005077	CID000005078	CID000005095	CID000005152
CID000005195	CID000005203	CID000005206	CID000005212	CID000005234
CID000005245	CID000005314	CID000005352	CID000005358	CID000005372
CID000005376	CID000005396	CID000005401	CID000005426	CID000005452
CID000005478	CID000005487	CID000005504	CID000005514	CID000005516
CID000005523	CID000005525	CID000005533	CID000005538	CID000005544
CID000005584	CID000005645	CID000005656	CID000005672	CID000005718
CID000005721	CID000005731	CID000005732	CID000005771	CID000005775
CID000005978	CID000007029	CID000008612	CID000009433	CID000014888
CID000020585	CID000027661	CID000027991	CID000028112	CID000036339
CID000039860	CID000041693	CID000042113	CID000047725	CID000051263
CID000054688	CID000057469	CID000057537	CID000060184	CID000060612
CID000060754	CID000060843	CID000060953	CID000064147	CID000065999
CID000071273	CID000071616	CID000077993	CID000082146	CID000093860
CID000104758	CID000104865	CID000110634	CID000110635	CID000115237
CID000119607	CID000122316	CID000123631	CID000125889	CID000147912
CID000148211	CID000150610	CID000166548	CID000170361	CID000176870
CID000216239	CID000216326	CID000444013	CID000477468	CID003002190

CID003062316 CID003081884 CID004183806 CID004659569 CID005281007
CID005281104 CID005282044 CID005329102 CID005487301

(48) $\mathbb{S}_{48}$: 276 drug compounds having side effect "Tinnitus"

CID000000444 CID000000450 CID000000564 CID000000767 CID000000807
CID000000815 CID000000937 CID000001065 CID000001690 CID000001775
CID000001935 CID000001972 CID000001978 CID000001986 CID000002083
CID000002118 CID000002156 CID000002160 CID000002162 CID000002170
CID000002182 CID000002244 CID000002249 CID000002250 CID000002269
CID000002274 CID000002284 CID000002311 CID000002349 CID000002369
CID000002405 CID000002474 CID000002476 CID000002477 CID000002487
CID000002520 CID000002541 CID000002554 CID000002564 CID000002585
CID000002662 CID000002678 CID000002719 CID000002725 CID000002751
CID000002762 CID000002764 CID000002771 CID000002781 CID000002800
CID000002801 CID000002803 CID000002895 CID000002909 CID000002913
CID000002958 CID000002973 CID000002978 CID000002983 CID000002995
CID000003032 CID000003059 CID000003066 CID000003075 CID000003100
CID000003108 CID000003121 CID000003148 CID000003152 CID000003154
CID000003157 CID000003158 CID000003203 CID000003222 CID000003255
CID000003261 CID000003278 CID000003308 CID000003324 CID000003325
CID000003342 CID000003345 CID000003355 CID000003373 CID000003386
CID000003394 CID000003404 CID000003419 CID000003440 CID000003446
CID000003449 CID000003454 CID000003467 CID000003475 CID000003519
CID000003648 CID000003652 CID000003672 CID000003676 CID000003696
CID000003702 CID000003715 CID000003724 CID000003741 CID000003746
CID000003793 CID000003825 CID000003826 CID000003878 CID000003883
CID000003911 CID000003928 CID000003929 CID000003937 CID000003948
CID000003961 CID000004011 CID000004033 CID000004036 CID000004044
CID000004046 CID000004054 CID000004062 CID000004075 CID000004100
CID000004112 CID000004140 CID000004170 CID000004171 CID000004178
CID000004200 CID000004205 CID000004259 CID000004409 CID000004411
CID000004428 CID000004440 CID000004449 CID000004473 CID000004485
CID000004539 CID000004543 CID000004583 CID000004585 CID000004594
CID000004614 CID000004634 CID000004635 CID000004666 CID000004679
CID000004691 CID000004727 CID000004736 CID000004739 CID000004745
CID000004819 CID000004828 CID000004856 CID000004885 CID000004889
CID000004893 CID000004914 CID000004920 CID000004927 CID000004932
CID000004943 CID000004946 CID000004976 CID000005002 CID000005005
CID000005029 CID000005038 CID000005040 CID000005064 CID000005071
CID000005073 CID000005076 CID000005077 CID000005078 CID000005090
CID000005095 CID000005195 CID000005203 CID000005210 CID000005212
CID000005215 CID000005245 CID000005291 CID000005344 CID000005352
CID000005358 CID000005372 CID000005379 CID000005394 CID000005401
CID000005426 CID000005430 CID000005466 CID000005472 CID000005478

CID000005487 CID000005496 CID000005508 CID000005514 CID000005523
CID000005525 CID000005530 CID000005533 CID000005538 CID000005556
CID000005584 CID000005625 CID000005645 CID000005651 CID000005656
CID000005718 CID000005719 CID000005731 CID000005732 CID000005734
CID000005735 CID000008612 CID000010631 CID000014888 CID000018140
CID000027661 CID000027686 CID000028112 CID000034312 CID000038904
CID000039860 CID000041317 CID000042615 CID000054547 CID000054688
CID000056959 CID000057537 CID000060184 CID000060613 CID000060714
CID000060754 CID000060787 CID000060795 CID000062959 CID000064147
CID000065999 CID000071158 CID000071273 CID000071616 CID000077992
CID000077993 CID000083786 CID000104865 CID000110634 CID000110635
CID000119607 CID000123606 CID000125017 CID000125889 CID000147912
CID000148192 CID000148211 CID000151165 CID000158440 CID000170361
CID000216239 CID000216326 CID000444013 CID003062316 CID003081884
CID004183806 CID004659569 CID005229711 CID005353980 CID006436173
CID011947681

(49) $\mathbb{S}_{49}$: 273 drug compounds having side effect “Anaphylaxis”

CID000000119 CID000000298 CID000000450 CID000000564 CID000000596
CID000000807 CID000000937 CID000001003 CID000001071 CID000001690
CID000001971 CID000001972 CID000001986 CID000002021 CID000002022
CID000002083 CID000002130 CID000002140 CID000002148 CID000002171
CID000002173 CID000002187 CID000002244 CID000002250 CID000002269
CID000002274 CID000002308 CID000002315 CID000002349 CID000002366
CID000002441 CID000002462 CID000002474 CID000002476 CID000002520
CID000002524 CID000002554 CID000002585 CID000002609 CID000002610
CID000002617 CID000002622 CID000002631 CID000002637 CID000002646
CID000002650 CID000002654 CID000002655 CID000002656 CID000002658
CID000002662 CID000002666 CID000002675 CID000002676 CID000002678
CID000002713 CID000002720 CID000002733 CID000002756 CID000002762
CID000002764 CID000002771 CID000002781 CID000002802 CID000002891
CID000002895 CID000002907 CID000002951 CID000002973 CID000002983
CID000003003 CID000003007 CID000003016 CID000003032 CID000003040
CID000003042 CID000003059 CID000003121 CID000003143 CID000003148
CID000003154 CID000003168 CID000003255 CID000003285 CID000003308
CID000003325 CID000003342 CID000003345 CID000003348 CID000003365
CID000003367 CID000003385 CID000003394 CID000003403 CID000003404
CID000003405 CID000003410 CID000003417 CID000003446 CID000003454
CID000003463 CID000003494 CID000003510 CID000003636 CID000003639
CID000003640 CID000003661 CID000003672 CID000003676 CID000003685
CID000003702 CID000003715 CID000003724 CID000003736 CID000003737
CID000003741 CID000003742 CID000003746 CID000003793 CID000003821
CID000003823 CID000003825 CID000003826 CID000003877 CID000003883
CID000003899 CID000003911 CID000003928 CID000003929 CID000003948

CID000003954 CID000003956 CID000003957 CID000003961 CID000003962
CID000004033 CID000004046 CID000004053 CID000004057 CID000004064
CID000004107 CID000004112 CID000004121 CID000004140 CID000004158
CID000004170 CID000004192 CID000004197 CID000004200 CID000004212
CID000004236 CID000004253 CID000004259 CID000004409 CID000004421
CID000004440 CID000004449 CID000004463 CID000004485 CID000004509
CID000004513 CID000004539 CID000004583 CID000004594 CID000004595
CID000004599 CID000004601 CID000004607 CID000004609 CID000004614
CID000004635 CID000004645 CID000004679 CID000004691 CID000004730
CID000004856 CID000004889 CID000004894 CID000004911 CID000004920
CID000004934 CID000004943 CID000004993 CID000005002 CID000005029
CID000005039 CID000005073 CID000005078 CID000005152 CID000005210
CID000005267 CID000005297 CID000005314 CID000005344 CID000005352
CID000005358 CID000005379 CID000005394 CID000005401 CID000005412
CID000005430 CID000005453 CID000005454 CID000005472 CID000005478
CID000005486 CID000005487 CID000005523 CID000005544 CID000005546
CID000005578 CID000005596 CID000005647 CID000005650 CID000005651
CID000005656 CID000005672 CID000005726 CID000005731 CID000005732
CID000005735 CID000005771 CID000005978 CID000006691 CID000009034
CID000010631 CID000016850 CID000027686 CID000027991 CID000034312
CID000038904 CID000041693 CID000047725 CID000050294 CID000050614
CID000051263 CID000054454 CID000054547 CID000054688 CID000060184
CID000060795 CID000062924 CID000062959 CID000065999 CID000068740
CID000071273 CID000072938 CID000077992 CID000077999 CID000093860
CID000104865 CID000110634 CID000115237 CID000119607 CID000123620
CID000124087 CID000130881 CID000150311 CID000150610 CID000151165
CID000477468 CID003002190 CID004183806 CID004479097 CID005281007
CID005282044 CID005311297 CID005353980 CID005381226 CID005493381
CID006398970 CID006447131 CID009571074

(50) $\mathbb{S}_{50}$: 276 drug compounds having side effect “Blurred vision”

CID000000158 CID000000191 CID000000444 CID000000453 CID000000596
CID000000767 CID000001065 CID000001972 CID000001978 CID000002118
CID000002156 CID000002160 CID000002170 CID000002179 CID000002182
CID000002216 CID000002249 CID000002267 CID000002284 CID000002315
CID000002344 CID000002349 CID000002369 CID000002435 CID000002441
CID000002462 CID000002474 CID000002476 CID000002477 CID000002487
CID000002520 CID000002550 CID000002554 CID000002564 CID000002578
CID000002585 CID000002662 CID000002676 CID000002719 CID000002720
CID000002725 CID000002764 CID000002771 CID000002781 CID000002800
CID000002802 CID000002803 CID000002895 CID000002905 CID000002907
CID000002913 CID000002973 CID000002995 CID000003016 CID000003019
CID000003032 CID000003042 CID000003059 CID000003075 CID000003100
CID000003114 CID000003121 CID000003152 CID000003154 CID000003157

CID000003158 CID000003203 CID000003219 CID000003222 CID000003278
CID000003279 CID000003308 CID000003342 CID000003345 CID000003348
CID000003354 CID000003355 CID000003372 CID000003373 CID000003379
CID000003381 CID000003384 CID000003385 CID000003393 CID000003404
CID000003406 CID000003440 CID000003446 CID000003449 CID000003463
CID000003476 CID000003478 CID000003488 CID000003494 CID000003518
CID000003519 CID000003559 CID000003639 CID000003648 CID000003652
CID000003661 CID000003672 CID000003675 CID000003676 CID000003690
CID000003696 CID000003702 CID000003715 CID000003736 CID000003741
CID000003746 CID000003793 CID000003821 CID000003826 CID000003878
CID000003883 CID000003890 CID000003899 CID000003902 CID000003911
CID000003929 CID000003937 CID000003958 CID000003961 CID000003962
CID000003964 CID000004011 CID000004032 CID000004034 CID000004036
CID000004043 CID000004044 CID000004054 CID000004057 CID000004062
CID000004075 CID000004078 CID000004086 CID000004091 CID000004107
CID000004112 CID000004121 CID000004158 CID000004170 CID000004171
CID000004178 CID000004192 CID000004200 CID000004212 CID000004253
CID000004411 CID000004419 CID000004440 CID000004449 CID000004473
CID000004485 CID000004493 CID000004506 CID000004510 CID000004539
CID000004543 CID000004583 CID000004594 CID000004595 CID000004601
CID000004614 CID000004616 CID000004666 CID000004679 CID000004691
CID000004736 CID000004740 CID000004748 CID000004819 CID000004856
CID000004885 CID000004889 CID000004893 CID000004894 CID000004914
CID000004917 CID000004919 CID000004920 CID000004927 CID000004932
CID000004934 CID000004976 CID000005002 CID000005029 CID000005039
CID000005064 CID000005076 CID000005077 CID000005078 CID000005090
CID000005095 CID000005195 CID000005210 CID000005212 CID000005291
CID000005352 CID000005379 CID000005391 CID000005394 CID000005401
CID000005430 CID000005452 CID000005453 CID000005454 CID000005478
CID000005487 CID000005514 CID000005516 CID000005523 CID000005530
CID000005533 CID000005538 CID000005566 CID000005572 CID000005584
CID000005593 CID000005596 CID000005625 CID000005650 CID000005656
CID000005718 CID000005732 CID000005746 CID000006476 CID000007029
CID000008612 CID000014888 CID000016362 CID000023897 CID000027661
CID000028112 CID000031477 CID000039507 CID000041317 CID000042615
CID000044564 CID000051263 CID000056959 CID000059708 CID000060795
CID000060865 CID000062867 CID000062924 CID000064147 CID000068740
CID000068844 CID000071273 CID000071616 CID000093860 CID000096312
CID000104865 CID000110634 CID000110635 CID000115237 CID000119607
CID000125017 CID000125889 CID000130881 CID000147912 CID000166548
CID000170361 CID000216326 CID002761171 CID003002190 CID004659569
CID005229711 CID005281007 CID005282226 CID005311027 CID005353894
CID005362420

(51) $\mathbb{S}_{51}$: 268 drug compounds having side effect "Flatulence"

CID000000159	CID000000444	CID000000450	CID000000598	CID000000738
CID000000767	CID000000937	CID000000942	CID000001690	CID000001775
CID000001935	CID000001972	CID000001978	CID000002022	CID000002083
CID000002162	CID000002170	CID000002182	CID000002250	CID000002269
CID000002284	CID000002311	CID000002375	CID000002462	CID000002476
CID000002477	CID000002487	CID000002512	CID000002559	CID000002575
CID000002585	CID000002609	CID000002654	CID000002656	CID000002658
CID000002662	CID000002675	CID000002676	CID000002678	CID000002751
CID000002764	CID000002769	CID000002771	CID000002786	CID000002801
CID000002802	CID000002806	CID000002895	CID000002909	CID000003032
CID000003043	CID000003059	CID000003075	CID000003108	CID000003114
CID000003121	CID000003148	CID000003152	CID000003157	CID000003261
CID000003285	CID000003308	CID000003324	CID000003333	CID000003339
CID000003342	CID000003345	CID000003348	CID000003355	CID000003365
CID000003386	CID000003394	CID000003403	CID000003404	CID000003405
CID000003414	CID000003417	CID000003419	CID000003446	CID000003449
CID000003454	CID000003463	CID000003475	CID000003478	CID000003510
CID000003639	CID000003648	CID000003672	CID000003702	CID000003715
CID000003730	CID000003749	CID000003750	CID000003793	CID000003825
CID000003826	CID000003878	CID000003883	CID000003899	CID000003911
CID000003929	CID000003937	CID000003948	CID000003954	CID000003961
CID000003962	CID000004036	CID000004044	CID000004075	CID000004091
CID000004138	CID000004171	CID000004173	CID000004195	CID000004205
CID000004236	CID000004253	CID000004259	CID000004409	CID000004411
CID000004428	CID000004449	CID000004451	CID000004485	CID000004509
CID000004513	CID000004539	CID000004542	CID000004583	CID000004585
CID000004599	CID000004609	CID000004614	CID000004634	CID000004635
CID000004679	CID000004691	CID000004739	CID000004740	CID000004745
CID000004819	CID000004828	CID000004856	CID000004873	CID000004885
CID000004889	CID000004893	CID000004920	CID000004932	CID000004991
CID000005002	CID000005005	CID000005029	CID000005035	CID000005040
CID000005070	CID000005073	CID000005076	CID000005077	CID000005078
CID000005090	CID000005095	CID000005155	CID000005195	CID000005203
CID000005210	CID000005212	CID000005245	CID000005253	CID000005291
CID000005344	CID000005352	CID000005358	CID000005372	CID000005379
CID000005401	CID000005402	CID000005426	CID000005466	CID000005472
CID000005487	CID000005508	CID000005512	CID000005514	CID000005523
CID000005525	CID000005533	CID000005538	CID000005596	CID000005625
CID000005645	CID000005647	CID000005650	CID000005656	CID000005718
CID000005719	CID000005726	CID000005732	CID000005734	CID000006691
CID000018140	CID000019090	CID000027661	CID000027686	CID000027991
CID000028112	CID000034312	CID000039042	CID000040976	CID000041744
CID000041774	CID000042615	CID000047725	CID000051577	CID000051634

CID000054454	CID000054547	CID000054688	CID000060184	CID000060613
CID000060787	CID000060795	CID000060871	CID000062816	CID000062959
CID000064147	CID000065027	CID000065999	CID000071158	CID000071616
CID000072054	CID000072938	CID000077992	CID000077993	CID000082146
CID000104865	CID000119607	CID000123620	CID000125889	CID000147912
CID000148192	CID000148211	CID000150610	CID000151165	CID000158440
CID000160051	CID000170361	CID000176870	CID000213039	CID000216326
CID000444013	CID003002190	CID003081884	CID004183806	CID004659568
CID004659569	CID005282044	CID005329102	CID005361912	CID005362070
CID005381226	CID005481350	CID005487301	CID006398525	CID006398970
CID006436173	CID006918453	CID009571074		

(52) $\mathbb{S}_{52}$: 266 drug compounds having side effect "Flushing"

CID000000158	CID000000159	CID000000187	CID000000191	CID000000214
CID000000232	CID000000444	CID000000581	CID000000598	CID000000807
CID000000853	CID000000888	CID000000937	CID000000942	CID000001065
CID000001935	CID000001972	CID000001978	CID000001986	CID000002083
CID000002092	CID000002140	CID000002141	CID000002148	CID000002156
CID000002162	CID000002187	CID000002215	CID000002249	CID000002267
CID000002274	CID000002284	CID000002311	CID000002349	CID000002369
CID000002370	CID000002375	CID000002405	CID000002431	CID000002462
CID000002476	CID000002478	CID000002512	CID000002520	CID000002541
CID000002550	CID000002551	CID000002554	CID000002576	CID000002578
CID000002637	CID000002656	CID000002662	CID000002676	CID000002678
CID000002751	CID000002764	CID000002771	CID000002800	CID000002801
CID000002907	CID000002909	CID000002949	CID000002958	CID000002978
CID000002995	CID000003007	CID000003019	CID000003032	CID000003059
CID000003066	CID000003075	CID000003108	CID000003143	CID000003148
CID000003151	CID000003156	CID000003157	CID000003158	CID000003203
CID000003222	CID000003261	CID000003308	CID000003310	CID000003325
CID000003333	CID000003340	CID000003345	CID000003348	CID000003355
CID000003373	CID000003386	CID000003393	CID000003403	CID000003406
CID000003414	CID000003419	CID000003478	CID000003636	CID000003637
CID000003648	CID000003676	CID000003696	CID000003702	CID000003715
CID000003724	CID000003734	CID000003737	CID000003739	CID000003741
CID000003742	CID000003746	CID000003749	CID000003750	CID000003779
CID000003784	CID000003793	CID000003826	CID000003869	CID000003878
CID000003902	CID000003911	CID000003937	CID000003948	CID000003961
CID000003962	CID000004011	CID000004046	CID000004053	CID000004058
CID000004091	CID000004095	CID000004107	CID000004158	CID000004163
CID000004168	CID000004171	CID000004173	CID000004195	CID000004211
CID000004253	CID000004419	CID000004425	CID000004428	CID000004449
CID000004473	CID000004485	CID000004493	CID000004497	CID000004510
CID000004543	CID000004583	CID000004594	CID000004595	CID000004609

CID000004675	CID000004736	CID000004740	CID000004812	CID000004819
CID000004828	CID000004845	CID000004889	CID000004893	CID000004911
CID000004913	CID000004915	CID000004920	CID000004927	CID000004932
CID000004943	CID000004946	CID000004976	CID000005035	CID000005038
CID000005064	CID000005070	CID000005073	CID000005077	CID000005078
CID000005090	CID000005095	CID000005212	CID000005291	CID000005344
CID000005352	CID000005358	CID000005372	CID000005376	CID000005394
CID000005396	CID000005408	CID000005426	CID000005430	CID000005479
CID000005504	CID000005512	CID000005514	CID000005523	CID000005525
CID000005538	CID000005584	CID000005596	CID000005651	CID000005672
CID000005718	CID000005732	CID000005775	CID000014888	CID000018140
CID000020585	CID000027661	CID000027991	CID000032800	CID000034312
CID000039860	CID000040976	CID000041317	CID000042615	CID000047319
CID000047320	CID000054454	CID000059768	CID000060184	CID000060198
CID000060754	CID000060787	CID000060795	CID000060953	CID000062959
CID000065999	CID000068740	CID000072938	CID000077992	CID000077993
CID000093860	CID000104741	CID000104865	CID000110634	CID000110635
CID000119182	CID000119607	CID000125017	CID000147912	CID000150610
CID000151165	CID000166548	CID000197712	CID000216239	CID000216326
CID000450096	CID000477468	CID003002190	CID003062316	CID003081884
CID003086672	CID004659568	CID005229711	CID005281007	CID005311181
CID005362420	CID005381226	CID005487301	CID006436173	CID006447131
CID011947681				

(53) $\mathbb{S}_{53}$: 259 drug compounds having side effect “Pharyngitis”

CID000000085	CID000000158	CID000000159	CID000000214	CID000000444
CID000000450	CID000000581	CID000000598	CID000000738	CID000000942
CID000001134	CID000001690	CID000001775	CID000001935	CID000001971
CID000001972	CID000001978	CID000002019	CID000002083	CID000002092
CID000002182	CID000002187	CID000002216	CID000002250	CID000002267
CID000002308	CID000002311	CID000002369	CID000002375	CID000002405
CID000002435	CID000002462	CID000002476	CID000002478	CID000002487
CID000002520	CID000002541	CID000002550	CID000002554	CID000002585
CID000002662	CID000002676	CID000002678	CID000002713	CID000002751
CID000002764	CID000002769	CID000002771	CID000002786	CID000002801
CID000002802	CID000002895	CID000002909	CID000002955	CID000003032
CID000003066	CID000003075	CID000003108	CID000003121	CID000003143
CID000003152	CID000003154	CID000003157	CID000003241	CID000003261
CID000003285	CID000003308	CID000003333	CID000003339	CID000003345
CID000003367	CID000003379	CID000003386	CID000003394	CID000003403
CID000003404	CID000003406	CID000003410	CID000003414	CID000003417
CID000003419	CID000003446	CID000003461	CID000003475	CID000003478
CID000003648	CID000003672	CID000003702	CID000003706	CID000003724
CID000003736	CID000003746	CID000003749	CID000003793	CID000003825

CID000003827	CID000003878	CID000003883	CID000003899	CID000003911
CID000003915	CID000003929	CID000003937	CID000003948	CID000003961
CID000004075	CID000004112	CID000004158	CID000004173	CID000004178
CID000004205	CID000004212	CID000004236	CID000004259	CID000004264
CID000004428	CID000004449	CID000004451	CID000004485	CID000004510
CID000004513	CID000004542	CID000004583	CID000004585	CID000004594
CID000004609	CID000004634	CID000004635	CID000004679	CID000004691
CID000004739	CID000004745	CID000004819	CID000004829	CID000004885
CID000004889	CID000004920	CID000004943	CID000004946	CID000005002
CID000005005	CID000005035	CID000005040	CID000005064	CID000005073
CID000005076	CID000005077	CID000005078	CID000005090	CID000005095
CID000005152	CID000005155	CID000005195	CID000005203	CID000005206
CID000005210	CID000005212	CID000005245	CID000005291	CID000005372
CID000005376	CID000005379	CID000005394	CID000005401	CID000005426
CID000005466	CID000005479	CID000005487	CID000005496	CID000005514
CID000005515	CID000005523	CID000005525	CID000005538	CID000005544
CID000005625	CID000005636	CID000005645	CID000005650	CID000005656
CID000005718	CID000005731	CID000005732	CID000005734	CID000005735
CID000010631	CID000012536	CID000013342	CID000019090	CID000025419
CID000027661	CID000027686	CID000032797	CID000034312	CID000039042
CID000039507	CID000039860	CID000040976	CID000041317	CID000042113
CID000047725	CID000050294	CID000054786	CID000057469	CID000059708
CID000059768	CID000060184	CID000060198	CID000060613	CID000060787
CID000060795	CID000060843	CID000060852	CID000060865	CID000060871
CID000060953	CID000062924	CID000064147	CID000065999	CID000068844
CID000071158	CID000071616	CID000072054	CID000072938	CID000077992
CID000077993	CID000082146	CID000083786	CID000104741	CID000110634
CID000110635	CID000119607	CID000123606	CID000123620	CID000124087
CID000125889	CID000130881	CID000147912	CID000150311	CID000150610
CID000151165	CID000160051	CID000197712	CID000216326	CID000444013
CID000444033	CID003081884	CID004659568	CID004659569	CID005281104
CID005282044	CID005311027	CID005311297	CID005362070	CID005362420
CID005487301	CID005493381	CID006436173	CID006447131	

(54) $\mathbb{S}_{54}$: 262 drug compounds having side effect “Angioedema”

CID000000119	CID000000298	CID000000444	CID000000450	CID000000581
CID000000767	CID000000772	CID000000807	CID000000853	CID000000937
CID000001065	CID000001972	CID000002022	CID000002083	CID000002088
CID000002092	CID000002140	CID000002148	CID000002156	CID000002162
CID000002177	CID000002187	CID000002244	CID000002250	CID000002269
CID000002274	CID000002308	CID000002349	CID000002366	CID000002375
CID000002405	CID000002462	CID000002474	CID000002476	CID000002477
CID000002520	CID000002541	CID000002554	CID000002576	CID000002585
CID000002609	CID000002610	CID000002631	CID000002637	CID000002646

CID000002650	CID000002658	CID000002662	CID000002666	CID000002675
CID000002676	CID000002678	CID000002708	CID000002726	CID000002733
CID000002762	CID000002764	CID000002769	CID000002771	CID000002803
CID000002806	CID000002895	CID000002909	CID000002973	CID000002983
CID000003003	CID000003007	CID000003032	CID000003040	CID000003059
CID000003075	CID000003108	CID000003114	CID000003148	CID000003154
CID000003285	CID000003305	CID000003308	CID000003325	CID000003333
CID000003342	CID000003348	CID000003365	CID000003372	CID000003386
CID000003394	CID000003403	CID000003404	CID000003410	CID000003417
CID000003446	CID000003463	CID000003488	CID000003512	CID000003636
CID000003640	CID000003652	CID000003672	CID000003676	CID000003702
CID000003715	CID000003736	CID000003737	CID000003741	CID000003742
CID000003746	CID000003749	CID000003784	CID000003793	CID000003826
CID000003869	CID000003878	CID000003899	CID000003928	CID000003929
CID000003948	CID000003954	CID000003961	CID000003962	CID000004030
CID000004044	CID000004062	CID000004064	CID000004075	CID000004078
CID000004107	CID000004158	CID000004168	CID000004173	CID000004189
CID000004200	CID000004236	CID000004259	CID000004409	CID000004421
CID000004428	CID000004449	CID000004463	CID000004485	CID000004509
CID000004539	CID000004542	CID000004583	CID000004585	CID000004594
CID000004595	CID000004599	CID000004607	CID000004609	CID000004614
CID000004645	CID000004679	CID000004691	CID000004737	CID000004740
CID000004748	CID000004856	CID000004889	CID000004894	CID000004913
CID000004914	CID000004917	CID000004920	CID000004943	CID000005002
CID000005029	CID000005039	CID000005040	CID000005070	CID000005073
CID000005078	CID000005090	CID000005095	CID000005152	CID000005193
CID000005203	CID000005210	CID000005245	CID000005291	CID000005297
CID000005344	CID000005352	CID000005358	CID000005372	CID000005376
CID000005379	CID000005402	CID000005412	CID000005426	CID000005430
CID000005452	CID000005472	CID000005478	CID000005479	CID000005512
CID000005515	CID000005523	CID000005544	CID000005566	CID000005596
CID000005625	CID000005647	CID000005650	CID000005665	CID000005672
CID000005717	CID000005726	CID000005731	CID000005732	CID000005735
CID000008612	CID000010631	CID000018140	CID000020585	CID000027686
CID000028112	CID000034312	CID000041317	CID000041781	CID000054454
CID000057469	CID000060613	CID000060795	CID000062867	CID000062924
CID000062959	CID000065999	CID000068740	CID000071273	CID000071301
CID000071329	CID000071616	CID000072054	CID000074989	CID000077999
CID000093860	CID000104741	CID000104865	CID000119607	CID000123620
CID000123631	CID000125889	CID000130881	CID000148192	CID000150310
CID000150311	CID000151165	CID000152945	CID000166548	CID000170361
CID000197712	CID000213039	CID000444033	CID003002190	CID003081884
CID005311297	CID005493381	CID005493444	CID006398525	CID006436173
CID006447131	CID006918453			

(55) $\mathbb{S}_{55}$: 260 drug compounds having side effect “Jaundice”

CID000000444	CID000000450	CID000000596	CID000000937	CID000001065
CID000001690	CID000001972	CID000001986	CID000002022	CID000002092
CID000002099	CID000002118	CID000002160	CID000002162	CID000002170
CID000002171	CID000002179	CID000002250	CID000002269	CID000002274
CID000002311	CID000002315	CID000002375	CID000002478	CID000002520
CID000002550	CID000002554	CID000002609	CID000002631	CID000002637
CID000002646	CID000002650	CID000002654	CID000002658	CID000002662
CID000002666	CID000002675	CID000002676	CID000002678	CID000002708
CID000002712	CID000002720	CID000002726	CID000002727	CID000002732
CID000002764	CID000002771	CID000002786	CID000002794	CID000002818
CID000002895	CID000002907	CID000002913	CID000002949	CID000002995
CID000003015	CID000003016	CID000003032	CID000003059	CID000003114
CID000003121	CID000003143	CID000003152	CID000003157	CID000003158
CID000003203	CID000003222	CID000003278	CID000003285	CID000003308
CID000003324	CID000003325	CID000003339	CID000003342	CID000003345
CID000003355	CID000003365	CID000003366	CID000003372	CID000003386
CID000003387	CID000003394	CID000003397	CID000003403	CID000003404
CID000003417	CID000003419	CID000003440	CID000003446	CID000003454
CID000003463	CID000003475	CID000003476	CID000003478	CID000003488
CID000003510	CID000003559	CID000003639	CID000003647	CID000003648
CID000003672	CID000003675	CID000003696	CID000003698	CID000003702
CID000003706	CID000003715	CID000003749	CID000003750	CID000003763
CID000003767	CID000003793	CID000003825	CID000003826	CID000003869
CID000003877	CID000003883	CID000003899	CID000003928	CID000003929
CID000003937	CID000003956	CID000003958	CID000003961	CID000003962
CID000003964	CID000004011	CID000004033	CID000004036	CID000004044
CID000004053	CID000004060	CID000004075	CID000004078	CID000004107
CID000004121	CID000004138	CID000004160	CID000004168	CID000004170
CID000004171	CID000004200	CID000004212	CID000004259	CID000004409
CID000004451	CID000004463	CID000004485	CID000004497	CID000004509
CID000004513	CID000004536	CID000004539	CID000004543	CID000004547
CID000004583	CID000004585	CID000004594	CID000004614	CID000004616
CID000004638	CID000004679	CID000004691	CID000004723	CID000004740
CID000004745	CID000004748	CID000004856	CID000004870	CID000004889
CID000004915	CID000004917	CID000004920	CID000004927	CID000004932
CID000004976	CID000005029	CID000005038	CID000005039	CID000005070
CID000005073	CID000005076	CID000005090	CID000005203	CID000005206
CID000005291	CID000005344	CID000005352	CID000005372	CID000005401
CID000005402	CID000005408	CID000005426	CID000005430	CID000005452
CID000005454	CID000005472	CID000005487	CID000005503	CID000005505
CID000005516	CID000005533	CID000005546	CID000005556	CID000005566
CID000005578	CID000005584	CID000005591	CID000005625	CID000005656

CID000005718	CID000005726	CID000005731	CID000005734	CID000005878
CID000006691	CID000010100	CID000010631	CID000041317	CID000041774
CID000054454	CID000054688	CID000060184	CID000060613	CID000060787
CID000060795	CID000060953	CID000062867	CID000062959	CID000064147
CID000065027	CID000071273	CID000071616	CID000072938	CID000083786
CID000104865	CID000119182	CID000122316	CID000125889	CID000130881
CID000147912	CID000148192	CID000150610	CID000213039	CID000216239
CID000443871	CID000477468	CID000657298	CID001349907	CID002761171
CID003002190	CID005282044	CID005329102	CID005353980	CID005362070
CID005381226	CID005473385	CID006398970	CID006918453	CID011954225

(56) $\mathbb{S}_{56}$: 257 drug compounds having side effect “Rhinitis”

CID000000085	CID000000159	CID000000214	CID000000444	CID000000450
CID000000453	CID000000598	CID000000738	CID000000750	CID000000772
CID000000807	CID000000937	CID000000942	CID000001690	CID000001775
CID000001935	CID000001972	CID000001978	CID000002083	CID000002092
CID000002140	CID000002162	CID000002182	CID000002187	CID000002216
CID000002250	CID000002269	CID000002284	CID000002311	CID000002369
CID000002375	CID000002405	CID000002435	CID000002462	CID000002476
CID000002478	CID000002487	CID000002512	CID000002520	CID000002541
CID000002550	CID000002554	CID000002585	CID000002609	CID000002662
CID000002676	CID000002678	CID000002713	CID000002751	CID000002764
CID000002769	CID000002771	CID000002801	CID000002802	CID000002806
CID000002891	CID000002909	CID000002958	CID000002978	CID000003016
CID000003032	CID000003066	CID000003075	CID000003108	CID000003121
CID000003152	CID000003157	CID000003219	CID000003241	CID000003261
CID000003285	CID000003308	CID000003339	CID000003345	CID000003348
CID000003350	CID000003386	CID000003394	CID000003403	CID000003404
CID000003410	CID000003414	CID000003417	CID000003419	CID000003446
CID000003449	CID000003454	CID000003461	CID000003475	CID000003478
CID000003510	CID000003519	CID000003648	CID000003672	CID000003702
CID000003724	CID000003734	CID000003736	CID000003749	CID000003750
CID000003793	CID000003825	CID000003826	CID000003827	CID000003878
CID000003883	CID000003899	CID000003911	CID000003937	CID000003948
CID000003956	CID000003961	CID000004075	CID000004091	CID000004158
CID000004159	CID000004171	CID000004173	CID000004200	CID000004201
CID000004205	CID000004212	CID000004236	CID000004253	CID000004259
CID000004264	CID000004449	CID000004451	CID000004473	CID000004485
CID000004493	CID000004510	CID000004513	CID000004542	CID000004547
CID000004583	CID000004585	CID000004594	CID000004609	CID000004634
CID000004635	CID000004679	CID000004691	CID000004739	CID000004745
CID000004819	CID000004885	CID000004889	CID000004893	CID000004920
CID000004932	CID000005002	CID000005005	CID000005035	CID000005040
CID000005064	CID000005070	CID000005073	CID000005076	CID000005077

CID000005095	CID000005152	CID000005155	CID000005195	CID000005203
CID000005210	CID000005212	CID000005245	CID000005291	CID000005372
CID000005401	CID000005419	CID000005426	CID000005466	CID000005487
CID000005496	CID000005514	CID000005523	CID000005544	CID000005596
CID000005625	CID000005636	CID000005645	CID000005650	CID000005656
CID000005726	CID000005731	CID000005732	CID000005734	CID000005735
CID000009034	CID000027661	CID000031477	CID000034312	CID000039507
CID000040976	CID000041317	CID000041781	CID000042615	CID000050294
CID000054547	CID000054688	CID000054786	CID000057469	CID000057537
CID000059708	CID000060184	CID000060198	CID000060613	CID000060714
CID000060754	CID000060787	CID000060795	CID000060865	CID000060877
CID000060953	CID000062819	CID000062959	CID000065999	CID000068844
CID000071158	CID000071273	CID000071616	CID000072054	CID000074989
CID000077992	CID000077993	CID000082146	CID000083786	CID000110634
CID000115237	CID000119607	CID000122316	CID000123606	CID000123620
CID000125889	CID000130881	CID000147912	CID000150610	CID000160051
CID000176168	CID000197712	CID000216326	CID000444013	CID000444033
CID003081884	CID004659569	CID005281104	CID005282226	CID005311027
CID005362070	CID005493381	CID005493444	CID006398525	CID006436173
CID006447131	CID009571074			

(57) $\mathbb{S}_{57}$: 252 drug compounds having side effect “Erythema”

CID000000137	CID000000143	CID000000214	CID000000450	CID000000581
CID000000598	CID000000612	CID000000772	CID000000807	CID000000838
CID000000942	CID000001546	CID000001972	CID000002022	CID000002140
CID000002156	CID000002162	CID000002171	CID000002216	CID000002269
CID000002274	CID000002369	CID000002405	CID000002435	CID000002474
CID000002477	CID000002478	CID000002484	CID000002487	CID000002522
CID000002554	CID000002585	CID000002622	CID000002646	CID000002658
CID000002662	CID000002708	CID000002713	CID000002749	CID000002762
CID000002764	CID000002786	CID000002800	CID000002803	CID000002812
CID000002818	CID000002907	CID000002951	CID000002955	CID000002973
CID000003000	CID000003003	CID000003009	CID000003032	CID000003075
CID000003121	CID000003143	CID000003152	CID000003154	CID000003198
CID000003203	CID000003255	CID000003310	CID000003333	CID000003339
CID000003345	CID000003372	CID000003381	CID000003385	CID000003386
CID000003397	CID000003403	CID000003405	CID000003467	CID000003475
CID000003476	CID000003478	CID000003488	CID000003494	CID000003512
CID000003553	CID000003639	CID000003652	CID000003657	CID000003672
CID000003676	CID000003685	CID000003702	CID000003715	CID000003724
CID000003736	CID000003737	CID000003741	CID000003742	CID000003749
CID000003793	CID000003821	CID000003823	CID000003869	CID000003877
CID000003878	CID000003890	CID000003911	CID000003915	CID000003929
CID000003937	CID000003961	CID000003998	CID000004046	CID000004062

CID000004075 CID000004078 CID000004091 CID000004095 CID000004114
CID000004158 CID000004189 CID000004192 CID000004200 CID000004201
CID000004212 CID000004259 CID000004264 CID000004421 CID000004440
CID000004451 CID000004463 CID000004510 CID000004539 CID000004583
CID000004594 CID000004603 CID000004609 CID000004623 CID000004634
CID000004679 CID000004691 CID000004725 CID000004748 CID000004812
CID000004834 CID000004856 CID000004865 CID000004885 CID000004889
CID000004894 CID000004900 CID000004914 CID000004917 CID000004927
CID000004932 CID000004943 CID000004946 CID000005038 CID000005039
CID000005076 CID000005077 CID000005078 CID000005090 CID000005195
CID000005212 CID000005300 CID000005320 CID000005358 CID000005372
CID000005376 CID000005379 CID000005394 CID000005402 CID000005408
CID000005452 CID000005478 CID000005496 CID000005503 CID000005505
CID000005523 CID000005538 CID000005544 CID000005566 CID000005578
CID000005625 CID000005647 CID000005656 CID000005672 CID000005726
CID000005731 CID000005746 CID000007029 CID000007187 CID000008612
CID000009034 CID000010631 CID000012536 CID000014888 CID000027686
CID000027991 CID000030623 CID000031378 CID000032797 CID000038904
CID000039042 CID000040159 CID000041317 CID000041693 CID000041774
CID000047319 CID000047641 CID000047725 CID000048175 CID000054786
CID000057469 CID000059768 CID000060164 CID000060184 CID000060787
CID000060852 CID000060953 CID000062867 CID000062924 CID000065863
CID000065999 CID000071273 CID000082146 CID000093860 CID000104865
CID000110634 CID000119182 CID000123606 CID000123620 CID000124087
CID000147912 CID000148211 CID000150610 CID000158440 CID000166548
CID000170361 CID000176870 CID000216239 CID000216326 CID000222786
CID000477468 CID003062316 CID003081884 CID004479097 CID004630253
CID005229711 CID005311027 CID005329102 CID005353980 CID006398970
CID006447131 CID011954225

(58) $\mathbb{S}_{58}$: 256 drug compounds having side effect “Agitation”

CID000000159 CID000000450 CID000000596 CID000000767 CID000000807
CID000000838 CID000000942 CID000001065 CID000001125 CID000001206
CID000001690 CID000001775 CID000001935 CID000001972 CID000002022
CID000002083 CID000002130 CID000002140 CID000002153 CID000002160
CID000002162 CID000002171 CID000002269 CID000002284 CID000002315
CID000002349 CID000002381 CID000002405 CID000002441 CID000002462
CID000002474 CID000002476 CID000002478 CID000002487 CID000002524
CID000002554 CID000002564 CID000002576 CID000002578 CID000002609
CID000002654 CID000002666 CID000002678 CID000002708 CID000002720
CID000002725 CID000002732 CID000002756 CID000002762 CID000002764
CID000002781 CID000002801 CID000002802 CID000002803 CID000002818
CID000002895 CID000002905 CID000002907 CID000002909 CID000002913
CID000002995 CID000003007 CID000003075 CID000003100 CID000003121

CID000003148	CID000003152	CID000003157	CID000003168	CID000003203
CID000003261	CID000003285	CID000003325	CID000003345	CID000003348
CID000003367	CID000003372	CID000003373	CID000003393	CID000003404
CID000003406	CID000003410	CID000003414	CID000003440	CID000003446
CID000003449	CID000003454	CID000003510	CID000003519	CID000003559
CID000003639	CID000003647	CID000003648	CID000003661	CID000003675
CID000003676	CID000003690	CID000003696	CID000003702	CID000003724
CID000003736	CID000003739	CID000003741	CID000003878	CID000003883
CID000003911	CID000003948	CID000003958	CID000003961	CID000003964
CID000004011	CID000004046	CID000004054	CID000004058	CID000004062
CID000004078	CID000004095	CID000004121	CID000004158	CID000004168
CID000004170	CID000004192	CID000004236	CID000004253	CID000004259
CID000004409	CID000004419	CID000004425	CID000004428	CID000004440
CID000004506	CID000004510	CID000004539	CID000004543	CID000004583
CID000004594	CID000004595	CID000004601	CID000004603	CID000004635
CID000004727	CID000004737	CID000004740	CID000004745	CID000004748
CID000004771	CID000004819	CID000004870	CID000004885	CID000004914
CID000004917	CID000004927	CID000004943	CID000004976	CID000004991
CID000005002	CID000005029	CID000005038	CID000005039	CID000005040
CID000005064	CID000005070	CID000005071	CID000005073	CID000005076
CID000005077	CID000005078	CID000005095	CID000005193	CID000005206
CID000005210	CID000005212	CID000005358	CID000005372	CID000005379
CID000005391	CID000005394	CID000005426	CID000005452	CID000005454
CID000005466	CID000005487	CID000005514	CID000005523	CID000005525
CID000005538	CID000005556	CID000005566	CID000005572	CID000005584
CID000005625	CID000005647	CID000005665	CID000005718	CID000005719
CID000005721	CID000005731	CID000005732	CID000005734	CID000005735
CID000005978	CID000007029	CID000008612	CID000009034	CID000009433
CID000010631	CID000014888	CID000020585	CID000023897	CID000027661
CID000027991	CID000028112	CID000034312	CID000042113	CID000051263
CID000057469	CID000059708	CID000059768	CID000060184	CID000060612
CID000060613	CID000060787	CID000062867	CID000062924	CID000062959
CID000064147	CID000068740	CID000071158	CID000071273	CID000071616
CID000077992	CID000077993	CID000082146	CID000093860	CID000115237
CID000122316	CID000123606	CID000124087	CID000125889	CID000130881
CID000148192	CID000150610	CID000158440	CID000170361	CID000216326
CID000444013	CID000450096	CID002761171	CID003081884	CID004479097
CID004659568	CID004659569	CID005281104	CID005311297	CID005487301
CID011947681				

(59) $\mathbb{S}_{59}$: 241 drug compounds having side effect “Hepatitis”

CID000000444	CID000000450	CID000000807	CID000001065	CID000001134
CID000001690	CID000001775	CID000001972	CID000002022	CID000002082
CID000002099	CID000002118	CID000002156	CID000002160	CID000002162

CID000002170	CID000002171	CID000002179	CID000002187	CID000002250
CID000002269	CID000002274	CID000002311	CID000002315	CID000002478
CID000002520	CID000002541	CID000002550	CID000002554	CID000002609
CID000002617	CID000002629	CID000002631	CID000002658	CID000002662
CID000002666	CID000002675	CID000002676	CID000002678	CID000002751
CID000002764	CID000002769	CID000002771	CID000002794	CID000002800
CID000002801	CID000002803	CID000002806	CID000002818	CID000002895
CID000002913	CID000002951	CID000002983	CID000002995	CID000003015
CID000003032	CID000003040	CID000003043	CID000003059	CID000003108
CID000003117	CID000003121	CID000003143	CID000003152	CID000003157
CID000003203	CID000003222	CID000003255	CID000003279	CID000003308
CID000003333	CID000003339	CID000003342	CID000003365	CID000003372
CID000003386	CID000003394	CID000003397	CID000003403	CID000003404
CID000003419	CID000003446	CID000003449	CID000003454	CID000003475
CID000003476	CID000003488	CID000003637	CID000003672	CID000003702
CID000003706	CID000003749	CID000003763	CID000003793	CID000003825
CID000003826	CID000003827	CID000003869	CID000003878	CID000003899
CID000003937	CID000003948	CID000003961	CID000003962	CID000003964
CID000004030	CID000004044	CID000004053	CID000004054	CID000004060
CID000004075	CID000004091	CID000004112	CID000004138	CID000004170
CID000004171	CID000004173	CID000004178	CID000004200	CID000004212
CID000004259	CID000004428	CID000004449	CID000004451	CID000004463
CID000004485	CID000004493	CID000004497	CID000004509	CID000004513
CID000004539	CID000004543	CID000004547	CID000004583	CID000004585
CID000004594	CID000004599	CID000004603	CID000004614	CID000004679
CID000004691	CID000004723	CID000004740	CID000004745	CID000004819
CID000004829	CID000004834	CID000004856	CID000004889	CID000004920
CID000004932	CID000005005	CID000005029	CID000005038	CID000005039
CID000005070	CID000005073	CID000005076	CID000005090	CID000005095
CID000005155	CID000005195	CID000005203	CID000005206	CID000005215
CID000005291	CID000005344	CID000005352	CID000005372	CID000005376
CID000005379	CID000005426	CID000005472	CID000005487	CID000005504
CID000005508	CID000005514	CID000005523	CID000005525	CID000005530
CID000005538	CID000005591	CID000005625	CID000005647	CID000005650
CID000005656	CID000005717	CID000005718	CID000005726	CID000005735
CID000006691	CID000010631	CID000041317	CID000041774	CID000042113
CID000054454	CID000054688	CID000059708	CID000060164	CID000060184
CID000060198	CID000060613	CID000060787	CID000060795	CID000060953
CID000062959	CID000064147	CID000065027	CID000071158	CID000071616
CID000072938	CID000074989	CID000077999	CID000083786	CID000093860
CID000104865	CID000119607	CID000124087	CID000125889	CID000147912
CID000148192	CID000150311	CID000153941	CID000213039	CID000443871
CID000657298	CID001349907	CID002761171	CID003002190	CID003062316
CID003081884	CID004659568	CID005311297	CID005353980	CID005361912

CID005381226 CID005481350 CID005487301 CID005493381 CID006323497
CID006398970

(60) $\mathbb{S}_{60}$: 243 drug compounds having side effect “Sweating”

CID000000159 CID000000187 CID000000191 CID000000444 CID000000450
CID000000581 CID000000807 CID000000838 CID000000888 CID000000937
CID000000951 CID000001065 CID000001690 CID000001775 CID000001972
CID000001978 CID000002083 CID000002099 CID000002131 CID000002140
CID000002160 CID000002170 CID000002182 CID000002187 CID000002244
CID000002249 CID000002250 CID000002269 CID000002284 CID000002311
CID000002369 CID000002370 CID000002375 CID000002405 CID000002471
CID000002476 CID000002487 CID000002520 CID000002541 CID000002551
CID000002564 CID000002585 CID000002609 CID000002658 CID000002725
CID000002764 CID000002771 CID000002781 CID000002801 CID000002803
CID000002818 CID000002895 CID000002907 CID000002913 CID000002949
CID000002951 CID000002978 CID000002995 CID000003003 CID000003007
CID000003016 CID000003019 CID000003032 CID000003042 CID000003059
CID000003066 CID000003075 CID000003080 CID000003100 CID000003121
CID000003152 CID000003154 CID000003156 CID000003158 CID000003261
CID000003308 CID000003339 CID000003340 CID000003345 CID000003372
CID000003373 CID000003379 CID000003393 CID000003394 CID000003403
CID000003404 CID000003440 CID000003446 CID000003454 CID000003461
CID000003475 CID000003478 CID000003494 CID000003510 CID000003519
CID000003640 CID000003648 CID000003661 CID000003675 CID000003696
CID000003715 CID000003736 CID000003737 CID000003739 CID000003742
CID000003746 CID000003750 CID000003759 CID000003779 CID000003793
CID000003825 CID000003826 CID000003869 CID000003877 CID000003878
CID000003883 CID000003902 CID000003911 CID000003937 CID000003961
CID000004011 CID000004046 CID000004057 CID000004058 CID000004075
CID000004095 CID000004112 CID000004158 CID000004171 CID000004173
CID000004205 CID000004212 CID000004236 CID000004253 CID000004259
CID000004411 CID000004425 CID000004436 CID000004440 CID000004449
CID000004451 CID000004485 CID000004493 CID000004510 CID000004513
CID000004543 CID000004583 CID000004585 CID000004609 CID000004635
CID000004679 CID000004724 CID000004739 CID000004740 CID000004745
CID000004748 CID000004812 CID000004819 CID000004828 CID000004885
CID000004914 CID000004915 CID000004934 CID000004976 CID000005002
CID000005029 CID000005035 CID000005040 CID000005073 CID000005076
CID000005078 CID000005090 CID000005095 CID000005155 CID000005195
CID000005210 CID000005212 CID000005253 CID000005358 CID000005372
CID000005376 CID000005379 CID000005396 CID000005401 CID000005403
CID000005408 CID000005426 CID000005466 CID000005478 CID000005479
CID000005486 CID000005487 CID000005496 CID000005514 CID000005515
CID000005516 CID000005523 CID000005525 CID000005533 CID000005538

CID000005544 CID000005584 CID000005645 CID000005719 CID000005731
CID000005734 CID000005735 CID000005978 CID000012536 CID000019090
CID000027661 CID000032800 CID000060184 CID000060198 CID000060613
CID000060714 CID000060795 CID000062819 CID000062867 CID000071158
CID000071329 CID000071616 CID000072054 CID000074989 CID000077993
CID000082146 CID000083786 CID000104741 CID000104758 CID000110634
CID000110635 CID000122316 CID000125889 CID000148192 CID000150610
CID000216326 CID003081884 CID005229711 CID005281104 CID005282044
CID005362420 CID005481350 CID009571074

(61) $\mathbb{S}_{61}$: 236 drug compounds having side effect “Influenza”

CID000000085 CID000000159 CID000000214 CID000000444 CID000000450
CID000000596 CID000000598 CID000000738 CID000000937 CID000000942
CID000001134 CID000001690 CID000001775 CID000001971 CID000001972
CID000002083 CID000002088 CID000002092 CID000002118 CID000002182
CID000002187 CID000002250 CID000002267 CID000002269 CID000002284
CID000002311 CID000002369 CID000002375 CID000002435 CID000002462
CID000002476 CID000002477 CID000002512 CID000002520 CID000002541
CID000002585 CID000002609 CID000002662 CID000002676 CID000002713
CID000002726 CID000002771 CID000002786 CID000002802 CID000002806
CID000002909 CID000002955 CID000002958 CID000003015 CID000003032
CID000003066 CID000003075 CID000003121 CID000003148 CID000003152
CID000003154 CID000003157 CID000003161 CID000003203 CID000003285
CID000003325 CID000003333 CID000003339 CID000003345 CID000003348
CID000003379 CID000003381 CID000003386 CID000003394 CID000003403
CID000003404 CID000003410 CID000003414 CID000003417 CID000003419
CID000003446 CID000003461 CID000003702 CID000003746 CID000003749
CID000003827 CID000003878 CID000003883 CID000003890 CID000003899
CID000003902 CID000003911 CID000003937 CID000003948 CID000003961
CID000003962 CID000004054 CID000004086 CID000004091 CID000004158
CID000004173 CID000004200 CID000004201 CID000004205 CID000004236
CID000004253 CID000004428 CID000004449 CID000004493 CID000004542
CID000004583 CID000004585 CID000004594 CID000004599 CID000004634
CID000004635 CID000004666 CID000004679 CID000004691 CID000004739
CID000004740 CID000004745 CID000004819 CID000004856 CID000004885
CID000004889 CID000004917 CID000004920 CID000004932 CID000005002
CID000005035 CID000005038 CID000005040 CID000005064 CID000005070
CID000005073 CID000005076 CID000005077 CID000005090 CID000005095
CID000005152 CID000005155 CID000005195 CID000005210 CID000005212
CID000005245 CID000005253 CID000005291 CID000005358 CID000005372
CID000005376 CID000005394 CID000005401 CID000005402 CID000005426
CID000005466 CID000005487 CID000005512 CID000005514 CID000005523
CID000005544 CID000005596 CID000005625 CID000005636 CID000005645
CID000005647 CID000005656 CID000005718 CID000005726 CID000005731

CID000005732	CID000005734	CID000005735	CID000010631	CID000027661
CID000032797	CID000039042	CID000040976	CID000041317	CID000051634
CID000054688	CID000054786	CID000057469	CID000057537	CID000059708
CID000060198	CID000060613	CID000060787	CID000060795	CID000060852
CID000060865	CID000060953	CID000062819	CID000062924	CID000064147
CID000065027	CID000065999	CID000068740	CID000071158	CID000071329
CID000071616	CID000072054	CID000074989	CID000077993	CID000077999
CID000082146	CID000104741	CID000104758	CID000104865	CID000110634
CID000119607	CID000122316	CID000123606	CID000123620	CID000125889
CID000130881	CID000147912	CID000148211	CID000150310	CID000150311
CID000160051	CID000170361	CID000197712	CID000216239	CID000216326
CID000443871	CID000444013	CID000444033	CID003081884	CID004659569
CID005281007	CID005281104	CID005282226	CID005311181	CID005311297
CID005361912	CID005362070	CID005362420	CID005493381	CID005493444
CID006447131				

(62) $\mathbb{S}_{62}$: 241 drug compounds having side effect "Seizures"

CID000000085	CID000000143	CID000000159	CID000000191	CID000000214
CID000000401	CID000000450	CID000000596	CID000000727	CID000000767
CID000000853	CID000001065	CID000001690	CID000001972	CID000002022
CID000002118	CID000002130	CID000002141	CID000002153	CID000002160
CID000002170	CID000002179	CID000002182	CID000002269	CID000002274
CID000002284	CID000002349	CID000002370	CID000002441	CID000002443
CID000002474	CID000002477	CID000002478	CID000002487	CID000002520
CID000002576	CID000002578	CID000002610	CID000002617	CID000002622
CID000002631	CID000002637	CID000002646	CID000002650	CID000002654
CID000002655	CID000002656	CID000002658	CID000002666	CID000002675
CID000002678	CID000002708	CID000002719	CID000002726	CID000002764
CID000002769	CID000002771	CID000002800	CID000002801	CID000002895
CID000002905	CID000002951	CID000002978	CID000002995	CID000003007
CID000003040	CID000003108	CID000003143	CID000003152	CID000003158
CID000003226	CID000003255	CID000003261	CID000003310	CID000003325
CID000003342	CID000003345	CID000003365	CID000003367	CID000003372
CID000003373	CID000003385	CID000003386	CID000003394	CID000003404
CID000003405	CID000003406	CID000003414	CID000003454	CID000003494
CID000003559	CID000003648	CID000003657	CID000003672	CID000003676
CID000003685	CID000003690	CID000003696	CID000003715	CID000003736
CID000003741	CID000003779	CID000003821	CID000003883	CID000003911
CID000003929	CID000003956	CID000003958	CID000003964	CID000004011
CID000004046	CID000004053	CID000004060	CID000004095	CID000004107
CID000004112	CID000004140	CID000004158	CID000004159	CID000004163
CID000004168	CID000004173	CID000004178	CID000004192	CID000004205
CID000004212	CID000004253	CID000004259	CID000004419	CID000004425
CID000004449	CID000004451	CID000004536	CID000004583	CID000004585

CID000004595	CID000004603	CID000004609	CID000004635	CID000004666
CID000004679	CID000004723	CID000004740	CID000004745	CID000004748
CID000004834	CID000004885	CID000004891	CID000004920	CID000004927
CID000004932	CID000004943	CID000004976	CID000004991	CID000005002
CID000005029	CID000005064	CID000005070	CID000005073	CID000005076
CID000005078	CID000005195	CID000005206	CID000005210	CID000005212
CID000005358	CID000005372	CID000005379	CID000005394	CID000005401
CID000005408	CID000005426	CID000005454	CID000005487	CID000005523
CID000005533	CID000005538	CID000005582	CID000005584	CID000005645
CID000005647	CID000005656	CID000005718	CID000005721	CID000005726
CID000005735	CID000005771	CID000005978	CID000006058	CID000009034
CID000009433	CID000010631	CID000014888	CID000019090	CID000028112
CID000036339	CID000039860	CID000040159	CID000047319	CID000050614
CID000054547	CID000054688	CID000059768	CID000060612	CID000060613
CID000060714	CID000060754	CID000060787	CID000060795	CID000062867
CID000064147	CID000071158	CID000071273	CID000071616	CID000077992
CID000077993	CID000093860	CID000096312	CID000110634	CID000115237
CID000122316	CID000123606	CID000124087	CID000125017	CID000145068
CID000148192	CID000150610	CID000151165	CID000166548	CID000170361
CID000444013	CID003062316	CID003081884	CID005281007	CID005311297
CID005329102	CID005361912	CID005493444	CID006398970	CID006435110
CID009571074				

(63) $\mathbb{S}_{63}$: 225 drug compounds having side effect "Sinusitis"

CID000000085	CID000000159	CID000000214	CID000000444	CID000000450
CID000000942	CID000001690	CID000001775	CID000001935	CID000001972
CID000002083	CID000002092	CID000002182	CID000002187	CID000002250
CID000002267	CID000002308	CID000002311	CID000002369	CID000002375
CID000002405	CID000002435	CID000002462	CID000002478	CID000002487
CID000002520	CID000002541	CID000002554	CID000002583	CID000002585
CID000002609	CID000002662	CID000002676	CID000002678	CID000002713
CID000002751	CID000002769	CID000002771	CID000002786	CID000002801
CID000002802	CID000002806	CID000002909	CID000002955	CID000002958
CID000002978	CID000003016	CID000003032	CID000003066	CID000003075
CID000003121	CID000003154	CID000003157	CID000003219	CID000003241
CID000003261	CID000003285	CID000003308	CID000003325	CID000003333
CID000003339	CID000003345	CID000003348	CID000003367	CID000003379
CID000003381	CID000003385	CID000003386	CID000003394	CID000003403
CID000003404	CID000003410	CID000003414	CID000003419	CID000003446
CID000003475	CID000003510	CID000003702	CID000003724	CID000003746
CID000003793	CID000003878	CID000003883	CID000003899	CID000003911
CID000003937	CID000003961	CID000003962	CID000004158	CID000004173
CID000004196	CID000004201	CID000004205	CID000004212	CID000004236
CID000004428	CID000004440	CID000004449	CID000004451	CID000004473

CID000004485	CID000004513	CID000004542	CID000004547	CID000004594
CID000004603	CID000004614	CID000004634	CID000004635	CID000004679
CID000004691	CID000004739	CID000004745	CID000004819	CID000004829
CID000004885	CID000004889	CID000004920	CID000005035	CID000005038
CID000005040	CID000005064	CID000005070	CID000005073	CID000005076
CID000005077	CID000005090	CID000005095	CID000005152	CID000005155
CID000005195	CID000005203	CID000005210	CID000005212	CID000005245
CID000005291	CID000005358	CID000005372	CID000005376	CID000005394
CID000005401	CID000005426	CID000005466	CID000005478	CID000005487
CID000005496	CID000005512	CID000005514	CID000005523	CID000005538
CID000005544	CID000005625	CID000005636	CID000005645	CID000005650
CID000005656	CID000005665	CID000005718	CID000005721	CID000005726
CID000005731	CID000005732	CID000010631	CID000014888	CID000027661
CID000034312	CID000040976	CID000041317	CID000047725	CID000050294
CID000054454	CID000054547	CID000054786	CID000057469	CID000057537
CID000059708	CID000060184	CID000060198	CID000060613	CID000060787
CID000060795	CID000060865	CID000060871	CID000060877	CID000062819
CID000062924	CID000062959	CID000064147	CID000065999	CID000071616
CID000072054	CID000074989	CID000077992	CID000077993	CID000077999
CID000093860	CID000096312	CID000104865	CID000110634	CID000119607
CID000123606	CID000123620	CID000125889	CID000130881	CID000147912
CID000148211	CID000150311	CID000158440	CID000160051	CID000170361
CID000197712	CID000216326	CID000444013	CID000444033	CID003081884
CID004659568	CID004659569	CID005281104	CID005282226	CID005311027
CID005311297	CID005481350	CID005487301	CID006447131	CID009571074

(64) $\mathbb{S}_{64}$: 232 drug compounds having side effect “Hallucinations”

CID000000206	CID000000444	CID000000564	CID000000767	CID000000807
CID000001935	CID000001972	CID000001978	CID000002022	CID000002118
CID000002130	CID000002156	CID000002160	CID000002170	CID000002215
CID000002249	CID000002284	CID000002344	CID000002349	CID000002369
CID000002405	CID000002441	CID000002476	CID000002477	CID000002478
CID000002487	CID000002512	CID000002554	CID000002578	CID000002609
CID000002622	CID000002662	CID000002666	CID000002678	CID000002708
CID000002756	CID000002764	CID000002769	CID000002771	CID000002801
CID000002802	CID000002803	CID000002806	CID000002818	CID000002895
CID000002905	CID000002913	CID000002958	CID000002978	CID000002995
CID000003007	CID000003016	CID000003032	CID000003059	CID000003062
CID000003075	CID000003121	CID000003125	CID000003152	CID000003154
CID000003156	CID000003158	CID000003203	CID000003255	CID000003261
CID000003279	CID000003305	CID000003308	CID000003324	CID000003325
CID000003345	CID000003355	CID000003366	CID000003373	CID000003386
CID000003393	CID000003394	CID000003404	CID000003414	CID000003446
CID000003449	CID000003454	CID000003467	CID000003559	CID000003648

CID000003657	CID000003658	CID000003661	CID000003672	CID000003690
CID000003696	CID000003741	CID000003759	CID000003763	CID000003821
CID000003825	CID000003826	CID000003878	CID000003883	CID000003948
CID000003958	CID000004011	CID000004044	CID000004046	CID000004054
CID000004058	CID000004095	CID000004140	CID000004158	CID000004168
CID000004171	CID000004173	CID000004178	CID000004192	CID000004205
CID000004236	CID000004253	CID000004259	CID000004411	CID000004419
CID000004425	CID000004428	CID000004440	CID000004449	CID000004506
CID000004539	CID000004543	CID000004583	CID000004585	CID000004594
CID000004601	CID000004603	CID000004614	CID000004616	CID000004635
CID000004679	CID000004691	CID000004723	CID000004736	CID000004737
CID000004739	CID000004745	CID000004819	CID000004828	CID000004856
CID000004893	CID000004913	CID000004915	CID000004927	CID000004943
CID000004946	CID000004976	CID000004991	CID000005002	CID000005039
CID000005040	CID000005064	CID000005070	CID000005071	CID000005073
CID000005076	CID000005077	CID000005090	CID000005095	CID000005193
CID000005195	CID000005203	CID000005215	CID000005344	CID000005358
CID000005372	CID000005379	CID000005391	CID000005394	CID000005466
CID000005478	CID000005512	CID000005514	CID000005523	CID000005525
CID000005533	CID000005538	CID000005556	CID000005572	CID000005584
CID000005596	CID000005625	CID000005647	CID000005656	CID000005665
CID000005718	CID000005719	CID000005721	CID000005731	CID000005732
CID000005735	CID000005978	CID000006058	CID000006476	CID000010100
CID000027686	CID000028112	CID000039860	CID000054547	CID000054688
CID000057537	CID000059708	CID000060612	CID000060613	CID000060787
CID000060795	CID000062959	CID000064147	CID000068740	CID000071158
CID000071273	CID000071616	CID000072054	CID000077993	CID000122316
CID000125889	CID000148192	CID000150610	CID000151165	CID000158440
CID000170361	CID000444013	CID003081884	CID004659568	CID004659569
CID005311297	CID005353980			

(65) $\mathbb{S}_{65}$: 225 drug compounds having side effect “Bradycardia”

CID000000158	CID000000159	CID000000187	CID000000191	CID000000214
CID000000564	CID000000681	CID000000767	CID000000951	CID000001065
CID000001690	CID000001775	CID000001935	CID000001972	CID000001978
CID000002141	CID000002156	CID000002162	CID000002179	CID000002216
CID000002232	CID000002249	CID000002284	CID000002369	CID000002375
CID000002381	CID000002405	CID000002431	CID000002435	CID000002443
CID000002474	CID000002476	CID000002477	CID000002478	CID000002554
CID000002583	CID000002585	CID000002751	CID000002756	CID000002764
CID000002771	CID000002801	CID000002803	CID000002818	CID000002995
CID000003003	CID000003016	CID000003019	CID000003032	CID000003108
CID000003114	CID000003121	CID000003148	CID000003152	CID000003154
CID000003157	CID000003222	CID000003251	CID000003325	CID000003333

CID000003340	CID000003345	CID000003355	CID000003373	CID000003386
CID000003404	CID000003406	CID000003419	CID000003446	CID000003449
CID000003510	CID000003518	CID000003519	CID000003639	CID000003640
CID000003648	CID000003672	CID000003676	CID000003715	CID000003724
CID000003734	CID000003736	CID000003741	CID000003750	CID000003821
CID000003826	CID000003869	CID000003883	CID000003911	CID000003914
CID000003937	CID000003948	CID000003958	CID000004046	CID000004054
CID000004058	CID000004062	CID000004095	CID000004107	CID000004138
CID000004140	CID000004158	CID000004168	CID000004171	CID000004178
CID000004192	CID000004205	CID000004236	CID000004253	CID000004259
CID000004411	CID000004419	CID000004440	CID000004485	CID000004497
CID000004583	CID000004585	CID000004594	CID000004595	CID000004691
CID000004724	CID000004737	CID000004739	CID000004745	CID000004748
CID000004819	CID000004828	CID000004885	CID000004891	CID000004893
CID000004894	CID000004913	CID000004914	CID000004927	CID000004932
CID000004943	CID000004991	CID000005002	CID000005029	CID000005039
CID000005052	CID000005064	CID000005070	CID000005073	CID000005077
CID000005078	CID000005090	CID000005095	CID000005193	CID000005203
CID000005206	CID000005210	CID000005253	CID000005314	CID000005358
CID000005372	CID000005379	CID000005426	CID000005478	CID000005486
CID000005487	CID000005514	CID000005523	CID000005525	CID000005533
CID000005544	CID000005625	CID000005656	CID000005731	CID000005734
CID000005771	CID000005775	CID000008612	CID000010631	CID000027661
CID000028112	CID000031477	CID000034312	CID000036339	CID000041693
CID000042113	CID000047319	CID000047320	CID000051263	CID000054786
CID000056959	CID000059768	CID000060184	CID000060612	CID000060714
CID000060753	CID000060795	CID000060953	CID000062959	CID000065999
CID000068740	CID000071273	CID000071301	CID000071329	CID000071616
CID000077992	CID000077993	CID000083786	CID000093860	CID000104865
CID000115237	CID000119607	CID000125889	CID000147912	CID000148211
CID000150610	CID000151165	CID000158440	CID000170361	CID000213039
CID000216326	CID000477468	CID003002190	CID003081884	CID004659568
CID004659569	CID005282044	CID005282226	CID005329102	CID011947681

(66) $\mathbb{S}_{66}$: 220 drug compounds having side effect “Hematuria”

CID000000159	CID000000175	CID000000214	CID000000450	CID000000598
CID000000772	CID000000807	CID000001690	CID000001935	CID000001972
CID000001986	CID000002022	CID000002083	CID000002140	CID000002179
CID000002182	CID000002232	CID000002250	CID000002267	CID000002284
CID000002349	CID000002375	CID000002462	CID000002478	CID000002541
CID000002554	CID000002585	CID000002654	CID000002656	CID000002662
CID000002678	CID000002720	CID000002764	CID000002771	CID000002801
CID000002806	CID000002907	CID000002909	CID000002949	CID000002951
CID000003015	CID000003032	CID000003040	CID000003059	CID000003125

CID000003148	CID000003152	CID000003157	CID000003261	CID000003278
CID000003291	CID000003308	CID000003325	CID000003342	CID000003345
CID000003355	CID000003365	CID000003367	CID000003386	CID000003394
CID000003397	CID000003404	CID000003410	CID000003414	CID000003417
CID000003446	CID000003449	CID000003454	CID000003461	CID000003648
CID000003672	CID000003690	CID000003706	CID000003715	CID000003724
CID000003734	CID000003736	CID000003741	CID000003793	CID000003825
CID000003826	CID000003878	CID000003883	CID000003899	CID000003911
CID000003948	CID000004044	CID000004054	CID000004075	CID000004100
CID000004101	CID000004112	CID000004140	CID000004205	CID000004211
CID000004212	CID000004236	CID000004259	CID000004409	CID000004449
CID000004485	CID000004493	CID000004539	CID000004542	CID000004583
CID000004585	CID000004594	CID000004607	CID000004609	CID000004614
CID000004634	CID000004635	CID000004679	CID000004691	CID000004727
CID000004745	CID000004819	CID000004856	CID000004865	CID000004885
CID000004911	CID000004915	CID000004932	CID000004993	CID000005005
CID000005029	CID000005040	CID000005070	CID000005073	CID000005076
CID000005077	CID000005095	CID000005155	CID000005195	CID000005203
CID000005210	CID000005212	CID000005291	CID000005344	CID000005352
CID000005358	CID000005372	CID000005379	CID000005408	CID000005426
CID000005430	CID000005466	CID000005472	CID000005486	CID000005487
CID000005504	CID000005508	CID000005514	CID000005523	CID000005533
CID000005538	CID000005596	CID000005625	CID000005645	CID000005647
CID000005656	CID000005719	CID000005726	CID000005731	CID000005734
CID000005735	CID000006049	CID000006476	CID000027661	CID000034312
CID000041317	CID000041744	CID000054547	CID000054688	CID000054786
CID000056959	CID000060184	CID000060613	CID000060795	CID000060865
CID000060871	CID000060877	CID000060953	CID000064147	CID000065999
CID000068740	CID000071158	CID000071273	CID000071616	CID000072938
CID000082146	CID000083786	CID000093860	CID000104865	CID000110634
CID000119182	CID000119607	CID000122316	CID000123631	CID000125889
CID000130881	CID000145068	CID000147912	CID000148192	CID000150610
CID000153941	CID000158440	CID000213039	CID000216239	CID000216326
CID003081884	CID004183806	CID004659568	CID004659569	CID005353980
CID005381226	CID005481350	CID006323497	CID006436173	CID006918453

(67) $\mathbb{S}_{67}$: 221 drug compounds having side effect “Conjunctivitis”

CID000000444	CID000000450	CID000000596	CID000000598	CID000001690
CID000001775	CID000001935	CID000001971	CID000001972	CID000001978
CID000002083	CID000002140	CID000002162	CID000002182	CID000002216
CID000002269	CID000002369	CID000002375	CID000002405	CID000002431
CID000002435	CID000002462	CID000002476	CID000002477	CID000002524
CID000002541	CID000002554	CID000002609	CID000002662	CID000002678
CID000002751	CID000002771	CID000002801	CID000002806	CID000002905

CID000002909 CID000002958 CID000002978 CID000003032 CID000003066
CID000003075 CID000003080 CID000003105 CID000003121 CID000003143
CID000003151 CID000003152 CID000003154 CID000003157 CID000003222
CID000003285 CID000003308 CID000003339 CID000003345 CID000003379
CID000003384 CID000003385 CID000003386 CID000003394 CID000003403
CID000003404 CID000003414 CID000003446 CID000003475 CID000003478
CID000003519 CID000003637 CID000003661 CID000003672 CID000003687
CID000003702 CID000003724 CID000003734 CID000003736 CID000003741
CID000003746 CID000003749 CID000003825 CID000003878 CID000003883
CID000003890 CID000003899 CID000003911 CID000003948 CID000003961
CID000004004 CID000004036 CID000004044 CID000004054 CID000004060
CID000004075 CID000004107 CID000004112 CID000004158 CID000004171
CID000004173 CID000004205 CID000004212 CID000004236 CID000004253
CID000004259 CID000004428 CID000004449 CID000004463 CID000004485
CID000004539 CID000004585 CID000004594 CID000004603 CID000004609
CID000004614 CID000004634 CID000004666 CID000004691 CID000004739
CID000004740 CID000004745 CID000004819 CID000004856 CID000004885
CID000004893 CID000004920 CID000004946 CID000005002 CID000005029
CID000005035 CID000005038 CID000005040 CID000005064 CID000005073
CID000005076 CID000005077 CID000005090 CID000005095 CID000005152
CID000005155 CID000005195 CID000005203 CID000005206 CID000005212
CID000005291 CID000005352 CID000005358 CID000005372 CID000005379
CID000005401 CID000005426 CID000005453 CID000005466 CID000005478
CID000005487 CID000005514 CID000005523 CID000005538 CID000005625
CID000005636 CID000005645 CID000005656 CID000005718 CID000005719
CID000005731 CID000005732 CID000005734 CID000005735 CID000027661
CID000027991 CID000031477 CID000041317 CID000042113 CID000054688
CID000057469 CID000059708 CID000060164 CID000060184 CID000060613
CID000060787 CID000060795 CID000060953 CID000062819 CID000062924
CID000062959 CID000065999 CID000068740 CID000068844 CID000071616
CID000077992 CID000077993 CID000082146 CID000083786 CID000104758
CID000104865 CID000110634 CID000110635 CID000119607 CID000122316
CID000123606 CID000123620 CID000123631 CID000125889 CID000147912
CID000151165 CID000158440 CID000170361 CID000176870 CID000216326
CID000444013 CID000444033 CID003062316 CID003081884 CID004183806
CID004659568 CID005281104 CID005282226 CID005311027 CID005311297
CID005362420 CID005381226 CID006398970 CID006435110 CID006447131
CID006918453

(68) $\mathbb{S}_{68}$: 228 drug compounds having side effect “Agranulocytosis”

CID000000298 CID000000564 CID000000598 CID000001065 CID000001690
CID000001775 CID000001972 CID000001978 CID000001986 CID000002082
CID000002130 CID000002145 CID000002156 CID000002160 CID000002170
CID000002171 CID000002173 CID000002179 CID000002249 CID000002265

CID000002315	CID000002369	CID000002405	CID000002478	CID000002541
CID000002554	CID000002564	CID000002609	CID000002610	CID000002617
CID000002622	CID000002631	CID000002637	CID000002646	CID000002650
CID000002654	CID000002655	CID000002656	CID000002658	CID000002662
CID000002666	CID000002675	CID000002712	CID000002719	CID000002720
CID000002725	CID000002726	CID000002727	CID000002732	CID000002751
CID000002756	CID000002764	CID000002769	CID000002771	CID000002781
CID000002786	CID000002806	CID000002895	CID000002913	CID000002995
CID000003032	CID000003040	CID000003059	CID000003100	CID000003114
CID000003121	CID000003143	CID000003154	CID000003158	CID000003261
CID000003278	CID000003291	CID000003305	CID000003308	CID000003325
CID000003342	CID000003355	CID000003365	CID000003366	CID000003372
CID000003385	CID000003393	CID000003394	CID000003404	CID000003414
CID000003440	CID000003454	CID000003467	CID000003475	CID000003476
CID000003478	CID000003488	CID000003512	CID000003559	CID000003637
CID000003639	CID000003647	CID000003648	CID000003652	CID000003672
CID000003696	CID000003702	CID000003715	CID000003767	CID000003825
CID000003826	CID000003869	CID000003878	CID000003883	CID000003899
CID000003928	CID000003948	CID000003956	CID000003958	CID000003961
CID000003964	CID000004011	CID000004030	CID000004033	CID000004036
CID000004044	CID000004060	CID000004064	CID000004075	CID000004078
CID000004100	CID000004112	CID000004121	CID000004138	CID000004168
CID000004170	CID000004171	CID000004173	CID000004178	CID000004200
CID000004205	CID000004212	CID000004236	CID000004409	CID000004411
CID000004463	CID000004506	CID000004509	CID000004539	CID000004543
CID000004583	CID000004594	CID000004607	CID000004609	CID000004614
CID000004616	CID000004691	CID000004724	CID000004727	CID000004739
CID000004748	CID000004828	CID000004834	CID000004856	CID000004870
CID000004909	CID000004917	CID000004927	CID000004932	CID000004946
CID000004976	CID000005002	CID000005029	CID000005039	CID000005070
CID000005073	CID000005090	CID000005095	CID000005203	CID000005215
CID000005253	CID000005267	CID000005320	CID000005342	CID000005344
CID000005352	CID000005402	CID000005426	CID000005452	CID000005454
CID000005478	CID000005487	CID000005496	CID000005503	CID000005505
CID000005508	CID000005514	CID000005530	CID000005538	CID000005566
CID000005584	CID000005651	CID000005656	CID000005672	CID000005717
CID000005726	CID000007029	CID000013342	CID000016362	CID000030623
CID000034312	CID000050614	CID000051634	CID000054547	CID000060953
CID000062867	CID000062959	CID000068740	CID000071616	CID000072938
CID000083786	CID000130881	CID000147912	CID000216326	CID000657298
CID001349907	CID005353980	CID005381226	CID005493381	CID006398970
CID006918453	CID009571074	CID011954225		

(69) $\mathbb{S}_{69}$: 227 drug compounds having side effect “Weakness”

CID000000158	CID000000191	CID000000206	CID000000401	CID000000596
CID000000838	CID000000853	CID000001065	CID000001546	CID000001690
CID000001935	CID000001978	CID000002083	CID000002118	CID000002130
CID000002140	CID000002156	CID000002160	CID000002170	CID000002215
CID000002274	CID000002284	CID000002315	CID000002405	CID000002471
CID000002474	CID000002476	CID000002477	CID000002478	CID000002487
CID000002524	CID000002576	CID000002585	CID000002610	CID000002676
CID000002708	CID000002719	CID000002720	CID000002732	CID000002762
CID000002764	CID000002800	CID000002801	CID000002802	CID000002803
CID000002818	CID000002895	CID000002907	CID000002909	CID000002949
CID000002951	CID000002995	CID000003003	CID000003015	CID000003019
CID000003042	CID000003062	CID000003080	CID000003121	CID000003143
CID000003154	CID000003157	CID000003158	CID000003203	CID000003249
CID000003251	CID000003285	CID000003310	CID000003339	CID000003355
CID000003366	CID000003367	CID000003379	CID000003393	CID000003403
CID000003419	CID000003440	CID000003446	CID000003461	CID000003475
CID000003494	CID000003518	CID000003519	CID000003639	CID000003647
CID000003648	CID000003652	CID000003661	CID000003675	CID000003676
CID000003696	CID000003702	CID000003736	CID000003737	CID000003742
CID000003767	CID000003779	CID000003784	CID000003877	CID000003902
CID000003911	CID000003937	CID000003958	CID000003961	CID000003962
CID000003964	CID000004011	CID000004032	CID000004033	CID000004057
CID000004058	CID000004062	CID000004064	CID000004078	CID000004086
CID000004095	CID000004112	CID000004121	CID000004138	CID000004163
CID000004170	CID000004171	CID000004173	CID000004178	CID000004192
CID000004212	CID000004253	CID000004419	CID000004421	CID000004425
CID000004436	CID000004485	CID000004506	CID000004510	CID000004543
CID000004583	CID000004595	CID000004601	CID000004614	CID000004635
CID000004666	CID000004691	CID000004736	CID000004740	CID000004828
CID000004845	CID000004870	CID000004873	CID000004889	CID000004893
CID000004913	CID000004915	CID000004932	CID000004934	CID000004943
CID000004946	CID000004976	CID000004991	CID000005077	CID000005078
CID000005095	CID000005155	CID000005195	CID000005253	CID000005291
CID000005344	CID000005358	CID000005391	CID000005394	CID000005401
CID000005403	CID000005419	CID000005426	CID000005430	CID000005453
CID000005454	CID000005478	CID000005479	CID000005487	CID000005503
CID000005516	CID000005523	CID000005530	CID000005533	CID000005538
CID000005546	CID000005556	CID000005572	CID000005584	CID000005625
CID000005656	CID000005672	CID000005732	CID000005775	CID000005978
CID000006691	CID000010100	CID000010631	CID000013342	CID000014888
CID000027400	CID000027661	CID000027686	CID000041781	CID000054454
CID000057469	CID000059768	CID000060754	CID000060787	CID000060953
CID000062816	CID000064147	CID000065999	CID000068740	CID000071273
CID000077993	CID000083786	CID000093860	CID000104865	CID000130881

CID000147912 CID000148211 CID000158440 CID004659569 CID005229711
CID005281104 CID006436173

(70) $\mathbb{S}_{70}$: 219 drug compounds having side effect “Chills”

CID000000158 CID000000159 CID000000444 CID000000453 CID000000738
CID000000750 CID000000772 CID000000807 CID000000937 CID000001065
CID000001546 CID000001775 CID000001935 CID000001971 CID000001972
CID000002021 CID000002083 CID000002141 CID000002182 CID000002269
CID000002284 CID000002349 CID000002375 CID000002474 CID000002476
CID000002477 CID000002478 CID000002487 CID000002554 CID000002564
CID000002609 CID000002622 CID000002656 CID000002658 CID000002676
CID000002725 CID000002764 CID000002771 CID000002781 CID000002801
CID000002818 CID000002913 CID000002949 CID000002951 CID000002958
CID000002978 CID000003007 CID000003015 CID000003016 CID000003032
CID000003043 CID000003100 CID000003121 CID000003143 CID000003148
CID000003152 CID000003158 CID000003168 CID000003261 CID000003278
CID000003285 CID000003308 CID000003310 CID000003345 CID000003367
CID000003373 CID000003379 CID000003394 CID000003403 CID000003404
CID000003446 CID000003454 CID000003461 CID000003478 CID000003510
CID000003637 CID000003648 CID000003657 CID000003672 CID000003676
CID000003734 CID000003736 CID000003737 CID000003739 CID000003741
CID000003742 CID000003749 CID000003750 CID000003759 CID000003823
CID000003825 CID000003826 CID000003877 CID000003878 CID000003883
CID000003911 CID000003929 CID000003937 CID000003948 CID000003958
CID000003962 CID000004046 CID000004062 CID000004064 CID000004075
CID000004086 CID000004091 CID000004112 CID000004158 CID000004170
CID000004192 CID000004195 CID000004196 CID000004205 CID000004212
CID000004236 CID000004253 CID000004259 CID000004409 CID000004428
CID000004440 CID000004449 CID000004485 CID000004509 CID000004539
CID000004583 CID000004585 CID000004635 CID000004638 CID000004666
CID000004679 CID000004736 CID000004739 CID000004745 CID000004819
CID000004885 CID000004889 CID000004913 CID000004915 CID000004943
CID000005002 CID000005005 CID000005029 CID000005040 CID000005070
CID000005076 CID000005078 CID000005090 CID000005155 CID000005195
CID000005212 CID000005215 CID000005344 CID000005352 CID000005358
CID000005372 CID000005379 CID000005396 CID000005404 CID000005408
CID000005426 CID000005430 CID000005466 CID000005486 CID000005496
CID000005515 CID000005523 CID000005530 CID000005533 CID000005625
CID000005645 CID000005651 CID000005718 CID000005719 CID000005726
CID000005731 CID000005735 CID000006049 CID000006691 CID000010631
CID000027991 CID000038904 CID000039860 CID000041693 CID000047725
CID000051634 CID000054454 CID000054547 CID000054688 CID000060184
CID000060612 CID000060613 CID000060795 CID000060843 CID000062959
CID000068740 CID000071158 CID000071273 CID000071616 CID000077993

CID000082146 CID000083786 CID000119607 CID000122316 CID000123606
CID000125017 CID000125889 CID000148211 CID000150610 CID000170361
CID000450096 CID003062316 CID003081884 CID004659569 CID005229711
CID005281104 CID005282044 CID005329102 CID005381226

(71) $\mathbb{S}_{71}$: 208 drug compounds having side effect “Peripheral edema”

CID000000085 CID000000159 CID000000444 CID000000450 CID000000596
CID000000598 CID000000772 CID000000937 CID000000942 CID000001125
CID000001690 CID000001935 CID000001972 CID000002022 CID000002088
CID000002130 CID000002162 CID000002182 CID000002187 CID000002216
CID000002250 CID000002284 CID000002311 CID000002375 CID000002405
CID000002512 CID000002520 CID000002541 CID000002554 CID000002578
CID000002585 CID000002609 CID000002662 CID000002678 CID000002726
CID000002764 CID000002771 CID000002806 CID000003016 CID000003032
CID000003075 CID000003121 CID000003125 CID000003143 CID000003148
CID000003152 CID000003203 CID000003285 CID000003333 CID000003339
CID000003342 CID000003345 CID000003350 CID000003372 CID000003379
CID000003386 CID000003404 CID000003410 CID000003446 CID000003449
CID000003461 CID000003648 CID000003702 CID000003724 CID000003741
CID000003749 CID000003793 CID000003878 CID000003883 CID000003899
CID000003902 CID000003911 CID000003937 CID000004054 CID000004064
CID000004075 CID000004091 CID000004163 CID000004171 CID000004205
CID000004236 CID000004253 CID000004259 CID000004449 CID000004485
CID000004493 CID000004510 CID000004547 CID000004585 CID000004594
CID000004609 CID000004634 CID000004635 CID000004679 CID000004691
CID000004739 CID000004745 CID000004748 CID000004819 CID000004829
CID000004885 CID000004917 CID000004920 CID000005002 CID000005005
CID000005029 CID000005035 CID000005038 CID000005040 CID000005070
CID000005073 CID000005076 CID000005077 CID000005090 CID000005095
CID000005195 CID000005203 CID000005210 CID000005212 CID000005245
CID000005291 CID000005372 CID000005376 CID000005379 CID000005381
CID000005394 CID000005401 CID000005408 CID000005426 CID000005452
CID000005454 CID000005466 CID000005478 CID000005512 CID000005514
CID000005523 CID000005525 CID000005538 CID000005566 CID000005596
CID000005625 CID000005645 CID000005719 CID000005731 CID000005734
CID000005735 CID000010631 CID000012536 CID000019090 CID000027661
CID000027991 CID000030623 CID000032797 CID000041744 CID000042615
CID000054547 CID000054786 CID000056959 CID000057537 CID000060184
CID000060198 CID000060612 CID000060613 CID000060795 CID000060953
CID000062867 CID000062959 CID000064147 CID000065999 CID000068740
CID000071158 CID000071273 CID000071301 CID000071329 CID000071616
CID000072054 CID000072938 CID000077993 CID000082146 CID000093860
CID000104741 CID000104865 CID000119607 CID000123631 CID000125889
CID000130881 CID000147912 CID000148192 CID000166548 CID000197712

CID000213039 CID000216239 CID000216326 CID000443871 CID000477468
CID003002190 CID003081884 CID004479097 CID004659569 CID005281104
CID005282044 CID005311027 CID005311181 CID005329102 CID005487301
CID005493444 CID006323497 CID009571074

(72) $\mathbb{S}_{72}$: 216 drug compounds having side effect "Epistaxis"

CID000000159 CID000000444 CID000000772 CID000000942 CID000001546
CID000001690 CID000001775 CID000001935 CID000001972 CID000002083
CID000002118 CID000002162 CID000002182 CID000002249 CID000002250
CID000002267 CID000002369 CID000002375 CID000002462 CID000002477
CID000002478 CID000002487 CID000002512 CID000002541 CID000002656
CID000002662 CID000002676 CID000002678 CID000002751 CID000002764
CID000002771 CID000002786 CID000002801 CID000002802 CID000002806
CID000002818 CID000002909 CID000003032 CID000003066 CID000003075
CID000003121 CID000003143 CID000003148 CID000003152 CID000003154
CID000003157 CID000003261 CID000003333 CID000003345 CID000003348
CID000003365 CID000003367 CID000003379 CID000003385 CID000003386
CID000003394 CID000003404 CID000003419 CID000003446 CID000003449
CID000003475 CID000003510 CID000003648 CID000003672 CID000003715
CID000003736 CID000003746 CID000003749 CID000003784 CID000003825
CID000003826 CID000003827 CID000003869 CID000003878 CID000003883
CID000003899 CID000003911 CID000003937 CID000003948 CID000003961
CID000004075 CID000004112 CID000004158 CID000004170 CID000004205
CID000004236 CID000004259 CID000004264 CID000004428 CID000004449
CID000004451 CID000004485 CID000004583 CID000004585 CID000004594
CID000004603 CID000004609 CID000004634 CID000004635 CID000004679
CID000004691 CID000004740 CID000004745 CID000004819 CID000004856
CID000004885 CID000004893 CID000004915 CID000004932 CID000004991
CID000005002 CID000005029 CID000005038 CID000005040 CID000005052
CID000005070 CID000005073 CID000005076 CID000005077 CID000005078
CID000005090 CID000005095 CID000005152 CID000005195 CID000005203
CID000005210 CID000005212 CID000005291 CID000005352 CID000005372
CID000005379 CID000005401 CID000005426 CID000005466 CID000005472
CID000005496 CID000005508 CID000005514 CID000005523 CID000005525
CID000005538 CID000005544 CID000005625 CID000005645 CID000005650
CID000005656 CID000005718 CID000005719 CID000005731 CID000005732
CID000005735 CID000010631 CID000014888 CID000027661 CID000027686
CID000027991 CID000031477 CID000034312 CID000039860 CID000041317
CID000042615 CID000047725 CID000054547 CID000054786 CID000059708
CID000060184 CID000060613 CID000060787 CID000060795 CID000060865
CID000060953 CID000062924 CID000062959 CID000065999 CID000071158
CID000071616 CID000077992 CID000077993 CID000083786 CID000093860
CID000096312 CID000104865 CID000110634 CID000110635 CID000119182
CID000119607 CID000122316 CID000123606 CID000123620 CID000123631

CID000124087 CID000125017 CID000125889 CID000147912 CID000148211
CID000150610 CID000158440 CID000163742 CID000170361 CID000176870
CID000216239 CID000216326 CID000444013 CID000444033 CID000477468
CID003081884 CID004183806 CID004659568 CID004659569 CID005229711
CID005281104 CID005311181 CID005311297 CID005329102 CID005493444
CID006447131

(73) $\mathbb{S}_{73}$: 211 drug compounds having side effect “Photosensitivity”

CID000000444 CID000001046 CID000001065 CID000001690 CID000001775
CID000001986 CID000002022 CID000002088 CID000002156 CID000002182
CID000002250 CID000002266 CID000002269 CID000002311 CID000002315
CID000002405 CID000002550 CID000002554 CID000002564 CID000002585
CID000002662 CID000002676 CID000002678 CID000002719 CID000002720
CID000002725 CID000002726 CID000002727 CID000002732 CID000002751
CID000002764 CID000002769 CID000002771 CID000002781 CID000002786
CID000002801 CID000002818 CID000002913 CID000002949 CID000002983
CID000003007 CID000003015 CID000003032 CID000003059 CID000003075
CID000003100 CID000003121 CID000003157 CID000003203 CID000003222
CID000003261 CID000003308 CID000003325 CID000003333 CID000003339
CID000003355 CID000003366 CID000003372 CID000003385 CID000003386
CID000003394 CID000003397 CID000003403 CID000003404 CID000003419
CID000003440 CID000003454 CID000003463 CID000003475 CID000003476
CID000003478 CID000003488 CID000003510 CID000003559 CID000003598
CID000003639 CID000003647 CID000003648 CID000003652 CID000003672
CID000003702 CID000003749 CID000003759 CID000003793 CID000003825
CID000003826 CID000003878 CID000003911 CID000003937 CID000003948
CID000003961 CID000003962 CID000003964 CID000004044 CID000004075
CID000004100 CID000004112 CID000004121 CID000004158 CID000004170
CID000004171 CID000004200 CID000004205 CID000004236 CID000004259
CID000004409 CID000004421 CID000004440 CID000004449 CID000004485
CID000004539 CID000004583 CID000004585 CID000004594 CID000004614
CID000004635 CID000004645 CID000004666 CID000004679 CID000004691
CID000004739 CID000004748 CID000004819 CID000004856 CID000004870
CID000004885 CID000004889 CID000004915 CID000004917 CID000004927
CID000005002 CID000005005 CID000005029 CID000005038 CID000005039
CID000005070 CID000005073 CID000005076 CID000005078 CID000005090
CID000005095 CID000005195 CID000005203 CID000005210 CID000005212
CID000005253 CID000005291 CID000005344 CID000005352 CID000005358
CID000005372 CID000005379 CID000005394 CID000005401 CID000005402
CID000005412 CID000005426 CID000005452 CID000005454 CID000005466
CID000005496 CID000005503 CID000005505 CID000005514 CID000005523
CID000005525 CID000005533 CID000005538 CID000005544 CID000005546
CID000005566 CID000005596 CID000005645 CID000005647 CID000005650
CID000005656 CID000005718 CID000005719 CID000005731 CID000005732

CID000005735 CID000027686 CID000034312 CID000039042 CID000039860
CID000041317 CID000042615 CID000054454 CID000057469 CID000060613
CID000060787 CID000060795 CID000060953 CID000062867 CID000062959
CID000064147 CID000071158 CID000071616 CID000072938 CID000110634
CID000119607 CID000122316 CID000123606 CID000125889 CID000130881
CID000148192 CID000170361 CID002761171 CID003062316 CID003081884
CID005362420

(74) $\mathbb{S}_{74}$: 204 drug compounds having side effect “Hyperglycemia”

CID000000444 CID000000596 CID000000767 CID000001690 CID000001775
CID000001971 CID000001972 CID000002083 CID000002099 CID000002153
CID000002162 CID000002177 CID000002250 CID000002284 CID000002315
CID000002369 CID000002375 CID000002462 CID000002471 CID000002478
CID000002541 CID000002554 CID000002578 CID000002585 CID000002662
CID000002720 CID000002726 CID000002732 CID000002751 CID000002764
CID000002769 CID000002771 CID000002801 CID000002818 CID000002907
CID000002909 CID000003000 CID000003003 CID000003015 CID000003019
CID000003032 CID000003043 CID000003075 CID000003152 CID000003154
CID000003157 CID000003278 CID000003308 CID000003345 CID000003355
CID000003367 CID000003382 CID000003386 CID000003394 CID000003404
CID000003410 CID000003440 CID000003446 CID000003449 CID000003461
CID000003559 CID000003639 CID000003647 CID000003648 CID000003661
CID000003672 CID000003702 CID000003706 CID000003715 CID000003763
CID000003767 CID000003793 CID000003826 CID000003877 CID000003878
CID000003883 CID000003899 CID000003911 CID000003929 CID000003948
CID000003961 CID000004044 CID000004054 CID000004121 CID000004170
CID000004212 CID000004236 CID000004259 CID000004409 CID000004436
CID000004440 CID000004451 CID000004485 CID000004493 CID000004583
CID000004585 CID000004609 CID000004614 CID000004635 CID000004666
CID000004679 CID000004691 CID000004745 CID000004748 CID000004829
CID000004856 CID000004870 CID000004885 CID000004894 CID000004917
CID000004932 CID000004943 CID000005002 CID000005005 CID000005029
CID000005040 CID000005073 CID000005076 CID000005077 CID000005095
CID000005152 CID000005155 CID000005195 CID000005203 CID000005206
CID000005210 CID000005212 CID000005352 CID000005358 CID000005372
CID000005379 CID000005394 CID000005426 CID000005430 CID000005454
CID000005466 CID000005478 CID000005487 CID000005514 CID000005523
CID000005525 CID000005566 CID000005591 CID000005625 CID000005656
CID000005718 CID000005719 CID000005731 CID000005732 CID000009433
CID000012536 CID000014888 CID000019090 CID000020585 CID000028112
CID000031378 CID000032797 CID000034312 CID000041744 CID000041781
CID000054547 CID000060612 CID000060613 CID000060787 CID000060795
CID000060877 CID000060953 CID000062924 CID000062959 CID000064147
CID000065027 CID000071158 CID000071273 CID000071616 CID000072938

CID000074989 CID000077993 CID000077999 CID000082146 CID000093860
CID000096312 CID000104865 CID000115237 CID000119607 CID000123606
CID000125889 CID000130881 CID000145068 CID000147912 CID000148192
CID000148211 CID000151165 CID000153941 CID000158440 CID000166548
CID000213039 CID000315411 CID000444013 CID000477468 CID004659568
CID005282044 CID005329102 CID005481350 CID009571074

(75) $\mathbb{S}_{75}$: 201 drug compounds having side effect “Pneumonia”

CID000000159 CID000000444 CID000000450 CID000000596 CID000000598
CID000000772 CID000001065 CID000001546 CID000001690 CID000001775
CID000001935 CID000001971 CID000001972 CID000001978 CID000002156
CID000002182 CID000002215 CID000002232 CID000002250 CID000002284
CID000002311 CID000002315 CID000002369 CID000002375 CID000002405
CID000002478 CID000002550 CID000002554 CID000002578 CID000002585
CID000002662 CID000002678 CID000002708 CID000002720 CID000002771
CID000002801 CID000002802 CID000002806 CID000002818 CID000002907
CID000002909 CID000003016 CID000003032 CID000003121 CID000003143
CID000003148 CID000003152 CID000003157 CID000003203 CID000003222
CID000003279 CID000003308 CID000003325 CID000003339 CID000003345
CID000003355 CID000003367 CID000003372 CID000003379 CID000003386
CID000003394 CID000003404 CID000003419 CID000003446 CID000003449
CID000003454 CID000003461 CID000003475 CID000003639 CID000003648
CID000003672 CID000003702 CID000003750 CID000003793 CID000003826
CID000003883 CID000003899 CID000003911 CID000003937 CID000003961
CID000004044 CID000004046 CID000004053 CID000004054 CID000004075
CID000004112 CID000004121 CID000004158 CID000004170 CID000004171
CID000004205 CID000004212 CID000004236 CID000004428 CID000004440
CID000004449 CID000004493 CID000004542 CID000004585 CID000004603
CID000004609 CID000004614 CID000004635 CID000004666 CID000004679
CID000004691 CID000004739 CID000004745 CID000004819 CID000004856
CID000004885 CID000004915 CID000004920 CID000005002 CID000005005
CID000005029 CID000005035 CID000005038 CID000005039 CID000005040
CID000005064 CID000005070 CID000005073 CID000005077 CID000005090
CID000005095 CID000005152 CID000005195 CID000005245 CID000005291
CID000005344 CID000005352 CID000005372 CID000005376 CID000005394
CID000005408 CID000005426 CID000005466 CID000005487 CID000005514
CID000005515 CID000005523 CID000005525 CID000005538 CID000005625
CID000005645 CID000005656 CID000005672 CID000005719 CID000005731
CID000005732 CID000019090 CID000027661 CID000030623 CID000034312
CID000050294 CID000060184 CID000060787 CID000060795 CID000060852
CID000060877 CID000060953 CID000062867 CID000062924 CID000064147
CID000065027 CID000065999 CID000071158 CID000082146 CID000093860
CID000096312 CID000104865 CID000119182 CID000119607 CID000123631
CID000125889 CID000130881 CID000147912 CID000150610 CID000151165

CID000163742	CID000176870	CID000197712	CID000216326	CID000444013
CID000444033	CID000477468	CID003062316	CID003081884	CID003086672
CID004183806	CID004659568	CID004659569	CID005281104	CID005311181
CID005311297	CID005353980	CID005362070	CID005362420	CID005481350
CID006447131				

(76) $\mathbb{S}_{76}$: 199 drug compounds having side effect “Myocardial infarction”

CID000000158	CID000000159	CID000000191	CID000000444	CID000000807
CID000000853	CID000000861	CID000001125	CID000001935	CID000001972
CID000002140	CID000002141	CID000002160	CID000002162	CID000002170
CID000002182	CID000002187	CID000002232	CID000002311	CID000002369
CID000002375	CID000002405	CID000002443	CID000002477	CID000002520
CID000002541	CID000002554	CID000002662	CID000002751	CID000002764
CID000002771	CID000002801	CID000002818	CID000002895	CID000002907
CID000002909	CID000002958	CID000002995	CID000003003	CID000003007
CID000003015	CID000003019	CID000003032	CID000003066	CID000003075
CID000003108	CID000003143	CID000003148	CID000003152	CID000003154
CID000003157	CID000003222	CID000003285	CID000003308	CID000003310
CID000003333	CID000003339	CID000003340	CID000003345	CID000003367
CID000003385	CID000003386	CID000003394	CID000003404	CID000003410
CID000003419	CID000003446	CID000003461	CID000003475	CID000003519
CID000003640	CID000003648	CID000003661	CID000003672	CID000003696
CID000003706	CID000003724	CID000003734	CID000003741	CID000003749
CID000003750	CID000003784	CID000003825	CID000003826	CID000003878
CID000003883	CID000003902	CID000003911	CID000003948	CID000004011
CID000004044	CID000004054	CID000004140	CID000004159	CID000004205
CID000004212	CID000004259	CID000004409	CID000004428	CID000004440
CID000004473	CID000004485	CID000004539	CID000004547	CID000004583
CID000004595	CID000004614	CID000004666	CID000004679	CID000004691
CID000004745	CID000004819	CID000004829	CID000004856	CID000004885
CID000004894	CID000004943	CID000004976	CID000005005	CID000005029
CID000005070	CID000005073	CID000005076	CID000005077	CID000005078
CID000005090	CID000005095	CID000005195	CID000005203	CID000005210
CID000005212	CID000005234	CID000005358	CID000005372	CID000005376
CID000005426	CID000005466	CID000005487	CID000005516	CID000005523
CID000005533	CID000005538	CID000005544	CID000005584	CID000005645
CID000005656	CID000005672	CID000005731	CID000005732	CID000005978
CID000010631	CID000013342	CID000018140	CID000027661	CID000027991
CID000031477	CID000039042	CID000041317	CID000042113	CID000047725
CID000057469	CID000059768	CID000060198	CID000060612	CID000060754
CID000060795	CID000060953	CID000065999	CID000071158	CID000071273
CID000071301	CID000071329	CID000071616	CID000077992	CID000077993
CID000077999	CID000093860	CID000104865	CID000110634	CID000110635
CID000119607	CID000122316	CID000123606	CID000125017	CID000125889

CID000147912 CID000151165 CID000158440 CID000170361 CID000176870
CID000213039 CID000216326 CID000477468 CID003062316 CID003081884
CID004183806 CID004659569 CID005281104 CID006398970

(77) $\mathbb{S}_{77}$: 199 drug compounds having side effect “Pancreatitis”

CID000000444 CID000000450 CID000000596 CID000000738 CID000001690
CID000001935 CID000001971 CID000002156 CID000002162 CID000002170
CID000002182 CID000002250 CID000002265 CID000002269 CID000002311
CID000002315 CID000002478 CID000002524 CID000002550 CID000002554
CID000002662 CID000002676 CID000002720 CID000002732 CID000002751
CID000002756 CID000002764 CID000002771 CID000002806 CID000002909
CID000002949 CID000002955 CID000002983 CID000003003 CID000003015
CID000003032 CID000003043 CID000003121 CID000003148 CID000003152
CID000003203 CID000003222 CID000003255 CID000003285 CID000003308
CID000003342 CID000003386 CID000003394 CID000003403 CID000003404
CID000003414 CID000003419 CID000003440 CID000003446 CID000003454
CID000003639 CID000003640 CID000003647 CID000003657 CID000003672
CID000003690 CID000003702 CID000003706 CID000003750 CID000003825
CID000003826 CID000003877 CID000003878 CID000003883 CID000003899
CID000003929 CID000003937 CID000003961 CID000003962 CID000004044
CID000004075 CID000004112 CID000004121 CID000004138 CID000004158
CID000004159 CID000004170 CID000004173 CID000004178 CID000004200
CID000004205 CID000004259 CID000004409 CID000004451 CID000004509
CID000004539 CID000004547 CID000004583 CID000004585 CID000004594
CID000004599 CID000004609 CID000004614 CID000004666 CID000004679
CID000004691 CID000004727 CID000004745 CID000004775 CID000004819
CID000004856 CID000004870 CID000004885 CID000004889 CID000004893
CID000004894 CID000004900 CID000004915 CID000004932 CID000004943
CID000005002 CID000005005 CID000005029 CID000005038 CID000005039
CID000005040 CID000005064 CID000005070 CID000005073 CID000005076
CID000005077 CID000005090 CID000005095 CID000005155 CID000005203
CID000005215 CID000005291 CID000005344 CID000005352 CID000005358
CID000005372 CID000005376 CID000005379 CID000005426 CID000005514
CID000005523 CID000005525 CID000005538 CID000005544 CID000005625
CID000005645 CID000005656 CID000005672 CID000005718 CID000005726
CID000005731 CID000010631 CID000031378 CID000034312 CID000041317
CID000054454 CID000054688 CID000059708 CID000060184 CID000060613
CID000060787 CID000060795 CID000060877 CID000062959 CID000064147
CID000065027 CID000071158 CID000071616 CID000072938 CID000074989
CID000082146 CID000083786 CID000093860 CID000119607 CID000123631
CID000125889 CID000130881 CID000147912 CID000148192 CID000150311
CID000150610 CID000153941 CID000176870 CID000213039 CID000216239
CID000216326 CID000222786 CID000667490 CID003002190 CID003062316
CID003081884 CID005281104 CID005282044 CID005311297 CID005329102

CID005353980 CID005362070 CID005481350 CID006323497

(78) $\mathbb{S}_{78}$: 199 drug compounds having side effect "Neutropenia"

CID000000143 CID000000596 CID000001065 CID000001125 CID000001134
CID000001971 CID000002019 CID000002022 CID000002082 CID000002130
CID000002140 CID000002145 CID000002156 CID000002171 CID000002179
CID000002269 CID000002274 CID000002311 CID000002349 CID000002375
CID000002541 CID000002559 CID000002575 CID000002578 CID000002609
CID000002610 CID000002617 CID000002629 CID000002631 CID000002637
CID000002646 CID000002650 CID000002654 CID000002655 CID000002656
CID000002658 CID000002666 CID000002673 CID000002675 CID000002708
CID000002719 CID000002751 CID000002756 CID000002786 CID000002806
CID000002818 CID000002907 CID000002958 CID000002983 CID000003015
CID000003016 CID000003040 CID000003114 CID000003143 CID000003222
CID000003278 CID000003279 CID000003308 CID000003310 CID000003324
CID000003325 CID000003345 CID000003365 CID000003367 CID000003385
CID000003394 CID000003414 CID000003419 CID000003461 CID000003657
CID000003672 CID000003685 CID000003690 CID000003734 CID000003741
CID000003749 CID000003750 CID000003793 CID000003877 CID000003878
CID000003883 CID000003899 CID000003928 CID000003929 CID000003937
CID000003956 CID000004030 CID000004036 CID000004053 CID000004060
CID000004075 CID000004091 CID000004112 CID000004163 CID000004168
CID000004173 CID000004178 CID000004200 CID000004205 CID000004212
CID000004451 CID000004463 CID000004539 CID000004583 CID000004585
CID000004594 CID000004607 CID000004609 CID000004645 CID000004834
CID000004913 CID000004915 CID000005002 CID000005029 CID000005038
CID000005039 CID000005040 CID000005064 CID000005070 CID000005073
CID000005076 CID000005155 CID000005291 CID000005300 CID000005352
CID000005372 CID000005376 CID000005379 CID000005394 CID000005396
CID000005402 CID000005412 CID000005453 CID000005479 CID000005514
CID000005515 CID000005538 CID000005578 CID000005582 CID000005625
CID000005650 CID000005651 CID000005656 CID000005672 CID000005717
CID000005718 CID000005721 CID000005726 CID000005978 CID000014888
CID000027686 CID000030623 CID000034312 CID000038904 CID000041317
CID000047725 CID000050294 CID000050614 CID000051634 CID000054547
CID000054688 CID000059708 CID000060184 CID000060613 CID000060787
CID000060795 CID000060843 CID000060877 CID000060953 CID000064147
CID000065027 CID000068740 CID000074989 CID000082146 CID000093860
CID000096312 CID000104758 CID000119182 CID000123631 CID000150610
CID000151165 CID000153941 CID000166548 CID000216239 CID000216326
CID000477468 CID003002190 CID003062316 CID005281007 CID005282044
CID005329102 CID005353980 CID005361912 CID005493381 CID006323497
CID006398970 CID006436173 CID006918453 CID009571074

(79) $\mathbb{S}_{79}$: 204 drug compounds having side effect “Weight gain”

CID000000085	CID000000159	CID000000444	CID000000450	CID000000596
CID000000767	CID000000942	CID000001690	CID000001935	CID000001972
CID000001978	CID000002118	CID000002160	CID000002162	CID000002170
CID000002179	CID000002182	CID000002187	CID000002250	CID000002267
CID000002284	CID000002369	CID000002375	CID000002405	CID000002462
CID000002477	CID000002478	CID000002512	CID000002554	CID000002585
CID000002662	CID000002678	CID000002771	CID000002800	CID000002801
CID000002802	CID000002803	CID000002818	CID000002895	CID000002907
CID000002909	CID000002913	CID000002949	CID000002995	CID000003003
CID000003015	CID000003043	CID000003075	CID000003114	CID000003121
CID000003143	CID000003152	CID000003157	CID000003158	CID000003261
CID000003285	CID000003339	CID000003379	CID000003404	CID000003419
CID000003446	CID000003475	CID000003518	CID000003639	CID000003640
CID000003675	CID000003696	CID000003715	CID000003784	CID000003825
CID000003826	CID000003827	CID000003878	CID000003883	CID000003902
CID000003911	CID000003937	CID000003961	CID000003964	CID000004060
CID000004075	CID000004078	CID000004095	CID000004138	CID000004158
CID000004163	CID000004171	CID000004201	CID000004205	CID000004212
CID000004236	CID000004259	CID000004409	CID000004411	CID000004428
CID000004449	CID000004485	CID000004493	CID000004536	CID000004542
CID000004543	CID000004585	CID000004594	CID000004609	CID000004614
CID000004679	CID000004723	CID000004745	CID000004775	CID000004828
CID000004829	CID000004845	CID000004885	CID000004889	CID000004893
CID000004894	CID000004920	CID000004976	CID000005002	CID000005005
CID000005029	CID000005035	CID000005038	CID000005040	CID000005052
CID000005070	CID000005073	CID000005077	CID000005090	CID000005095
CID000005195	CID000005212	CID000005291	CID000005358	CID000005372
CID000005376	CID000005394	CID000005401	CID000005408	CID000005426
CID000005452	CID000005466	CID000005508	CID000005512	CID000005514
CID000005515	CID000005533	CID000005538	CID000005544	CID000005584
CID000005591	CID000005596	CID000005625	CID000005645	CID000005656
CID000005665	CID000005719	CID000005734	CID000005735	CID000010631
CID000014888	CID000019090	CID000023897	CID000027400	CID000027661
CID000027991	CID000028112	CID000034312	CID000040976	CID000041317
CID000042615	CID000047725	CID000054547	CID000057537	CID000059708
CID000060198	CID000060613	CID000060787	CID000060795	CID000060865
CID000060953	CID000062819	CID000062924	CID000062959	CID000068740
CID000071158	CID000072054	CID000077993	CID000077999	CID000082146
CID000083786	CID000104865	CID000115237	CID000119607	CID000125889
CID000147912	CID000148192	CID000170361	CID000213039	CID000216326
CID000444033	CID003062316	CID003081884	CID005473385	

(80) $\mathbb{S}_{80}$: 194 drug compounds having side effect "Stomatitis"

CID000000143	CID000000298	CID000000444	CID000000581	CID000000937
CID000000942	CID000001690	CID000001935	CID000001972	CID000002160
CID000002170	CID000002173	CID000002179	CID000002250	CID000002269
CID000002349	CID000002478	CID000002554	CID000002578	CID000002656
CID000002662	CID000002678	CID000002713	CID000002764	CID000002771
CID000002806	CID000002895	CID000002907	CID000002909	CID000002958
CID000002995	CID000003032	CID000003040	CID000003059	CID000003121
CID000003143	CID000003148	CID000003151	CID000003161	CID000003222
CID000003308	CID000003310	CID000003345	CID000003367	CID000003385
CID000003386	CID000003394	CID000003404	CID000003446	CID000003461
CID000003467	CID000003648	CID000003657	CID000003672	CID000003676
CID000003685	CID000003690	CID000003696	CID000003702	CID000003715
CID000003750	CID000003793	CID000003825	CID000003826	CID000003877
CID000003878	CID000003883	CID000003899	CID000003902	CID000003928
CID000003929	CID000003948	CID000003950	CID000004011	CID000004036
CID000004044	CID000004053	CID000004064	CID000004075	CID000004112
CID000004173	CID000004200	CID000004205	CID000004212	CID000004259
CID000004409	CID000004449	CID000004510	CID000004539	CID000004543
CID000004583	CID000004585	CID000004594	CID000004607	CID000004609
CID000004614	CID000004634	CID000004635	CID000004679	CID000004691
CID000004739	CID000004819	CID000004845	CID000004856	CID000004915
CID000004976	CID000005002	CID000005029	CID000005040	CID000005070
CID000005071	CID000005073	CID000005076	CID000005095	CID000005195
CID000005203	CID000005212	CID000005215	CID000005344	CID000005352
CID000005372	CID000005379	CID000005394	CID000005396	CID000005408
CID000005412	CID000005426	CID000005453	CID000005466	CID000005479
CID000005508	CID000005514	CID000005515	CID000005523	CID000005538
CID000005556	CID000005584	CID000005625	CID000005656	CID000005672
CID000005718	CID000005719	CID000005726	CID000005731	CID000005734
CID000005735	CID000005746	CID000005978	CID000027686	CID000030623
CID000034312	CID000038904	CID000041317	CID000054547	CID000054688
CID000060184	CID000060613	CID000060787	CID000060795	CID000060843
CID000060953	CID000062959	CID000068740	CID000071616	CID000072938
CID000077992	CID000077993	CID000083786	CID000093860	CID000096312
CID000104758	CID000119607	CID000122316	CID000123631	CID000125889
CID000147912	CID000150610	CID000151165	CID000176870	CID000216239
CID000216326	CID002761171	CID003002190	CID003062316	CID003081884
CID005281007	CID005329102	CID005353980	CID005362070	CID005473385
CID006398970	CID006918453	CID009571074	CID011954225	

(81) $\mathbb{S}_{81}$: 196 drug compounds having side effect "Stevens - Johnson syndrome"

CID000000444	CID000001065	CID000001546	CID000001690	CID000001775

CID000001971 CID000001972 CID000001986 CID000002022 CID000002082
CID000002088 CID000002118 CID000002141 CID000002156 CID000002162
CID000002171 CID000002177 CID000002187 CID000002250 CID000002269
CID000002311 CID000002520 CID000002550 CID000002554 CID000002585
CID000002609 CID000002610 CID000002617 CID000002622 CID000002631
CID000002637 CID000002646 CID000002650 CID000002654 CID000002656
CID000002658 CID000002662 CID000002666 CID000002675 CID000002676
CID000002708 CID000002720 CID000002751 CID000002756 CID000002764
CID000002771 CID000002786 CID000002806 CID000002818 CID000002907
CID000002949 CID000002983 CID000003007 CID000003032 CID000003059
CID000003075 CID000003121 CID000003143 CID000003203 CID000003222
CID000003255 CID000003261 CID000003291 CID000003292 CID000003305
CID000003308 CID000003310 CID000003324 CID000003325 CID000003342
CID000003345 CID000003365 CID000003367 CID000003386 CID000003394
CID000003403 CID000003404 CID000003414 CID000003419 CID000003446
CID000003454 CID000003639 CID000003652 CID000003672 CID000003702
CID000003706 CID000003715 CID000003793 CID000003825 CID000003826
CID000003827 CID000003878 CID000003883 CID000003899 CID000003914
CID000003928 CID000003929 CID000003937 CID000003948 CID000003954
CID000003956 CID000003961 CID000003962 CID000004011 CID000004036
CID000004044 CID000004046 CID000004054 CID000004060 CID000004064
CID000004100 CID000004112 CID000004121 CID000004158 CID000004170
CID000004173 CID000004178 CID000004200 CID000004201 CID000004259
CID000004409 CID000004421 CID000004449 CID000004463 CID000004485
CID000004509 CID000004539 CID000004547 CID000004583 CID000004594
CID000004595 CID000004603 CID000004614 CID000004666 CID000004679
CID000004834 CID000004856 CID000004889 CID000004946 CID000004993
CID000005002 CID000005005 CID000005029 CID000005038 CID000005073
CID000005077 CID000005090 CID000005203 CID000005215 CID000005291
CID000005320 CID000005344 CID000005352 CID000005372 CID000005376
CID000005379 CID000005394 CID000005402 CID000005426 CID000005430
CID000005472 CID000005514 CID000005523 CID000005578 CID000005596
CID000005625 CID000005651 CID000005656 CID000005726 CID000006476
CID000034312 CID000050614 CID000054454 CID000054547 CID000054688
CID000060184 CID000060787 CID000062959 CID000071616 CID000077999
CID000083786 CID000104865 CID000110635 CID000119607 CID000123631
CID000125889 CID000130881 CID000147912 CID000151165 CID000213039
CID003002190 CID005353980 CID005381226 CID006398970 CID006435110
CID009571074

(82) $\mathbb{S}_{82}$: 195 drug compounds having side effect “Impotence”

CID000000444 CID000000767 CID000000937 CID000001206 CID000001935
CID000001978 CID000002092 CID000002156 CID000002160 CID000002170
CID000002249 CID000002250 CID000002284 CID000002311 CID000002349

CID000002369	CID000002375	CID000002405	CID000002477	CID000002520
CID000002541	CID000002550	CID000002554	CID000002585	CID000002720
CID000002726	CID000002732	CID000002751	CID000002756	CID000002771
CID000002801	CID000002802	CID000002803	CID000002818	CID000002895
CID000002995	CID000003007	CID000003015	CID000003032	CID000003042
CID000003075	CID000003114	CID000003117	CID000003125	CID000003154
CID000003157	CID000003222	CID000003325	CID000003333	CID000003339
CID000003350	CID000003355	CID000003386	CID000003394	CID000003397
CID000003404	CID000003446	CID000003454	CID000003463	CID000003475
CID000003494	CID000003518	CID000003519	CID000003559	CID000003639
CID000003648	CID000003675	CID000003696	CID000003702	CID000003759
CID000003784	CID000003793	CID000003823	CID000003825	CID000003869
CID000003878	CID000003883	CID000003911	CID000003914	CID000003937
CID000003958	CID000003961	CID000004011	CID000004032	CID000004054
CID000004057	CID000004078	CID000004112	CID000004138	CID000004168
CID000004170	CID000004171	CID000004178	CID000004205	CID000004212
CID000004236	CID000004253	CID000004409	CID000004411	CID000004449
CID000004473	CID000004485	CID000004493	CID000004513	CID000004543
CID000004583	CID000004585	CID000004594	CID000004635	CID000004638
CID000004679	CID000004691	CID000004724	CID000004739	CID000004745
CID000004771	CID000004828	CID000004885	CID000004889	CID000004893
CID000004917	CID000004932	CID000004934	CID000004946	CID000004976
CID000005002	CID000005005	CID000005029	CID000005038	CID000005039
CID000005040	CID000005052	CID000005070	CID000005073	CID000005076
CID000005077	CID000005095	CID000005195	CID000005203	CID000005210
CID000005376	CID000005394	CID000005401	CID000005408	CID000005426
CID000005454	CID000005466	CID000005478	CID000005514	CID000005523
CID000005525	CID000005530	CID000005533	CID000005566	CID000005584
CID000005625	CID000005650	CID000005656	CID000005719	CID000005732
CID000005734	CID000007029	CID000009904	CID000019090	CID000023897
CID000027400	CID000027661	CID000028112	CID000041317	CID000041781
CID000042615	CID000047725	CID000057537	CID000060787	CID000060795
CID000062867	CID000062959	CID000064147	CID000065999	CID000071158
CID000071616	CID000072054	CID000077993	CID000083786	CID000119607
CID000122316	CID000125889	CID000148192	CID000152945	CID002761171
CID003081884	CID004183806	CID004659568	CID004659569	CID005281104

(83) $\mathbb{S}_{83}$: 196 drug compounds having side effect “Eosinophilia”

CID000001690	CID000001775	CID000001972	CID000002142	CID000002160
CID000002170	CID000002171	CID000002173	CID000002274	CID000002311
CID000002349	CID000002375	CID000002477	CID000002550	CID000002554
CID000002559	CID000002575	CID000002576	CID000002609	CID000002610
CID000002617	CID000002622	CID000002629	CID000002631	CID000002637
CID000002646	CID000002650	CID000002655	CID000002656	CID000002658

CID000002666	CID000002673	CID000002675	CID000002676	CID000002726
CID000002727	CID000002762	CID000002764	CID000002771	CID000002786
CID000002794	CID000002802	CID000002806	CID000002818	CID000002895
CID000002949	CID000002951	CID000002983	CID000002995	CID000003032
CID000003040	CID000003121	CID000003125	CID000003152	CID000003158
CID000003222	CID000003279	CID000003291	CID000003308	CID000003339
CID000003354	CID000003366	CID000003372	CID000003385	CID000003394
CID000003403	CID000003406	CID000003419	CID000003440	CID000003454
CID000003463	CID000003467	CID000003510	CID000003637	CID000003672
CID000003696	CID000003767	CID000003826	CID000003878	CID000003883
CID000003899	CID000003929	CID000003937	CID000003948	CID000003956
CID000003961	CID000003962	CID000003998	CID000004011	CID000004036
CID000004044	CID000004060	CID000004064	CID000004075	CID000004078
CID000004112	CID000004138	CID000004163	CID000004173	CID000004200
CID000004236	CID000004259	CID000004421	CID000004463	CID000004485
CID000004509	CID000004513	CID000004539	CID000004543	CID000004583
CID000004607	CID000004614	CID000004645	CID000004679	CID000004691
CID000004727	CID000004730	CID000004736	CID000004745	CID000004748
CID000004834	CID000004856	CID000004889	CID000004891	CID000004913
CID000004915	CID000004917	CID000004976	CID000004993	CID000005002
CID000005038	CID000005039	CID000005095	CID000005195	CID000005253
CID000005291	CID000005297	CID000005300	CID000005344	CID000005352
CID000005412	CID000005426	CID000005452	CID000005454	CID000005472
CID000005496	CID000005514	CID000005523	CID000005525	CID000005566
CID000005584	CID000005625	CID000005651	CID000005656	CID000005717
CID000005718	CID000005719	CID000005731	CID000006476	CID000016362
CID000027686	CID000034312	CID000050294	CID000050614	CID000054454
CID000054547	CID000054688	CID000060184	CID000060795	CID000062867
CID000062924	CID000062959	CID000064147	CID000065999	CID000071158
CID000071616	CID000072938	CID000082146	CID000083786	CID000119607
CID000125889	CID000147912	CID000150610	CID000477468	CID003002190
CID003081884	CID005281007	CID005282044	CID005311297	CID005353980
CID005361912	CID005362070	CID005362420	CID005381226	CID006918453
CID011954225				

(84) $\mathbb{S}_{84}$: 186 drug compounds having side effect “Urinary tract infection”

CID000000159	CID000000214	CID000000444	CID000000450	CID000000596
CID000001125	CID000001690	CID000001935	CID000002083	CID000002182
CID000002187	CID000002215	CID000002232	CID000002250	CID000002311
CID000002375	CID000002462	CID000002524	CID000002554	CID000002578
CID000002585	CID000002662	CID000002678	CID000002769	CID000002771
CID000002786	CID000002801	CID000002802	CID000002803	CID000002806
CID000002909	CID000003007	CID000003016	CID000003075	CID000003121
CID000003152	CID000003154	CID000003157	CID000003222	CID000003340

CID000003345	CID000003367	CID000003379	CID000003386	CID000003394
CID000003403	CID000003404	CID000003414	CID000003446	CID000003449
CID000003463	CID000003475	CID000003510	CID000003672	CID000003702
CID000003724	CID000003746	CID000003749	CID000003793	CID000003877
CID000003878	CID000003883	CID000003899	CID000003902	CID000003911
CID000003937	CID000003961	CID000003962	CID000004054	CID000004075
CID000004158	CID000004173	CID000004189	CID000004205	CID000004212
CID000004236	CID000004253	CID000004259	CID000004428	CID000004449
CID000004493	CID000004542	CID000004547	CID000004585	CID000004594
CID000004599	CID000004635	CID000004666	CID000004679	CID000004691
CID000004739	CID000004745	CID000004819	CID000004885	CID000004920
CID000005002	CID000005005	CID000005035	CID000005040	CID000005070
CID000005073	CID000005076	CID000005077	CID000005090	CID000005095
CID000005152	CID000005155	CID000005195	CID000005210	CID000005212
CID000005372	CID000005376	CID000005394	CID000005401	CID000005408
CID000005466	CID000005508	CID000005512	CID000005514	CID000005523
CID000005525	CID000005538	CID000005544	CID000005596	CID000005625
CID000005645	CID000005665	CID000005731	CID000005732	CID000005735
CID000010631	CID000019090	CID000027661	CID000034312	CID000041317
CID000041744	CID000047725	CID000054786	CID000057469	CID000057537
CID000059708	CID000060184	CID000060198	CID000060613	CID000060753
CID000060787	CID000060795	CID000060852	CID000060865	CID000060953
CID000062819	CID000064147	CID000065999	CID000068740	CID000071158
CID000071273	CID000071329	CID000071616	CID000072054	CID000082146
CID000093860	CID000104741	CID000104865	CID000119607	CID000123620
CID000124087	CID000125889	CID000130881	CID000147912	CID000148192
CID000151165	CID000158440	CID000163742	CID000216326	CID000444013
CID003081884	CID004183806	CID004659569	CID005229711	CID005281104
CID005282044	CID005282226	CID005353980	CID005362070	CID005487301
CID005493444				

(85) $\mathbb{S}_{85}$: 192 drug compounds having side effect "Purpura"

CID000000450	CID000000772	CID000000942	CID000001065	CID000001546
CID000001690	CID000001935	CID000001972	CID000001978	CID000001986
CID000002022	CID000002160	CID000002162	CID000002170	CID000002171
CID000002173	CID000002179	CID000002244	CID000002249	CID000002265
CID000002274	CID000002315	CID000002369	CID000002405	CID000002462
CID000002520	CID000002554	CID000002585	CID000002676	CID000002678
CID000002720	CID000002726	CID000002732	CID000002751	CID000002764
CID000002771	CID000002801	CID000002806	CID000002895	CID000002909
CID000002949	CID000002995	CID000003032	CID000003075	CID000003148
CID000003154	CID000003157	CID000003158	CID000003261	CID000003278
CID000003308	CID000003342	CID000003367	CID000003372	CID000003386
CID000003387	CID000003394	CID000003403	CID000003404	CID000003440

CID000003446 CID000003449 CID000003467 CID000003488 CID000003519
CID000003637 CID000003639 CID000003647 CID000003672 CID000003696
CID000003702 CID000003715 CID000003741 CID000003825 CID000003826
CID000003869 CID000003928 CID000003948 CID000003961 CID000003962
CID000004011 CID000004036 CID000004044 CID000004064 CID000004121
CID000004158 CID000004160 CID000004170 CID000004171 CID000004411
CID000004440 CID000004485 CID000004513 CID000004542 CID000004543
CID000004583 CID000004594 CID000004609 CID000004614 CID000004638
CID000004691 CID000004724 CID000004727 CID000004740 CID000004745
CID000004748 CID000004828 CID000004856 CID000004870 CID000004885
CID000004889 CID000004915 CID000004917 CID000004920 CID000004927
CID000004932 CID000004946 CID000004976 CID000005038 CID000005040
CID000005052 CID000005070 CID000005073 CID000005077 CID000005095
CID000005203 CID000005215 CID000005253 CID000005291 CID000005344
CID000005352 CID000005372 CID000005376 CID000005394 CID000005408
CID000005412 CID000005426 CID000005472 CID000005478 CID000005487
CID000005508 CID000005514 CID000005523 CID000005544 CID000005566
CID000005584 CID000005625 CID000005656 CID000005665 CID000005718
CID000005719 CID000005732 CID000005878 CID000009904 CID000010631
CID000016362 CID000027661 CID000027686 CID000031378 CID000034312
CID000039042 CID000041317 CID000042615 CID000054454 CID000054547
CID000057469 CID000057537 CID000060184 CID000060613 CID000060953
CID000062867 CID000065999 CID000071273 CID000071616 CID000077992
CID000077993 CID000083786 CID000122316 CID000125889 CID000130881
CID000147912 CID000148192 CID000163742 CID002761171 CID004183806
CID004659568 CID005353980 CID005381226 CID005473385 CID005493381
CID006323497 CID006398970

(86) $\mathbb{S}_{86}$: 184 drug compounds having side effect “Increased sweating”

CID000000158 CID000000214 CID000000444 CID000000450 CID000000596
CID000000598 CID000000738 CID000000807 CID000000853 CID000000942
CID000001546 CID000001935 CID000002022 CID000002083 CID000002118
CID000002130 CID000002160 CID000002162 CID000002215 CID000002274
CID000002284 CID000002349 CID000002405 CID000002431 CID000002443
CID000002462 CID000002474 CID000002520 CID000002541 CID000002554
CID000002585 CID000002656 CID000002662 CID000002678 CID000002751
CID000002764 CID000002771 CID000002801 CID000002909 CID000002958
CID000003003 CID000003015 CID000003032 CID000003066 CID000003075
CID000003108 CID000003114 CID000003148 CID000003152 CID000003157
CID000003203 CID000003222 CID000003310 CID000003324 CID000003342
CID000003345 CID000003355 CID000003365 CID000003367 CID000003372
CID000003373 CID000003386 CID000003404 CID000003414 CID000003419
CID000003446 CID000003476 CID000003559 CID000003640 CID000003648
CID000003676 CID000003724 CID000003734 CID000003736 CID000003750

CID000003793	CID000003869	CID000003899	CID000003902	CID000003911
CID000003929	CID000003937	CID000003948	CID000004062	CID000004086
CID000004091	CID000004140	CID000004159	CID000004171	CID000004178
CID000004212	CID000004253	CID000004409	CID000004419	CID000004428
CID000004473	CID000004485	CID000004493	CID000004497	CID000004510
CID000004542	CID000004583	CID000004594	CID000004609	CID000004614
CID000004666	CID000004691	CID000004736	CID000004739	CID000004812
CID000004889	CID000004893	CID000004894	CID000004900	CID000004915
CID000004920	CID000004932	CID000004943	CID000004991	CID000005005
CID000005038	CID000005064	CID000005071	CID000005073	CID000005077
CID000005095	CID000005195	CID000005203	CID000005352	CID000005394
CID000005452	CID000005454	CID000005514	CID000005523	CID000005538
CID000005544	CID000005596	CID000005625	CID000005718	CID000005726
CID000005732	CID000007029	CID000008612	CID000010631	CID000014888
CID000027661	CID000031378	CID000034312	CID000039860	CID000041317
CID000042615	CID000054547	CID000057537	CID000059768	CID000060198
CID000060612	CID000060754	CID000060787	CID000060953	CID000062959
CID000064147	CID000065999	CID000068740	CID000071273	CID000077992
CID000104865	CID000119607	CID000123606	CID000147912	CID000151165
CID000158440	CID000166548	CID000216326	CID000222786	CID000443871
CID000444013	CID003002190	CID004183806	CID004659568	CID004659569
CID005229711	CID005487301	CID006436173	CID011947681	

(87) $\mathbb{S}_{87}$: 181 drug compounds having side effect “Dysuria”

CID000000444	CID000001046	CID000001690	CID000001775	CID000001935
CID000001972	CID000001978	CID000002162	CID000002182	CID000002250
CID000002269	CID000002284	CID000002344	CID000002369	CID000002375
CID000002462	CID000002477	CID000002478	CID000002609	CID000002654
CID000002658	CID000002662	CID000002678	CID000002751	CID000002764
CID000002771	CID000002786	CID000002801	CID000002802	CID000002907
CID000002909	CID000002958	CID000002973	CID000003032	CID000003059
CID000003114	CID000003121	CID000003125	CID000003148	CID000003151
CID000003152	CID000003157	CID000003308	CID000003325	CID000003333
CID000003339	CID000003342	CID000003345	CID000003355	CID000003367
CID000003386	CID000003394	CID000003404	CID000003414	CID000003417
CID000003446	CID000003478	CID000003510	CID000003648	CID000003657
CID000003672	CID000003690	CID000003702	CID000003706	CID000003736
CID000003746	CID000003759	CID000003784	CID000003826	CID000003869
CID000003878	CID000003883	CID000003899	CID000003911	CID000003937
CID000003948	CID000004044	CID000004054	CID000004075	CID000004101
CID000004112	CID000004163	CID000004173	CID000004195	CID000004205
CID000004253	CID000004259	CID000004409	CID000004449	CID000004485
CID000004542	CID000004583	CID000004585	CID000004594	CID000004595
CID000004609	CID000004614	CID000004635	CID000004679	CID000004691

CID000004745	CID000004819	CID000004856	CID000004885	CID000004889
CID000004920	CID000004932	CID000005002	CID000005005	CID000005029
CID000005040	CID000005052	CID000005070	CID000005073	CID000005076
CID000005077	CID000005078	CID000005090	CID000005095	CID000005155
CID000005195	CID000005203	CID000005352	CID000005358	CID000005372
CID000005379	CID000005394	CID000005401	CID000005408	CID000005426
CID000005453	CID000005466	CID000005508	CID000005512	CID000005514
CID000005523	CID000005538	CID000005596	CID000005645	CID000005656
CID000005718	CID000005719	CID000005732	CID000005734	CID000005735
CID000005978	CID000007029	CID000010631	CID000027661	CID000027686
CID000034312	CID000040976	CID000041317	CID000041744	CID000054547
CID000059768	CID000060613	CID000060795	CID000060953	CID000062959
CID000064147	CID000065999	CID000071273	CID000071616	CID000082146
CID000083786	CID000104865	CID000119607	CID000122316	CID000125889
CID000147912	CID000150610	CID000151165	CID000158440	CID000216326
CID000444013	CID003081884	CID004183806	CID004659568	CID004659569
CID006436173				

(88) $\mathbb{S}_{88}$: 184 drug compounds having side effect “Bronchitis”

CID000000085	CID000000214	CID000000444	CID000000450	CID000000807
CID000000937	CID000000942	CID000001690	CID000001775	CID000001935
CID000001971	CID000002092	CID000002182	CID000002187	CID000002250
CID000002267	CID000002269	CID000002311	CID000002369	CID000002375
CID000002405	CID000002435	CID000002462	CID000002487	CID000002541
CID000002554	CID000002585	CID000002662	CID000002676	CID000002678
CID000002713	CID000002751	CID000002771	CID000002801	CID000002802
CID000002806	CID000002818	CID000002909	CID000003032	CID000003066
CID000003075	CID000003121	CID000003152	CID000003154	CID000003157
CID000003222	CID000003285	CID000003308	CID000003325	CID000003333
CID000003339	CID000003345	CID000003367	CID000003379	CID000003386
CID000003394	CID000003403	CID000003404	CID000003410	CID000003446
CID000003449	CID000003475	CID000003702	CID000003724	CID000003746
CID000003793	CID000003877	CID000003878	CID000003883	CID000003899
CID000003911	CID000003937	CID000003961	CID000004054	CID000004075
CID000004158	CID000004173	CID000004201	CID000004205	CID000004236
CID000004428	CID000004440	CID000004449	CID000004542	CID000004547
CID000004583	CID000004594	CID000004603	CID000004634	CID000004635
CID000004679	CID000004691	CID000004739	CID000004745	CID000004819
CID000004885	CID000004920	CID000005035	CID000005038	CID000005040
CID000005070	CID000005076	CID000005077	CID000005090	CID000005095
CID000005152	CID000005155	CID000005195	CID000005203	CID000005210
CID000005212	CID000005245	CID000005358	CID000005372	CID000005376
CID000005394	CID000005401	CID000005426	CID000005466	CID000005487
CID000005512	CID000005514	CID000005523	CID000005525	CID000005544

CID000005625	CID000005636	CID000005645	CID000005650	CID000005656
CID000005719	CID000005721	CID000005731	CID000005732	CID000005735
CID000010631	CID000027661	CID000031477	CID000034312	CID000039042
CID000047725	CID000050294	CID000054454	CID000054547	CID000054786
CID000057469	CID000059708	CID000060184	CID000060198	CID000060613
CID000060787	CID000060852	CID000060953	CID000062819	CID000062924
CID000064147	CID000065027	CID000065999	CID000071158	CID000072054
CID000077993	CID000082146	CID000104865	CID000115237	CID000119607
CID000123606	CID000123620	CID000124087	CID000125889	CID000130881
CID000170361	CID000176168	CID000216326	CID000443871	CID003081884
CID004183806	CID004659568	CID004659569	CID005281104	CID005282226
CID005311027	CID005493381	CID005493444	CID006447131	

(89) $\mathbb{S}_{89}$: 188 drug compounds having side effect “Dermatitis”

CID000000143	CID000000298	CID000000444	CID000000450	CID000000727
CID000001065	CID000001690	CID000001775	CID000001935	CID000002099
CID000002118	CID000002130	CID000002156	CID000002162	CID000002171
CID000002216	CID000002266	CID000002269	CID000002274	CID000002311
CID000002369	CID000002405	CID000002462	CID000002522	CID000002578
CID000002585	CID000002662	CID000002678	CID000002751	CID000002764
CID000002771	CID000002786	CID000002800	CID000002801	CID000002806
CID000002818	CID000002907	CID000002909	CID000003000	CID000003003
CID000003032	CID000003075	CID000003108	CID000003143	CID000003152
CID000003157	CID000003203	CID000003219	CID000003279	CID000003310
CID000003339	CID000003355	CID000003375	CID000003381	CID000003382
CID000003385	CID000003403	CID000003419	CID000003440	CID000003454
CID000003463	CID000003475	CID000003510	CID000003518	CID000003519
CID000003553	CID000003598	CID000003657	CID000003661	CID000003676
CID000003690	CID000003749	CID000003823	CID000003826	CID000003878
CID000003911	CID000003928	CID000003929	CID000003961	CID000003964
CID000004053	CID000004054	CID000004060	CID000004064	CID000004091
CID000004112	CID000004158	CID000004170	CID000004173	CID000004201
CID000004205	CID000004264	CID000004440	CID000004451	CID000004485
CID000004510	CID000004539	CID000004542	CID000004583	CID000004594
CID000004603	CID000004679	CID000004736	CID000004745	CID000004819
CID000004845	CID000004885	CID000004889	CID000004911	CID000004915
CID000004927	CID000004932	CID000005005	CID000005040	CID000005064
CID000005073	CID000005077	CID000005095	CID000005203	CID000005210
CID000005212	CID000005291	CID000005352	CID000005372	CID000005379
CID000005381	CID000005394	CID000005408	CID000005452	CID000005453
CID000005514	CID000005515	CID000005516	CID000005523	CID000005538
CID000005544	CID000005556	CID000005625	CID000005656	CID000005672
CID000005718	CID000005732	CID000006691	CID000012536	CID000014888
CID000031477	CID000032797	CID000041317	CID000048175	CID000052421

CID000054547	CID000057469	CID000060164	CID000060184	CID000060198
CID000060787	CID000060852	CID000060953	CID000062816	CID000062867
CID000062924	CID000062959	CID000064147	CID000065999	CID000068740
CID000068844	CID000072938	CID000082146	CID000083786	CID000104865
CID000119182	CID000119607	CID000123606	CID000123620	CID000123631
CID000125889	CID000130881	CID000147912	CID000150610	CID000170361
CID000213039	CID003062316	CID003081884	CID004183806	CID004630253
CID004659568	CID005311297	CID006447131		

(90) $\mathbb{S}_{90}$: 181 drug compounds having side effect “Angina pectoris”

CID000000159	CID000000453	CID000000681	CID000000750	CID000000807
CID000000838	CID000000853	CID000000861	CID000000937	CID000001065
CID000001690	CID000001935	CID000002083	CID000002092	CID000002182
CID000002187	CID000002215	CID000002232	CID000002311	CID000002369
CID000002375	CID000002541	CID000002550	CID000002585	CID000002662
CID000002676	CID000002751	CID000002764	CID000002771	CID000002818
CID000002958	CID000003007	CID000003019	CID000003032	CID000003066
CID000003108	CID000003143	CID000003152	CID000003154	CID000003157
CID000003222	CID000003333	CID000003339	CID000003340	CID000003345
CID000003355	CID000003367	CID000003385	CID000003386	CID000003394
CID000003404	CID000003410	CID000003419	CID000003446	CID000003475
CID000003510	CID000003518	CID000003637	CID000003639	CID000003648
CID000003702	CID000003706	CID000003724	CID000003734	CID000003741
CID000003749	CID000003750	CID000003779	CID000003869	CID000003878
CID000003883	CID000003890	CID000003899	CID000003902	CID000003911
CID000003937	CID000003948	CID000003961	CID000004054	CID000004075
CID000004138	CID000004158	CID000004178	CID000004197	CID000004205
CID000004259	CID000004409	CID000004428	CID000004440	CID000004449
CID000004493	CID000004547	CID000004583	CID000004594	CID000004595
CID000004603	CID000004679	CID000004691	CID000004739	CID000004740
CID000004745	CID000004819	CID000004829	CID000004856	CID000004885
CID000004893	CID000004915	CID000004920	CID000004932	CID000005002
CID000005005	CID000005029	CID000005038	CID000005039	CID000005052
CID000005064	CID000005070	CID000005073	CID000005077	CID000005078
CID000005090	CID000005095	CID000005195	CID000005210	CID000005212
CID000005245	CID000005358	CID000005372	CID000005376	CID000005401
CID000005408	CID000005426	CID000005466	CID000005478	CID000005487
CID000005514	CID000005516	CID000005523	CID000005525	CID000005596
CID000005645	CID000005656	CID000005719	CID000005731	CID000005732
CID000006691	CID000013342	CID000031477	CID000036811	CID000059768
CID000060184	CID000060198	CID000060787	CID000060795	CID000060953
CID000062816	CID000062959	CID000065999	CID000071158	CID000071329
CID000077993	CID000082146	CID000093860	CID000104865	CID000110634
CID000110635	CID000119607	CID000122316	CID000123606	CID000125889

CID000150310 CID000158440 CID000170361 CID000197712 CID000216326
CID003062316 CID003081884 CID004659568 CID004659569 CID005282226
CID005487301

(91) $\mathbb{S}_{91}$: 180 drug compounds having side effect "Asthma"

CID000000444 CID000000450 CID000000772 CID000000807 CID000001065
CID000001775 CID000001935 CID000001972 CID000002140 CID000002171
CID000002173 CID000002182 CID000002216 CID000002244 CID000002249
CID000002250 CID000002266 CID000002267 CID000002269 CID000002311
CID000002349 CID000002370 CID000002375 CID000002405 CID000002478
CID000002576 CID000002585 CID000002609 CID000002673 CID000002678
CID000002726 CID000002764 CID000002769 CID000002771 CID000002802
CID000002973 CID000003015 CID000003016 CID000003032 CID000003152
CID000003158 CID000003203 CID000003222 CID000003261 CID000003285
CID000003305 CID000003308 CID000003339 CID000003345 CID000003372
CID000003386 CID000003394 CID000003404 CID000003417 CID000003419
CID000003446 CID000003475 CID000003518 CID000003648 CID000003672
CID000003715 CID000003724 CID000003734 CID000003736 CID000003737
CID000003741 CID000003742 CID000003826 CID000003878 CID000003883
CID000003890 CID000003899 CID000003911 CID000003937 CID000004044
CID000004054 CID000004075 CID000004078 CID000004158 CID000004168
CID000004173 CID000004200 CID000004205 CID000004236 CID000004253
CID000004259 CID000004409 CID000004419 CID000004440 CID000004449
CID000004542 CID000004583 CID000004585 CID000004594 CID000004599
CID000004603 CID000004614 CID000004634 CID000004679 CID000004691
CID000004727 CID000004739 CID000004745 CID000004748 CID000004856
CID000004885 CID000004917 CID000004920 CID000004927 CID000004991
CID000005002 CID000005005 CID000005029 CID000005040 CID000005064
CID000005070 CID000005073 CID000005076 CID000005090 CID000005095
CID000005195 CID000005212 CID000005253 CID000005358 CID000005372
CID000005379 CID000005401 CID000005408 CID000005452 CID000005453
CID000005466 CID000005487 CID000005496 CID000005514 CID000005523
CID000005538 CID000005566 CID000005645 CID000005656 CID000005719
CID000005735 CID000010631 CID000012536 CID000025419 CID000027661
CID000034312 CID000040976 CID000042113 CID000054547 CID000054688
CID000059708 CID000060184 CID000060613 CID000060787 CID000060795
CID000060953 CID000062867 CID000062959 CID000065999 CID000068740
CID000071158 CID000077993 CID000083786 CID000104865 CID000122316
CID000125889 CID000150610 CID000170361 CID000216326 CID000444013
CID003062316 CID003081884 CID004659569 CID005229711 CID005281104
CID005487301 CID006398970 CID006435110 CID006447131 CID009571074

(92) $\mathbb{S}_{92}$: 178 drug compounds having side effect "Urinary frequency"

CID000000214 CID000000444 CID000000450 CID000001690 CID000001935

CID000002099	CID000002118	CID000002160	CID000002162	CID000002170
CID000002182	CID000002250	CID000002267	CID000002269	CID000002284
CID000002311	CID000002375	CID000002443	CID000002462	CID000002477
CID000002520	CID000002550	CID000002554	CID000002564	CID000002585
CID000002662	CID000002678	CID000002725	CID000002751	CID000002764
CID000002769	CID000002771	CID000002781	CID000002800	CID000002801
CID000002802	CID000002812	CID000002818	CID000002895	CID000002907
CID000002909	CID000002913	CID000002951	CID000002995	CID000003015
CID000003032	CID000003066	CID000003075	CID000003100	CID000003114
CID000003121	CID000003151	CID000003152	CID000003157	CID000003285
CID000003308	CID000003325	CID000003333	CID000003339	CID000003345
CID000003348	CID000003386	CID000003404	CID000003419	CID000003440
CID000003446	CID000003449	CID000003454	CID000003476	CID000003519
CID000003648	CID000003690	CID000003696	CID000003702	CID000003715
CID000003746	CID000003759	CID000003826	CID000003878	CID000003883
CID000003899	CID000003902	CID000003911	CID000003961	CID000004011
CID000004075	CID000004168	CID000004173	CID000004195	CID000004205
CID000004236	CID000004259	CID000004449	CID000004473	CID000004485
CID000004542	CID000004543	CID000004583	CID000004585	CID000004594
CID000004609	CID000004679	CID000004691	CID000004745	CID000004819
CID000004828	CID000004885	CID000004893	CID000004911	CID000004915
CID000004920	CID000004932	CID000004976	CID000004991	CID000005002
CID000005029	CID000005040	CID000005071	CID000005073	CID000005076
CID000005077	CID000005078	CID000005095	CID000005195	CID000005203
CID000005210	CID000005212	CID000005291	CID000005358	CID000005372
CID000005394	CID000005401	CID000005426	CID000005466	CID000005487
CID000005514	CID000005523	CID000005530	CID000005533	CID000005538
CID000005584	CID000005645	CID000005656	CID000005718	CID000005719
CID000005726	CID000005731	CID000005732	CID000005734	CID000005735
CID000010631	CID000019090	CID000027661	CID000027686	CID000034312
CID000039860	CID000041744	CID000054547	CID000060184	CID000060795
CID000062819	CID000062959	CID000064147	CID000065999	CID000071158
CID000077992	CID000077993	CID000104865	CID000119607	CID000125889
CID000147912	CID000148192	CID000158440	CID003062316	CID003081884
CID004659569	CID006436173	CID009571074		

(93) $\mathbb{S}_{93}$: 176 drug compounds having side effect “Upper respiratory tract infection”

CID000000214	CID000000444	CID000000450	CID000000807	CID000001125
CID000001134	CID000001935	CID000002083	CID000002092	CID000002118
CID000002308	CID000002369	CID000002405	CID000002462	CID000002487
CID000002520	CID000002541	CID000002585	CID000002662	CID000002678
CID000002713	CID000002769	CID000002771	CID000002786	CID000002802
CID000002806	CID000002895	CID000002909	CID000003066	CID000003075
CID000003154	CID000003157	CID000003222	CID000003241	CID000003285

CID000003333 CID000003342 CID000003345 CID000003348 CID000003367
CID000003372 CID000003379 CID000003381 CID000003386 CID000003404
CID000003410 CID000003419 CID000003446 CID000003449 CID000003454
CID000003475 CID000003706 CID000003746 CID000003750 CID000003793
CID000003890 CID000003899 CID000003929 CID000003937 CID000003961
CID000004054 CID000004075 CID000004091 CID000004112 CID000004158
CID000004173 CID000004189 CID000004212 CID000004428 CID000004485
CID000004493 CID000004542 CID000004547 CID000004583 CID000004594
CID000004599 CID000004603 CID000004609 CID000004614 CID000004634
CID000004666 CID000004679 CID000004691 CID000004724 CID000004739
CID000004829 CID000004885 CID000004889 CID000004920 CID000004932
CID000005002 CID000005038 CID000005040 CID000005073 CID000005077
CID000005078 CID000005090 CID000005095 CID000005152 CID000005203
CID000005212 CID000005245 CID000005253 CID000005291 CID000005394
CID000005402 CID000005426 CID000005478 CID000005496 CID000005514
CID000005523 CID000005525 CID000005544 CID000005625 CID000005645
CID000005647 CID000005650 CID000005656 CID000005665 CID000005732
CID000010631 CID000012536 CID000014888 CID000027661 CID000027991
CID000032797 CID000034312 CID000040976 CID000042615 CID000047725
CID000050294 CID000054454 CID000057469 CID000057537 CID000059708
CID000060184 CID000060198 CID000060787 CID000060795 CID000060852
CID000060877 CID000060953 CID000062867 CID000062924 CID000062959
CID000064147 CID000065999 CID000068740 CID000077999 CID000093860
CID000104865 CID000115237 CID000119607 CID000123620 CID000124087
CID000130881 CID000147912 CID000150311 CID000150610 CID000151165
CID000158440 CID000197712 CID000216326 CID000443871 CID000444033
CID003062316 CID004183806 CID004659569 CID005229711 CID005311027
CID005311297 CID005481350 CID005487301 CID005493444 CID006436173
CID006447131

(94) $\mathbb{S}_{94}$: 182 drug compounds having side effect “Ataxia”

CID000000444 CID000000767 CID000001065 CID000001690 CID000001775
CID000001935 CID000001986 CID000002022 CID000002083 CID000002118
CID000002123 CID000002130 CID000002156 CID000002160 CID000002162
CID000002170 CID000002249 CID000002284 CID000002369 CID000002441
CID000002443 CID000002477 CID000002487 CID000002550 CID000002554
CID000002576 CID000002578 CID000002656 CID000002662 CID000002678
CID000002708 CID000002712 CID000002751 CID000002764 CID000002771
CID000002801 CID000002802 CID000002818 CID000002895 CID000002905
CID000002958 CID000002978 CID000002995 CID000003016 CID000003121
CID000003143 CID000003148 CID000003152 CID000003157 CID000003158
CID000003203 CID000003222 CID000003261 CID000003291 CID000003292
CID000003355 CID000003366 CID000003373 CID000003385 CID000003386
CID000003393 CID000003394 CID000003404 CID000003414 CID000003446

CID000003449	CID000003454	CID000003648	CID000003652	CID000003661
CID000003675	CID000003696	CID000003702	CID000003759	CID000003878
CID000003914	CID000003937	CID000003948	CID000003950	CID000003958
CID000003961	CID000004011	CID000004046	CID000004054	CID000004060
CID000004064	CID000004078	CID000004112	CID000004163	CID000004173
CID000004178	CID000004192	CID000004205	CID000004236	CID000004253
CID000004449	CID000004485	CID000004506	CID000004539	CID000004543
CID000004583	CID000004585	CID000004609	CID000004616	CID000004666
CID000004691	CID000004737	CID000004739	CID000004745	CID000004748
CID000004885	CID000004909	CID000004915	CID000004927	CID000004932
CID000004976	CID000005002	CID000005070	CID000005071	CID000005073
CID000005076	CID000005077	CID000005078	CID000005095	CID000005193
CID000005195	CID000005203	CID000005212	CID000005215	CID000005267
CID000005344	CID000005379	CID000005391	CID000005394	CID000005426
CID000005466	CID000005479	CID000005514	CID000005516	CID000005523
CID000005530	CID000005533	CID000005556	CID000005584	CID000005647
CID000005656	CID000005665	CID000005718	CID000005719	CID000005731
CID000005732	CID000005734	CID000005735	CID000005978	CID000006058
CID000006476	CID000010631	CID000028112	CID000034312	CID000039860
CID000057537	CID000059708	CID000060613	CID000060754	CID000060787
CID000060795	CID000060953	CID000062959	CID000064147	CID000071616
CID000077992	CID000077993	CID000082146	CID000093860	CID000096312
CID000122316	CID000125889	CID000147912	CID003081884	CID004659568
CID005353980	CID005381226			

(95) $\mathbb{S}_{95}$: 177 drug compounds having side effect “Dysphagia”

CID000000206	CID000000444	CID000000596	CID000000853	CID000000937
CID000001003	CID000001690	CID000001775	CID000001935	CID000001972
CID000002118	CID000002130	CID000002162	CID000002179	CID000002182
CID000002250	CID000002284	CID000002344	CID000002369	CID000002375
CID000002443	CID000002487	CID000002578	CID000002662	CID000002726
CID000002764	CID000002771	CID000002801	CID000002818	CID000002909
CID000002951	CID000002958	CID000002983	CID000003016	CID000003066
CID000003108	CID000003121	CID000003143	CID000003152	CID000003222
CID000003278	CID000003310	CID000003345	CID000003367	CID000003372
CID000003386	CID000003404	CID000003414	CID000003419	CID000003446
CID000003449	CID000003454	CID000003519	CID000003559	CID000003648
CID000003661	CID000003702	CID000003793	CID000003878	CID000003883
CID000003911	CID000003948	CID000003964	CID000004011	CID000004054
CID000004075	CID000004178	CID000004200	CID000004253	CID000004259
CID000004409	CID000004449	CID000004485	CID000004539	CID000004583
CID000004585	CID000004594	CID000004609	CID000004634	CID000004635
CID000004645	CID000004679	CID000004691	CID000004739	CID000004745
CID000004748	CID000004819	CID000004885	CID000004893	CID000004915

CID000004917	CID000004920	CID000004932	CID000005002	CID000005029
CID000005038	CID000005040	CID000005070	CID000005071	CID000005073
CID000005076	CID000005077	CID000005078	CID000005095	CID000005195
CID000005203	CID000005212	CID000005245	CID000005358	CID000005372
CID000005379	CID000005394	CID000005412	CID000005426	CID000005454
CID000005466	CID000005487	CID000005514	CID000005523	CID000005538
CID000005566	CID000005625	CID000005645	CID000005656	CID000005672
CID000005718	CID000005719	CID000005726	CID000005731	CID000005732
CID000005734	CID000005735	CID000010631	CID000023897	CID000025419
CID000027686	CID000030623	CID000034312	CID000060613	CID000060714
CID000060787	CID000060795	CID000060843	CID000060953	CID000062816
CID000062959	CID000064147	CID000068740	CID000071158	CID000071616
CID000072938	CID000077992	CID000077993	CID000093860	CID000110634
CID000110635	CID000115237	CID000119607	CID000122316	CID000125889
CID000147912	CID000150610	CID000151165	CID000170361	CID000176870
CID000216239	CID000216326	CID000444013	CID003062316	CID003081884
CID004183806	CID004479097	CID004659568	CID004659569	CID005281104
CID006436173	CID006918453			

(96) $\mathbb{S}_{96}$: 179 drug compounds having side effect “Convulsions”

CID000000191	CID000000206	CID000000401	CID000000453	CID000000564
CID000000596	CID000000750	CID000000807	CID000001065	CID000001125
CID000001690	CID000001935	CID000001972	CID000001986	CID000002022
CID000002171	CID000002269	CID000002284	CID000002349	CID000002474
CID000002476	CID000002487	CID000002554	CID000002564	CID000002585
CID000002631	CID000002678	CID000002725	CID000002762	CID000002764
CID000002771	CID000002781	CID000002801	CID000002818	CID000002895
CID000002909	CID000002913	CID000002949	CID000002958	CID000003003
CID000003007	CID000003019	CID000003032	CID000003075	CID000003100
CID000003152	CID000003156	CID000003157	CID000003203	CID000003255
CID000003278	CID000003308	CID000003325	CID000003345	CID000003355
CID000003365	CID000003366	CID000003373	CID000003386	CID000003394
CID000003446	CID000003449	CID000003463	CID000003467	CID000003640
CID000003648	CID000003652	CID000003657	CID000003658	CID000003672
CID000003675	CID000003676	CID000003696	CID000003715	CID000003724
CID000003734	CID000003736	CID000003737	CID000003741	CID000003742
CID000003767	CID000003826	CID000003877	CID000003878	CID000003929
CID000003948	CID000003958	CID000004030	CID000004032	CID000004044
CID000004046	CID000004054	CID000004058	CID000004062	CID000004100
CID000004112	CID000004158	CID000004159	CID000004178	CID000004192
CID000004200	CID000004253	CID000004259	CID000004421	CID000004425
CID000004428	CID000004440	CID000004449	CID000004539	CID000004583
CID000004614	CID000004634	CID000004666	CID000004739	CID000004834
CID000004856	CID000004894	CID000004900	CID000004914	CID000004915

CID000004917	CID000004927	CID000004943	CID000005038	CID000005071
CID000005073	CID000005077	CID000005095	CID000005195	CID000005203
CID000005210	CID000005215	CID000005291	CID000005344	CID000005352
CID000005358	CID000005394	CID000005430	CID000005479	CID000005514
CID000005523	CID000005538	CID000005544	CID000005566	CID000005647
CID000005656	CID000005718	CID000005731	CID000005771	CID000005978
CID000008612	CID000009034	CID000010100	CID000010631	CID000013342
CID000016850	CID000020585	CID000027686	CID000027991	CID000031378
CID000034312	CID000039860	CID000051634	CID000054688	CID000057469
CID000057537	CID000060164	CID000060754	CID000060787	CID000060795
CID000062959	CID000071273	CID000096312	CID000119607	CID000147912
CID000148211	CID000222786	CID000477468	CID005353980	

(97) $\mathbb{S}_{97}$: 173 drug compounds having side effect “Weight loss”

CID000000085	CID000000159	CID000000444	CID000000450	CID000000767
CID000001690	CID000001935	CID000001972	CID000002118	CID000002162
CID000002179	CID000002182	CID000002187	CID000002284	CID000002369
CID000002375	CID000002477	CID000002478	CID000002512	CID000002524
CID000002550	CID000002585	CID000002771	CID000002794	CID000002801
CID000002802	CID000002818	CID000002909	CID000003007	CID000003015
CID000003032	CID000003121	CID000003143	CID000003152	CID000003154
CID000003249	CID000003261	CID000003291	CID000003345	CID000003386
CID000003404	CID000003446	CID000003449	CID000003454	CID000003467
CID000003648	CID000003652	CID000003702	CID000003750	CID000003793
CID000003825	CID000003878	CID000003899	CID000003902	CID000003911
CID000003937	CID000003964	CID000004011	CID000004075	CID000004095
CID000004158	CID000004163	CID000004205	CID000004212	CID000004236
CID000004253	CID000004409	CID000004428	CID000004449	CID000004485
CID000004493	CID000004542	CID000004583	CID000004585	CID000004594
CID000004609	CID000004614	CID000004635	CID000004679	CID000004691
CID000004723	CID000004745	CID000004920	CID000005002	CID000005029
CID000005040	CID000005064	CID000005070	CID000005073	CID000005076
CID000005077	CID000005095	CID000005155	CID000005195	CID000005203
CID000005245	CID000005291	CID000005358	CID000005372	CID000005376
CID000005394	CID000005402	CID000005426	CID000005466	CID000005478
CID000005487	CID000005496	CID000005508	CID000005514	CID000005523
CID000005533	CID000005538	CID000005572	CID000005656	CID000005672
CID000005718	CID000005719	CID000005726	CID000005732	CID000005734
CID000005735	CID000005978	CID000006476	CID000010631	CID000014888
CID000023897	CID000027661	CID000028112	CID000034312	CID000040976
CID000041317	CID000042615	CID000051634	CID000057537	CID000059708
CID000060613	CID000060787	CID000060795	CID000060953	CID000062959
CID000064147	CID000065027	CID000068740	CID000071158	CID000077993
CID000082146	CID000093860	CID000104758	CID000115237	CID000119182

CID000119607	CID000122316	CID000123631	CID000125017	CID000125889
CID000147912	CID000148192	CID000150610	CID000151165	CID000176870
CID000216239	CID000216326	CID002761171	CID003062316	CID003081884
CID004659568	CID004659569	CID005281104	CID005329102	CID005481350
CID006436173	CID006918453	CID009571074		

(98) $\mathbb{S}_{98}$: 175 drug compounds having side effect "Erythema multiforme"

CID000000444	CID000000450	CID000001065	CID000001690	CID000001971
CID000002022	CID000002082	CID000002141	CID000002156	CID000002162
CID000002171	CID000002173	CID000002187	CID000002250	CID000002269
CID000002274	CID000002478	CID000002520	CID000002524	CID000002550
CID000002554	CID000002576	CID000002585	CID000002609	CID000002610
CID000002617	CID000002622	CID000002631	CID000002637	CID000002646
CID000002650	CID000002655	CID000002656	CID000002658	CID000002662
CID000002666	CID000002675	CID000002676	CID000002708	CID000002720
CID000002727	CID000002751	CID000002756	CID000002762	CID000002764
CID000002771	CID000002786	CID000002800	CID000002806	CID000002818
CID000002983	CID000003032	CID000003059	CID000003075	CID000003121
CID000003143	CID000003203	CID000003222	CID000003255	CID000003279
CID000003285	CID000003305	CID000003308	CID000003324	CID000003333
CID000003367	CID000003379	CID000003386	CID000003394	CID000003403
CID000003414	CID000003440	CID000003446	CID000003512	CID000003639
CID000003672	CID000003702	CID000003706	CID000003715	CID000003793
CID000003825	CID000003826	CID000003827	CID000003883	CID000003899
CID000003911	CID000003928	CID000003937	CID000003954	CID000003956
CID000003961	CID000003962	CID000004033	CID000004036	CID000004044
CID000004046	CID000004060	CID000004064	CID000004112	CID000004158
CID000004195	CID000004200	CID000004236	CID000004409	CID000004421
CID000004463	CID000004485	CID000004509	CID000004539	CID000004583
CID000004594	CID000004603	CID000004614	CID000004679	CID000004691
CID000004834	CID000004856	CID000004889	CID000004920	CID000004946
CID000004993	CID000005029	CID000005038	CID000005039	CID000005070
CID000005073	CID000005076	CID000005203	CID000005215	CID000005291
CID000005344	CID000005352	CID000005376	CID000005394	CID000005402
CID000005426	CID000005430	CID000005472	CID000005487	CID000005508
CID000005514	CID000005578	CID000005625	CID000005647	CID000005656
CID000005735	CID000010631	CID000034312	CID000050614	CID000054454
CID000054547	CID000057469	CID000060184	CID000060198	CID000060843
CID000071616	CID000072938	CID000074989	CID000083786	CID000104865
CID000119607	CID000123631	CID000130881	CID000213039	CID000216239
CID000444013	CID000477468	CID003002190	CID003062316	CID004659568
CID004659569	CID005353980	CID005381226	CID006398970	CID009571074

(99) $\mathbb{S}_{99}$: 170 drug compounds having side effect "Gastritis"

CID000000085	CID000000159	CID000000444	CID000000450	CID000000767
CID000001125	CID000001134	CID000001690	CID000001775	CID000001935
CID000001971	CID000002099	CID000002162	CID000002182	CID000002250
CID000002269	CID000002311	CID000002375	CID000002405	CID000002519
CID000002609	CID000002662	CID000002666	CID000002678	CID000002764
CID000002771	CID000002801	CID000002802	CID000002806	CID000002895
CID000002909	CID000002958	CID000003032	CID000003059	CID000003121
CID000003152	CID000003261	CID000003305	CID000003308	CID000003339
CID000003342	CID000003386	CID000003394	CID000003403	CID000003404
CID000003410	CID000003446	CID000003449	CID000003454	CID000003475
CID000003648	CID000003672	CID000003702	CID000003793	CID000003825
CID000003826	CID000003878	CID000003883	CID000003899	CID000003929
CID000003937	CID000003957	CID000003961	CID000004044	CID000004054
CID000004075	CID000004158	CID000004205	CID000004259	CID000004409
CID000004440	CID000004449	CID000004583	CID000004585	CID000004614
CID000004635	CID000004679	CID000004691	CID000004745	CID000004775
CID000004819	CID000004856	CID000004885	CID000004920	CID000004932
CID000005002	CID000005029	CID000005040	CID000005070	CID000005073
CID000005076	CID000005077	CID000005078	CID000005090	CID000005095
CID000005195	CID000005203	CID000005210	CID000005212	CID000005245
CID000005267	CID000005291	CID000005352	CID000005358	CID000005372
CID000005379	CID000005401	CID000005402	CID000005466	CID000005508
CID000005514	CID000005523	CID000005525	CID000005538	CID000005596
CID000005625	CID000005645	CID000005656	CID000005718	CID000005719
CID000005731	CID000005732	CID000005734	CID000005735	CID000027661
CID000028112	CID000034312	CID000039042	CID000039860	CID000040976
CID000041317	CID000054454	CID000054547	CID000060613	CID000060787
CID000060795	CID000060852	CID000060953	CID000062959	CID000064147
CID000065999	CID000071158	CID000072938	CID000077993	CID000083786
CID000093860	CID000110634	CID000110635	CID000119607	CID000123606
CID000125889	CID000147912	CID000148192	CID000148211	CID000150610
CID000151165	CID000170361	CID000176870	CID000197712	CID000216239
CID000216326	CID003002190	CID003062316	CID003081884	CID004659568
CID005281104	CID005487301	CID005493381	CID006323497	CID009571074

(100) $\mathbb{S}_{100}$: 166 drug compounds having side effect “Abnormal vision”

CID000000450	CID000000564	CID000000807	CID000000937	CID000000942
CID000001690	CID000001972	CID000001978	CID000002156	CID000002162
CID000002182	CID000002216	CID000002267	CID000002269	CID000002284
CID000002369	CID000002375	CID000002405	CID000002435	CID000002462
CID000002512	CID000002520	CID000002578	CID000002585	CID000002678
CID000002726	CID000002751	CID000002764	CID000002769	CID000002771
CID000002794	CID000002801	CID000002909	CID000002958	CID000003015
CID000003016	CID000003032	CID000003066	CID000003121	CID000003148

CID000003152	CID000003157	CID000003203	CID000003261	CID000003279
CID000003285	CID000003339	CID000003345	CID000003355	CID000003373
CID000003386	CID000003403	CID000003404	CID000003410	CID000003419
CID000003446	CID000003454	CID000003475	CID000003510	CID000003519
CID000003648	CID000003672	CID000003724	CID000003736	CID000003746
CID000003749	CID000003793	CID000003826	CID000003878	CID000003883
CID000003911	CID000003937	CID000003948	CID000004158	CID000004159
CID000004236	CID000004259	CID000004409	CID000004449	CID000004473
CID000004485	CID000004493	CID000004542	CID000004583	CID000004585
CID000004594	CID000004609	CID000004634	CID000004635	CID000004679
CID000004691	CID000004739	CID000004745	CID000004819	CID000004889
CID000004893	CID000004920	CID000004932	CID000004943	CID000004946
CID000005002	CID000005005	CID000005029	CID000005040	CID000005073
CID000005076	CID000005077	CID000005095	CID000005195	CID000005203
CID000005210	CID000005212	CID000005358	CID000005372	CID000005379
CID000005394	CID000005401	CID000005430	CID000005466	CID000005478
CID000005512	CID000005514	CID000005516	CID000005523	CID000005538
CID000005596	CID000005636	CID000005645	CID000005656	CID000005665
CID000005718	CID000005719	CID000005732	CID000027661	CID000031477
CID000034312	CID000039860	CID000040976	CID000042615	CID000057537
CID000059768	CID000060184	CID000060612	CID000060613	CID000060754
CID000060953	CID000062959	CID000064147	CID000065999	CID000071158
CID000071616	CID000072054	CID000072938	CID000077992	CID000077993
CID000110634	CID000110635	CID000119607	CID000125889	CID000158440
CID003081884	CID004183806	CID005282044	CID005282226	CID005329102
CID006918453				
