# Supplementary material for: Predicting Drugs Side Effects Based on Chemical-Chemical Interactions and Protein-Chemical Interactions
Source: Biomed Res Int. 2013 Sep 4;2013:485034. doi: 10.1155/2013/485034 (PMC3776367; doi:10.1155/2013/485034)
Supplement: Supplementary file 2 [file 485034.f2.pdf]

Supplementary Material II: Breakdown of the benchmark dataset **S** using the 100 side effects

Tag	Side effect	Number of drugs
C_1	Nausea	703
C_2	Headache	663
C_3	Vomiting	652
C_4	Rash	604
C_5	Dizziness	588
C_6	Diarrhea	582
C_7	Pruritus	561
C_8	Urticaria	490
C_9	Fever	480
C_{10}	Edema	461
C_{11}	Abdominal pain	448
C_{12}	Somnolence	447
C_{13}	Pain	431
C_{14}	Constipation	431
C_{15}	Dyspnea	409
C_{16}	Anorexia	409
C_{17}	Insomnia	409
C_{18}	Thrombocytopenia	403
C_{19}	Paresthesia	393

C_{20}	Fatigue	385
C_{21}	Hypotension	378
C_{22}	Dyspepsia	360
C_{23}	Tachycardia	363
C_{24}	Hypersensitivity	361
C_{25}	Confusion	363
C_{26}	Leukopenia	360
C_{27}	Dry mouth	354
C_{28}	Cough	341
C_{29}	Asthenia	347
C_{30}	Arthralgia	344
C_{31}	Vertigo	337
C_{32}	Myalgia	331
C_{33}	Anxiety	334
C_{34}	Syncope	328
C_{35}	Hypertension	325
C_{36}	Anemia	326
C_{37}	Chest pain	322
C_{38}	Infection	299
C_{39}	Palpitations	302
C_{40}	Alopecia	308
C_{41}	Malaise	301

C_{42}	Allergic reaction	302
C_{43}	Back pain	292
C_{44}	Tremor	296
C_{45}	Nervousness	288
C_{46}	Hemorrhage	282
C_{47}	Arrhythmia	284
C_{48}	Tinnitus	276
C_{49}	Anaphylaxis	273
C_{50}	Blurred vision	276
C_{51}	Flatulence	268
C_{52}	Flushing	266
C_{53}	Pharyngitis	259
C_{54}	Angioedema	262
C_{55}	Jaundice	260
C_{56}	Rhinitis	257
C_{57}	Erythema	252
C_{58}	Agitation	256
C_{59}	Hepatitis	241
C_{60}	Sweating	243
C_{61}	Influenza	236
C_{62}	Seizures	241
C_{63}	Sinusitis	225

C_{64}	Hallucinations	232
C_{65}	Bradycardia	225
C_{66}	Hematuria	220
C_{67}	Conjunctivitis	221
C_{68}	Agranulocytosis	228
C_{69}	Weakness	227
C_{70}	Chills	219
C_{71}	Peripheral edema	208
C_{72}	Epistaxis	216
C_{73}	Photosensitivity	211
C_{74}	Hyperglycemia	204
C_{75}	Pneumonia	201
C_{76}	Myocardial infarction	199
C_{77}	Pancreatitis	199
C_{78}	Neutropenia	199
C_{79}	Weight gain	204
C_{80}	Stomatitis	194
C_{81}	Stevens - Johnson syndrome	196
C_{82}	Impotence	195
C_{83}	Eosinophilia	196
C_{84}	Urinary tract infection	186
C_{85}	Purpura	192

C_{86}	Increased sweating	184
C_{87}	Dysuria	181
C_{88}	Bronchitis	184
C_{89}	Dermatitis	188
C_{90}	Angina pectoris	181
C_{91}	Asthma	180
C_{92}	Urinary frequency	178
C_{93}	Upper respiratory tract infection	176
C_{94}	Ataxia	182
C_{95}	Dysphagia	177
C_{96}	Convulsions	179
C_{97}	Weight loss	173
C_{98}	Erythema multiforme	175
C_{99}	Gastritis	170
C_{100}	Abnormal vision	166
Total	----	30,114
