# Supplementary material for: Predicting Drugs Side Effects Based on Chemical-Chemical Interactions and Protein-Chemical Interactions
Source: Biomed Res Int. 2013 Sep 4;2013:485034. doi: 10.1155/2013/485034 (PMC3776367; doi:10.1155/2013/485034)
Supplement: Supplementary file 3 [file 485034.f3.pdf]

Supplementary Material III: The prediction accuracies of the interaction-based and similarity-based method in identifying drugs side effects.

Prediction order	Interaction-based		Similarity-based	
	Training dataset	Test dataset	Training dataset	Test dataset
1	0.863032	0.891566	0.836436	0.879518
2	0.804521	0.831325	0.791223	0.831325
3	0.771277	0.843373	0.75	0.795181
4	0.726064	0.819277	0.714096	0.759036
5	0.734043	0.771084	0.682181	0.746988
6	0.6875	0.759036	0.668883	0.710843
7	0.676862	0.674699	0.647606	0.578313
8	0.642287	0.650602	0.599734	0.650602
9	0.636968	0.686747	0.587766	0.578313
10	0.577128	0.578313	0.573138	0.60241
11	0.591755	0.60241	0.56383	0.674699
12	0.591755	0.698795	0.566489	0.518072
13	0.573138	0.614458	0.547872	0.53012
14	0.558511	0.590361	0.538564	0.626506
15	0.541223	0.542169	0.506649	0.578313
16	0.538564	0.590361	0.526596	0.554217
17	0.513298	0.39759	0.5	0.60241
18	0.545213	0.626506	0.517287	0.53012

19	0.5	0.566265	0.5	0.385542
20	0.472074	0.445783	0.477394	0.518072
21	0.477394	0.457831	0.456117	0.578313
22	0.510638	0.506024	0.482713	0.566265
23	0.472074	0.46988	0.478723	0.518072
24	0.441489	0.481928	0.472074	0.481928
25	0.469415	0.542169	0.442819	0.506024
26	0.428191	0.554217	0.452128	0.421687
27	0.444149	0.53012	0.461436	0.421687
28	0.444149	0.481928	0.457447	0.421687
29	0.402926	0.493976	0.412234	0.46988
30	0.410904	0.493976	0.412234	0.481928
31	0.421543	0.433735	0.425532	0.493976
32	0.385638	0.421687	0.404255	0.421687
33	0.404255	0.39759	0.400266	0.457831
34	0.378989	0.39759	0.412234	0.493976
35	0.357713	0.421687	0.396277	0.46988
36	0.361702	0.349398	0.344415	0.409639
37	0.393617	0.421687	0.371011	0.457831
38	0.353723	0.337349	0.37367	0.421687
39	0.381649	0.361446	0.382979	0.373494
40	0.359043	0.39759	0.347074	0.506024

41	0.327128	0.337349	0.336436	0.421687
42	0.337766	0.385542	0.357713	0.349398
43	0.337766	0.409639	0.382979	0.409639
44	0.308511	0.337349	0.324468	0.409639
45	0.333777	0.373494	0.356383	0.409639
46	0.344415	0.337349	0.329787	0.39759
47	0.328457	0.313253	0.351064	0.301205
48	0.301862	0.313253	0.3125	0.445783
49	0.305851	0.26506	0.347074	0.325301
50	0.31117	0.349398	0.324468	0.313253
51	0.300532	0.325301	0.325798	0.26506
52	0.284574	0.349398	0.329787	0.361446
53	0.303191	0.325301	0.307181	0.277108
54	0.288564	0.349398	0.331117	0.277108
55	0.271277	0.337349	0.303191	0.385542
56	0.259309	0.301205	0.297872	0.277108
57	0.261968	0.26506	0.31117	0.277108
58	0.272606	0.277108	0.291223	0.289157
59	0.275266	0.192771	0.304521	0.325301
60	0.232713	0.301205	0.268617	0.228916
61	0.231383	0.228916	0.287234	0.289157
62	0.25266	0.289157	0.261968	0.277108

63	0.244681	0.228916	0.292553	0.313253
64	0.269947	0.180723	0.239362	0.325301
65	0.242021	0.216867	0.295213	0.253012
66	0.236702	0.228916	0.259309	0.204819
67	0.24734	0.228916	0.253989	0.277108
68	0.210106	0.168675	0.235372	0.192771
69	0.196809	0.240964	0.234043	0.277108
70	0.224734	0.240964	0.261968	0.180723
71	0.214096	0.253012	0.24867	0.240964
72	0.222074	0.216867	0.234043	0.301205
73	0.191489	0.216867	0.243351	0.253012
74	0.199468	0.240964	0.244681	0.180723
75	0.214096	0.277108	0.240691	0.253012
76	0.19016	0.228916	0.238032	0.216867
77	0.191489	0.168675	0.227394	0.192771
78	0.171543	0.277108	0.214096	0.192771
79	0.172872	0.13253	0.239362	0.277108
80	0.183511	0.228916	0.234043	0.253012
81	0.154255	0.180723	0.224734	0.240964
82	0.155585	0.240964	0.199468	0.192771
83	0.144947	0.180723	0.216755	0.228916
84	0.144947	0.240964	0.236702	0.253012

85	0.178191	0.156627	0.223404	0.289157
86	0.162234	0.156627	0.206117	0.168675
87	0.166223	0.084337	0.199468	0.228916
88	0.136968	0.13253	0.199468	0.228916
89	0.12633	0.13253	0.207447	0.240964
90	0.140957	0.168675	0.208777	0.192771
91	0.12766	0.144578	0.206117	0.168675
92	0.12234	0.13253	0.199468	0.180723
93	0.119681	0.156627	0.208777	0.204819
94	0.117021	0.168675	0.191489	0.144578
95	0.107713	0.13253	0.182181	0.204819
96	0.103723	0.108434	0.171543	0.144578
97	0.105053	0.120482	0.180851	0.144578
98	0.105053	0.108434	0.167553	0.156627
99	0.082447	0.060241	0.158245	0.156627
100	0.055851	0.060241	0.136968	0.108434
